# Supplementary material for: Impaired eIF5A function causes a Mendelian disorder that is partially rescued in model systems by spermidine
Source: Nat Commun. 2021 Feb 5;12:833. doi: 10.1038/s41467-021-21053-2 (PMC7864902; doi:10.1038/s41467-021-21053-2)
Supplement: Supplementary file 1 — Supplementary Information [file 41467_2021_21053_MOESM1_ESM.pdf]

Supplementary Information

Impaired eIF5A function causes a Mendelian disorder that is partially rescued in model systems by spermidine

Víctor Faundes, Martin D. Jennings, Siobhan Crilly, Sarah Legraie, Sarah E. Withers, Sara Cuvertino, Sally J. Davies, Andrew G.L. Douglas, Andrew E. Fry, Victoria Harrison, Jeanne Amiel, Daphné Lehalle, William G. Newman, Patricia Newkirk, Judith Ranells, Miranda Splitt, Laura Cross, Carol J. Saunders, Bonnie R Sullivan, Jorge L. Granadillo, Christopher T. Gordon, Paul R. Kasher, Graham D. Pavitt, Siddharth Banka

Supplementary Note 1

Case reports from individuals described in Table 1

Individual 1; *de novo*, c.143C>A, p.T48N
This is a 6.9-year-old female born to non-consanguineous, healthy parents. She had congenital microcephaly (head circumference of 31 cm at 1 month old) and intra-uterine growth retardation (birth weight 2260 g). She has moderate to severe global developmental delay. She walked independently from 24 months of age, and speaks in short sentences with difficult articulation at 6.9 years of age. She has abnormal mineralisation of the skull, bilateral ethmoidal, zygomatic, malar and mandibular hypoplasia, defects of the orbital floor, and hemivertebrae (L3). She does not have cardiac anomalies. She has eyelid hypoplasia, corneal ulceration and strabismus. At 6.8 years of age her height was 119 cm (0.5 SD), weight was 25.8 Kg (2 SD) and head circumference was 46 cm (-3 SD). She has sloping forehead, broad eyebrows, small lower eyelids, hypoplastic supraorbital ridges, bulbous nasal tip, microretrognathia, high arched palate, abnormal pinnae, low set ears and short neck. Hands and feet are also short with hypoplastic thumbs with limited metacarpophalangeal flexion, clinodactyly of 5th fingers and brachydactyly of toes. The initial clinical suspicion was a syndromic mandibulofacial dysostosis with microcephaly.

Individual 2; *de novo*, c.316G>A, p.G106R
This is an 8.4-year-old female born to consanguineous, healthy parents, without other affected individuals in her family. She had intra-uterine growth retardation, congenital microcephaly and neonatal feeding difficulties. She had a normal computed tomography scan at birth. She was also noted to have cardiac anomalies prenatally. She had a dysplastic tricuspid valve, tricuspid regurgitations and secundum atrial septal defect that were treated surgically at 11 months of age. She has moderate global developmental delay. She could sit unsupported at 18 months, walked independently from 23 months of age, and said her first words from 2.5 years of age. She is fed via a gastrostomy tube. She has constipation and gastroesophageal reflux. At 6.7 years of age her height was 105.3 cm (-2.82 SD), weight was 16.4 Kg (-2.28 SD) and head circumference was 43.5 cm (-7.47 SD). She has broad eyebrows, hypoplastic supraorbital ridges, epi- and telecanthus, bulbous nasal tip, thin upper lip, micrognathia and low set ears. The initial clinical suspicion was a Kabuki-like syndrome.

Individual 3; *de novo*, c.324dupA, p.R109TfsTer8
This is an 8.4-year-old female born to non-consanguineous, healthy parents. She had congenital microcephaly, a cleft palate, and neonatal feeding difficulties. The cleft palate was corrected surgically. She was also noted to have cardiac anomalies neonatally. She had a functional bicuspid aortic valve with fusion of the left and right coronary cusps, and trace aortic regurgitation. However, by 3 years of age her heart scan showed a structurally and functionally normal heart with a trileaflet aortic valve. She had mild global developmental delay and autistic traits. She walked independently from 17 months of age and said her first word from 12 months of age. She has conductive deafness that required grommets, premature thelarche and joint hypermobility. No ophthalmological, urogenital, immunity or brain anomalies have been detected. At 26 months of age her height was 90.3 cm (0.86 SD), weight was 11.3 Kg (-0.41 SD) and head circumference was 44.5 cm (-2.11 SD). She has broad eyebrows, hypoplastic supraorbital ridges, epi- and telecanthus, hypertelorism, bulbous nasal tip, thin upper lip and micrognathia. The initial clinical suspicion was a Kabuki-like syndrome.

Individual 4; *de novo*, c.325C>G, p.R109G
This is an 18.3-year-old male born to non-consanguineous, healthy parents. He had intra-uterine growth retardation, neonatal hypotonia, and neonatal feeding difficulties. He had mild global developmental delay. He could sit unsupported at 1 year of age, walked independently from 1.5 years of age, and said his first words from 12 months of age. He has an IQ level of 50. He has small bilateral high intensity signals in the peritrigonal regions of the deep frontoparietal white matter. He is not known to have cardiac anomalies, but has not had any cardiac imaging. He has constipation, glaucoma, cryptorchidism and joint hypermobility. At 17.4 years of age his height was 172.5 cm (-0.45 SD), weight was 66.4 Kg (0.06 SD) and head circumference was 52.2 cm (-2.62 SD). He has broad eyebrows, prominent supraorbital ridges, epicanthus, long eyelashes, malar hypoplasia, prominent ears, bulbous nasal tip, thin upper lip, micrognathia, pes planus, and sandal gap. The initial clinical suspicion was a Kabuki-like syndrome.

Individual 5; *de novo*, c.325C>T, p.R109*
This is an eight-month-old male born to non-consanguineous, healthy parents. The pregnancy was complicated by maternal hypertension and early pre-eclampsia and polyhydramnios. He was delivered at 35 weeks 4 days gestation and weighed 3.20Kg. He initially required positive pressure ventilation for poor respiratory. He

required feeding via a naso gastric tube feeding and a gastrostomy tube was placed at four months of age due to continued poor growth and oral aversion. He has developmental delays with minimal babbling and poor head control at eight months of age. He could roll from belly to back but could not sit unassisted. He has an atrial septal defect, moderate right atrial enlargement and mild right ventricular dilation. He has been diagnosed with dysphagia and failure to thrive. At eight months of age, his length was 68 cm (-1.45 SD), weight was 6.18 Kg (-3.14 SD) and head circumference was 43 cm (-0.45 SD). Dysmorphic features noted on physical exam included plagiocephaly, sparse/furry scalp hair, frontal bossing, epicanthal folds, sparse eyebrows, downslanting palpebral fissures, cupped and prominent ears, an ear lobe crease on the right ear, a thin upper lip, and paucity of subcutaneous fat. Physical exam also noted hypotonia with a significant head lag and the inability to sit independently. His initial clinical suspicion was Mowat-Wilson-like syndrome

Individual 6; *de novo*, c.343 C>T; p.P115S
This is a 4-years 8 month-old male born to non-consanguineous, healthy parents. He had no prenatal and neonatal anomalies. He has global developmental delay. He walked independently from 18 months of age, and said his first words at 2 years of age. He spoke in full sentences at almost 4 years. He has no major malformations. He has mild diffuse hypotonia, esophoria, anxiety-induced tic. At 4 years 8 months of age his height was 111.7 cm (+1 SD), weight was 17.6 Kg (-0.09 SD) and head circumference was 49 cm (-1.09 SD). On physical exam, he was found to be thin. He had long palpebral fissures (length at the 90th percentile), broad eyebrows and protruding ears bilaterally. He also had micrognathia, a high-arched palate, a long nasal bridge with small ala nasi. Flat feet were noticed bilaterally, with talus pronation.

Individual 7; *de novo*, c.364G>A, p.E122K
This is a 16.4-year-old female born to non-consanguineous, healthy parents. She had foetal ascites, which spontaneously resolved prenatally, and positional talipes. She had moderate global developmental delay. She could sit unsupported at 9 months, walked independently from 3 years of age, and said her first words from 3 years of age. She has also autism spectrum disorder and attention deficit hyperactivity disorder. She does not have brain anomalies. She is not known to have cardiac anomalies, but has not had any cardiac imaging. She has left convergent strabismus, nasal polyps, and delayed puberty. At 16 years of age her height was 147.6 cm (-2.59 SD), weight was 61.95 Kg (-0.69 SD) and head circumference was 52.7 cm (-1.94 SD). She has epicanthus, deep-set eyes, prominent creasing and lacrimal punctae of the lower eyelids, small low-set ears, unilateral single palmar crease, small 4th and 5th toenails, contractures of the 5th toes and pes planus. The initial clinical suspicion was of an unidentified genetic syndrome.

Supplementary Note 2

*h*eIF5A-*WT gene sequence*

ggCTCGAGtctgaggggtgagaacagcaaaacgggaacggcgacttggagtagtataacgctgtagctggacgct
gaggggccaaggccggtaaaattctgcaacacccagaccttgctgcagtgatatcatccacccatacccctcgat
gtattccgtagcgttatatcgggtccgtgtgaggcgacaagggacctcccgcaccgcacataccgcaggagctaa
aagaaacgccgtcgcccgaaaaaaagtttcgaaggtgaaggaacagtggtaataaaaatttttttcgtcgtcgtc
gtcgatcggcttttggccgcgttgatcgtcgtgttccacgattatataatgcacaaggtttttgccatatcttga
cattggaatccctcatttcctaaattcctctccttctatcaattgcttcctttccttctttctaacgattctact
tctgtagccaattactcatagactcccaaacacacacaaataccaactcatatatacaATGGCTGACGACTTGGA
CTTTGAAACTGGTGACGCTGGTGCCTCCGCCACCTTCCCAATGCAATGTTCTGCCTTGAGAAAGAACGGTTTCGT
TGTCTTGAAGGGTAGACCATGTAAGATTGTCGAAATGTCCACTTCTAAGACTGGTAAGCACGGTCACGCTAAAGT
CCATTTGGTTGGTATTGATATCTTCACTGGTAAGAAGTATGAAGATATTTGTCCATCTACTCACAACATGGATGT
TCCAAATATTAAGAGAAACGATTTCCAATTGATTGGTATTCAAGACGGTTACTTGTCTTTGTTGCAAGATTCTGG
TGAAGTTAGAGAGGACCTTCGTCTCCCTGAAGGTGATTTGGGTAAGGAAATTGAACAGAAGTATGATTGTGGTGA
AGAAATTTTGATTACCGTCTTGTCCGCTATGACTGAAGAAGCCGCCGTCGCCATCAAGGCAATGGCTAAGTAAac
cggttaacatcatggcatgggatataaatgaaaaagaaaaaaaaactccgacgccccttccatcacatcatgta
ctcttcgctgaaccgggtttttttctttgcaattttttttttcgttctcctaaagcatacacaaataaatcctttt
ttttattttctatttattttgttatttatcatctatatagcaataatatactttgtttttattcgtatttcacac
ttttctttttccttatgcaggcagtgtaattcattggggaggatgattttcatgtgcgcatatctaccggctgca
agcagccggtcggtggcaaatccggcgcttccccctcaaaaaaaaaaaaaaaaaaaaaaaaagggaactctcaAC
TAGTgg

Color key:

Additional GG, Xho1 restriction site, yeast 5’-500 bases, human *EIF5A* ORF optimised for yeast codon usage, yeast 3’-375 bases, Spe1 restriction site.

Supplementary Table 1. List of plasmids used in this study.

Plasmid	Precursor	Description	Source
pAV1360	YCplac111	sc *LEU2*	Rizzardi, et al. [1]
pAV2565	pC3294	sc *LEU2 y*eIF5A-S149P	Saini, et al. [2]
pAV2566	pC4351	hc URA3 *GAL1* promoter-*LDB17-His6-HA-3C-ZZ*	Gutierrez, et al. [3]
pAV2569	pC3287	sc *LEU2 y*eIF5A	Saini, et al. [2]
pAV2570	pC4353	hc URA3 *GAL1* promoter-*EAP1-His6-HA-3C-ZZ*	Gutierrez, et al. [3]
pAV2571	pC3288	sc *URA3 y*eIF5A	Saini, et al. [2]
pAV2578	pUC19	h*EIF5A*	This study
pAV2580	pAV1360 and pAV2578	sc *LEU2 h*eIF5A	This study
pAV2584	pAV2580	sc *LEU2 h*eIF5A-T48N	This study
pAV2585	pAV2580	sc *LEU2 h*eIF5A-G106R	This study
pAV2586	pAV2580	sc *LEU2 h*eIF5A-R109Tfs*8	This study
pAV2587	pAV2580	sc *LEU2 h*eIF5A-E122K	This study
pAV2590	pAV2582	hc LEU2 *h*eIF5A-R109Tfs*8	This study
pAV2592	pAV1361 and pAV2578	sc URA3 *h*eIF5A	This study

Abbreviations used: *h*:human (human protein sequence with yeast optimised codon usage), hc: high copy number (2 micron) plasmid, sc: single copy number (centromeric) plasmid, *y*: yeast.

Supplementary Table 2. Sequences of primers used for site-directed mutagenesis.

**Variant[a] (CDS; Protein)**	**Direction**	**Primer (5'-3')**
c.143C>A; p.T48N	Forward	CCACTTCTAAGAATGGTAAGCACGGTCACGCTAAAGTCC
	Reverse	CCGTGCTTACCATTCTTAGAAGTGGACATTTCGACAATCTTAC
c.316G>A; p.G106R	Forward	GCAAGATTCTCGTGAAGTTAGAGAGGACCTTCGTC
	Reverse	CTCTAACTTCACGAGAATCTTGCAACAAAGACAAGTAACCG
c.324dupA[b]; p.R109Tfs*8	Forward	GGTGAAGTTACGAGAGGACCTTCGTCTCCCTG
	Reverse	AGGTCCTCTCGTAACTTCACCAGAATCTTGCAACAAAG
c.364G>A; p.E122K	Forward	GTGATTTGGGTAAGAAAATTGAACAGAAGTATGATTGTGGTGAAG
	Reverse	CTGTTCAATTTTCTTACCCAAATCACCTTCAGGGAGACG

[a] As detected in individuals

[b] For optimising the translation according to yeast codon usage but keeping the predicted truncated amino acid sequence of *EIF5A* seen in individual 3, the dupA was replaced by insC

Abbreviation: CDS=Coding sequence

Supplementary Table 3. List of yeast strains created and used in this study.

Strain	Precursor	Genotype[a]
J696		pAV2571(pC3288)[*y*eIF5A*URA3*]
GP7439	J696	pAV2571[*y*eIF5A *URA3*] pAV2569 [*y*eIF5A *LEU2*]
GP7440	GP7439	pAV2569[*y*eIF5A *LEU2*]
GP7441	J696	pAV2571[*y*eIF5A *URA3*] pAV2580[*h*eIF5A*LEU2*]
GP7443	J696	pAV2571[*y*eIF5A *URA3*] pAV2565[*y*eIF5A-S149P *LEU2*]
GP7444	GP7441	pAV2580[*h*eIF5A *LEU2*]
GP7446	GP7443	pAV2565[*y*eIF5A-S149P *LEU2*]
GP7447	J696	pAV2571[*y*eIF5A *URA3*] pAV2584[*h*eIF5A-T48N *LEU2*]
GP7448	J696	pAV2571[*y*eIF5A *URA3*] pAV2585[*h*eIF5A-G106R, *LEU2*]
GP7449	J696	pAV2571[*y*eIF5A *URA3*] pAV2586[*h*eIF5A-R109Tfs*8 *LEU2*]
GP7450	J696	pAV2571[*y*eIF5A *URA3*] pAV2587[*h*eIF5A-E122K *LEU2*]
GP7455	GP7447	pAV2584[*h*eIF5A-T48N *LEU2*]
GP7456	GP7450	pAV2587[*h*eIF5A-E122K *LEU2*]
GP7469	GP7444	pAV2580[*h*eIF5A *LEU2*] pAV2592[*h*eIF5A *URA3*]
GP7474	GP7448	pAV2585[*h*eIF5A-G106R, *LEU2*]
GP7480	GP7469	pAV2592[*h*eIF5A *URA3*]
GP7482	GP7480	pAV2592[*h*eIF5A *URA3*] pAV2586[*h*eIF5A-R109Tfs*8 LEU2]
GP7484	GP7480	pAV2592[*h*eIF5A *URA3*] YCplac111[*LEU2*]
GP7485	GP7480	pAV2592[*h*eIF5A *URA3*] pAV2590[*h*eIF5A-R109Tfs*8 *LEU2*]
GP7490	GP7444	pAV2580[*h*eIF5A *LEU2*] pAV2566[*GAL1* promoter-LDB17-*His6-HA-3C-ZZ URA3*]
GP7491	GP7455	pAV2584[*h*eIF5A-T48N *LEU2*] pAV2566[*GAL1* promoter-LDB17-*His6-HA-3C-ZZ URA3*]
GP7492	GP7474	pAV2585[*h*eIF5A-G106R, *LEU2*] pAV2566[*GAL1* promoter-LDB17-*His6-HA-3C-ZZ URA3*]
GP7493	GP7456	pAV2587[*h*eIF5A-E122K *LEU2*] pAV2566[*GAL1* promoter-LDB17-*His6-HA-3C-ZZ URA3*]
GP7500	GP7444	pAV2580[*h*eIF5A *LEU2*] pAV2570[*GAL1* promoter-*EAP1-His6-HA-3C-ZZ URA3*]
GP7501	GP7455	pAV2584[*h*eIF5A-T48N *LEU2*] pAV2570[*GAL1* promoter-*EAP1-His6-HA-3C-ZZ URA3*]
GP7502	GP7474	pAV2585[*h*eIF5A-G106R, *LEU2*] pAV2570[*GAL1* promoter-*EAP1-His6-HA-3C-ZZ URA3*]
GP7503	GP7456	pAV2587[*h*eIF5A-E122K *LEU2*] pAV2570[*GAL1* promoter-*EAP1-His6-HA-3C-ZZ URA3*]

[a] The genotype for all yeast strains is *MATα trp1-63 ura3-52 leu2-3 leu2-112 GAL2+ gcn2Δ tif51b::NAT tif51a::KANMX4* from Saini, et al. [2] but with the specific plasmids detailed above. All strains made for this study except J696

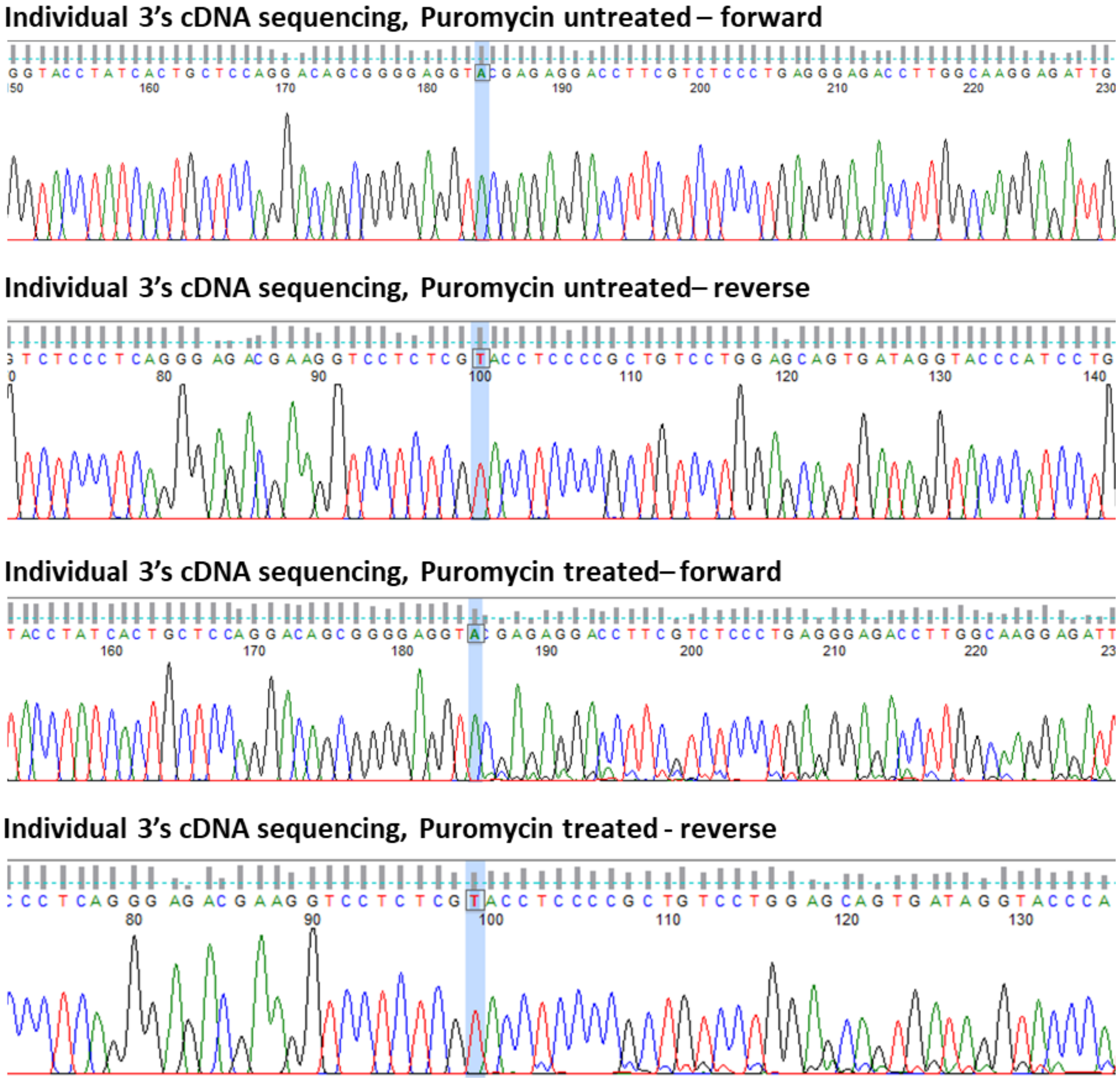

Supplementary Figure 1. Individual 3 cDNA Sequencing.

Lymphoblastoid cells from Individual 3 were harvested either after 6-hrs-treatment with puromycin (200µg/mL) or not, washed in PBS and then kept at -80°C. After standard reverse transcription, cDNA from both conditions were Sanger sequenced bidirectionally. While cDNA sequences from puromycin untreated cells only show the WT allele, the cDNA sequences from puromycin treated cells show low peaks for other nucleotides compatible with the frameshift allele

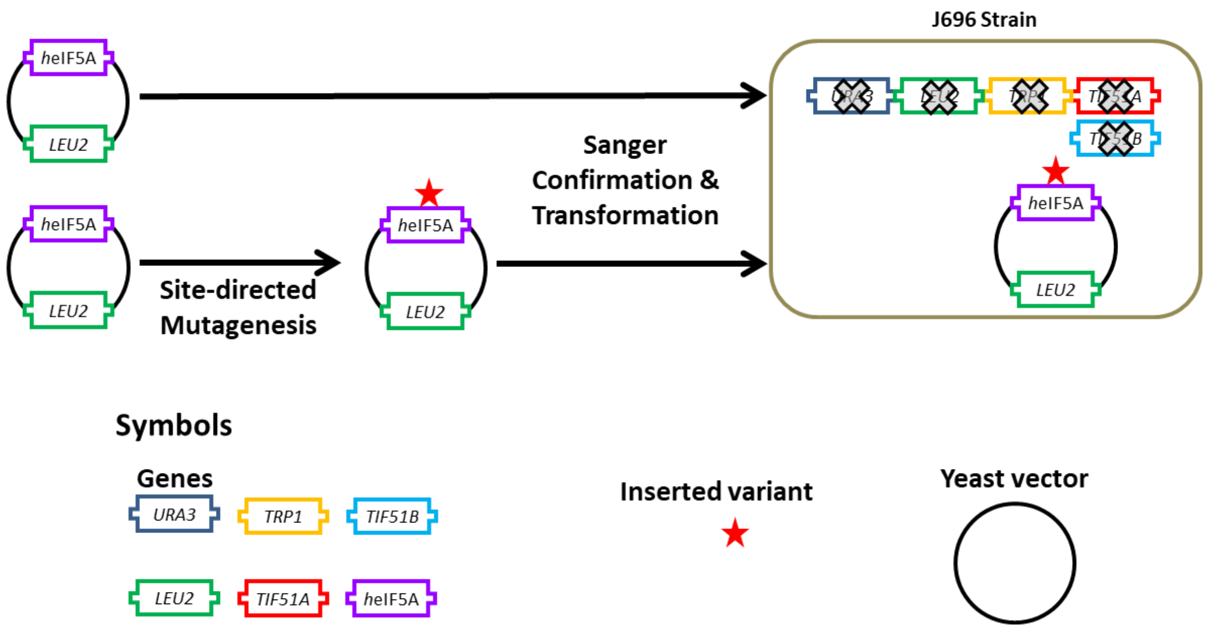

Supplementary Figure 2. Summary of the yeast strain construction.

A yeast codon and expression optimised h*EIF5A* cDNA was synthesised and cloned into yeast plasmid vectors, which were subsequently subject to site-directed mutagenesis. The J696 strain, with both *TIF51A* and *TIF51B* genes deleted, was transformed with the indicated vectors to create strains for this study.

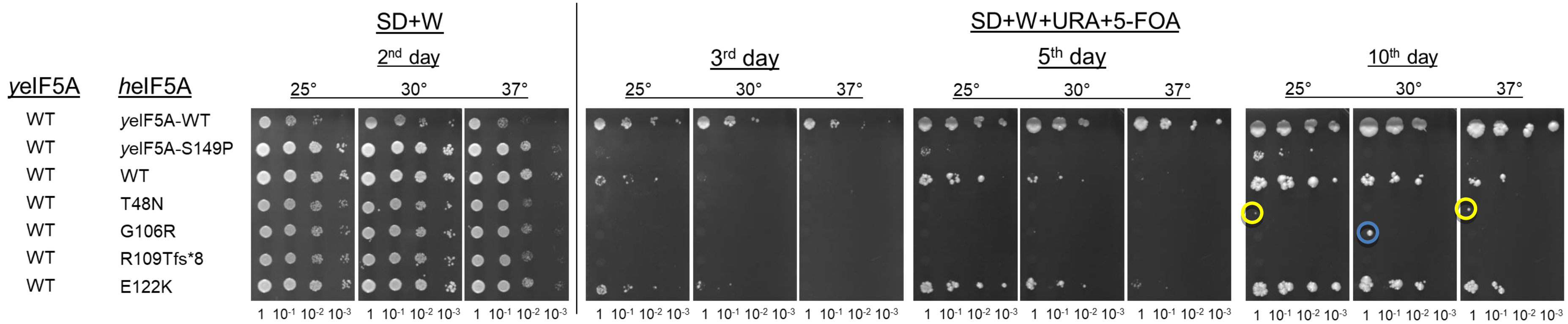

Supplementary Figure 3. Growth of human eIF5A alleles in yeast.

Growth of strains with indicated eIF5A alleles on synthetic dextrose (SD) plus Tryptophan (+W) (left) and SD+W+ Uracil (URA) + 5-Fluoroorotic acid (5-FOA) incubated for 2, 3, 5 and 10 days at 25°C, 30°C and 37°C (right). Small colonies with *h*eIF5A p.T48N only (yellow circles) and p.G106R only (blue circle) were detected after 10 days. Eighteen replicates per temperature per condition were performed.

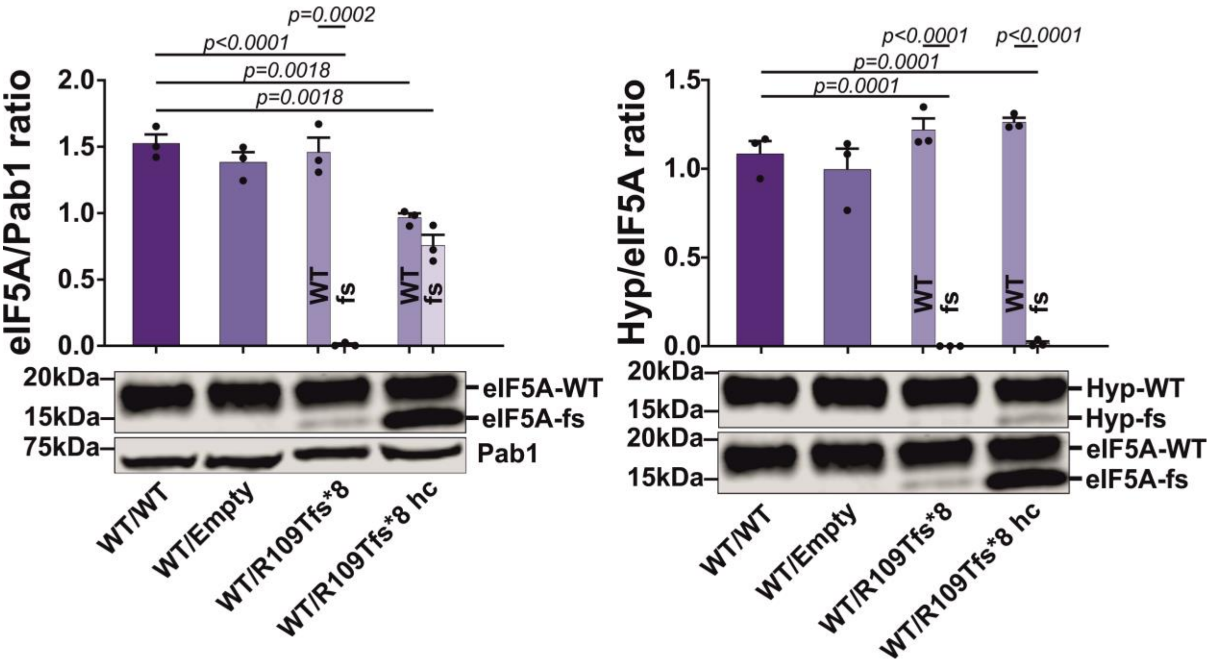

Supplementary Figure 4. Expression and hypusination of eIF5A-R109Tfs*8 in strains maintained with WT *h*eIF5A.

Immunoblots showing eIF5A expression (left) and hypusination (Hyp; right) comparing *h*eIF5A-R109Tfs*8 from single copy or high-copy (hc) plasmids co-expressed with *h*eIF5A-WT. WT only controls are also shown. Cells were grown at 30°C in SCD-URA-LEU liquid medium. Data presentation and statistical treatment as described for Fig. 2b. Where 2 alleles are expressed their levels were quantified separately as indicated by the labelled half-width bars. Full uncropped images of gel blots are shown in Supplementary Figure 9.

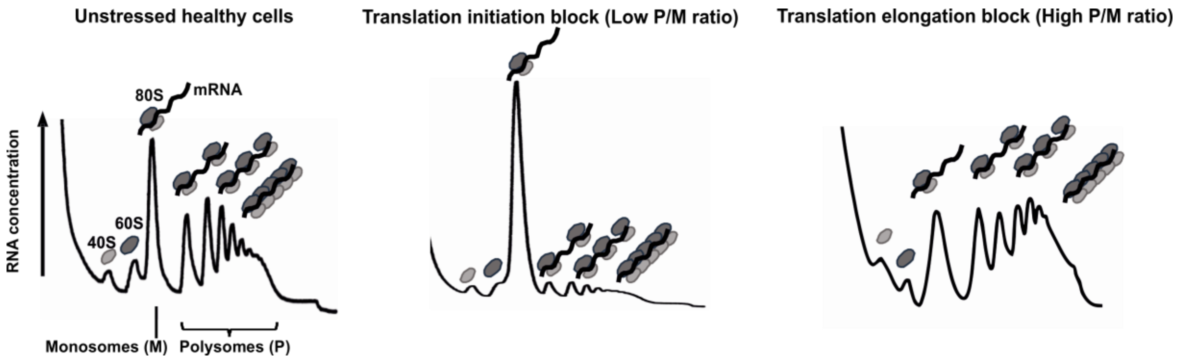

Supplementary Figure 5. Possible results for polysome profiling of eIF5A variants

Under normal conditions (left panel), cells exhibit a Polysome-to-Monosome (P/M) ratio slightly above 1, and a low 60S/80S ratio. When there is a defect in translation initiation (middle panel), both the P/M and 60S/80S will be very low, whereas in translation elongation defects (right panel), the P/M ratio will be high and the 60S/80S ratio will be low. While the former situation would reflect a loss of interaction of eIF5A with the ribosome, the latter would reflect an accumulation of eIF5A in the ribosome.

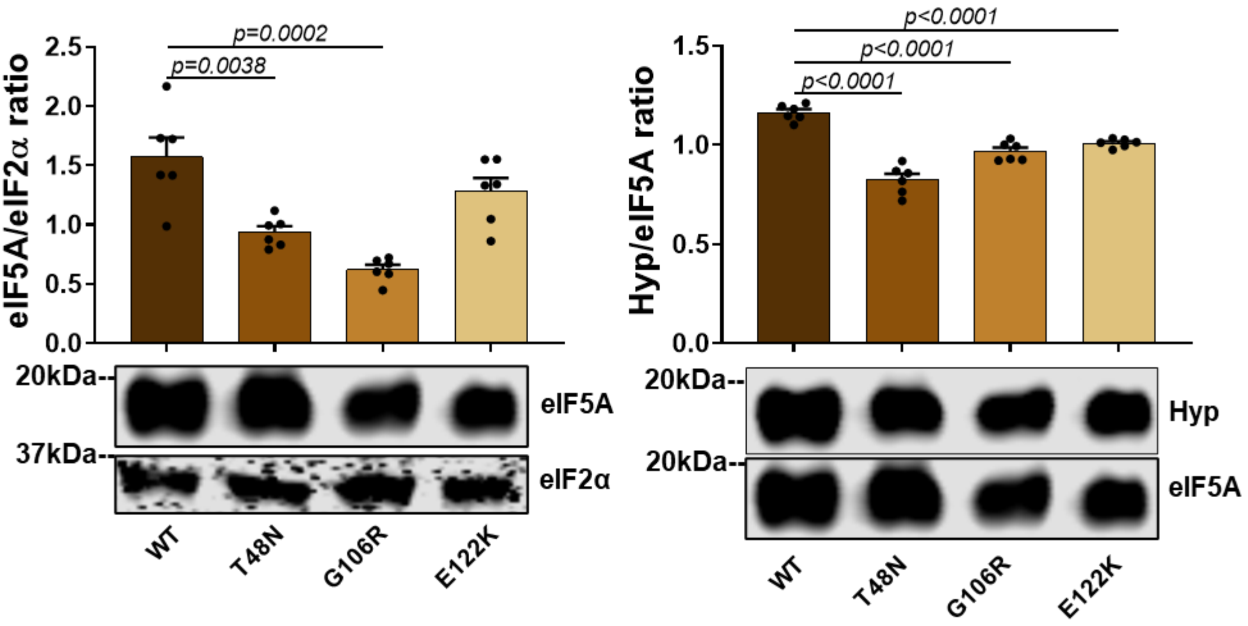

Supplementary Figure 6. Expression and hypusination of eIF5A alleles in Ldb17-HA PPT reporter strains.

eIF5A expression (left) and hypusination (Hyp; right) amongst yeast strains haploid for *h*eIF5A with the Haemagglutinin (HA)-tagged Ldb17 reporter, grown at 30°C in SCGal medium. Data presentation and statistical treatment as described for Fig. 2b. Full uncropped images of gel blots are shown in Supplementary Figure 14.

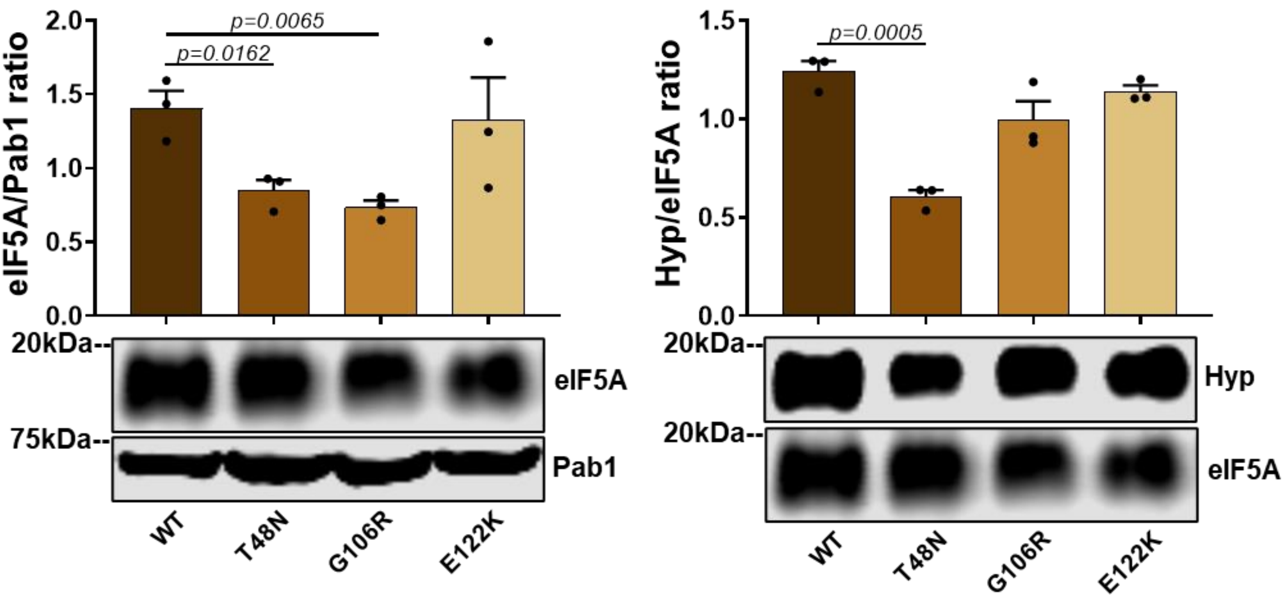

Supplementary Figure 7. Expression and hypusination of eIF5A alleles in Eap1-HA PPT reporter strains.

eIF5A expression (left) and hypusination (Hyp; right) amongst yeast strains haploid for *h*eIF5A with the Haemagglutinin (HA)-tagged Eap1 reporter, grown at 30°C in SCGal medium. Data presentation and statistical treatment as described for Fig. 2b. Full uncropped images of gel blots are shown in Supplementary Figure 15.

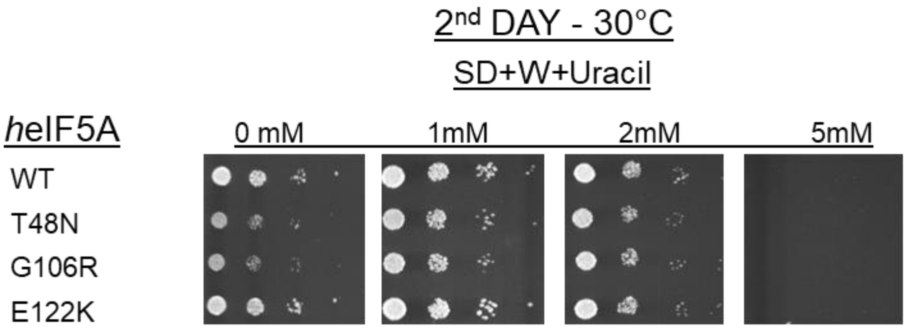

Supplementary Figure 8. Extended Figure 3a. Growth of Yeast strains with Spermidine

Growth of yeast strains haploid for human eIF5A (*h*eIF5A) or its variants in minimum media supplemented with 0 mM (Untreated), 1mM, 2mM and 5mM of spermidine at 30°C until day 2. Eighteen replicates per condition were performed.

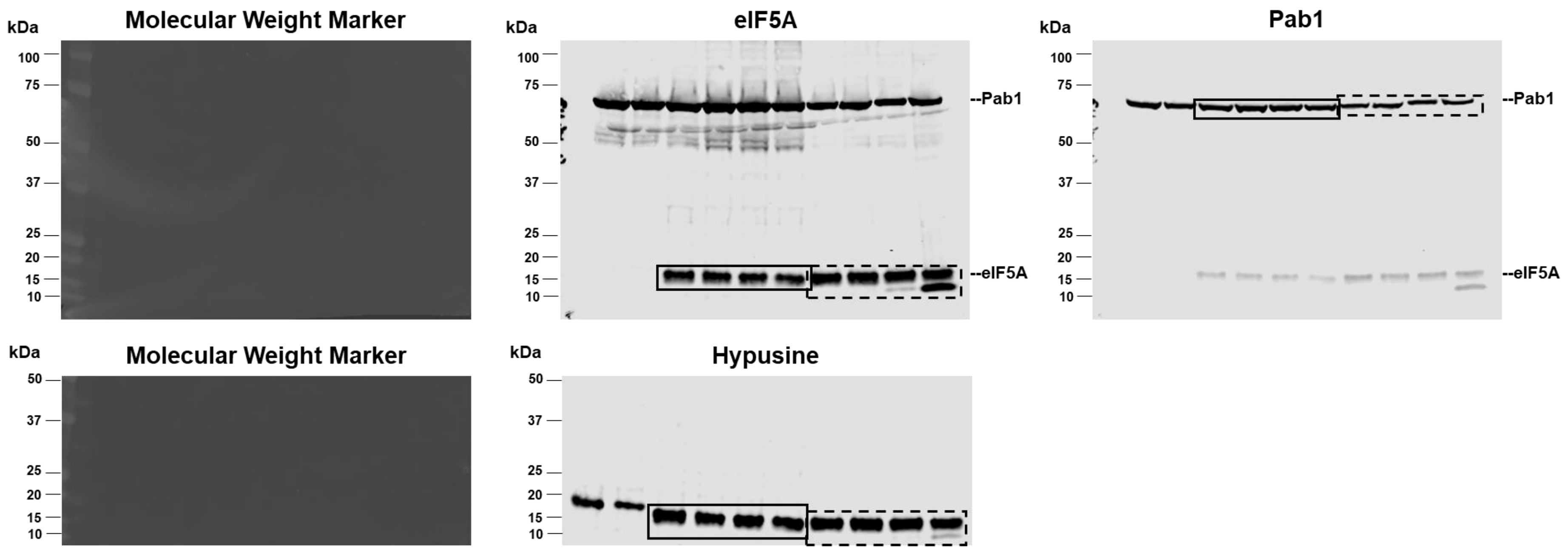

Supplementary Figure 9. Full scans of western blots shown in Fig 2b and Supplementary Fig 4.

Scans of molecular weight marker, eIF5A, Hypusine and loading control (Pab1). The black boxes and black dashed-line boxes depict the areas shown in Fig. 2b and in Supplementary Fig 4, respectively.

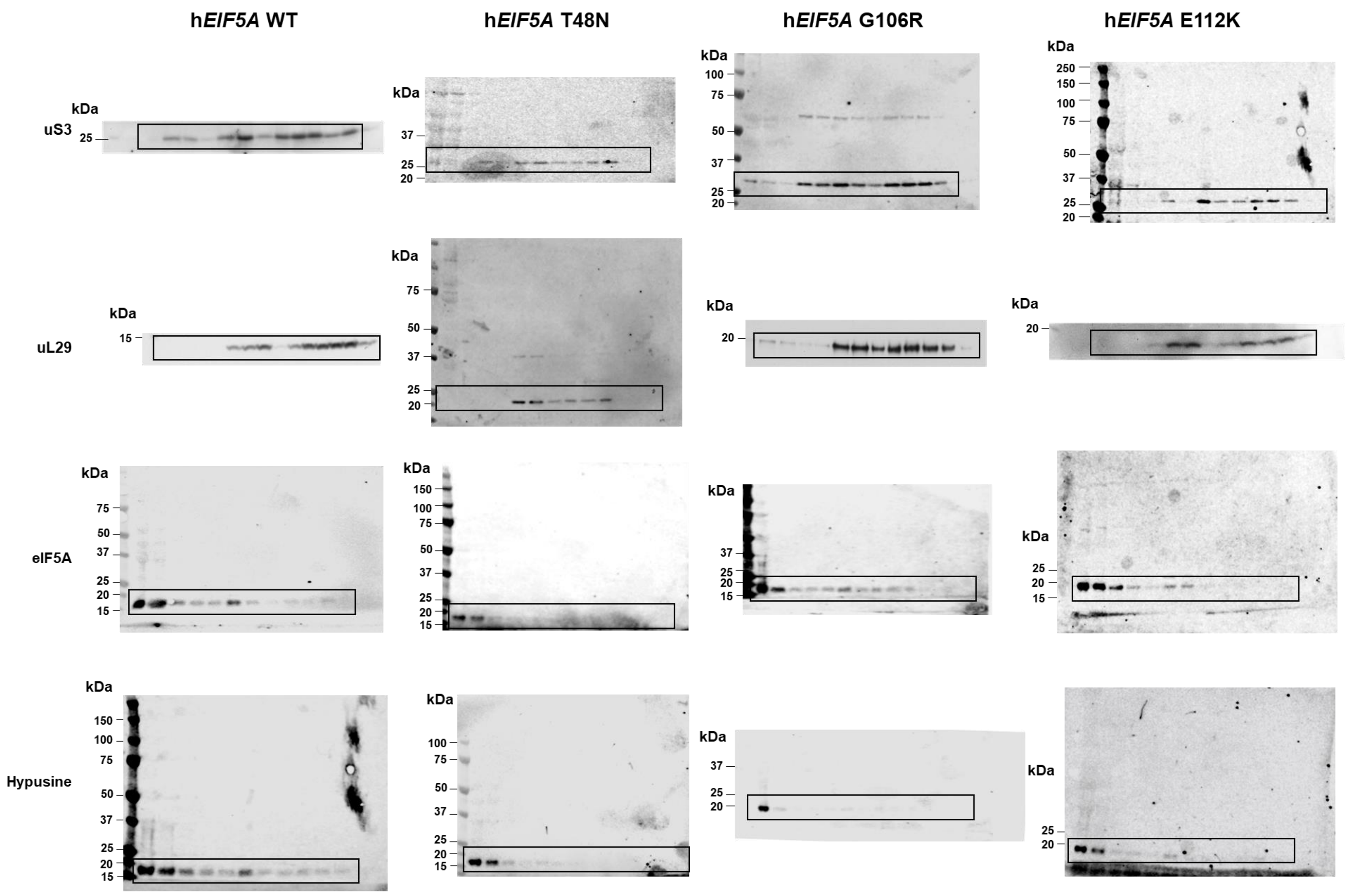

Supplementary Figure 10. Full scans of western blots shown in Fig 2c.

Scans of proteins tested (rows) in polysome profiles according to the strain tested (columns). The black boxes depict the areas shown in Fig. 2c.

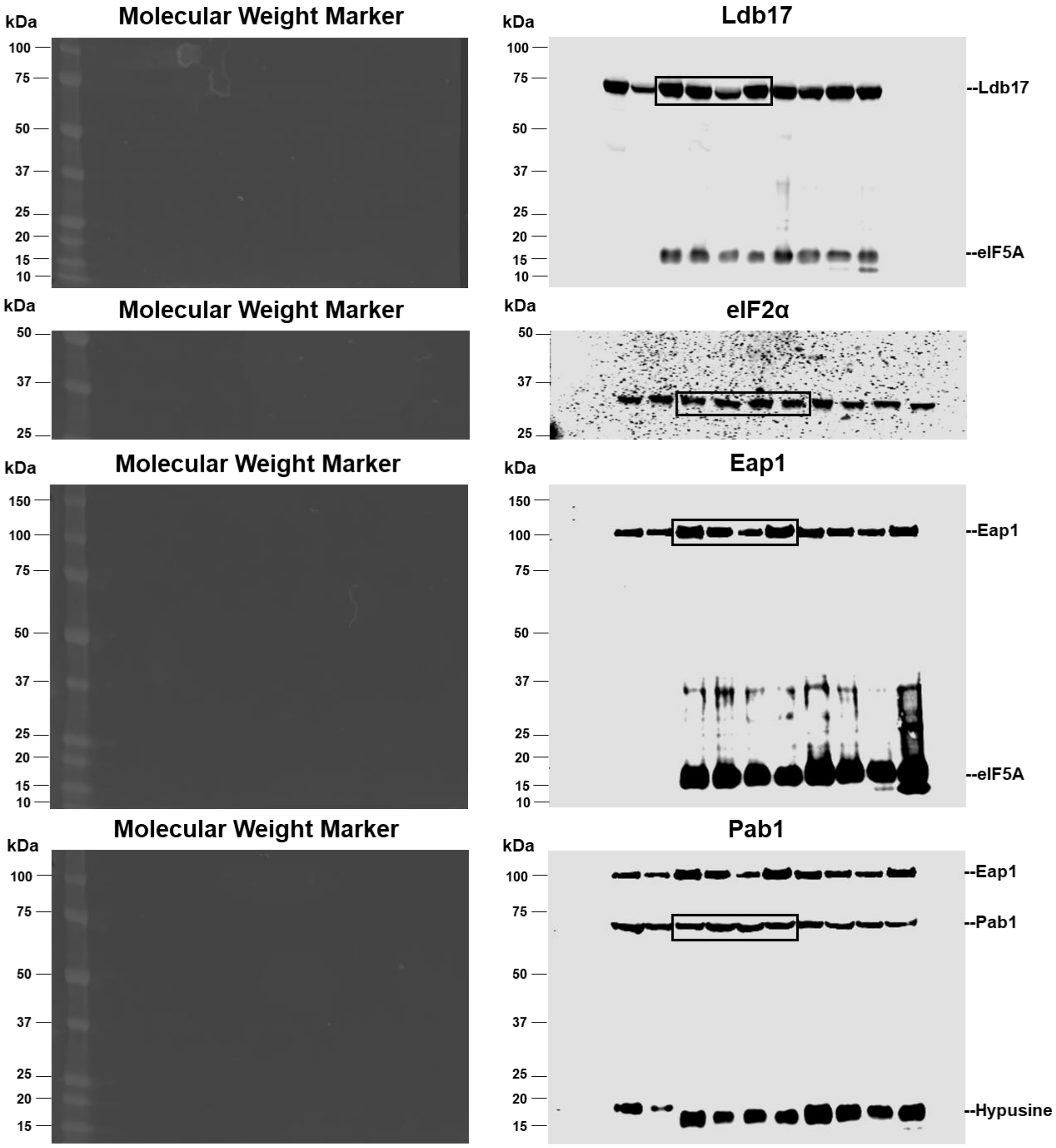

Supplementary Figure 11. Full scans of western blots shown in Fig 2d.

Scans of molecular weight marker, HA-tagged reporters (Ldb17 and Eap1) and loading controls (eIF2α and Pab1). The black boxes depict the areas shown in Fig. 2d.

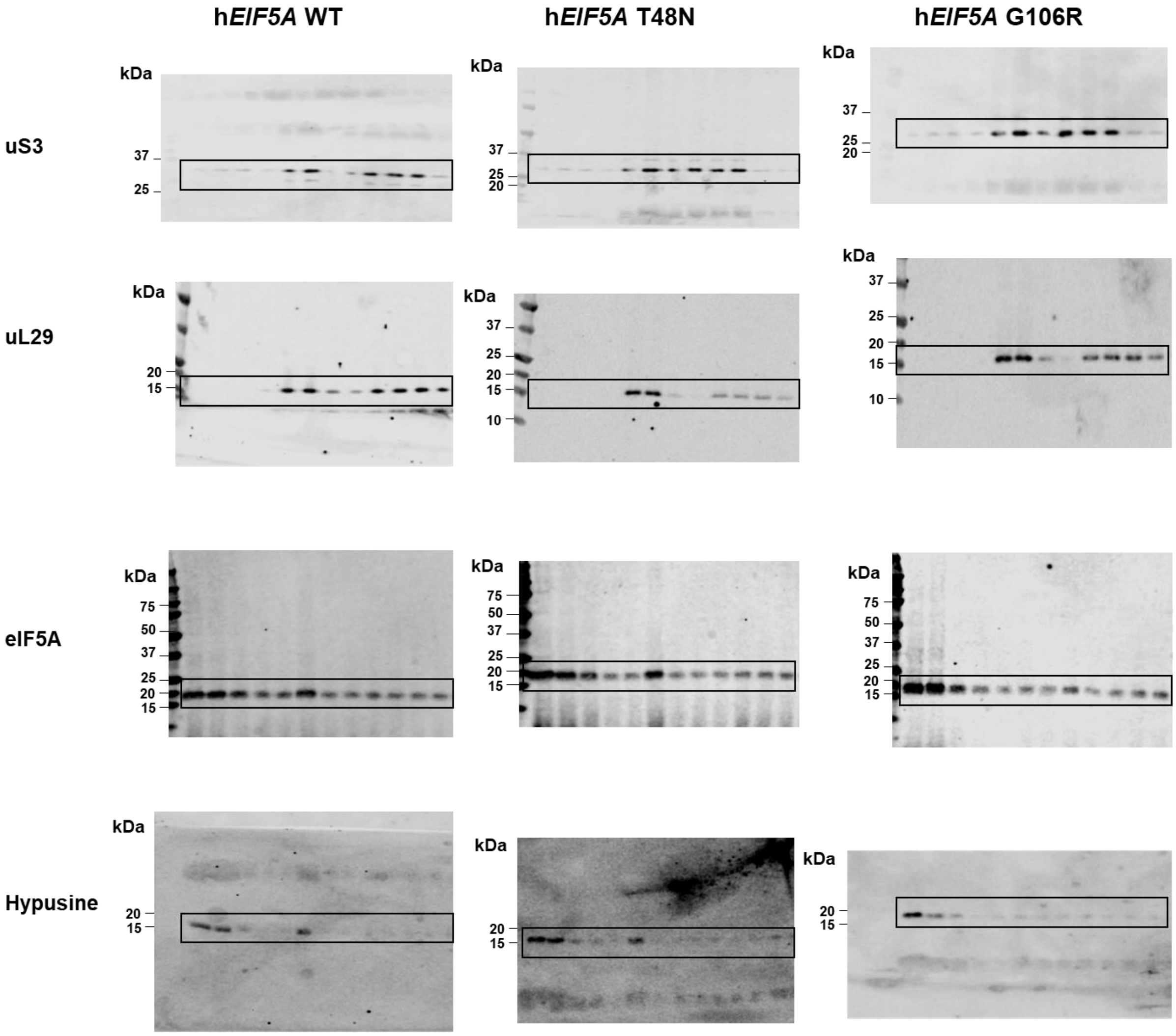

Supplementary Figure 12. Full scans of western blots shown in Fig 3b.

Scans of proteins tested (rows) in polysome profiles under 1mM spermidine treatment according to the strain tested (columns). The black boxes depict the areas shown in Fig. 3b.

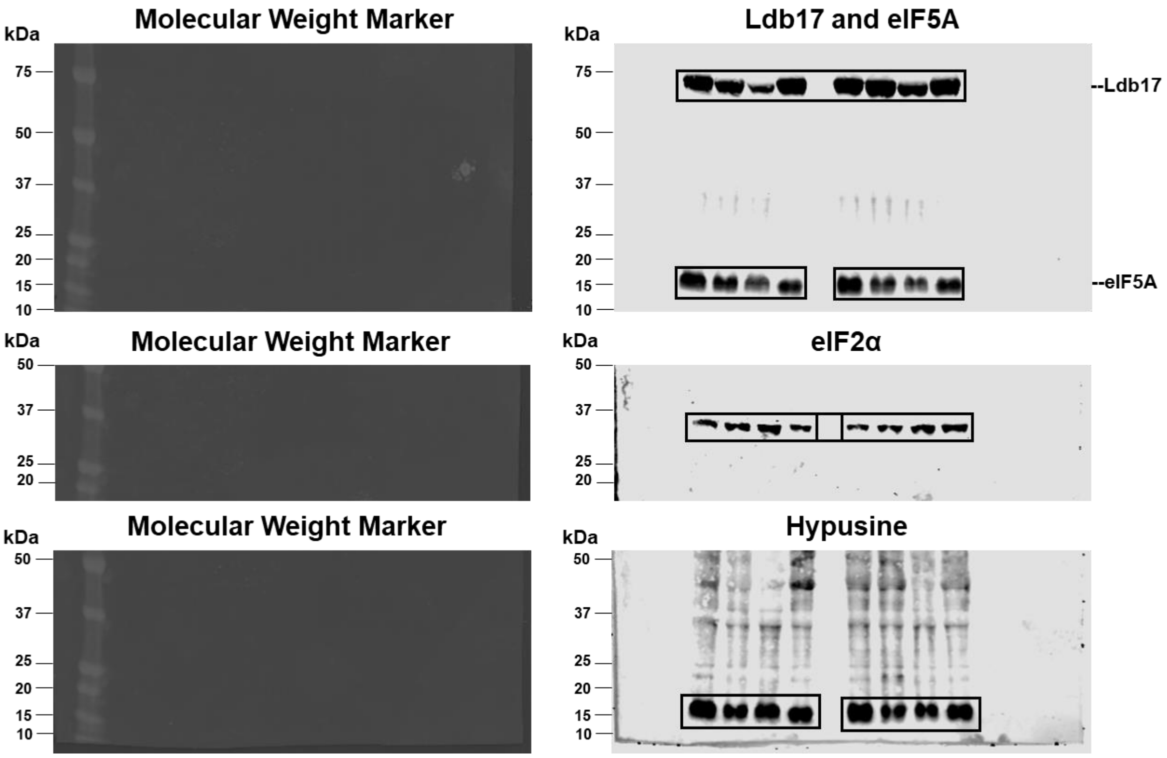

Supplementary Figure 13. Full scans of western blots shown in Fig 3c.

Scans of molecular weight marker, HA-tagged reporter (Ldb17), eIF5A, hypusine and loading control (eIF2α) from strains untreated (first four lanes) and treated with 1mM spermidine (last four lanes). The black boxes depict the areas shown in Fig. 2d

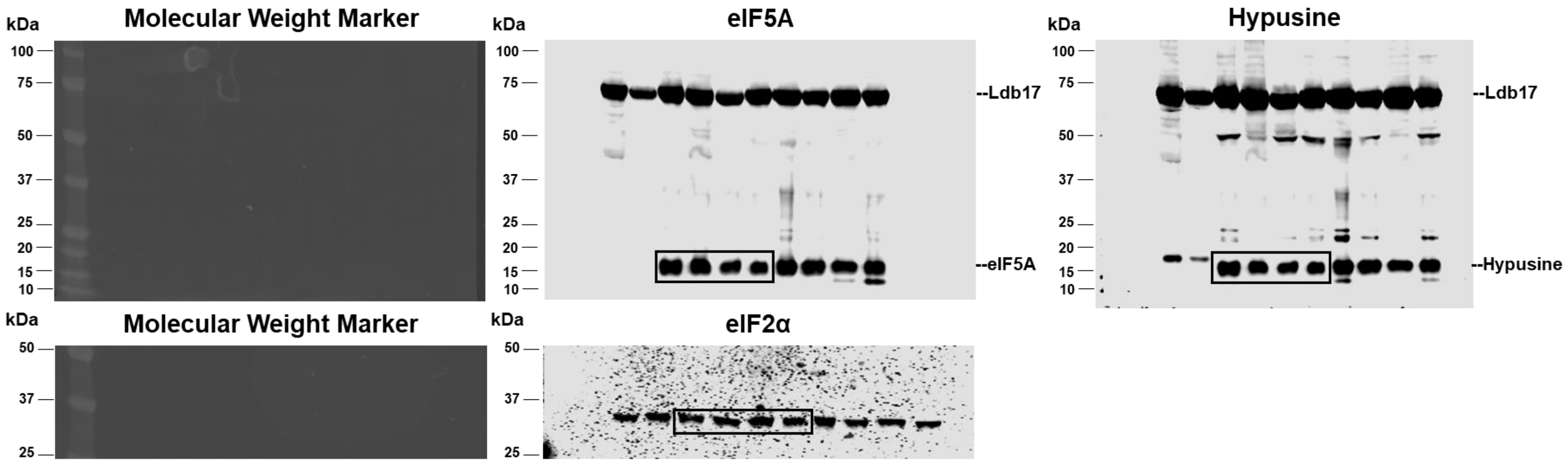

Supplementary Figure 14. Full scans of western blots shown in Supplementary Fig 6.

Scans of molecular weight marker, eIF5A, hypusine and loading control (eIF2α) from strains with HA-tagged Ldb17 reporter. The black boxes depict the areas shown in Supplementary Fig. 6

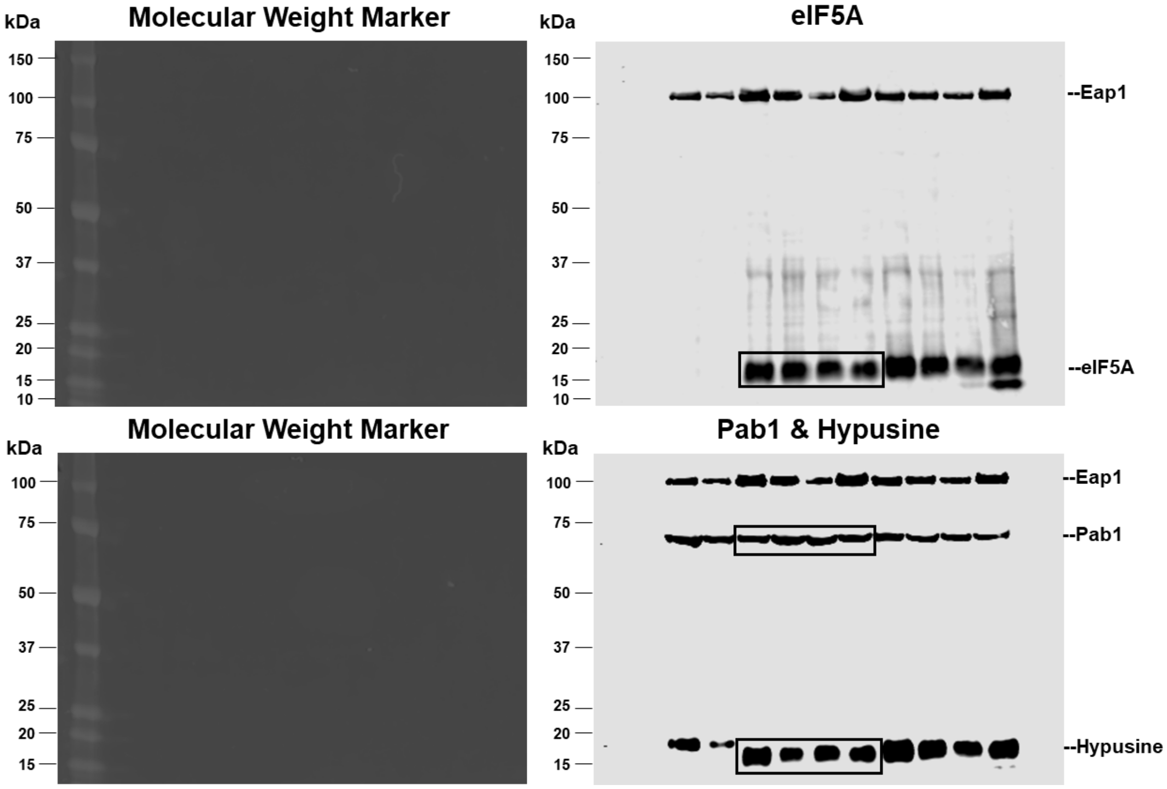

Supplementary Figure 15. Full scans of western blots shown in Supplementary Fig 7.

Scans of molecular weight marker, eIF5A, hypusine and loading control (Pab1) from strains with HA-tagged Eap1 reporter. The black boxes depict the areas shown in Supplementary Fig. 7

Supplementary References

1. Rizzardi, L.F., Dorn, E.S., Strahl, B.D. & Cook, J.G. DNA Replication Origin Function Is Promoted by H3K4 Di-methylation in Saccharomyces cerevisiae. *Genetics* **192**, 371-384 (2012).

2. Saini, P., Eyler, D.E., Green, R. & Dever, T.E. Hypusine-containing protein eIF5A promotes translation elongation. *Nature* **459**, 118-21 (2009).

3. Gutierrez, E. *et al.* eIF5A promotes translation of polyproline motifs. *Mol Cell* **51**, 35-45 (2013).
