# Supplementary material for: Impaired eIF5A function causes a Mendelian disorder that is partially rescued in model systems by spermidine
Source: Nat Commun. 2021 Feb 5;12:833. doi: 10.1038/s41467-021-21053-2 (PMC7864902; doi:10.1038/s41467-021-21053-2)
Supplement: Supplementary file 3 — Description of Additional Supplementary Files [file 41467_2021_21053_MOESM3_ESM.pdf]

Description of Additional Supplementary Files

File Name: Supplementary Data 1.

Description: Catalogue of known human microcephaly-associated genes and their polyproline tract content.
