# Supplementary material for: Impaired eIF5A function causes a Mendelian disorder that is partially rescued in model systems by spermidine
Source: Nat Commun. 2021 Feb 5;12:833. doi: 10.1038/s41467-021-21053-2 (PMC7864902; doi:10.1038/s41467-021-21053-2)
Supplement: Supplementary file 5 — Reporting Summary [file 41467_2021_21053_MOESM5_ESM.pdf]

nature research

Corresponding author(s): Siddharth Banka, Graham Pavitt, Paul Kasher

Last updated by author(s): Aug 31, 2020

Reporting Summary

Nature Research wishes to improve the reproducibility of the work that we publish. This form provides structure for consistency and transparency in reporting. For further information on Nature Research policies, see Authors & Referees and the Editorial Policy Checklist .

Statistics

For all statistical analyses, confirm that the following items are present in the figure legend, table legend, main text, or Methods section.

n/a	Confirmed	
☐	☒	The exact sample size (n) for each experimental group/condition, given as a discrete number and unit of measurement
☐	☒	A statement on whether measurements were taken from distinct samples or whether the same sample was measured repeatedly
☐	☒	The statistical test(s) used AND whether they are one- or two-sided<br>*Only common tests should be described solely by name; describe more complex techniques in the Methods section.*
☐	☒	A description of all covariates tested
☐	☒	A description of any assumptions or corrections, such as tests of normality and adjustment for multiple comparisons
☐	☒	A full description of the statistical parameters including central tendency (e.g. means) or other basic estimates (e.g. regression coefficient) AND variation (e.g. standard deviation) or associated estimates of uncertainty (e.g. confidence intervals)
☐	☒	For null hypothesis testing, the test statistic (e.g. F, t, r) with confidence intervals, effect sizes, degrees of freedom and P value noted<br>*Give P values as exact values whenever suitable.*
☒	☐	For Bayesian analysis, information on the choice of priors and Markov chain Monte Carlo settings
☒	☐	For hierarchical and complex designs, identification of the appropriate level for tests and full reporting of outcomes
☒	☐	Estimates of effect sizes (e.g. Cohen's d, Pearson's r), indicating how they were calculated

Our web collection on statistics for biologists contains articles on many of the points above.

Software and code

Policy information about availability of computer code

Data collection	GraphPad Prism v8.3.0, LASX image capture v3.4.2, ImageJ v1.52, LI-COR Odyssey® CLx Imaging System, Excel Professional Plus 2016
Data analysis	GraphPad Prism 8.3.0, SPSS v25

For manuscripts utilizing custom algorithms or software that are central to the research but not yet described in published literature, software must be made available to editors/reviewers. We strongly encourage code deposition in a community repository (e.g. GitHub). See the Nature Research guidelines for submitting code & software for further information.

Data

Policy information about availability of data

All manuscripts must include a data availability statement. This statement should provide the following information, where applicable:

- Accession codes, unique identifiers, or web links for publicly available datasets
- A list of figures that have associated raw data
- A description of any restrictions on data availability

The datasets generated during and/or analysed during the current study are available in the "Data Availability". Public databases used for the study Constrained Coding Regions, https://s3.us-east-2.amazonaws.com/ccrs/ccr.html; DECIPHER, https://decipher.sanger.ac.uk; DOMINO, https://wwwfbm.unil.ch/domino/search_results.php; Ensembl GRCh37, http://grch37.ensembl.org; Exome Variant Server, http://evs.gs.washington.edu/EVS/; GeVIR, http://www.gevirank.org/; gnomAD, http://gnomad.broadinstitute.org/; GTEx Portal, https://www.gtexportal.org/home; InterPro, https://www.ebi.ac.uk/interpro/; MutationMapper, http://www.cbioportal.org/mutation_mapper.jsp; OMIM, https://www.omim.org/; Protein Data Bank in Europe, http://www.ebi.ac.uk/pdbe/; The 1000 Genomes Project, http://phase3browser.1000genomes.org/index.html; UK10K Project, https://www.uk10k.org/; UniProtKB, https://www.uniprot.org/

Field-specific reporting

Please select the one below that is the best fit for your research. If you are not sure, read the appropriate sections before making your selection.

☒ Life sciences ☐ Behavioural & social sciences ☐ Ecological, evolutionary & environmental sciences

For a reference copy of the document with all sections, see nature.com/documents/nr-reporting-summary-flat.pdf

Life sciences study design

All studies must disclose on these points even when the disclosure is negative.

Sample size	No sample size calculations were required as use of three technical or biological replicates has been historically accepted as valid. The number of zebrafish eggs injected was the highest number that each fertilization process allowed, and each process was considered as one replicate. For yeast analyses, one liquid culture of one strain was considered as one replicate.
Data exclusions	No exclusion criteria was used, and therefore, no data were excluded.
Replication	Yeast and zebrafish experiments were performed using three biological replicates, except for RT-PCR of EIF5A transcript from Individual 3, which was performed using three technical replicates. Each replication was successful at the first attemp.
Randomization	No exclusion criteria was used as it was not relevant to our study.
Blinding	Investigators who performed egg injections were blinded to measurements of zebrafish parameters. For remaining experiments, blinding was not relevant.

Reporting for specific materials, systems and methods

We require information from authors about some types of materials, experimental systems and methods used in many studies. Here, indicate whether each material, system or method listed is relevant to your study. If you are not sure if a list item applies to your research, read the appropriate section before selecting a response.

Materials & experimental systems

n/a	Involved in the study	
☐	☒	Antibodies
☒	☐	Eukaryotic cell lines
☒	☐	Palaeontology
☐	☒	Animals and other organisms
☐	☒	Human research participants
☒	☐	Clinical data

Methods

n/a	Involved in the study	
☒	☐	ChIP-seq
☒	☐	Flow cytometry
☒	☐	MRI-based neuroimaging

Antibodies

Antibodies used	anti-eIF5A (611977, BD Biosciences), rabbit anti-hypusine (ABS1064, EMD Millipore), mouse anti-Pab1 (MCA-1G1, EnCor Biotechnology), mouse anti-HA.11 (901513, BioLegend), chicken anti-eIF2α (custom designed, Cambridge Research Biochemicals), secondary fluorescent labelled donkey anti-chicken, goat anti-rabbit, anti-mouse antibodies (IRDye® 800CW, LI-COR Biosciences), Rps3(uS3)/ Rpl35(uL29) rabbit polyclonal antibodies (a kind gift from Dr Martin Pool, University of Manchester). Dilutions described in the Methods section of the manuscript.
Validation	DOIs numbers: 10.1186/2193-1801-2-421; 10.7554/eLife.24542; 10.1186/s13059-017-1338-4; 10.1016/j.molcel.2013.04.021

Animals and other organisms

Policy information about studies involving animals; ARRIVE guidelines recommended for reporting animal research

Laboratory animals	Nacre zebrafish adults (6 months of age) were used in a ratio of 2F:1M for breeding purposes
Wild animals	This study did not involve wild animals
Field-collected samples	This study did not involve field-collected samples
Ethics oversight	Zebrafish husbandry was approved by The University of Manchester Ethical Review Board and all experiments were performed in accordance with UK Home Office regulations (PPL P132EB6D7).

Note that full information on the approval of the study protocol must also be provided in the manuscript.

Human research participants

Policy information about studies involving human research participants

Population characteristics	Patients with heterozygous, de novo EIF5A variants, regardless of their phenotype. No other characteristic was a prerequisite for the study.
Recruitment	The clinical observations were gathered through the Matchmaker Exchange and GeneMatcher initiatives and shared by the clinicans who are controlling patients with EIF5A variants. No patient with EIF5A variant was excluded so there was no selection bias

Ethics oversight	The Central Manchester, Cambridge South, and the Republic of Ireland RECs approved this study (02/CM/238, 10/H0305/83 and GEN/284/12, respectively).

Note that full information on the approval of the study protocol must also be provided in the manuscript.

Clinical data

Policy information about clinical studies

All manuscripts should comply with the ICMJEguidelines for publication of clinical research and a completedCONSORT checklist must be included with all submissions.

Clinical trial registration	*Provide the trial registration number from ClinicalTrials.gov or an equivalent agency.*
Study protocol	*Note where the full trial protocol can be accessed OR if not available, explain why.*
Data collection	*Describe the settings and locales of data collection, noting the time periods of recruitment and data collection.*
Outcomes	*Describe how you pre-defined primary and secondary outcome measures and how you assessed these measures.*
