# Supplementary material for: Impaired eIF5A function causes a Mendelian disorder that is partially rescued in model systems by spermidine
Source: Nat Commun. 2021 Feb 5;12:833. doi: 10.1038/s41467-021-21053-2 (PMC7864902; doi:10.1038/s41467-021-21053-2)
Supplement: Supplementary file 6 — Source Data [file 41467_2021_21053_MOESM6_ESM.zip › Source Data/Public databases and programmes used for the study.docx]

**Public databases and programmes used for the study**

Constrained Coding Regions, <https://s3.us-east-2.amazonaws.com/ccrs/ccr.html>

DECIPHER, <https://decipher.sanger.ac.uk>

DOMINO, <https://wwwfbm.unil.ch/domino/search_results.php>

Ensembl GRCh37, <http://grch37.ensembl.org>

Exome Variant Server, <http://evs.gs.washington.edu/EVS/>

GeVIR, <http://www.gevirank.org/>

gnomAD, <http://gnomad.broadinstitute.org/>

GTEx Portal, <https://www.gtexportal.org/home>

InterPro, <https://www.ebi.ac.uk/interpro/>

MutationMapper, <http://www.cbioportal.org/mutation_mapper.jsp>

OMIM, <https://www.omim.org/>

Protein Data Bank in Europe, <http://www.ebi.ac.uk/pdbe/>

The 1000 Genomes Project, <http://phase3browser.1000genomes.org/index.html>

UK10K Project, <https://www.uk10k.org/>;

UniProtKB, <https://www.uniprot.org/>
