# Supplementary material for: Impaired eIF5A function causes a Mendelian disorder that is partially rescued in model systems by spermidine
Source: Nat Commun. 2021 Feb 5;12:833. doi: 10.1038/s41467-021-21053-2 (PMC7864902; doi:10.1038/s41467-021-21053-2)

SD+ W+URA+5FOA, Day 3, 25 degrees, Plate 1


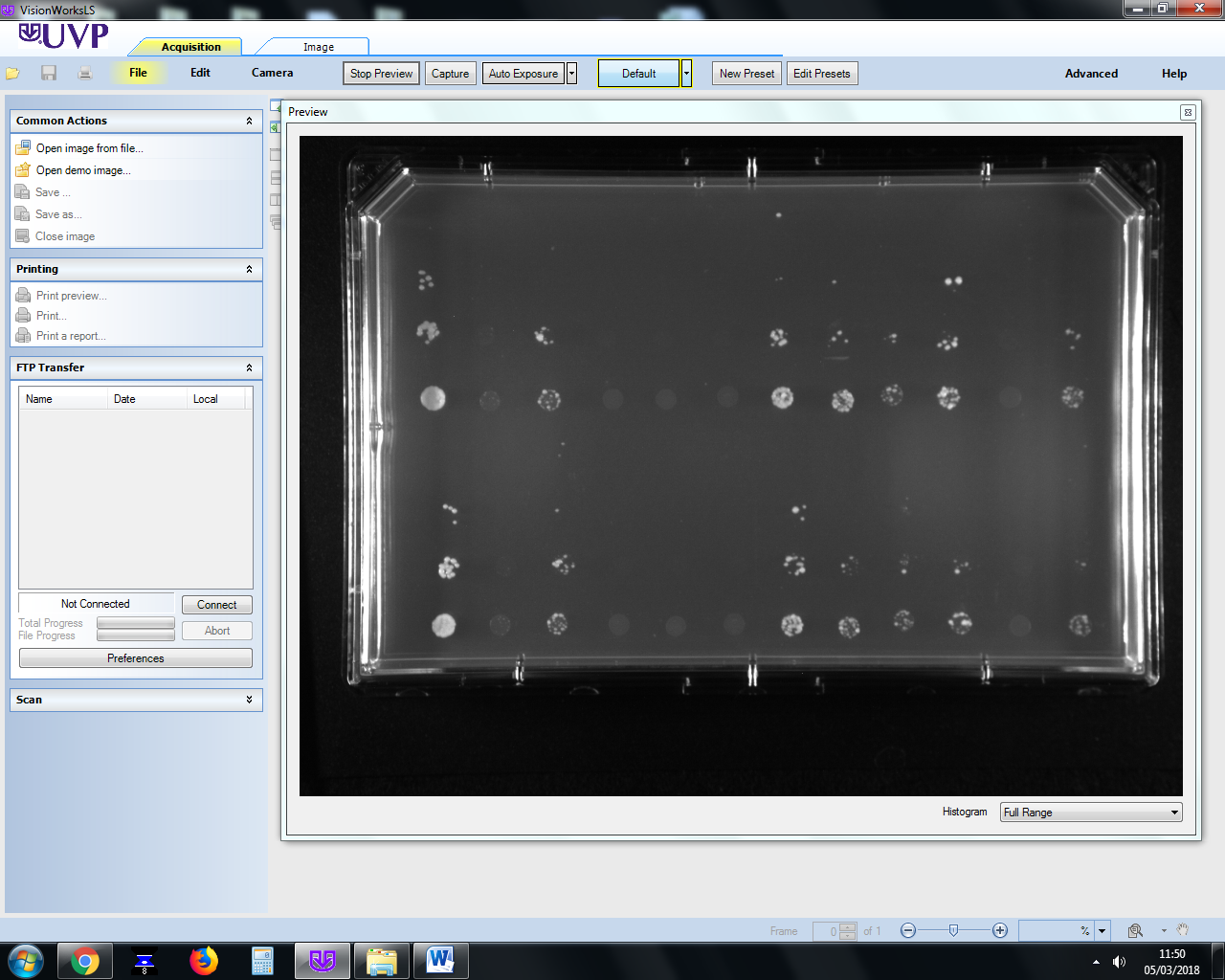


SD+ W+URA+5FOA, Day 3, 25 degrees, Plate 2


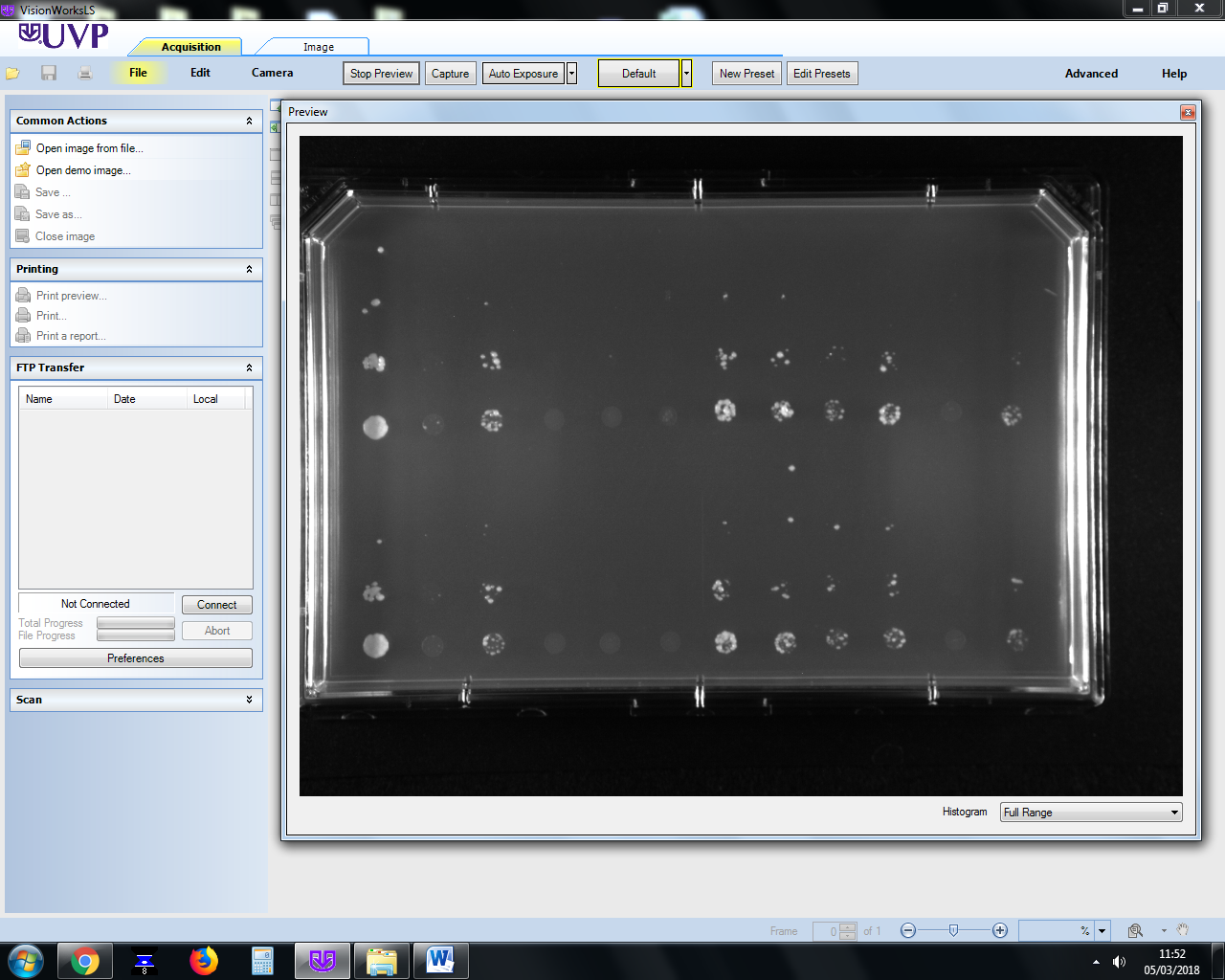


SD+ W+URA+5FOA, Day 3, 25 degrees, Plate 3


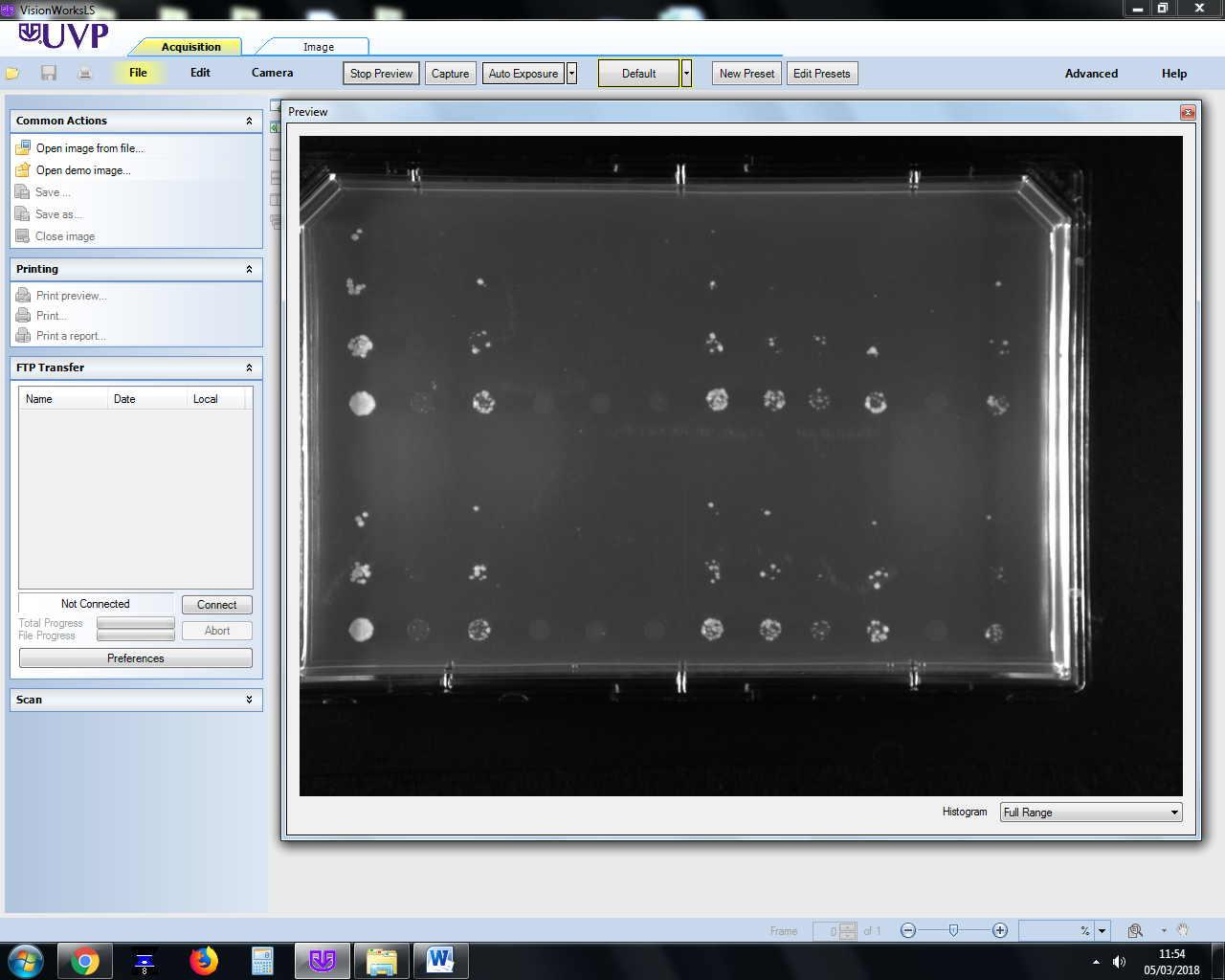


SD+ W+URA+5FOA, Day 3, 30 degrees, Plate 1


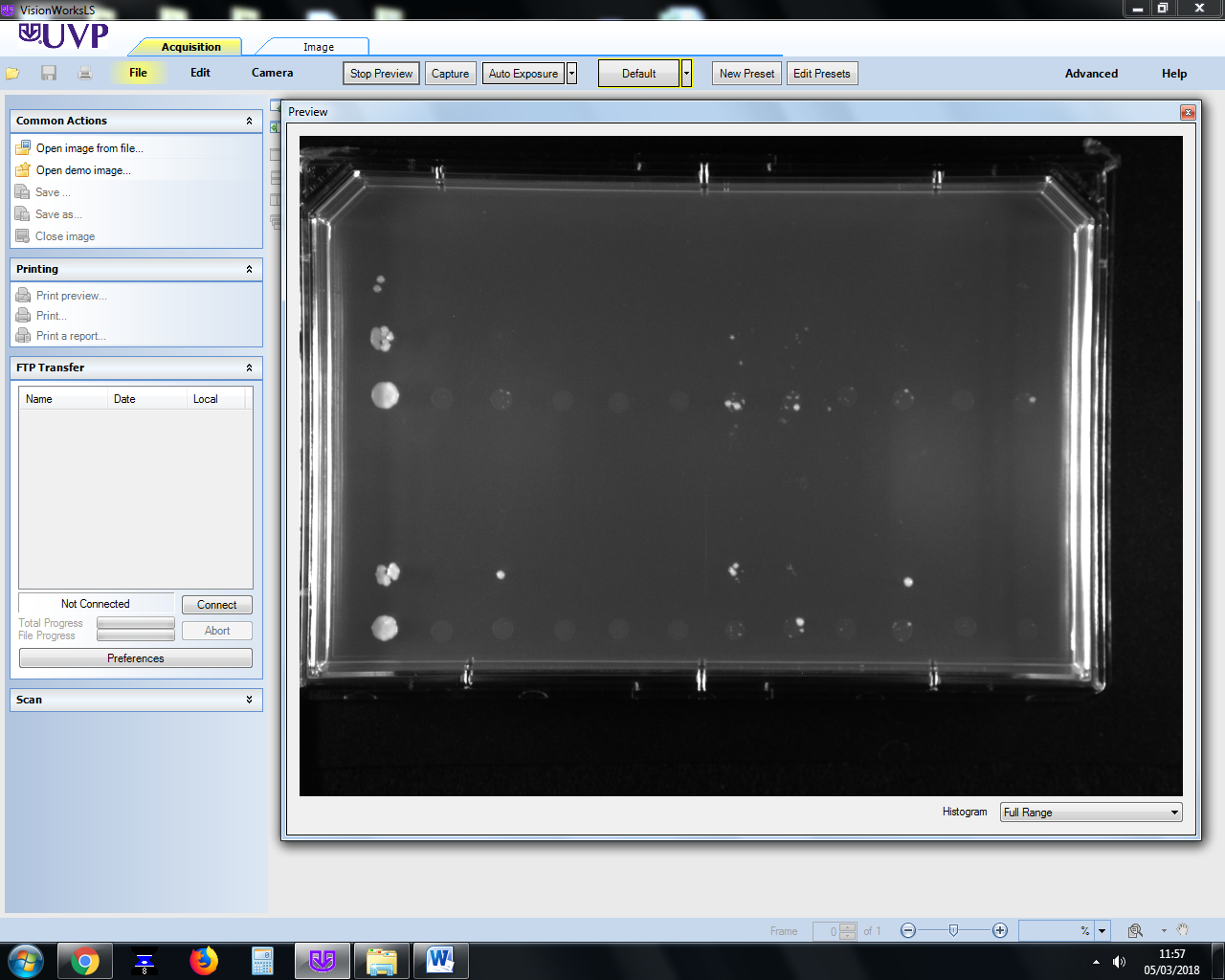


SD+ W+URA+5FOA, Day 3, 30 degrees, Plate 2


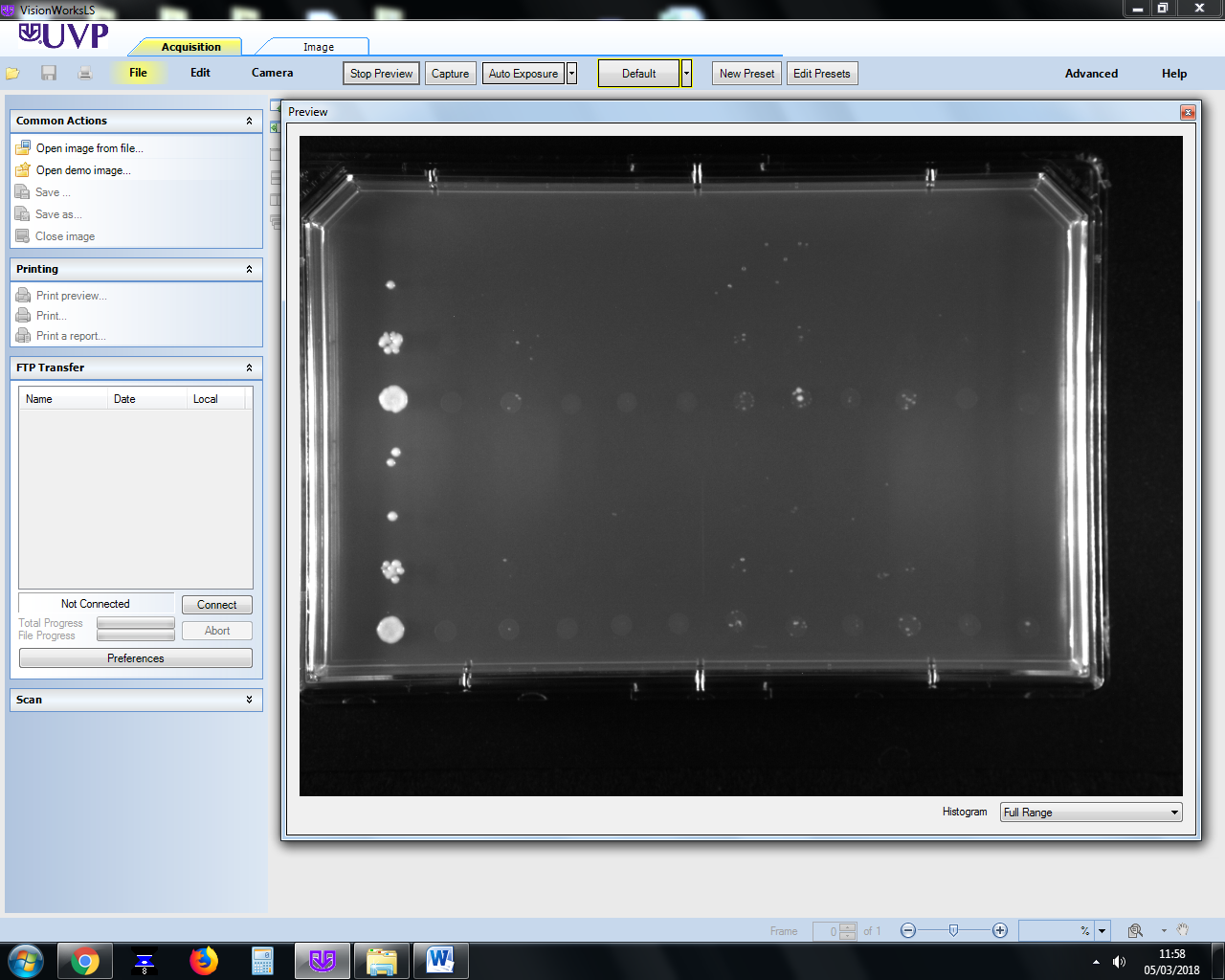


SD+ W+URA+5FOA, Day 3, 30 degrees, Plate 3


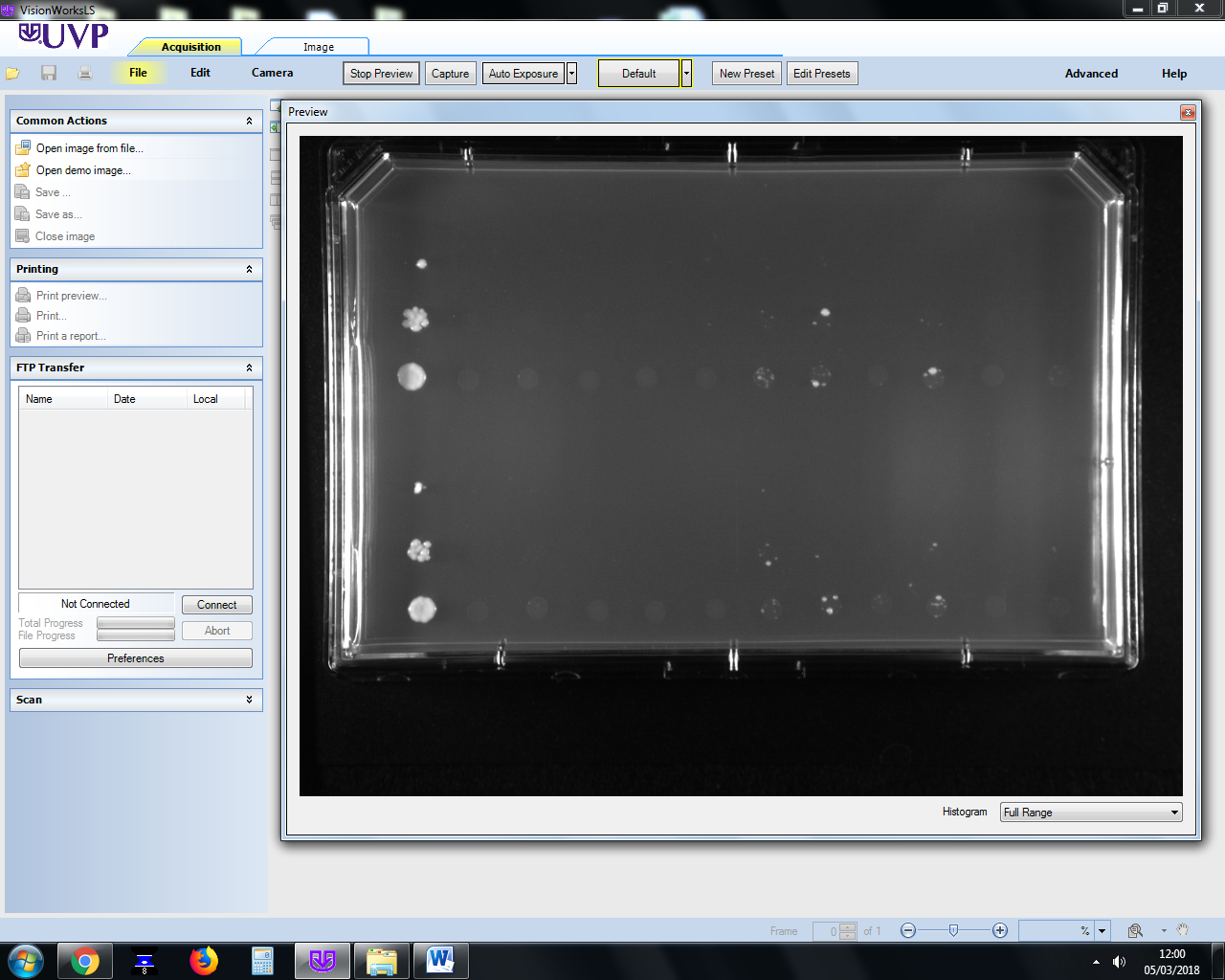


SD+ W+URA+5FOA, Day 3, 37 degrees, Plate 1


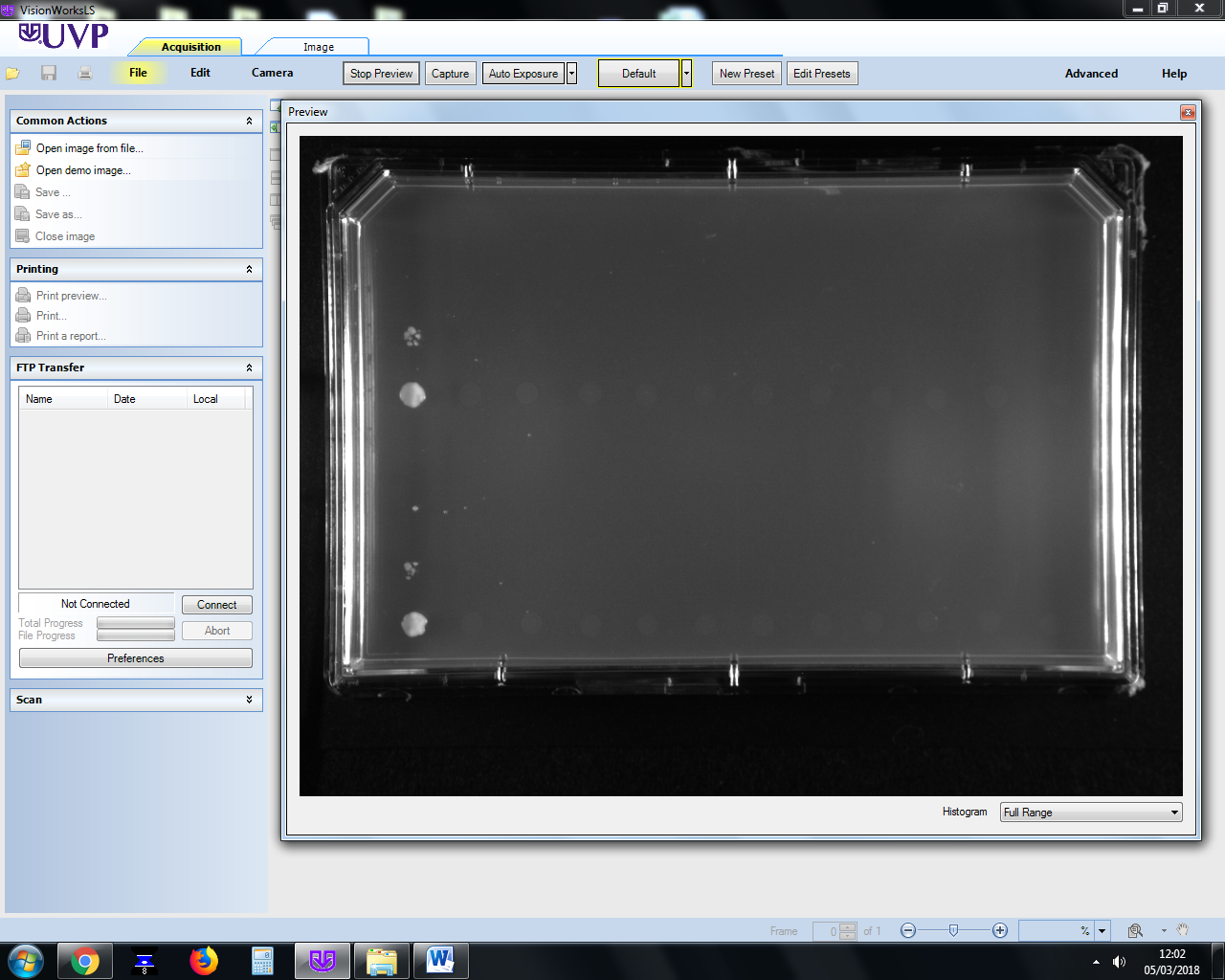


SD+ W+URA+5FOA, Day 3, 37 degrees, Plate 2


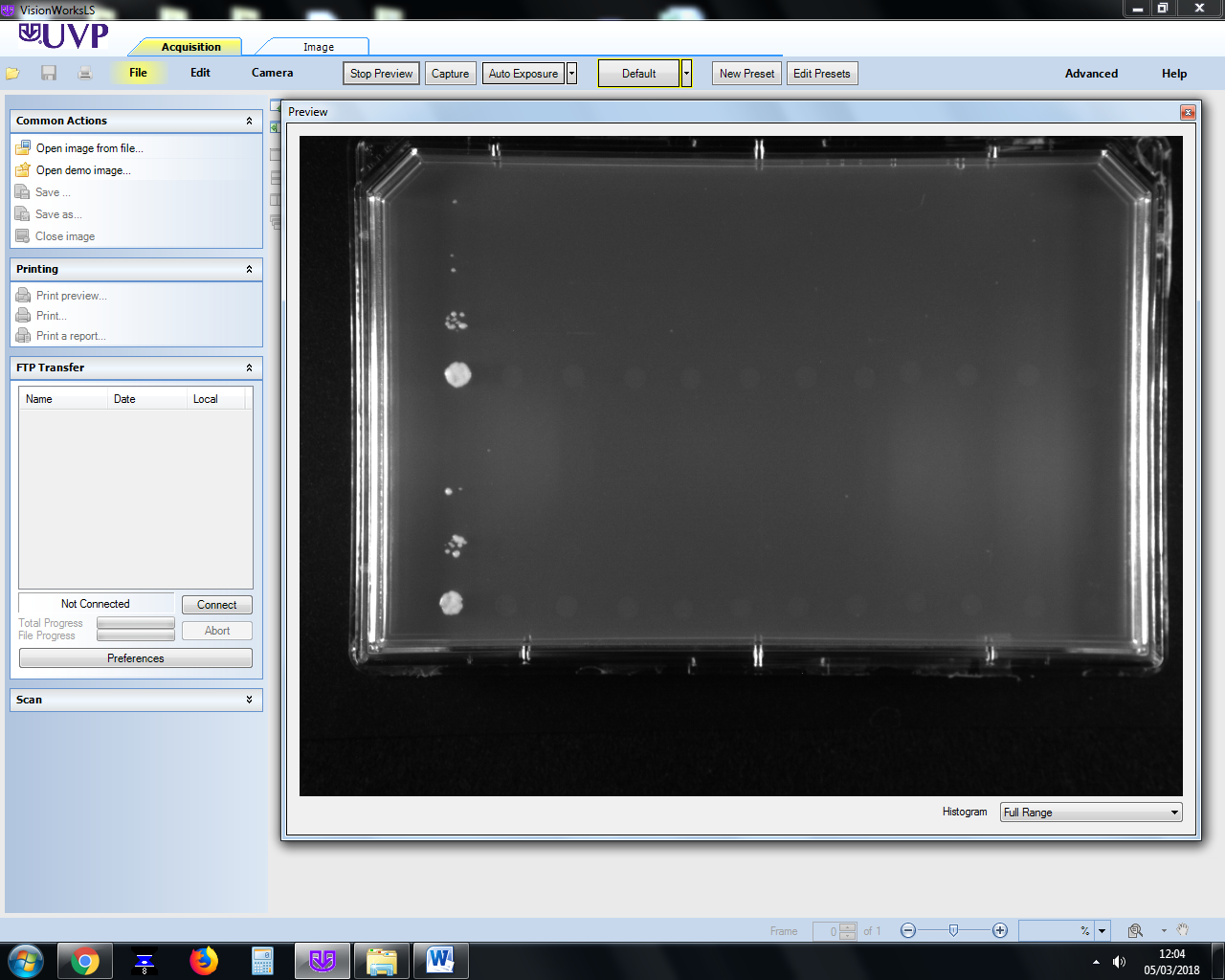


SD+ W+URA+5FOA, Day 3, 37 degrees, Plate 3


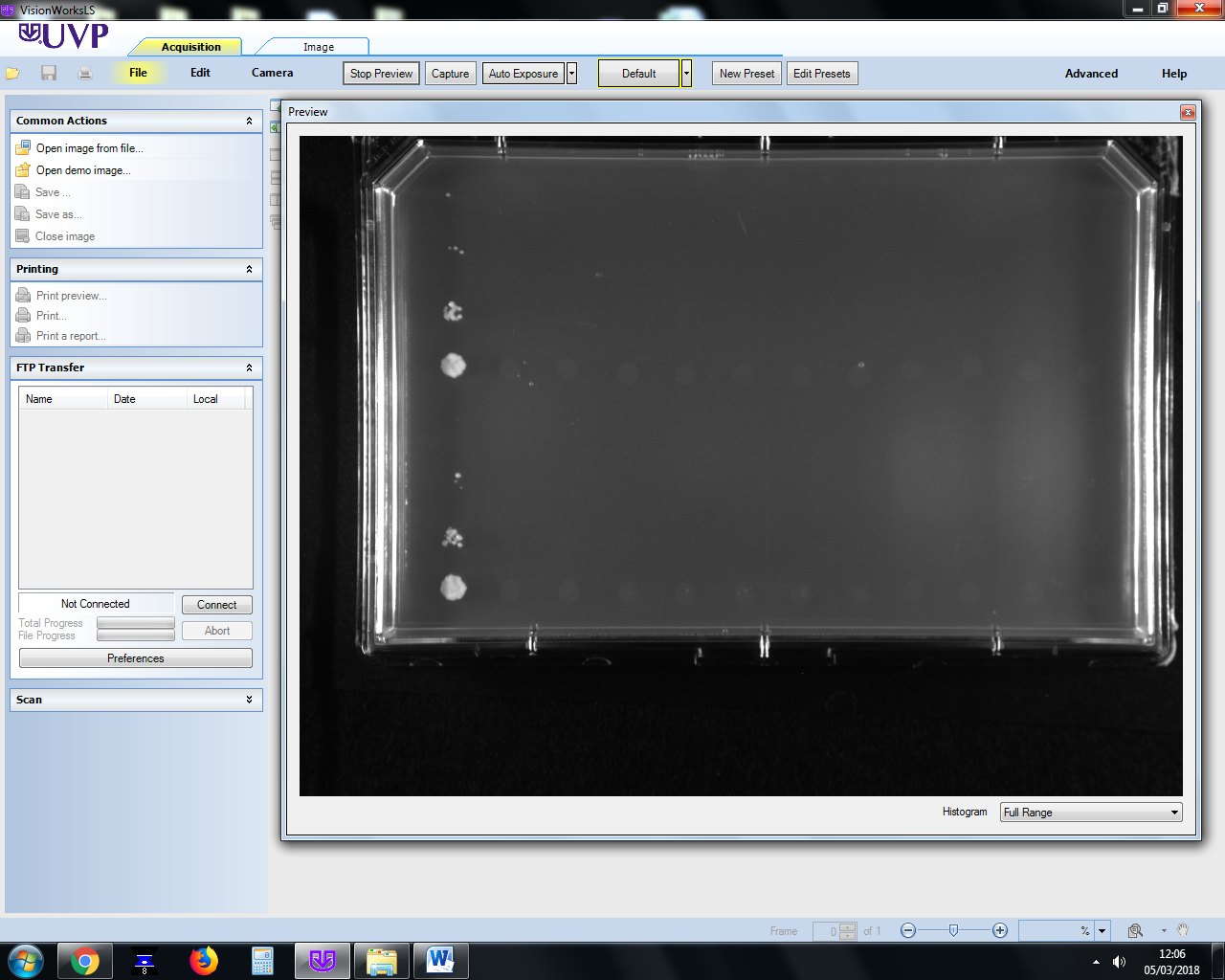


SD+ W+URA+5FOA, Day 3, 37 degrees, Plate 4


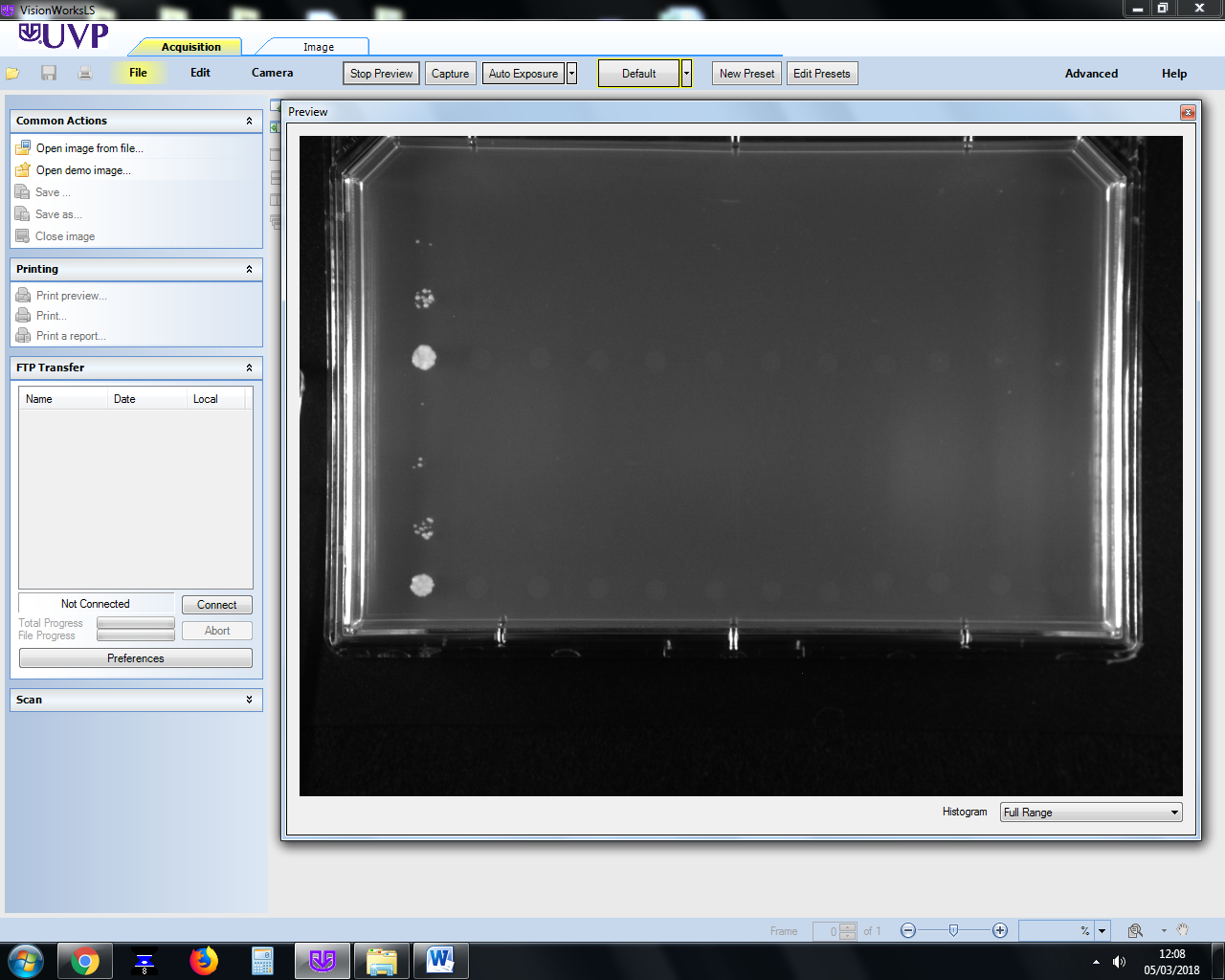

Supplement: Supplementary file 6 — Source Data [file 41467_2021_21053_MOESM6_ESM.zip › Source Data/Yeast Work/Dilutions Sup. Fig. 3/SD+W+Ura+5FOA/SD+W+URA+5FOA, Day 3.docx]
