# Supplementary figures and images for: Impaired eIF5A function causes a Mendelian disorder that is partially rescued in model systems by spermidine (part 2 of 2)
Source: Nat Commun. 2021 Feb 5;12:833. doi: 10.1038/s41467-021-21053-2 (PMC7864902; doi:10.1038/s41467-021-21053-2)

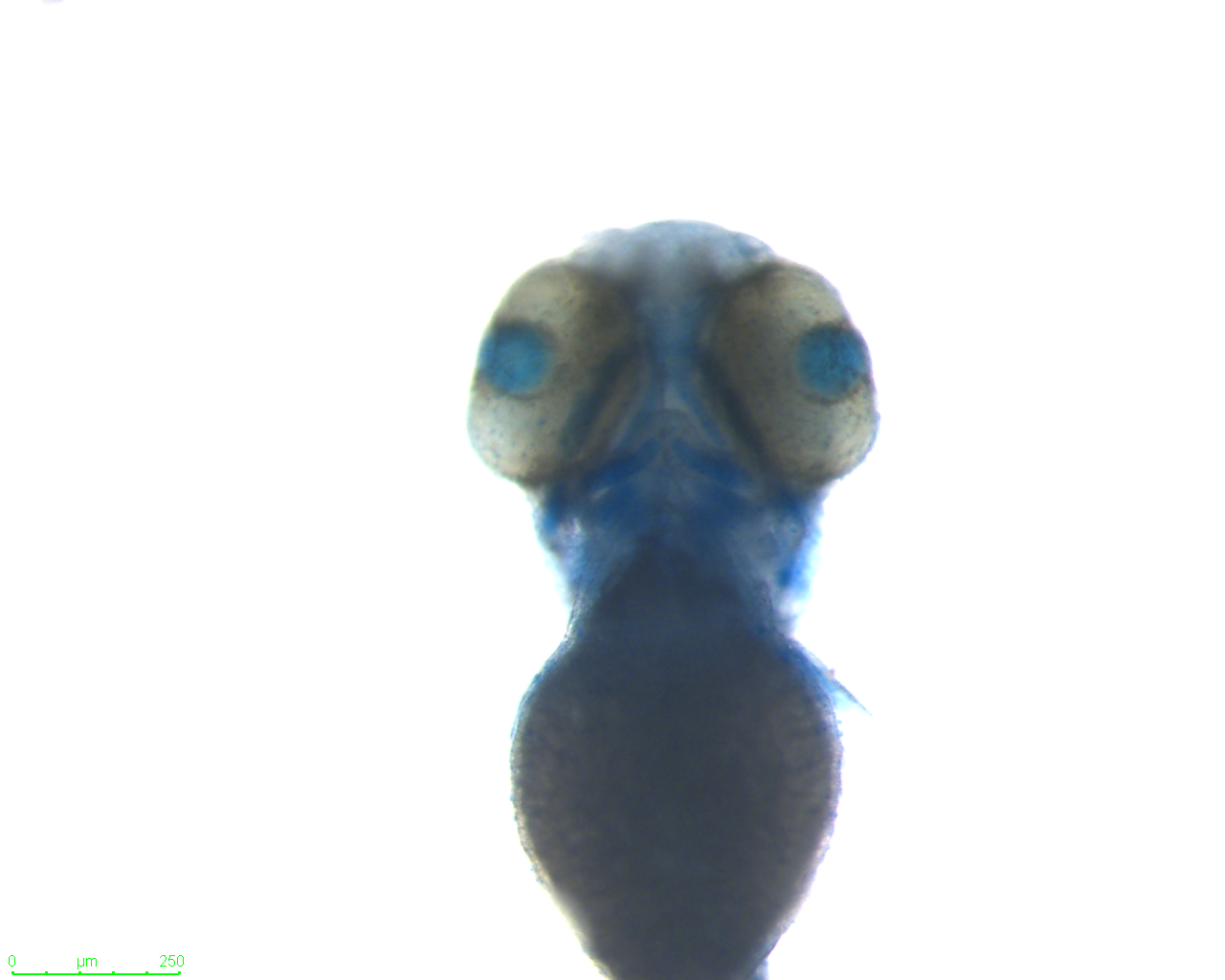

Supplement: Supplementary file 6 — Source Data [file 41467_2021_21053_MOESM6_ESM.zip › Source Data/Zebrafish Morpholino work/Third replicate/EIF5A images 090219_Control_UNT_02.tif]

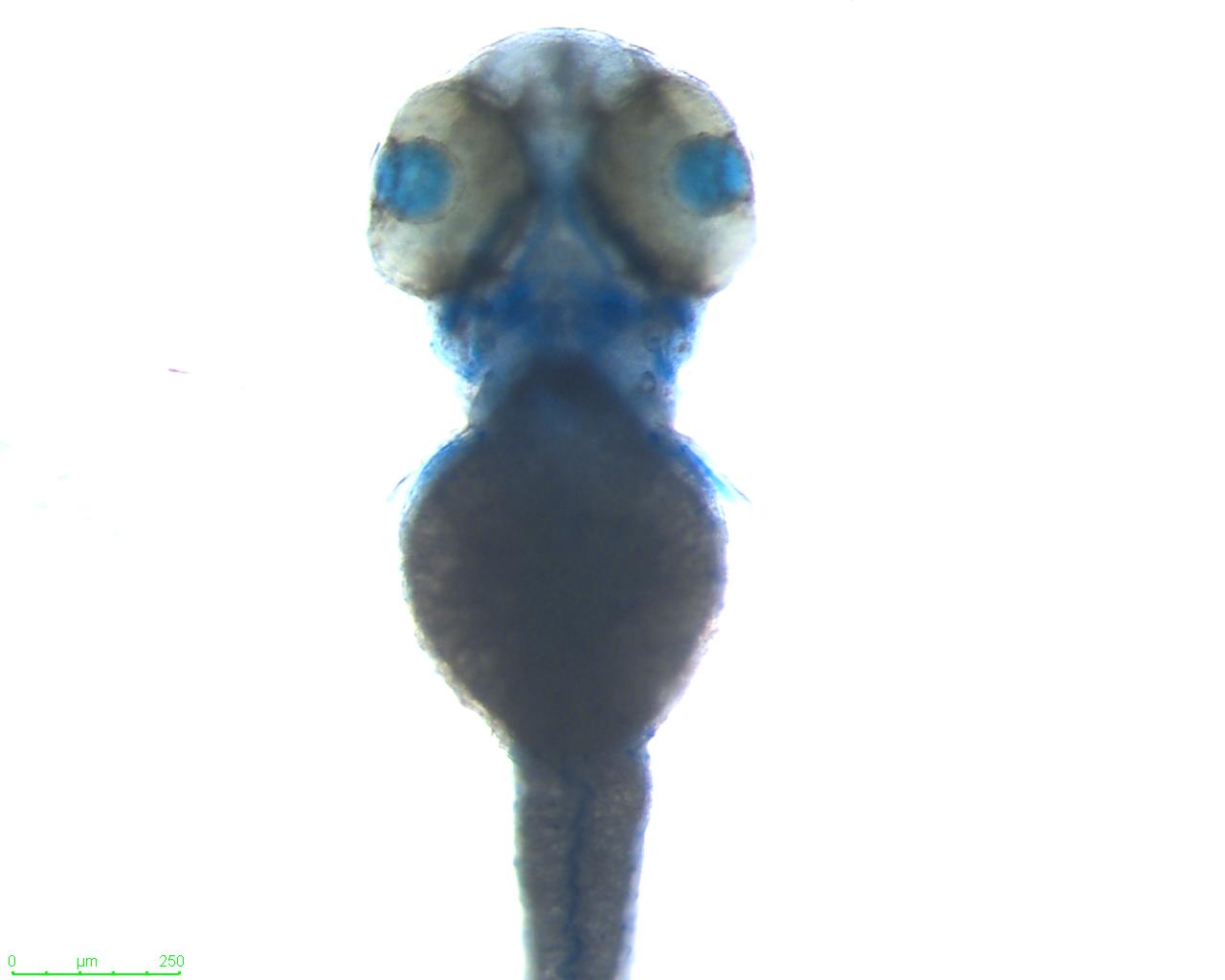

Supplement: Supplementary file 6 — Source Data [file 41467_2021_21053_MOESM6_ESM.zip › Source Data/Zebrafish Morpholino work/Third replicate/EIF5A images 090219_Control_UNT_03.tif]

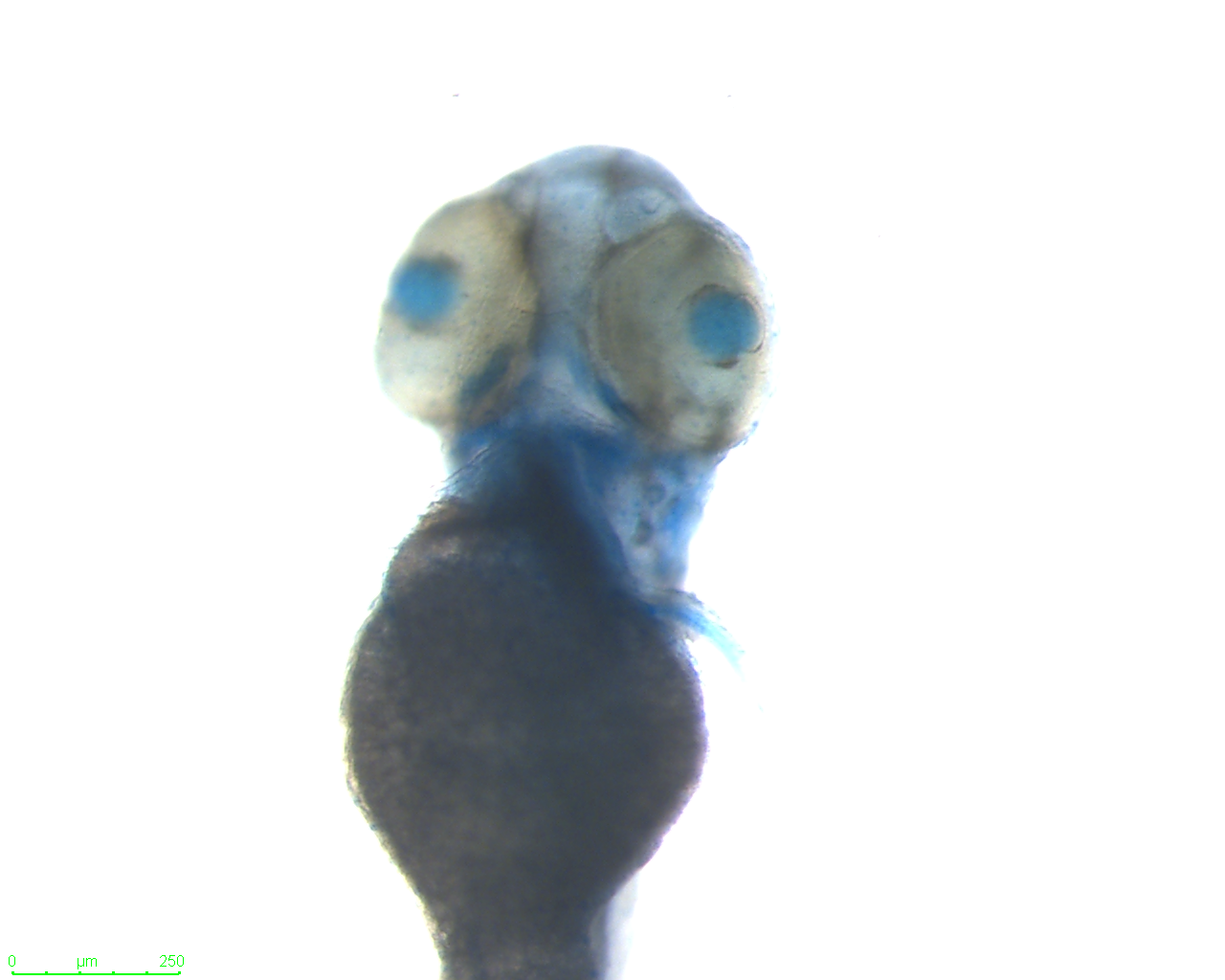

Supplement: Supplementary file 6 — Source Data [file 41467_2021_21053_MOESM6_ESM.zip › Source Data/Zebrafish Morpholino work/Third replicate/EIF5A images 090219_Control_UNT_04.tif]

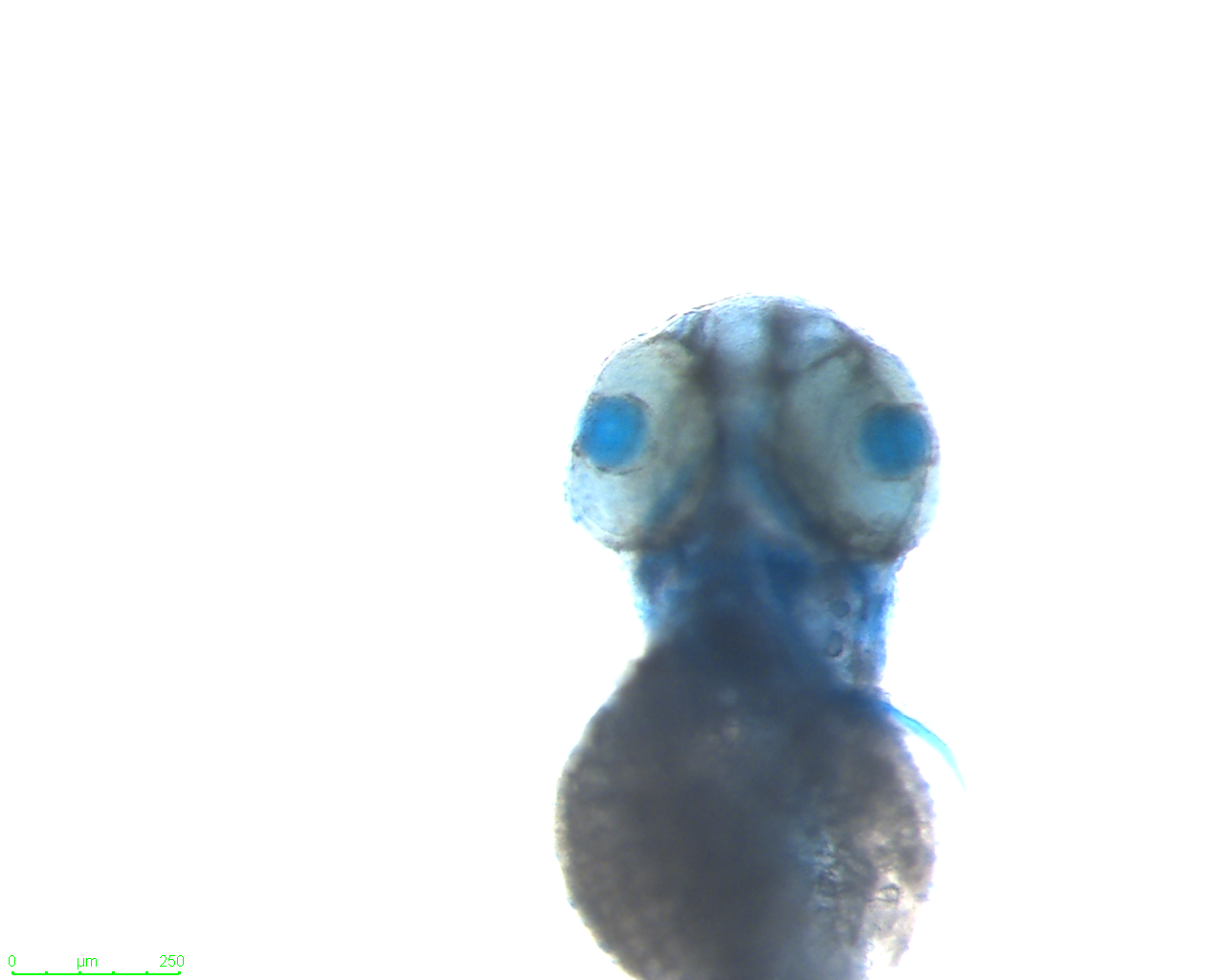

Supplement: Supplementary file 6 — Source Data [file 41467_2021_21053_MOESM6_ESM.zip › Source Data/Zebrafish Morpholino work/Third replicate/EIF5A images 090219_Control_UNT_05.tif]

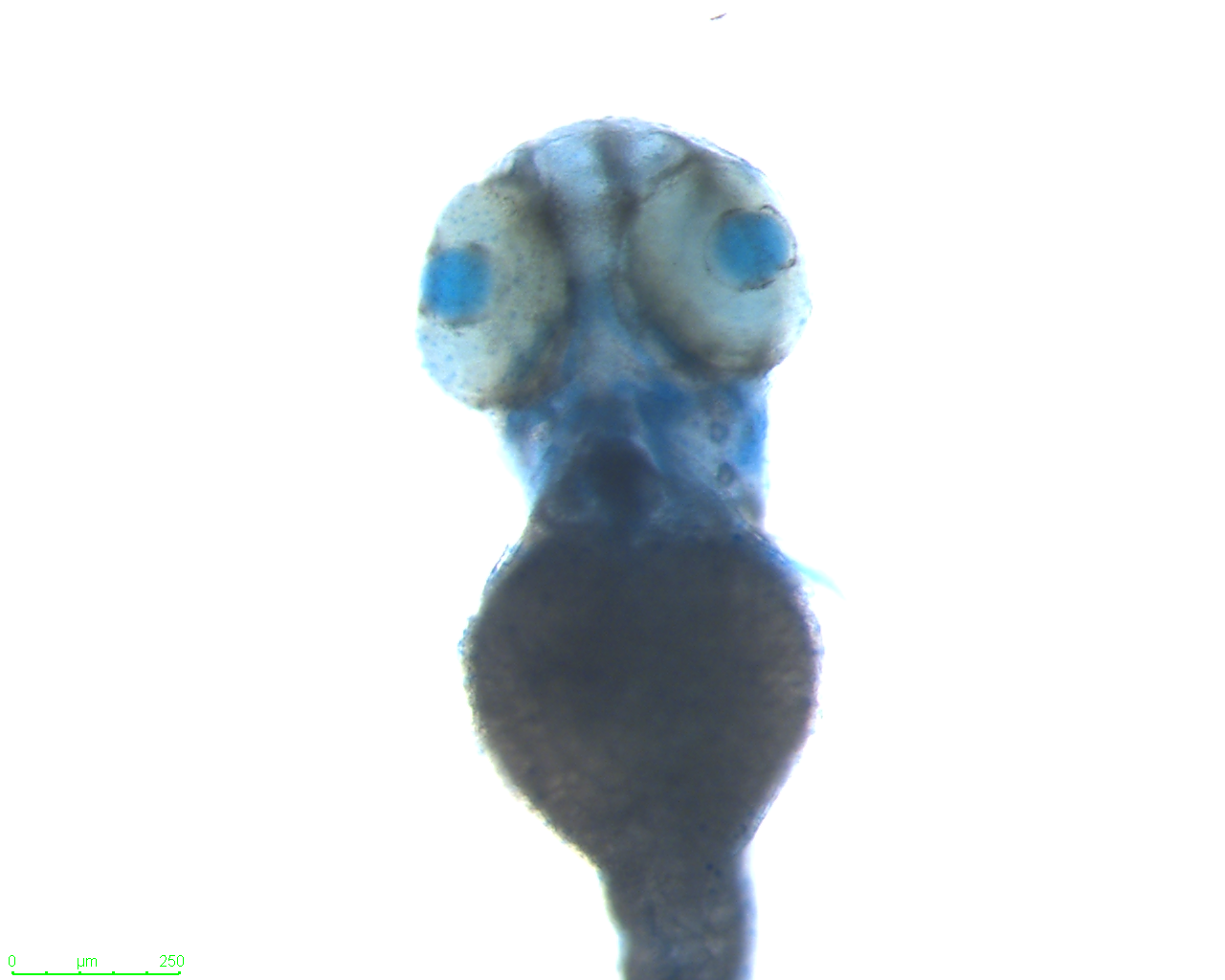

Supplement: Supplementary file 6 — Source Data [file 41467_2021_21053_MOESM6_ESM.zip › Source Data/Zebrafish Morpholino work/Third replicate/EIF5A images 090219_Control_UNT_06.tif]

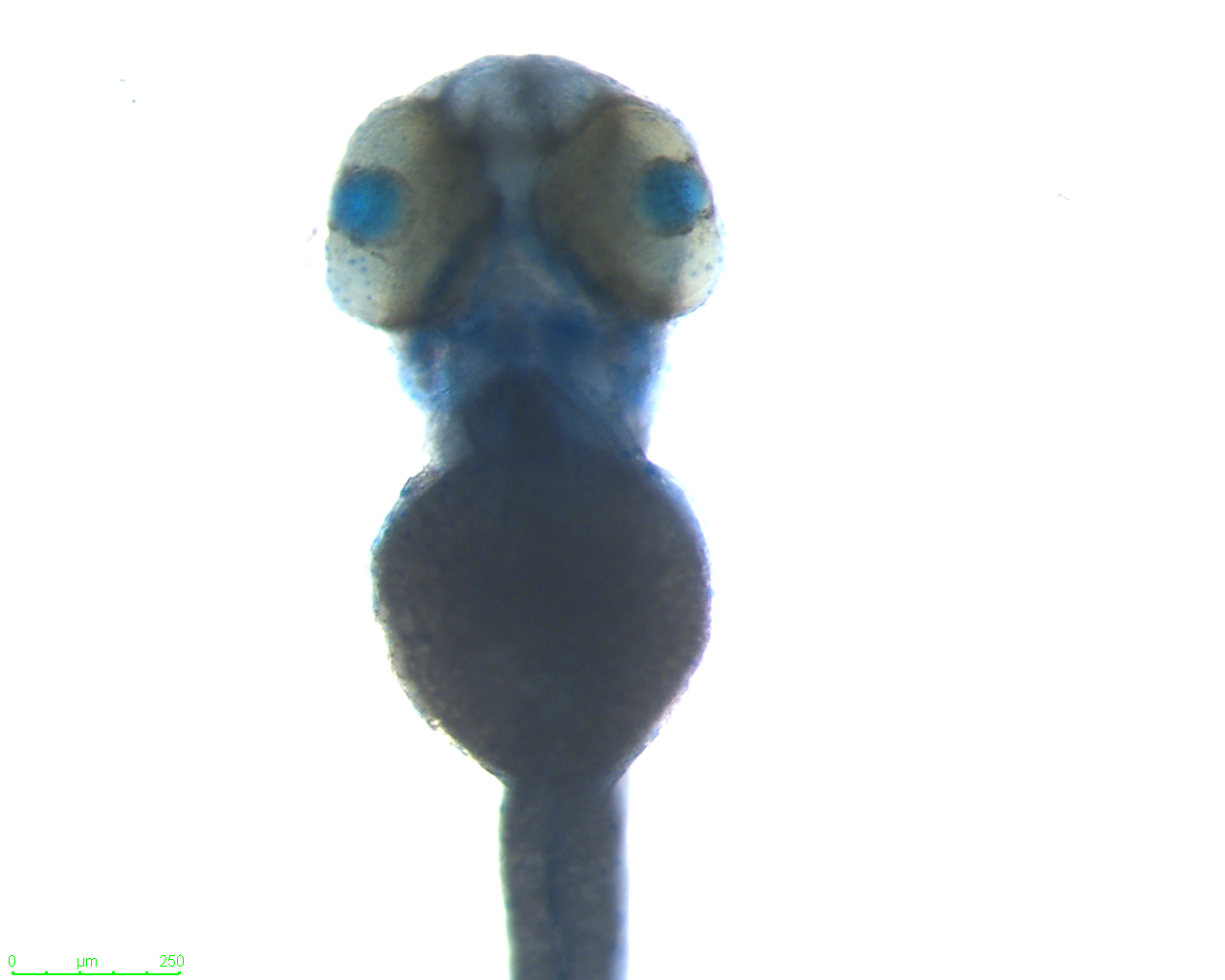

Supplement: Supplementary file 6 — Source Data [file 41467_2021_21053_MOESM6_ESM.zip › Source Data/Zebrafish Morpholino work/Third replicate/EIF5A images 090219_Control_UNT_07.tif]

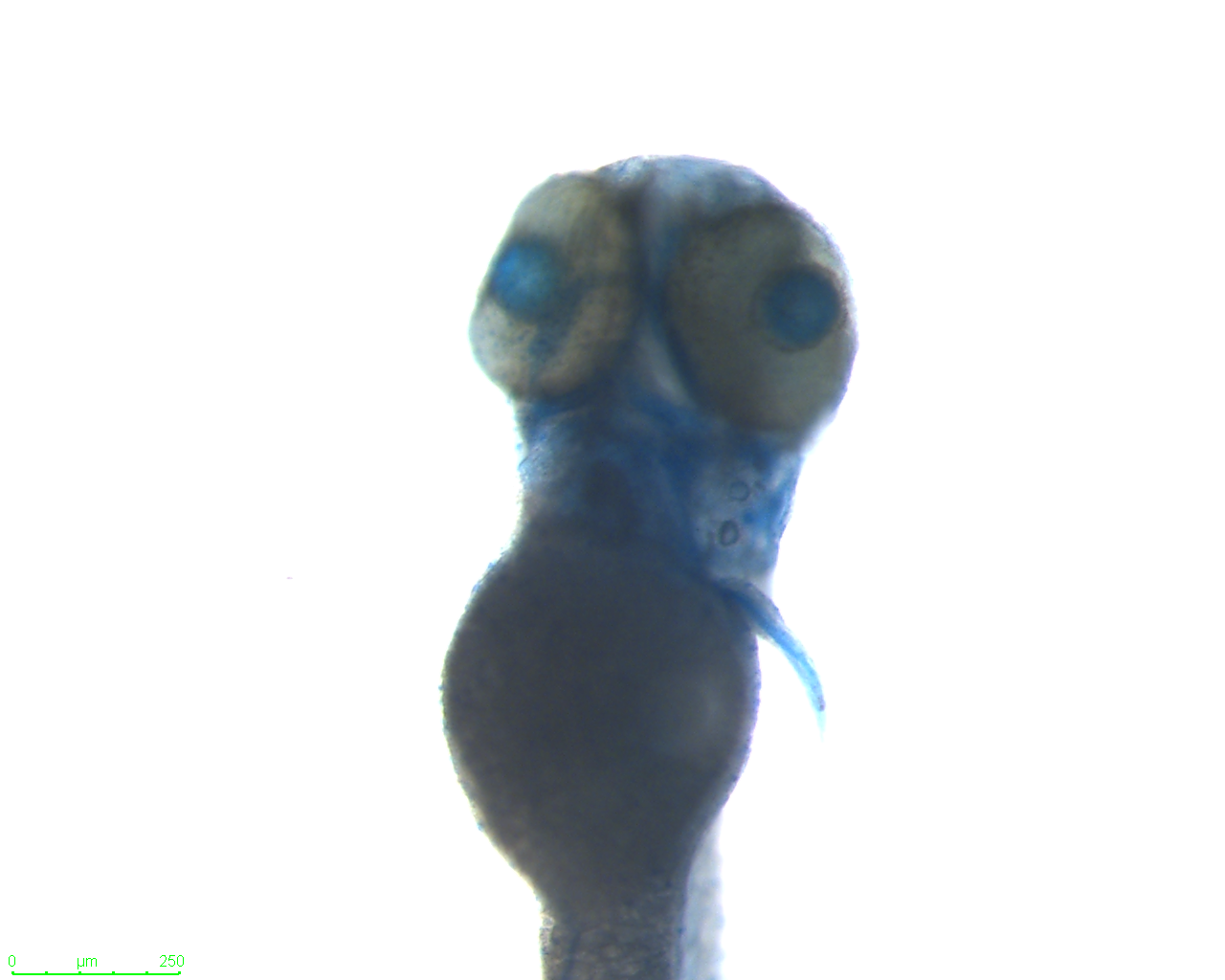

Supplement: Supplementary file 6 — Source Data [file 41467_2021_21053_MOESM6_ESM.zip › Source Data/Zebrafish Morpholino work/Third replicate/EIF5A images 090219_Control_UNT_08.tif]

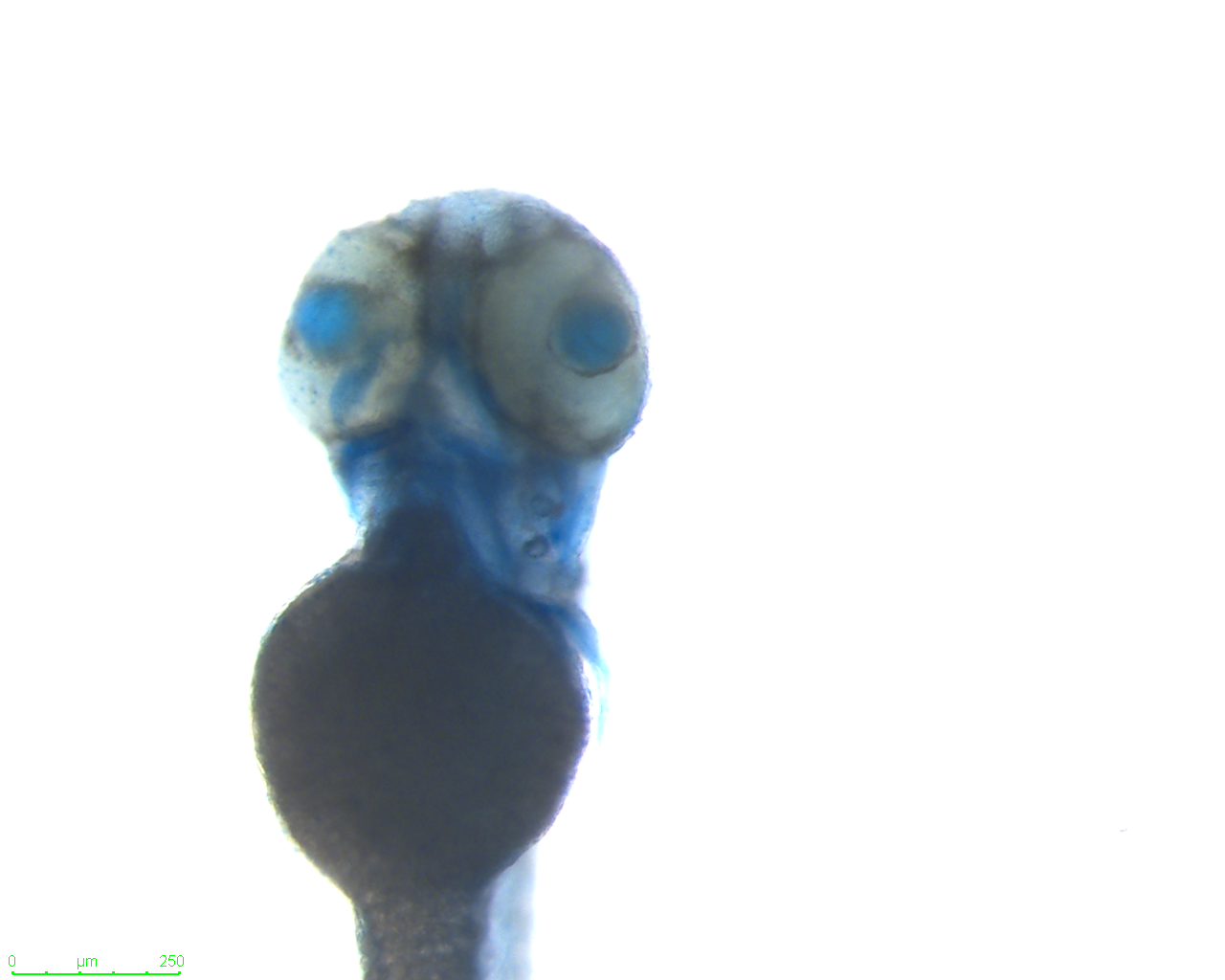

Supplement: Supplementary file 6 — Source Data [file 41467_2021_21053_MOESM6_ESM.zip › Source Data/Zebrafish Morpholino work/Third replicate/EIF5A images 090219_Control_UNT_09.tif]

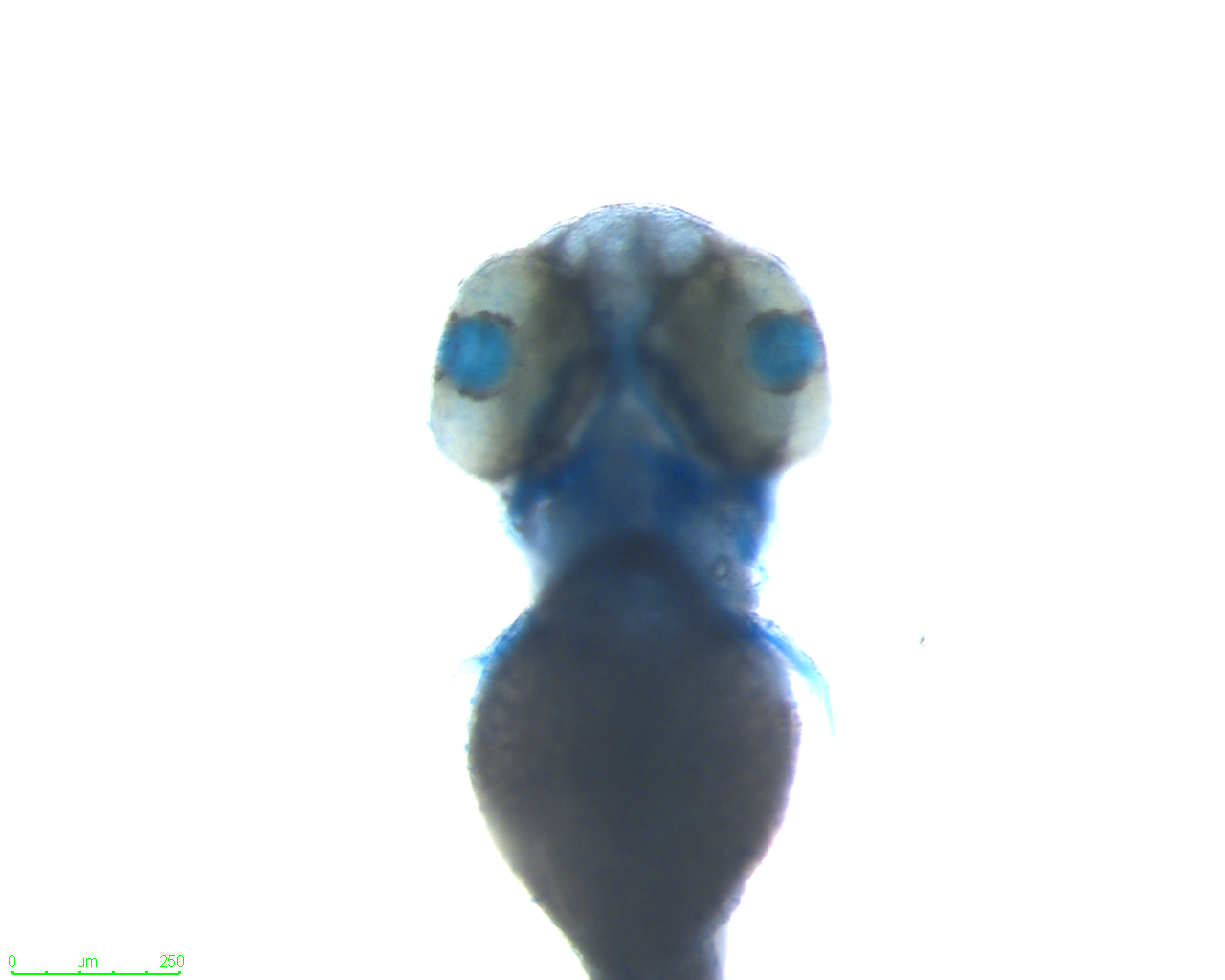

Supplement: Supplementary file 6 — Source Data [file 41467_2021_21053_MOESM6_ESM.zip › Source Data/Zebrafish Morpholino work/Third replicate/EIF5A images 090219_Control_UNT_10.tif]

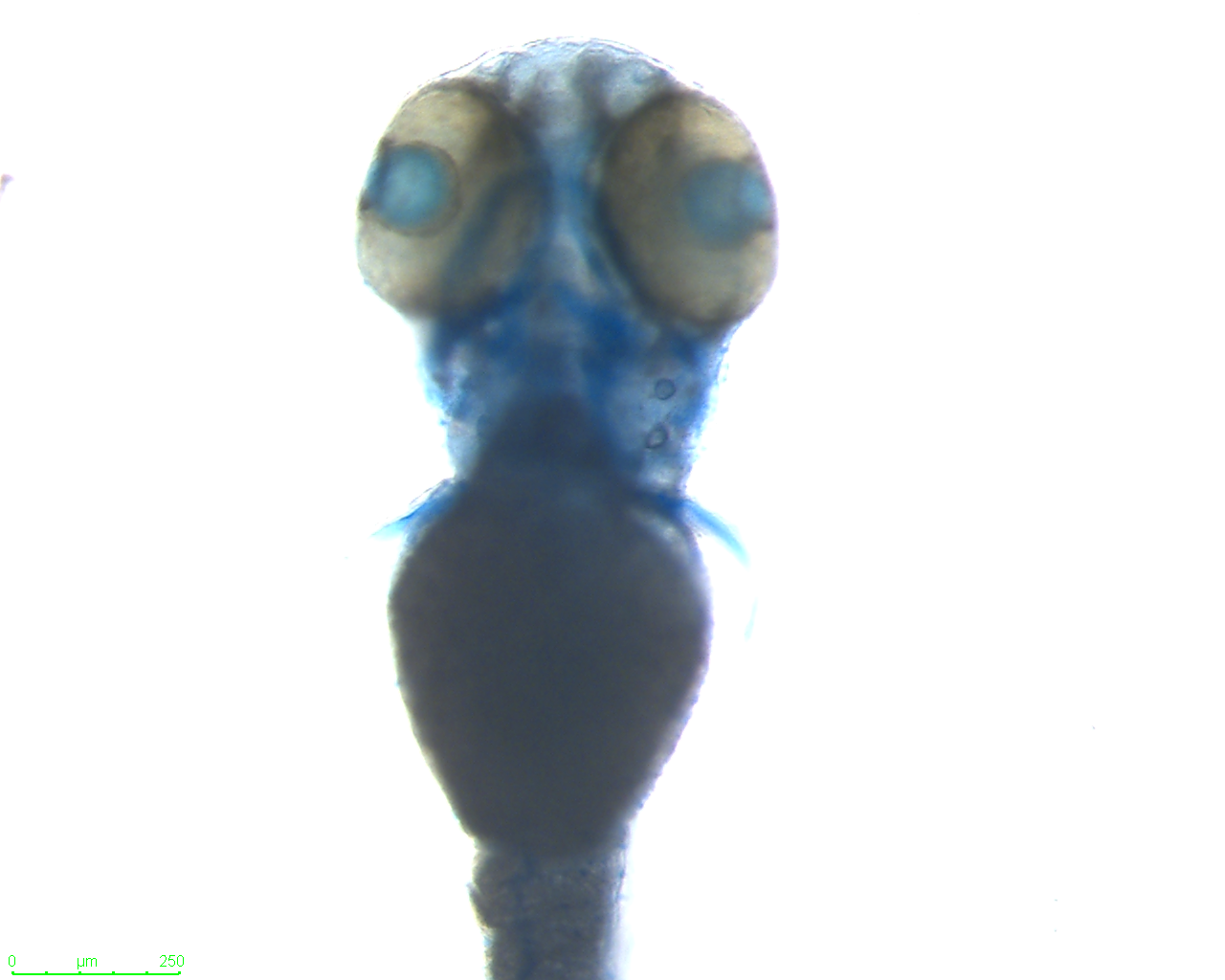

Supplement: Supplementary file 6 — Source Data [file 41467_2021_21053_MOESM6_ESM.zip › Source Data/Zebrafish Morpholino work/Third replicate/EIF5A images 090219_Control_UNT_11.tif]

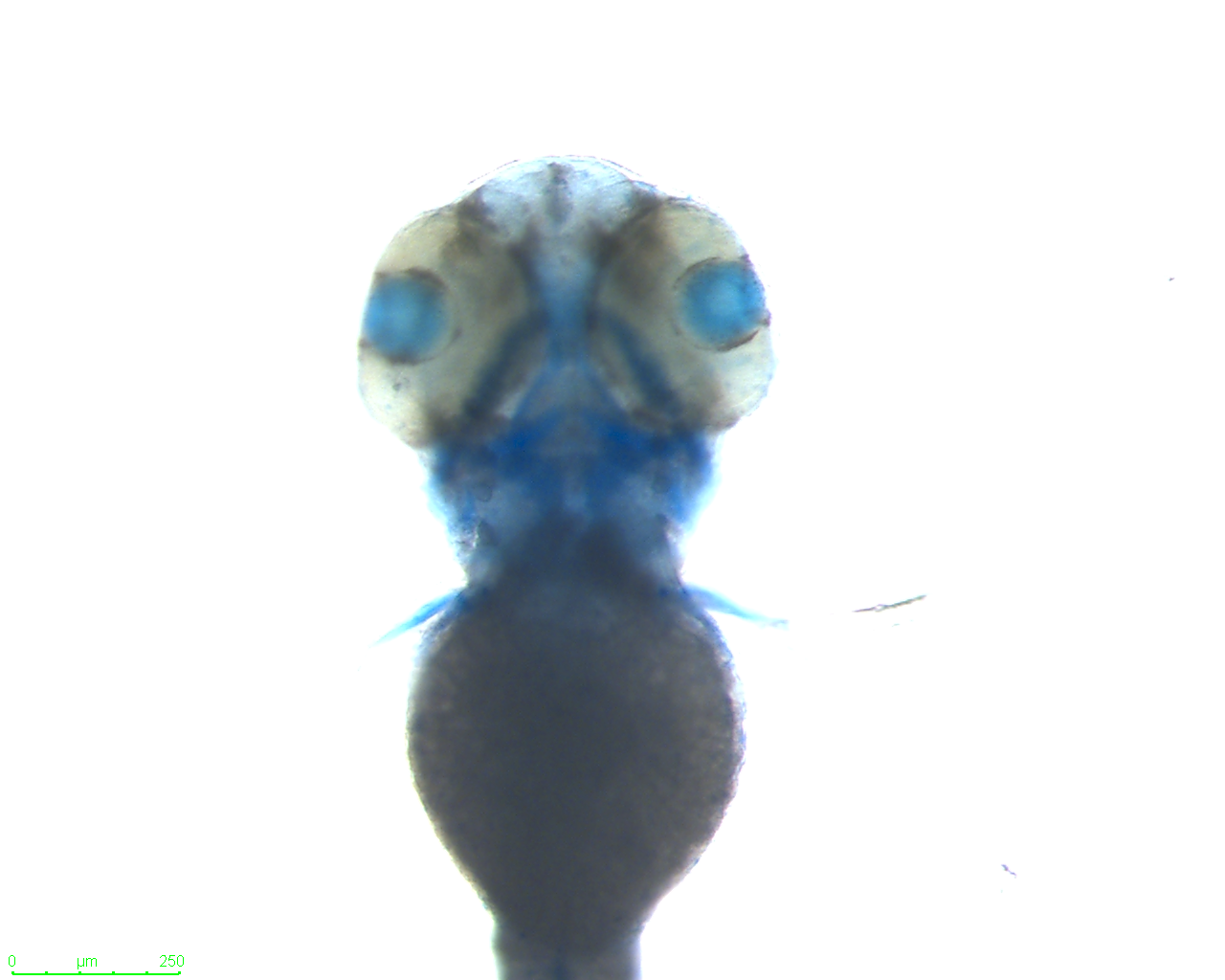

Supplement: Supplementary file 6 — Source Data [file 41467_2021_21053_MOESM6_ESM.zip › Source Data/Zebrafish Morpholino work/Third replicate/EIF5A images 090219_Control_UNT_12.tif]

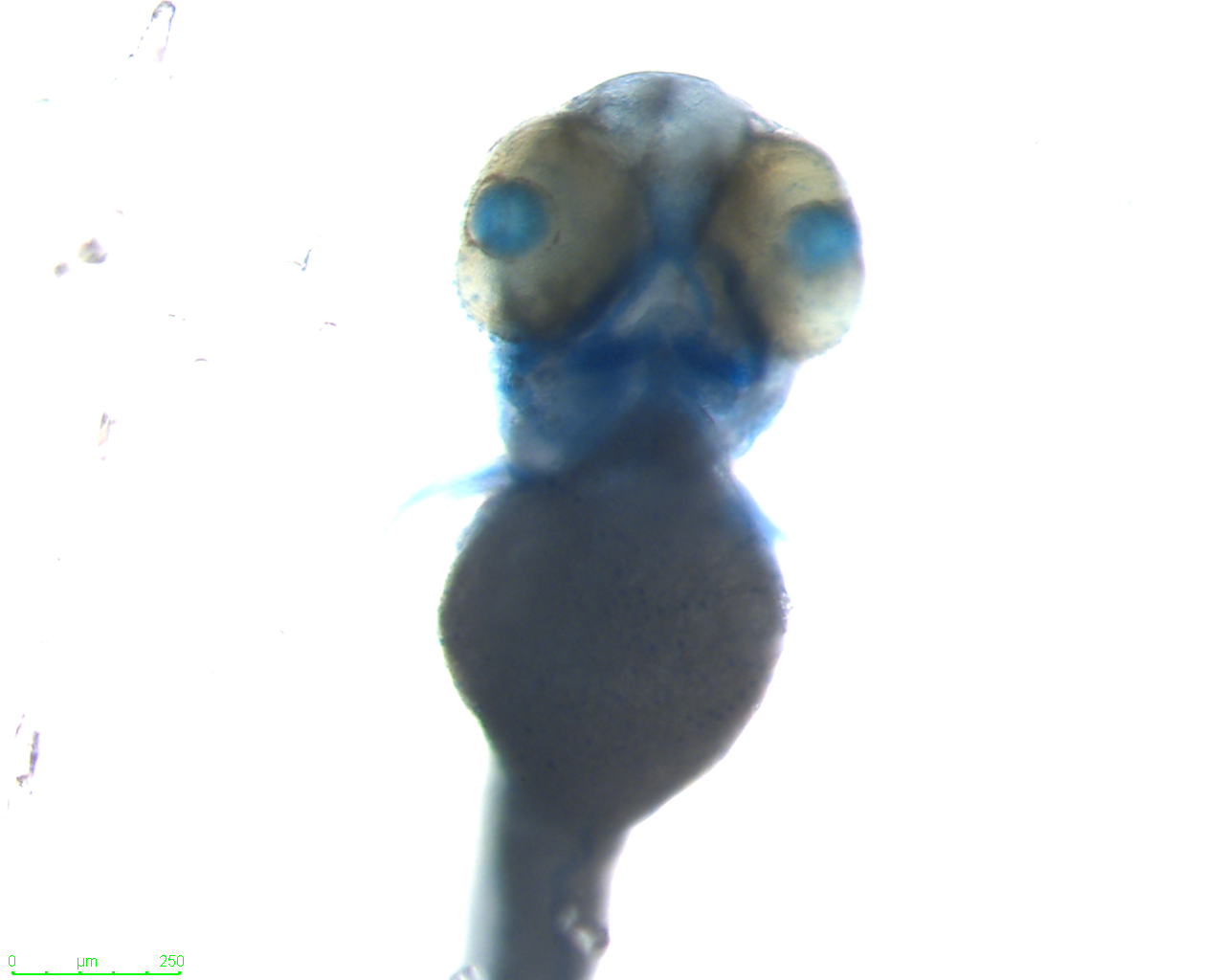

Supplement: Supplementary file 6 — Source Data [file 41467_2021_21053_MOESM6_ESM.zip › Source Data/Zebrafish Morpholino work/Third replicate/EIF5A images 090219_Control_UNT_13.tif]

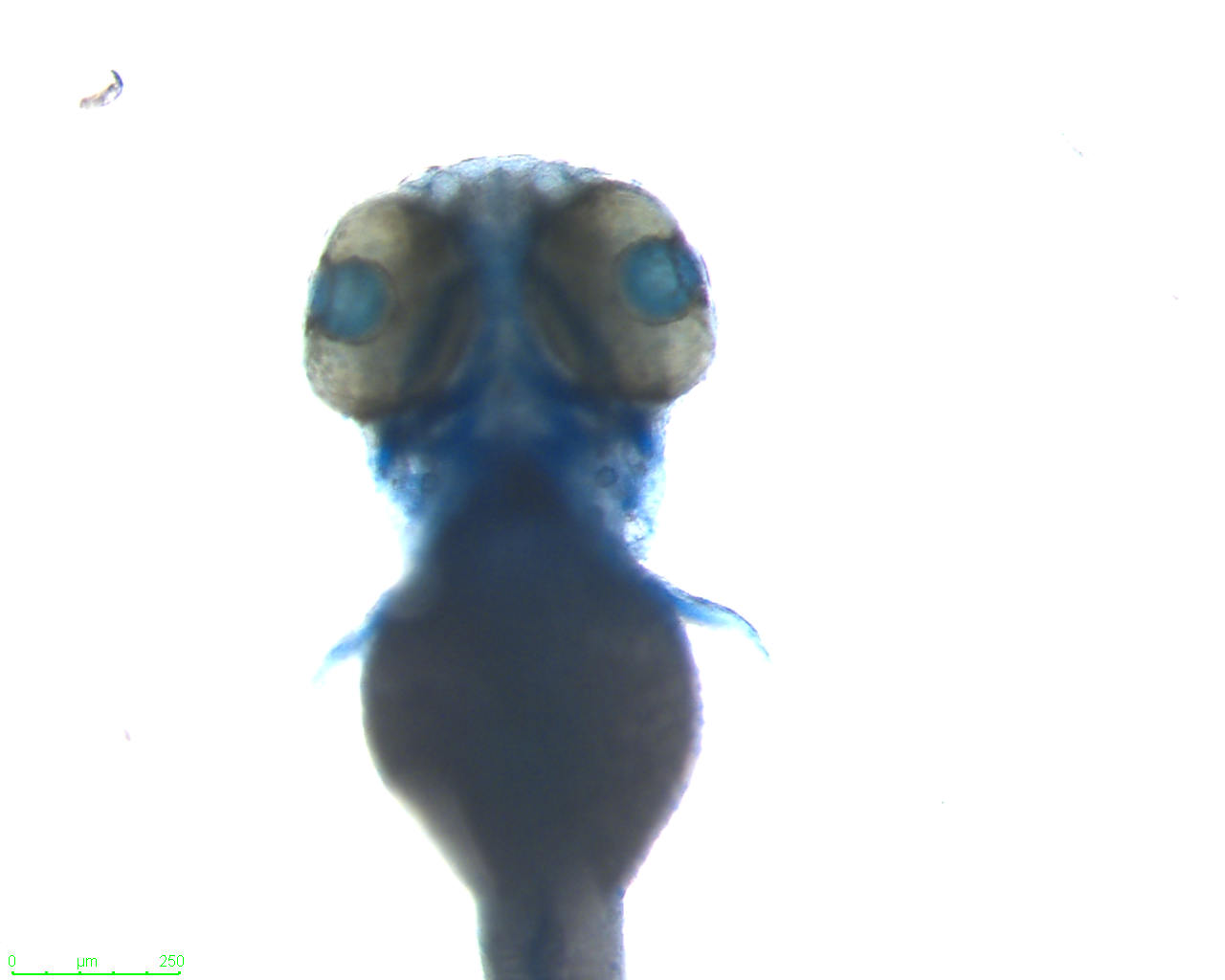

Supplement: Supplementary file 6 — Source Data [file 41467_2021_21053_MOESM6_ESM.zip › Source Data/Zebrafish Morpholino work/Third replicate/EIF5A images 090219_Control_UNT_14.tif]

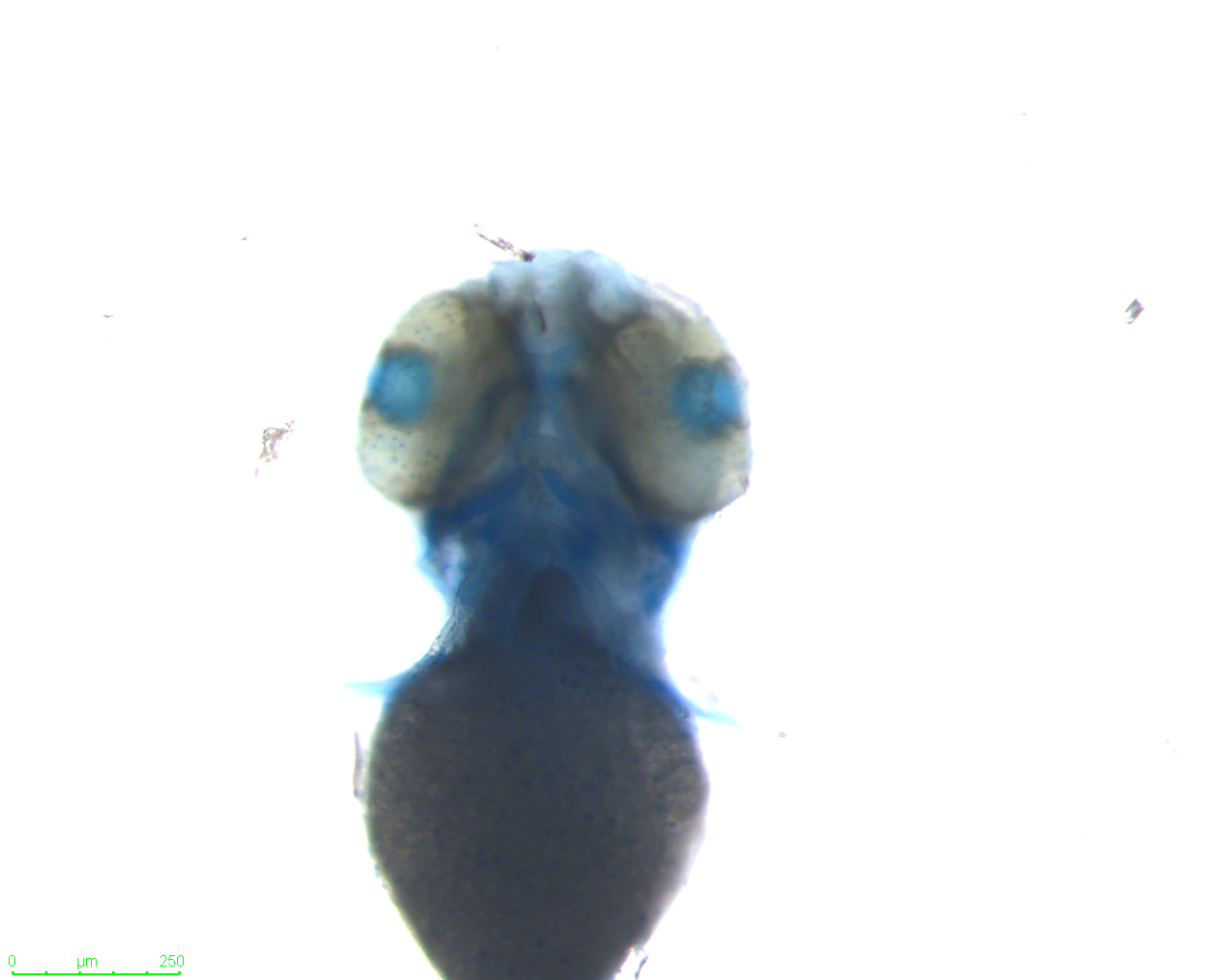

Supplement: Supplementary file 6 — Source Data [file 41467_2021_21053_MOESM6_ESM.zip › Source Data/Zebrafish Morpholino work/Third replicate/EIF5A images 090219_Control_UNT_15.tif]

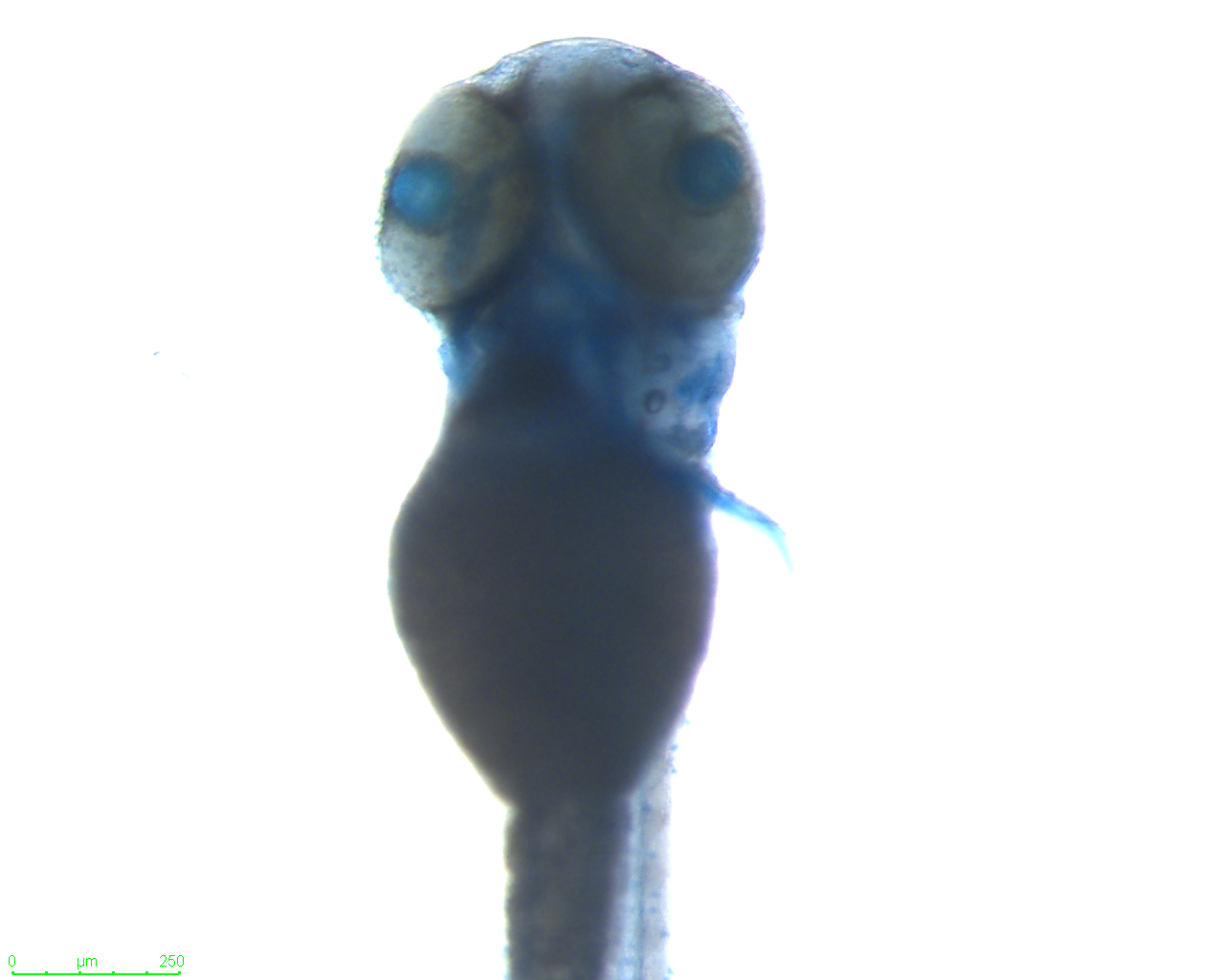

Supplement: Supplementary file 6 — Source Data [file 41467_2021_21053_MOESM6_ESM.zip › Source Data/Zebrafish Morpholino work/Third replicate/EIF5A images 090219_EIF5a_Sperm_01.tif]

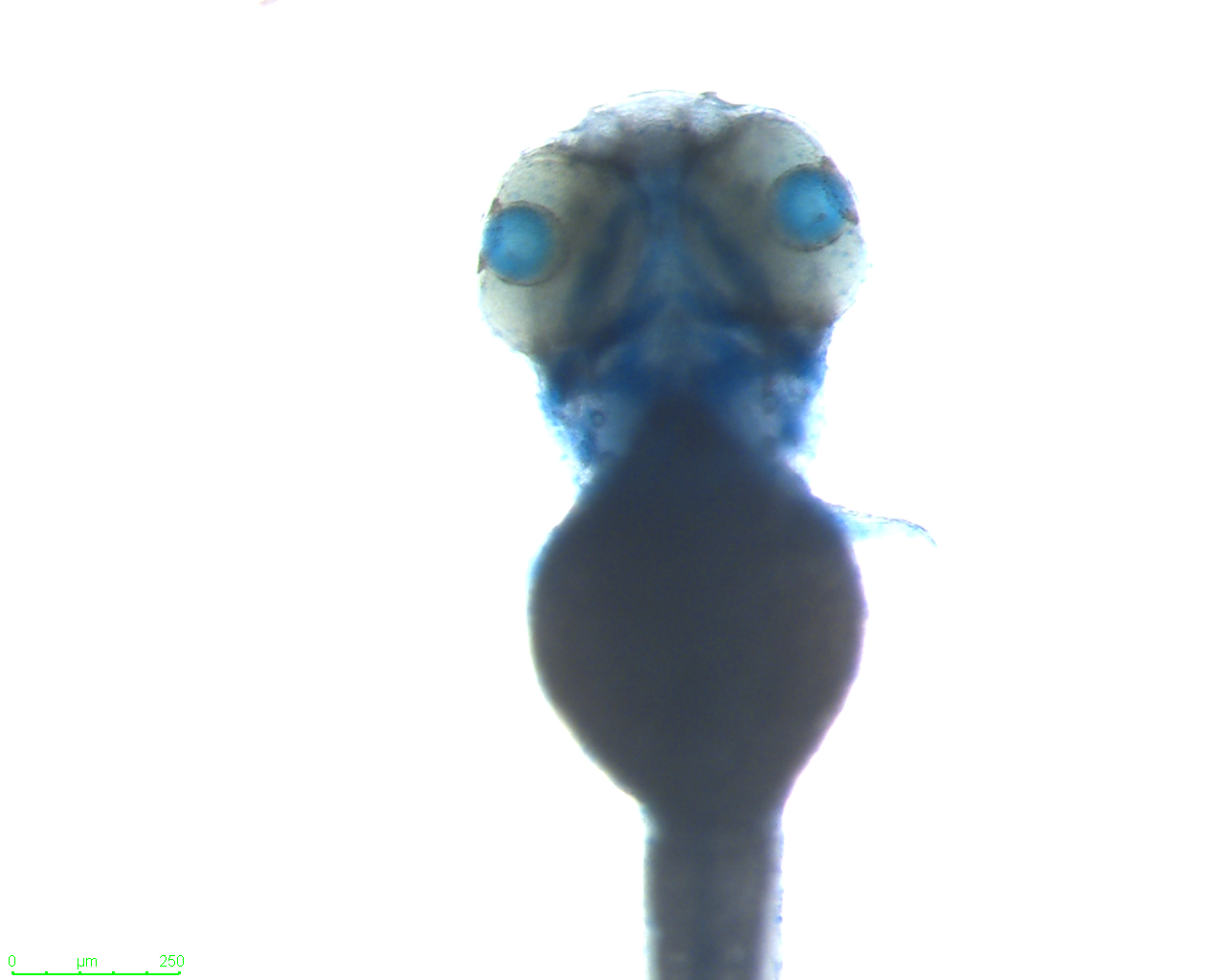

Supplement: Supplementary file 6 — Source Data [file 41467_2021_21053_MOESM6_ESM.zip › Source Data/Zebrafish Morpholino work/Third replicate/EIF5A images 090219_EIF5a_Sperm_02.tif]

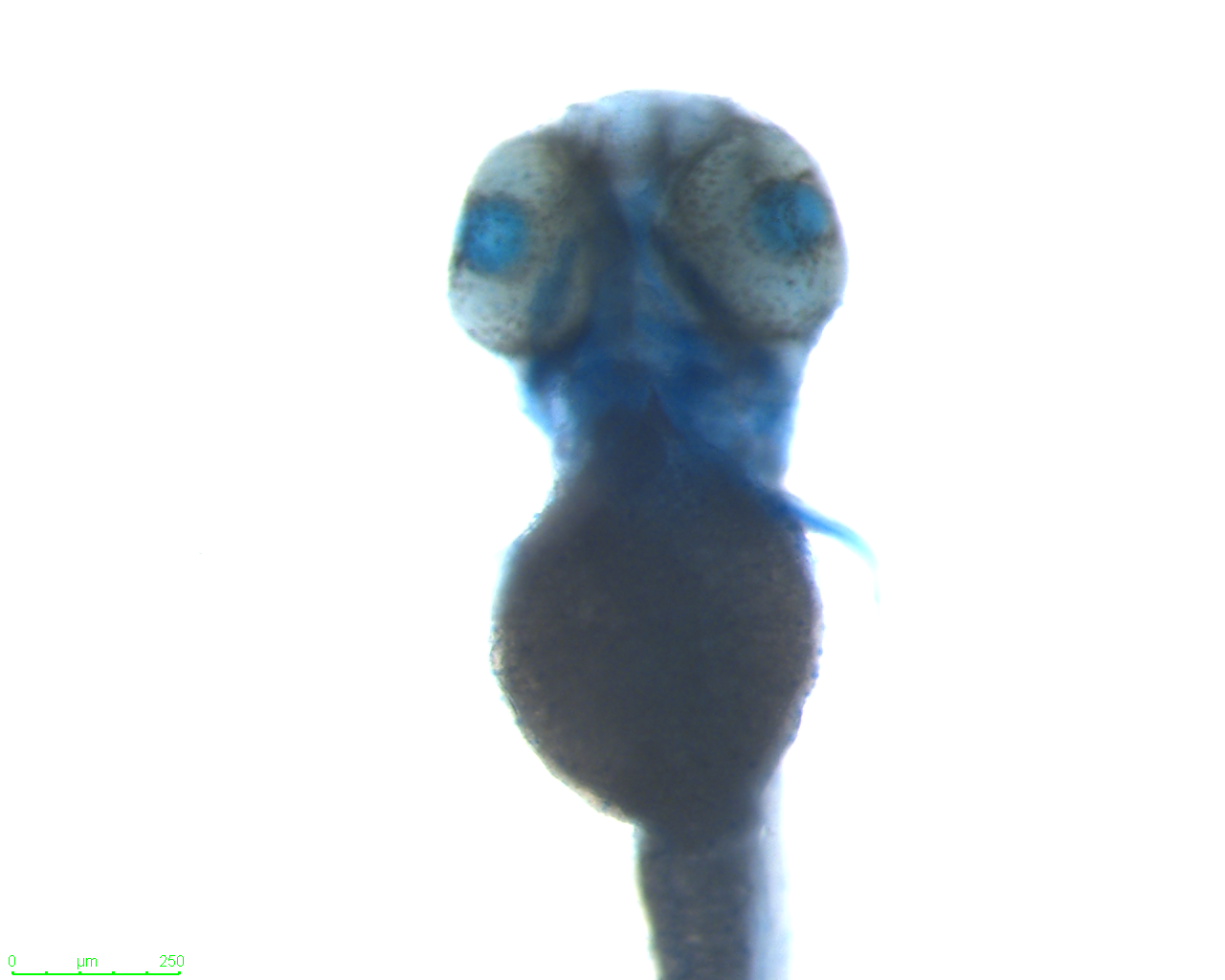

Supplement: Supplementary file 6 — Source Data [file 41467_2021_21053_MOESM6_ESM.zip › Source Data/Zebrafish Morpholino work/Third replicate/EIF5A images 090219_EIF5a_Sperm_03.tif]

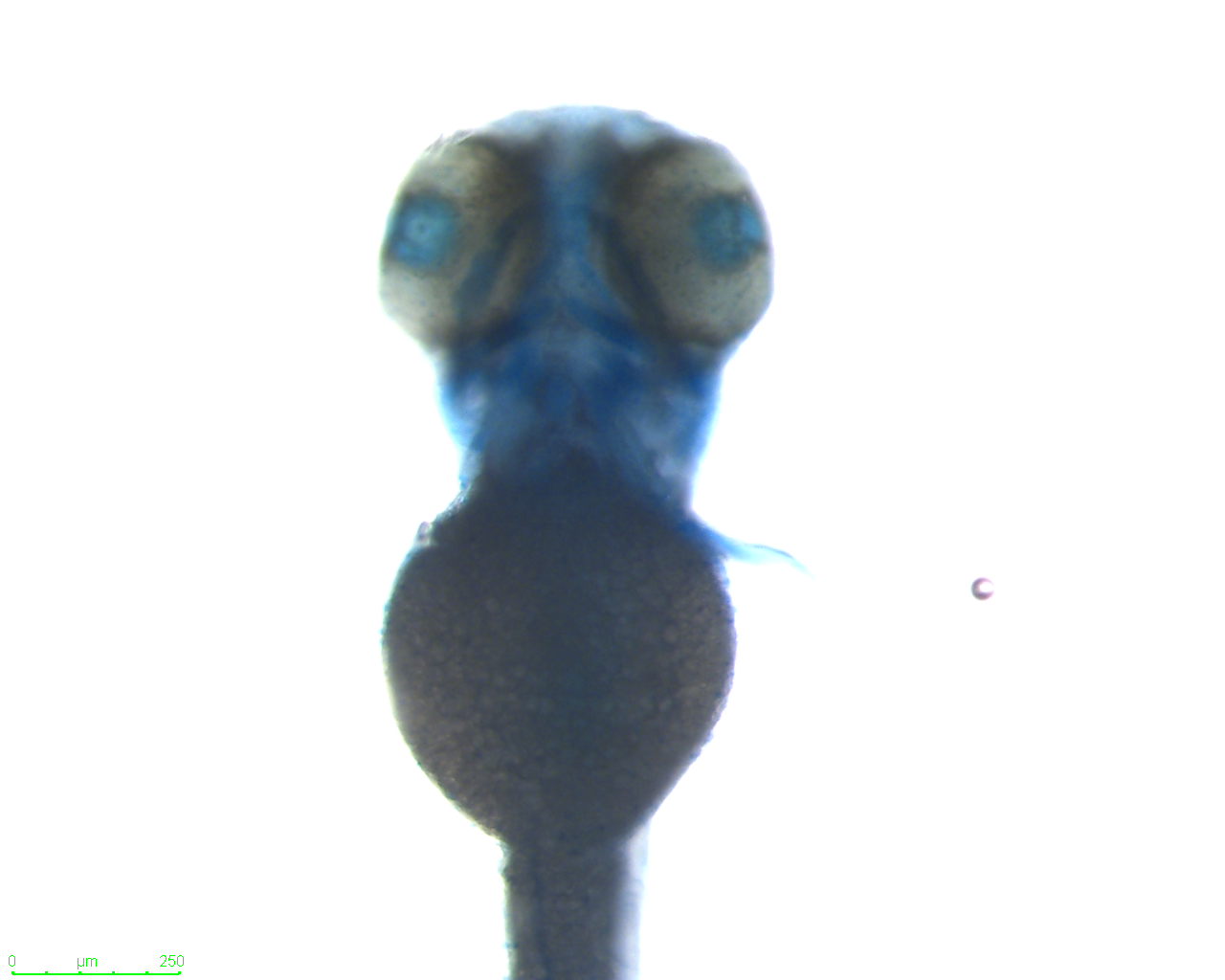

Supplement: Supplementary file 6 — Source Data [file 41467_2021_21053_MOESM6_ESM.zip › Source Data/Zebrafish Morpholino work/Third replicate/EIF5A images 090219_EIF5a_Sperm_04.tif]

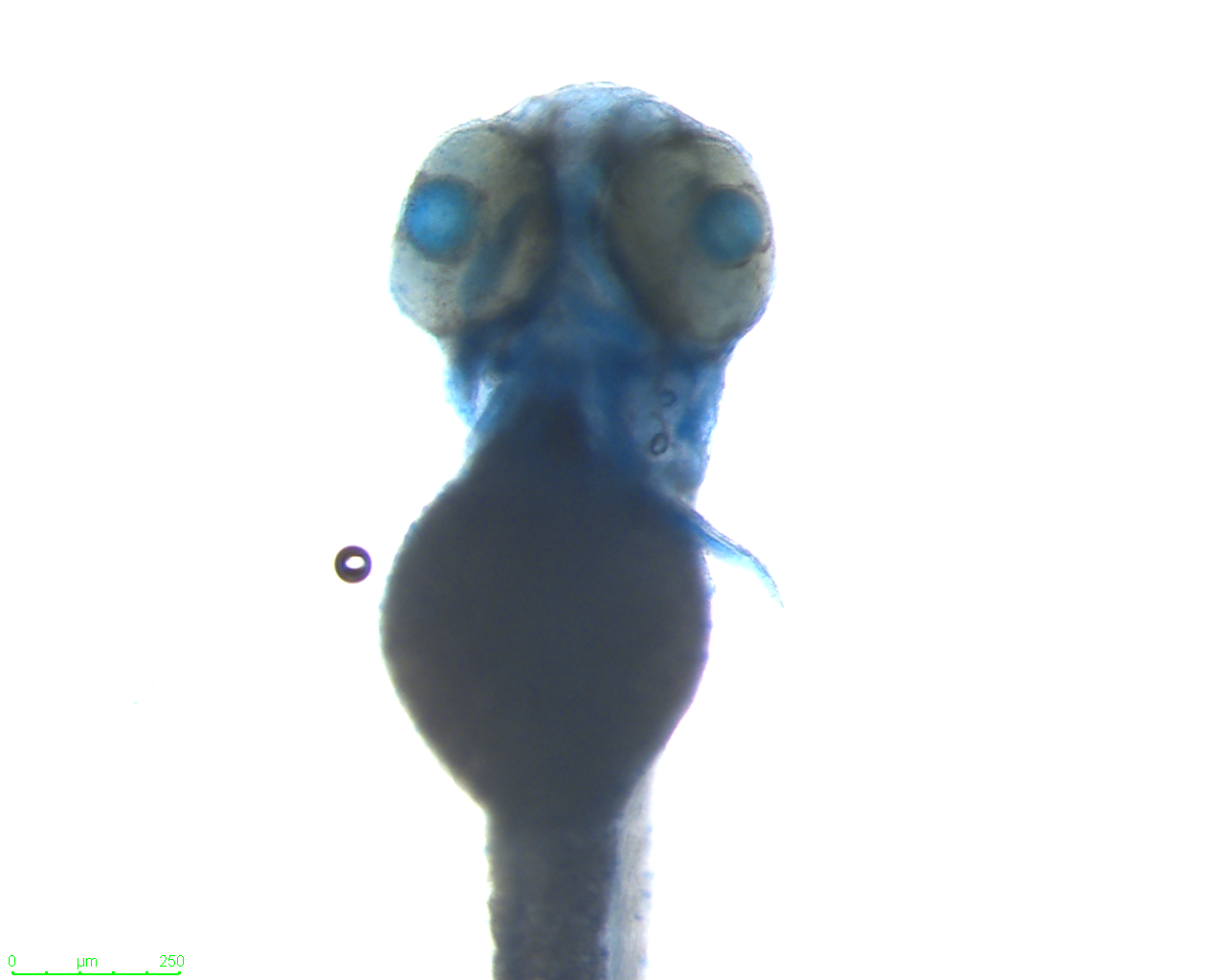

Supplement: Supplementary file 6 — Source Data [file 41467_2021_21053_MOESM6_ESM.zip › Source Data/Zebrafish Morpholino work/Third replicate/EIF5A images 090219_EIF5a_Sperm_05.tif]

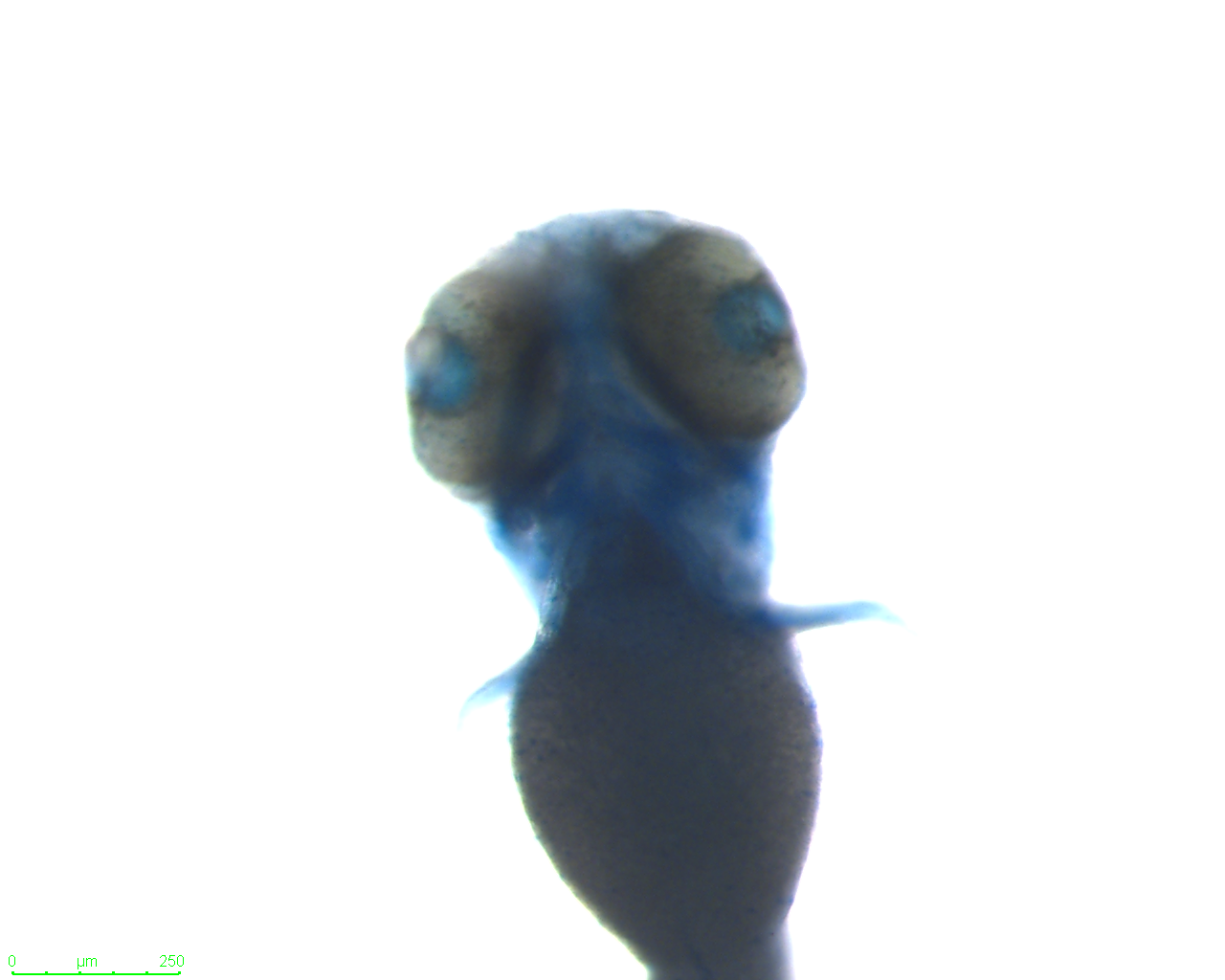

Supplement: Supplementary file 6 — Source Data [file 41467_2021_21053_MOESM6_ESM.zip › Source Data/Zebrafish Morpholino work/Third replicate/EIF5A images 090219_EIF5a_Sperm_06.tif]

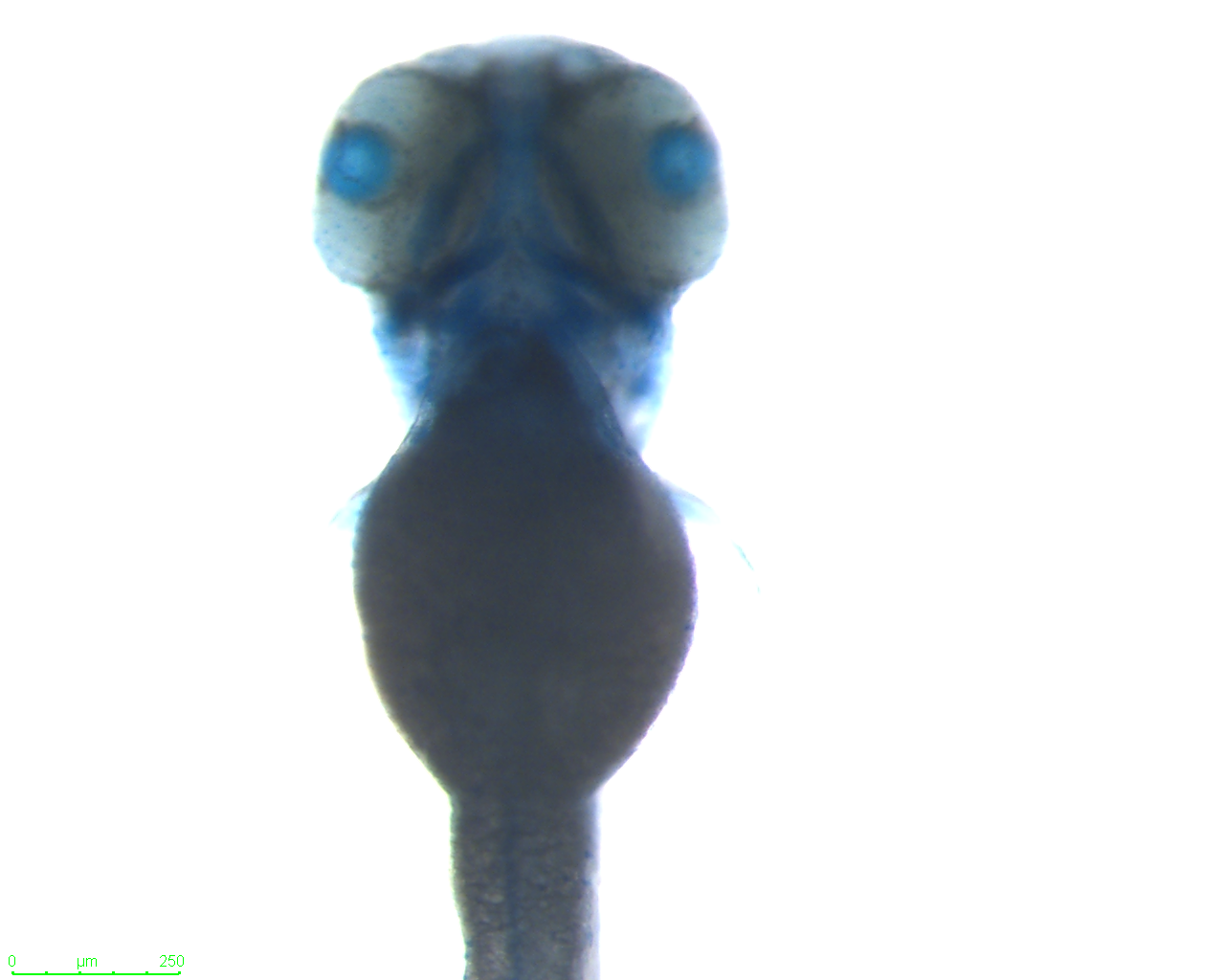

Supplement: Supplementary file 6 — Source Data [file 41467_2021_21053_MOESM6_ESM.zip › Source Data/Zebrafish Morpholino work/Third replicate/EIF5A images 090219_EIF5a_Sperm_07.tif]

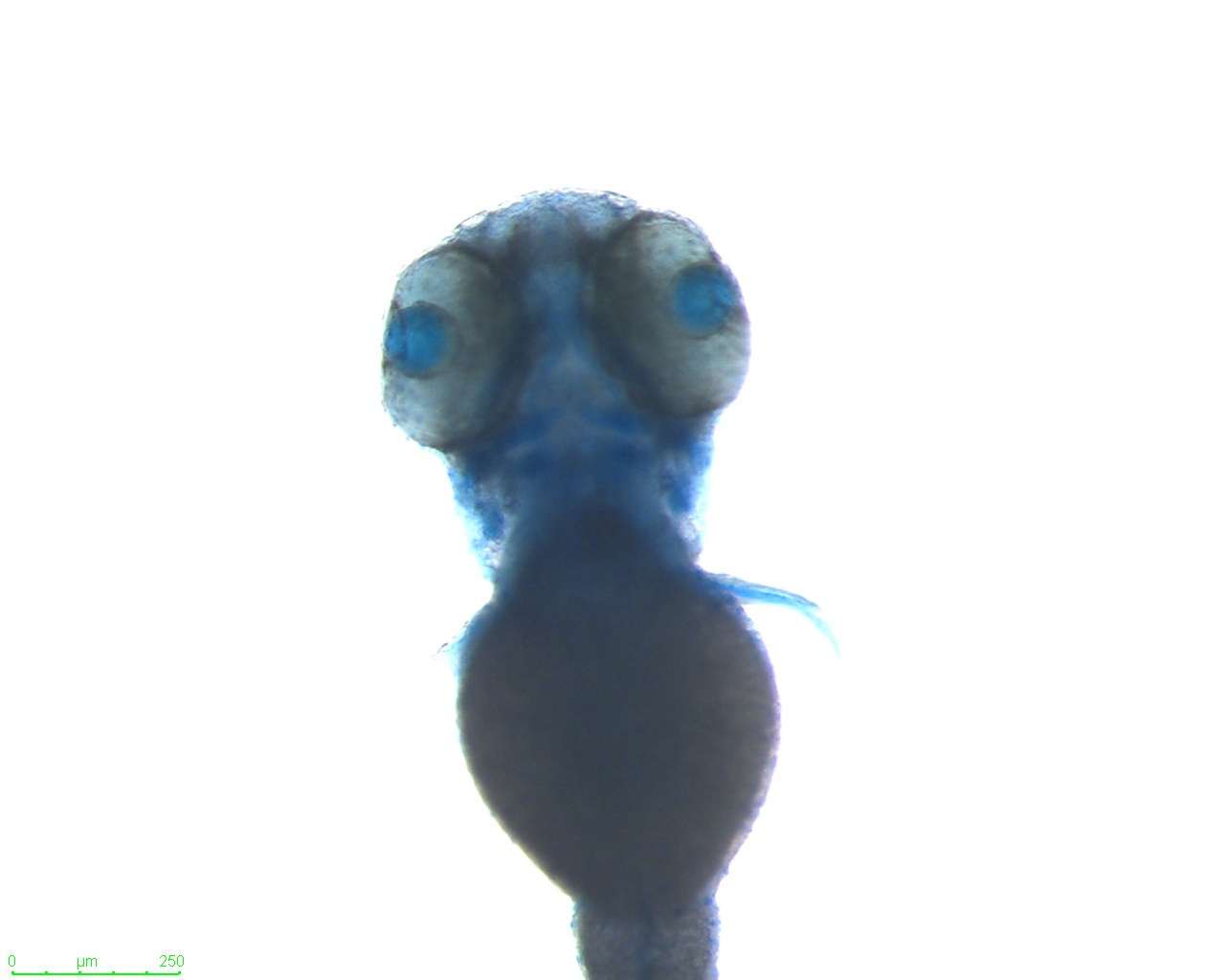

Supplement: Supplementary file 6 — Source Data [file 41467_2021_21053_MOESM6_ESM.zip › Source Data/Zebrafish Morpholino work/Third replicate/EIF5A images 090219_EIF5a_Sperm_08.tif]

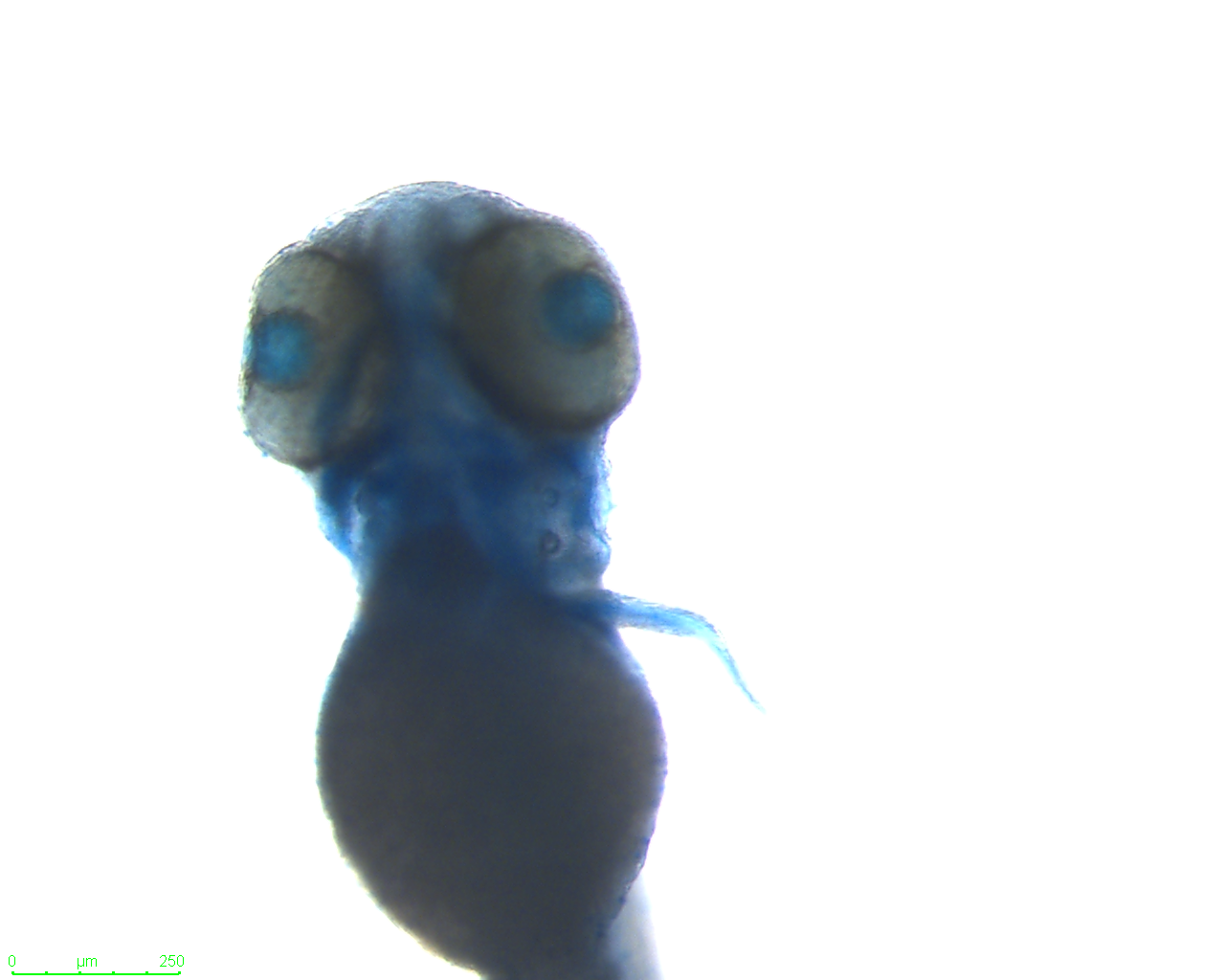

Supplement: Supplementary file 6 — Source Data [file 41467_2021_21053_MOESM6_ESM.zip › Source Data/Zebrafish Morpholino work/Third replicate/EIF5A images 090219_EIF5a_Sperm_09.tif]

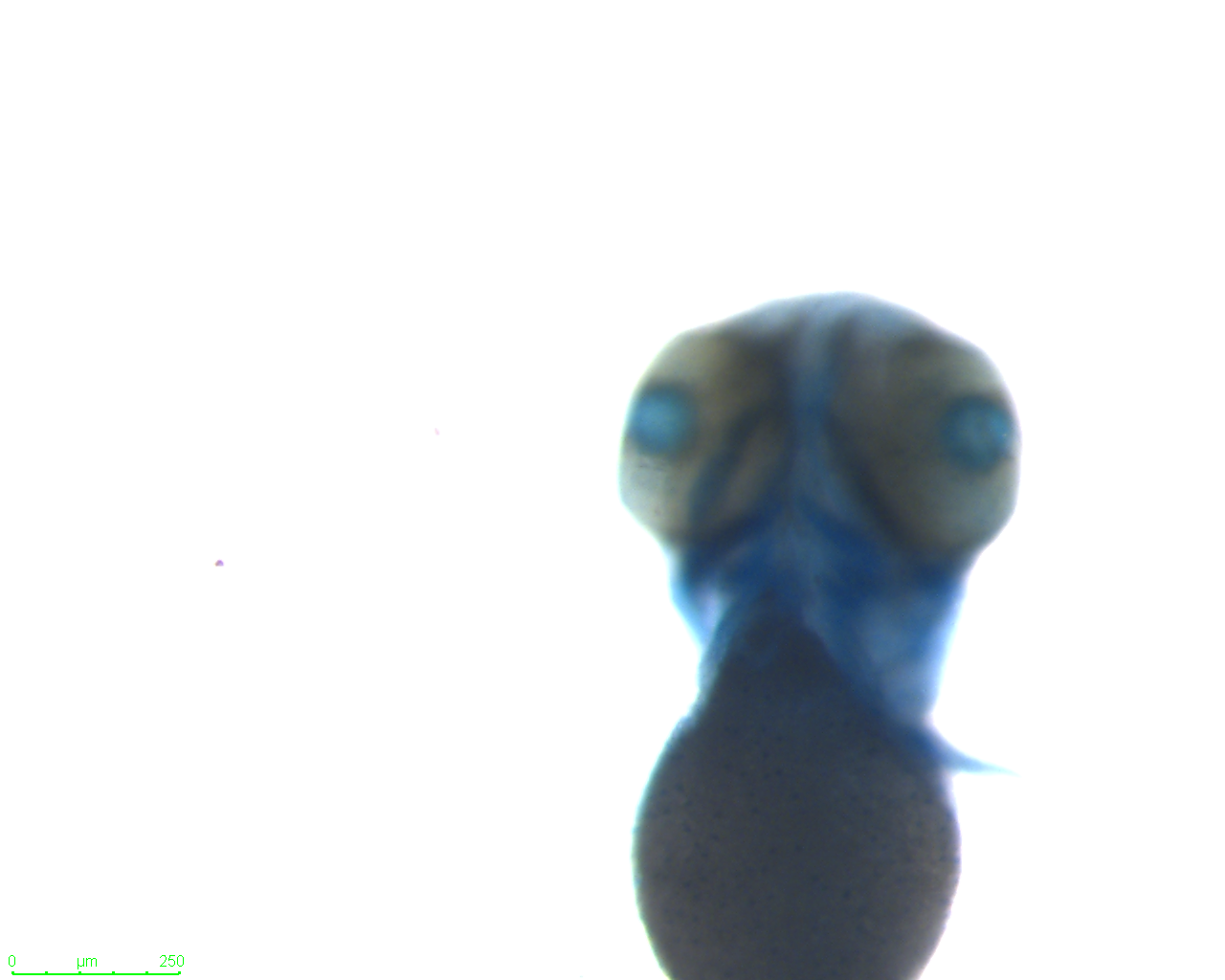

Supplement: Supplementary file 6 — Source Data [file 41467_2021_21053_MOESM6_ESM.zip › Source Data/Zebrafish Morpholino work/Third replicate/EIF5A images 090219_EIF5a_Sperm_10.tif]

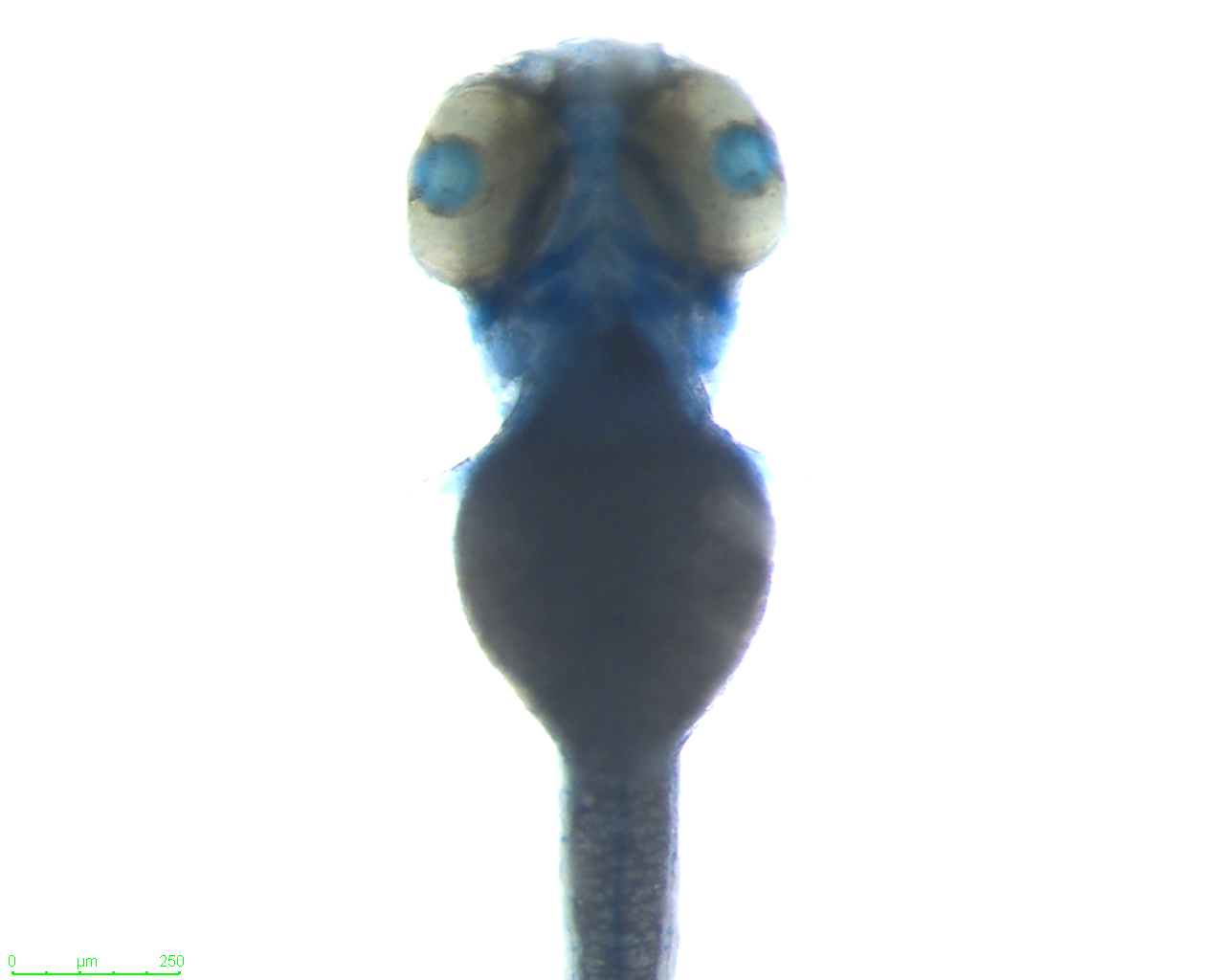

Supplement: Supplementary file 6 — Source Data [file 41467_2021_21053_MOESM6_ESM.zip › Source Data/Zebrafish Morpholino work/Third replicate/EIF5A images 090219_EIF5a_Sperm_11.tif]

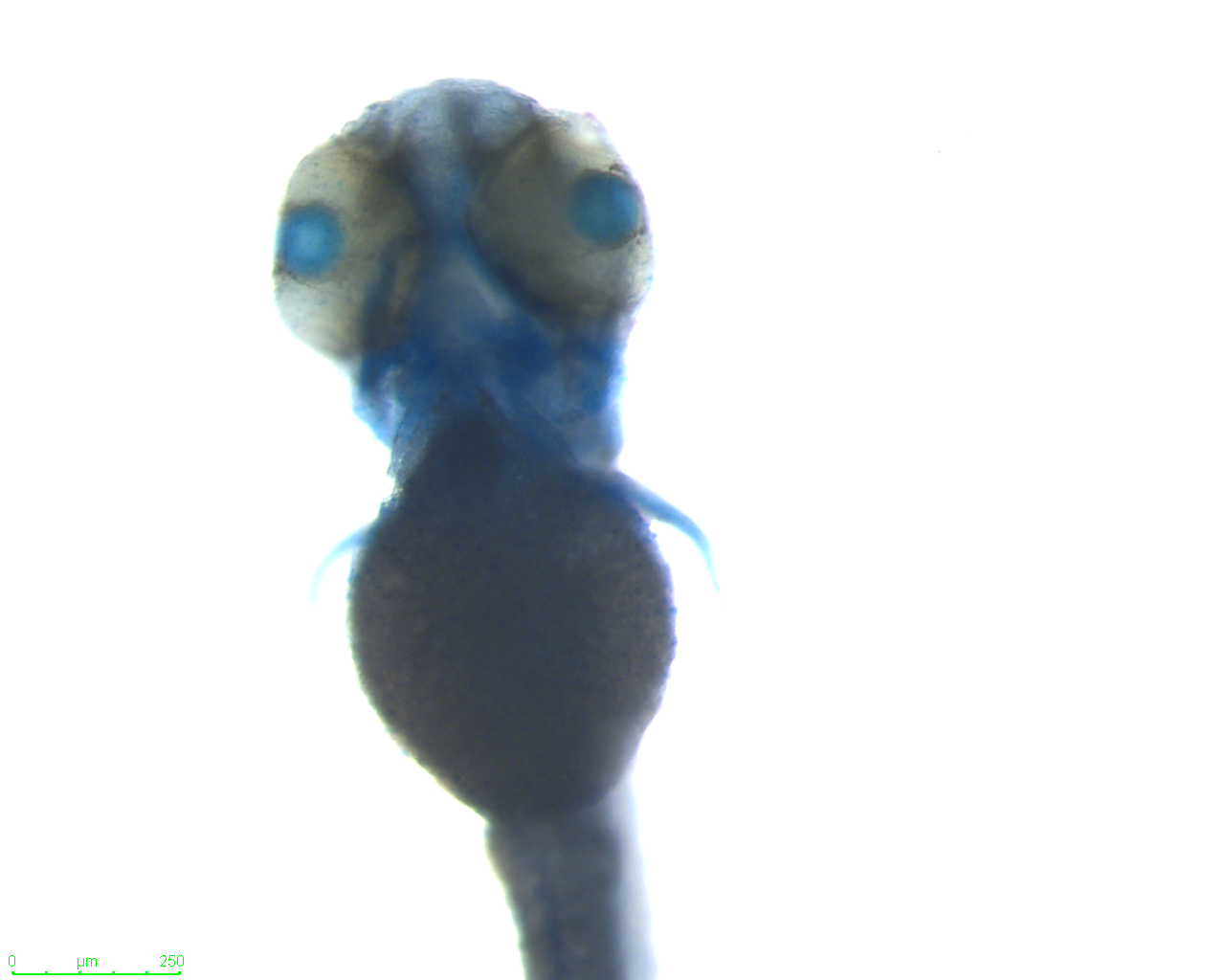

Supplement: Supplementary file 6 — Source Data [file 41467_2021_21053_MOESM6_ESM.zip › Source Data/Zebrafish Morpholino work/Third replicate/EIF5A images 090219_EIF5a_Sperm_12.tif]

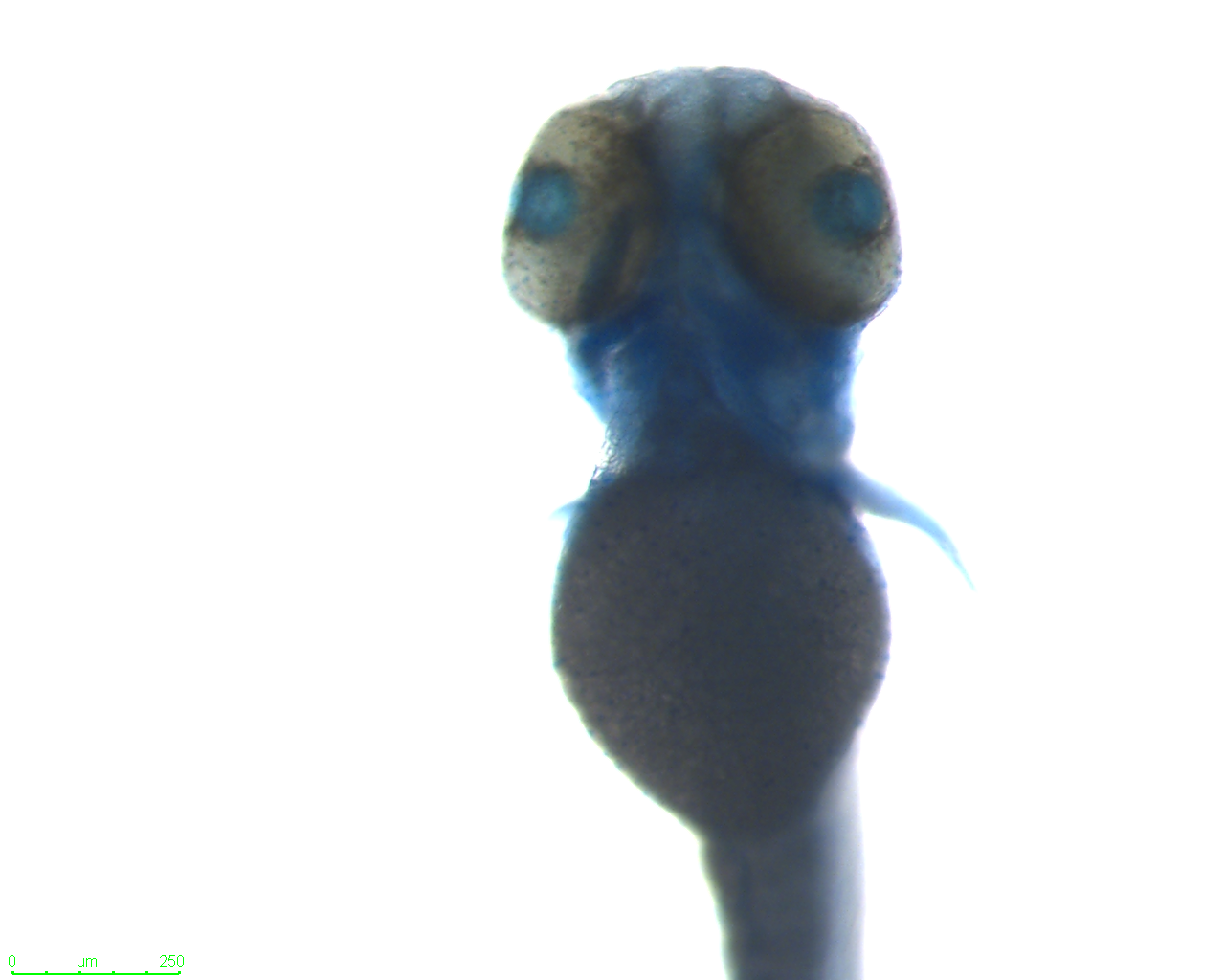

Supplement: Supplementary file 6 — Source Data [file 41467_2021_21053_MOESM6_ESM.zip › Source Data/Zebrafish Morpholino work/Third replicate/EIF5A images 090219_EIF5a_Sperm_13.tif]

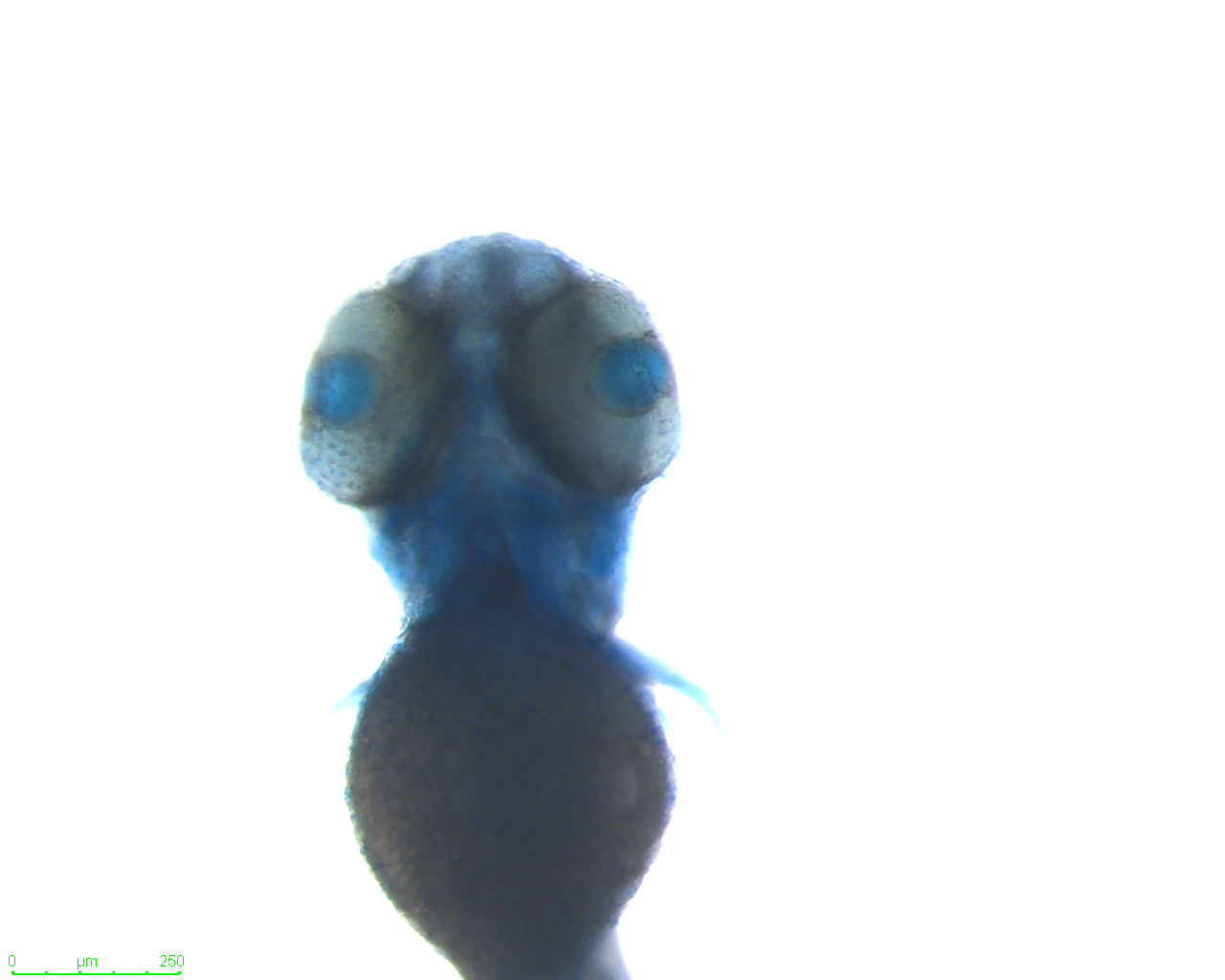

Supplement: Supplementary file 6 — Source Data [file 41467_2021_21053_MOESM6_ESM.zip › Source Data/Zebrafish Morpholino work/Third replicate/EIF5A images 090219_EIF5a_Sperm_14.tif]

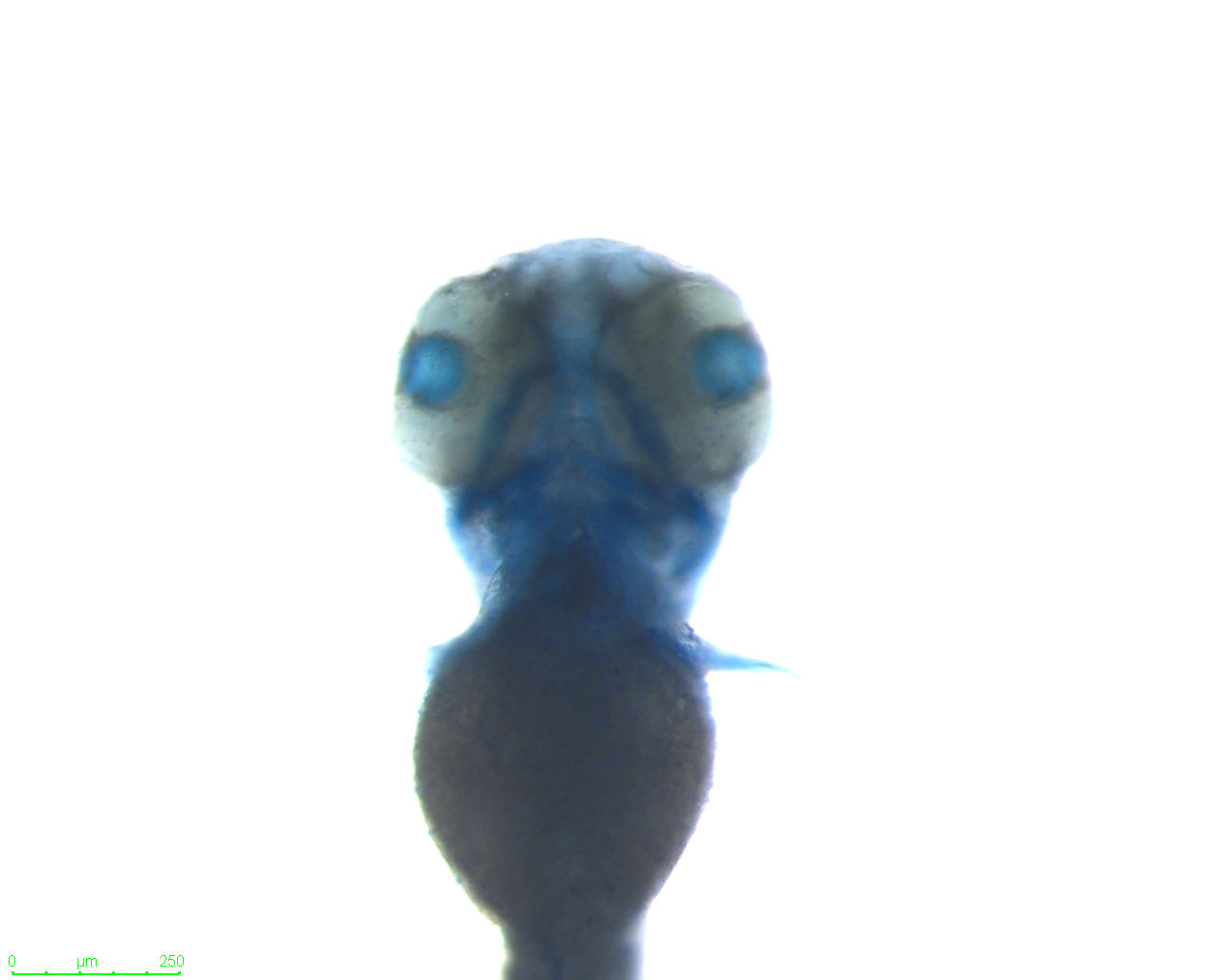

Supplement: Supplementary file 6 — Source Data [file 41467_2021_21053_MOESM6_ESM.zip › Source Data/Zebrafish Morpholino work/Third replicate/EIF5A images 090219_EIF5a_Sperm_15.tif]

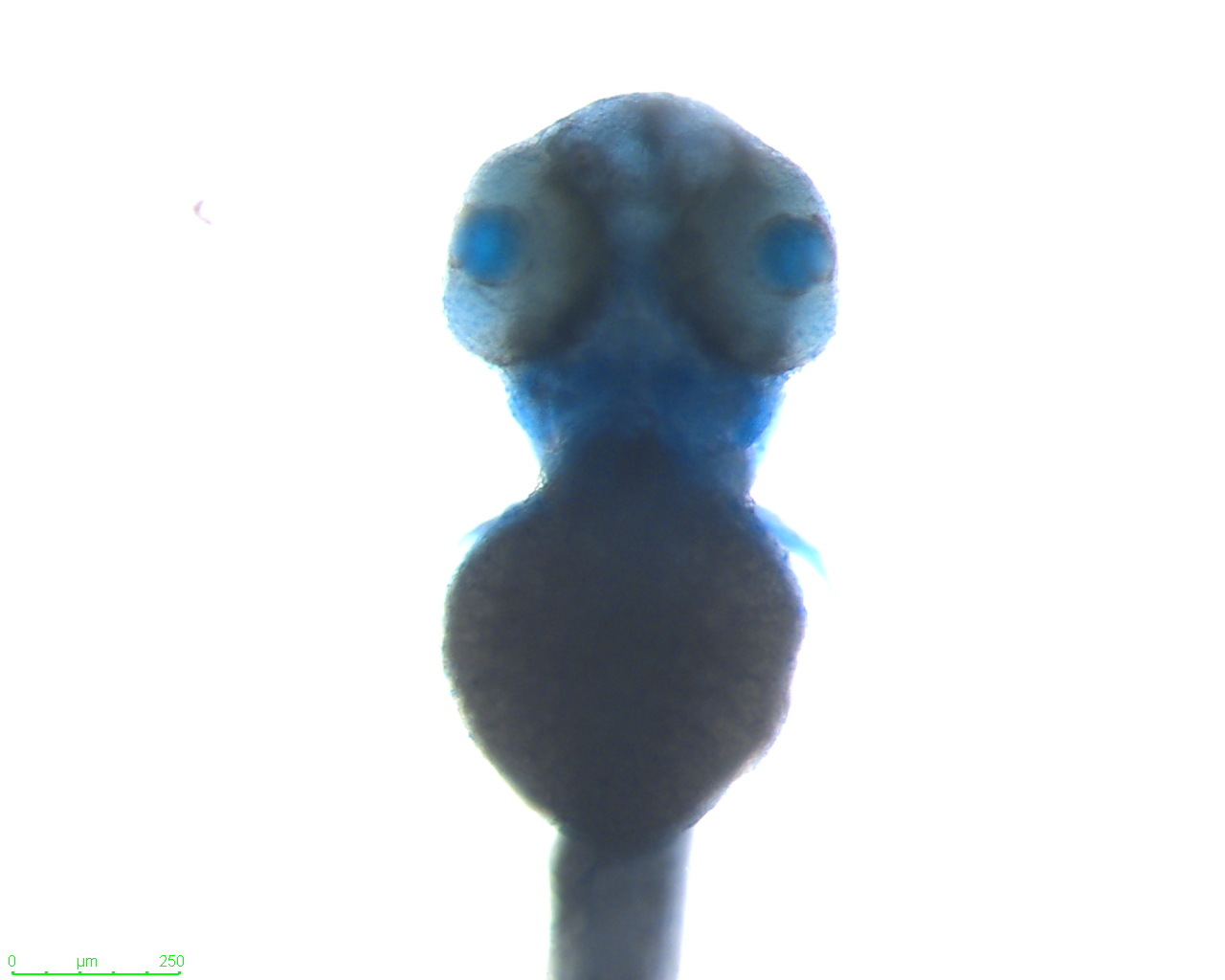

Supplement: Supplementary file 6 — Source Data [file 41467_2021_21053_MOESM6_ESM.zip › Source Data/Zebrafish Morpholino work/Third replicate/EIF5A images 090219_EIF5a_UNT_01.tif]

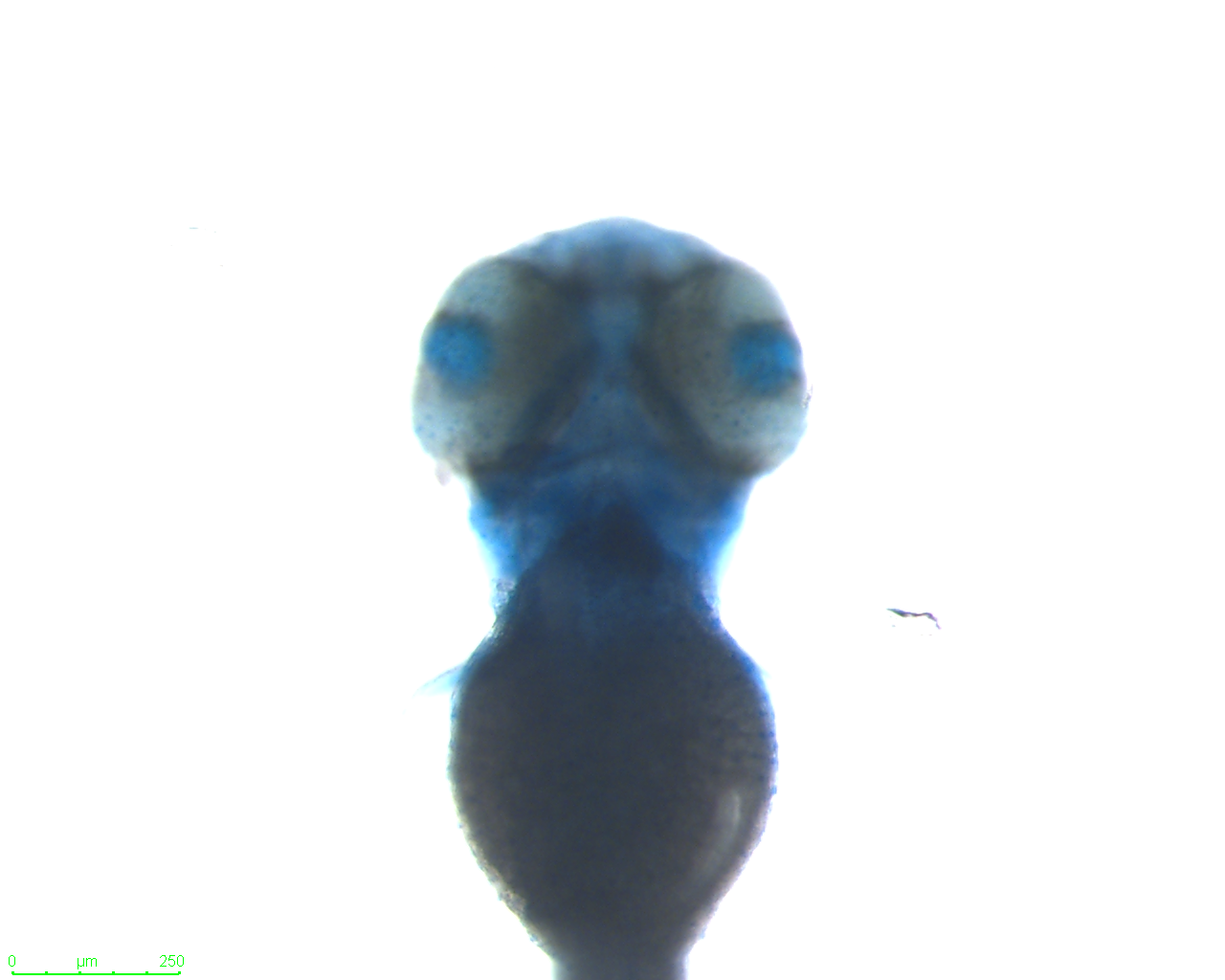

Supplement: Supplementary file 6 — Source Data [file 41467_2021_21053_MOESM6_ESM.zip › Source Data/Zebrafish Morpholino work/Third replicate/EIF5A images 090219_EIF5a_UNT_02.tif]

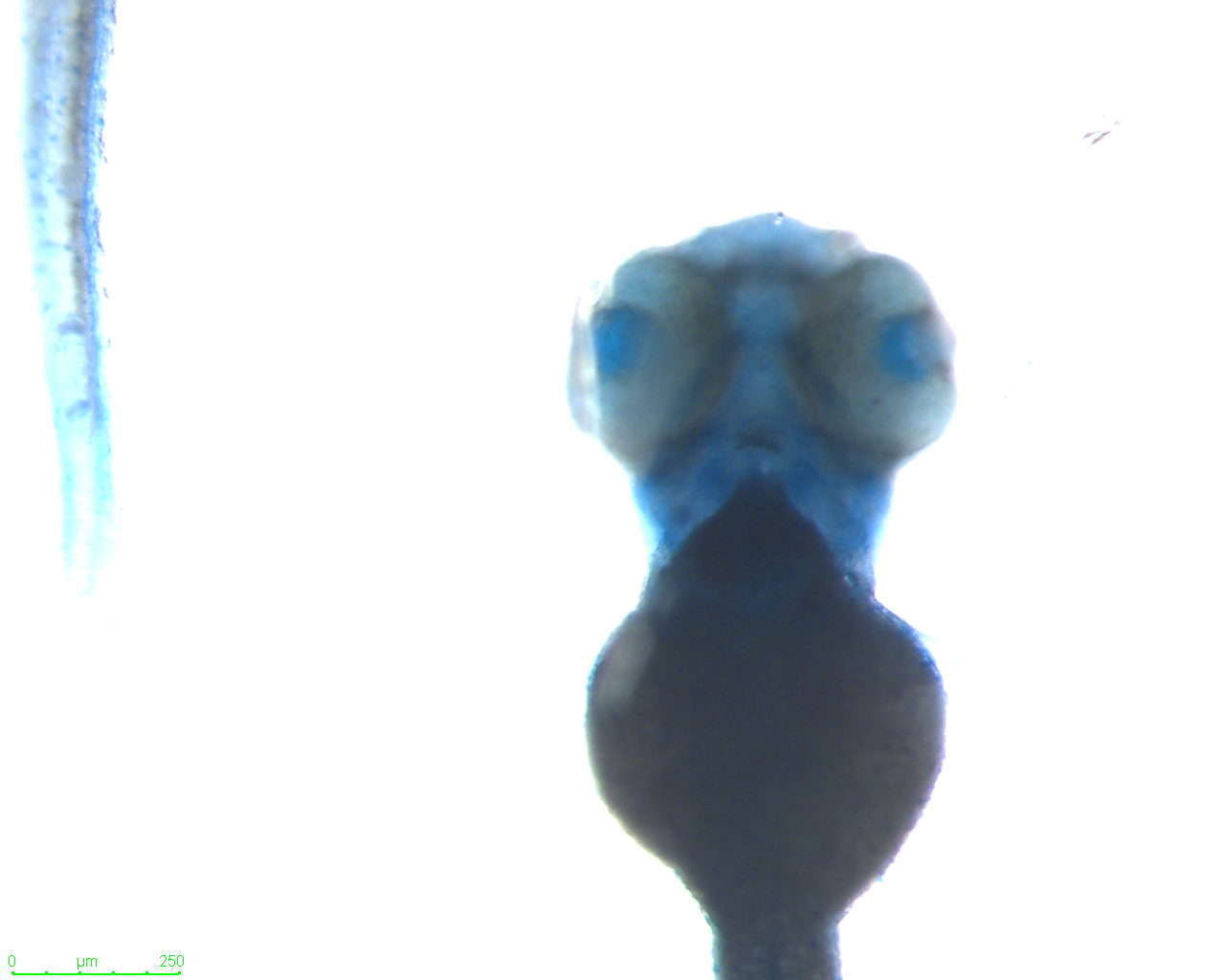

Supplement: Supplementary file 6 — Source Data [file 41467_2021_21053_MOESM6_ESM.zip › Source Data/Zebrafish Morpholino work/Third replicate/EIF5A images 090219_EIF5a_UNT_03.tif]

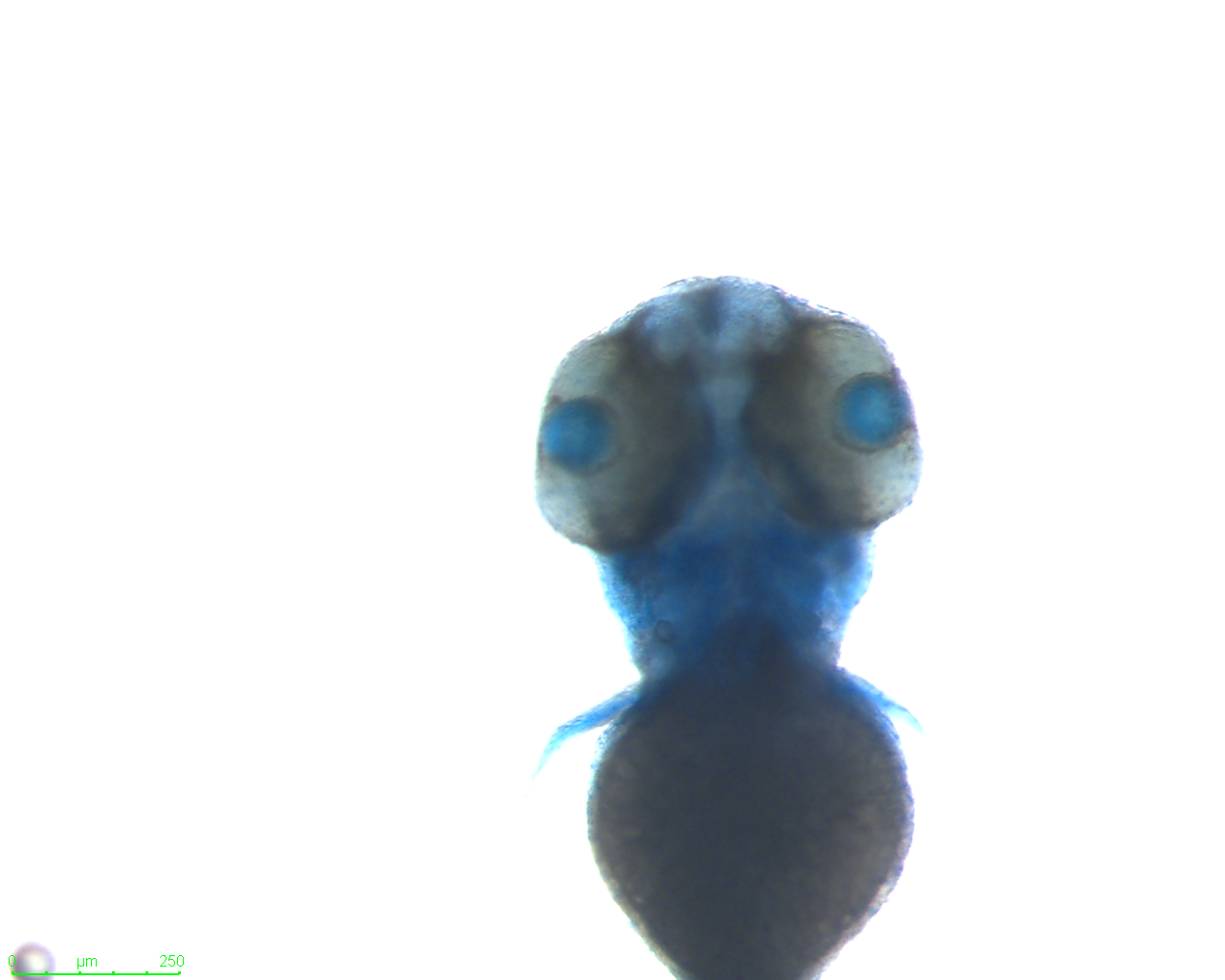

Supplement: Supplementary file 6 — Source Data [file 41467_2021_21053_MOESM6_ESM.zip › Source Data/Zebrafish Morpholino work/Third replicate/EIF5A images 090219_EIF5a_UNT_04.tif]

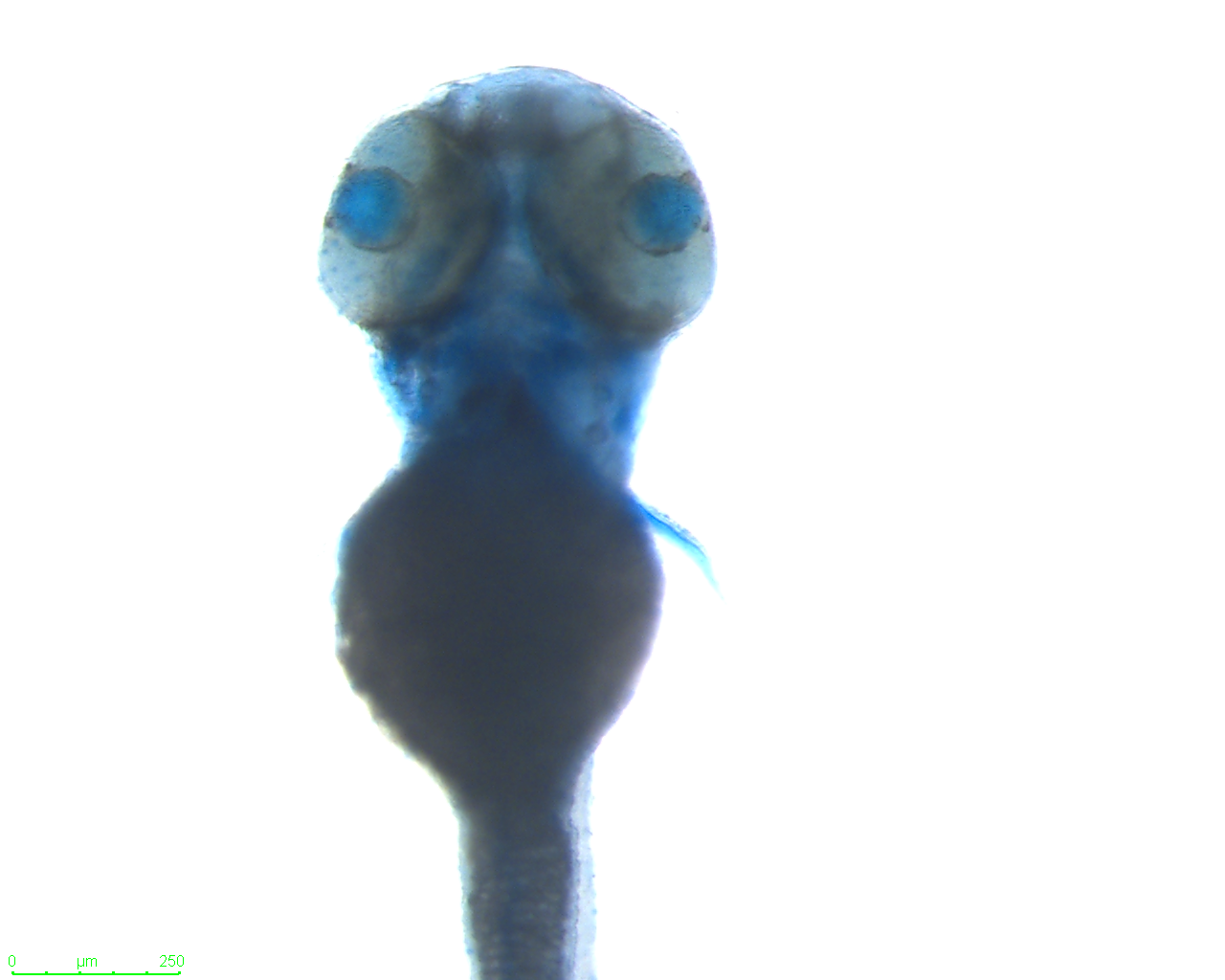

Supplement: Supplementary file 6 — Source Data [file 41467_2021_21053_MOESM6_ESM.zip › Source Data/Zebrafish Morpholino work/Third replicate/EIF5A images 090219_EIF5a_UNT_05.tif]

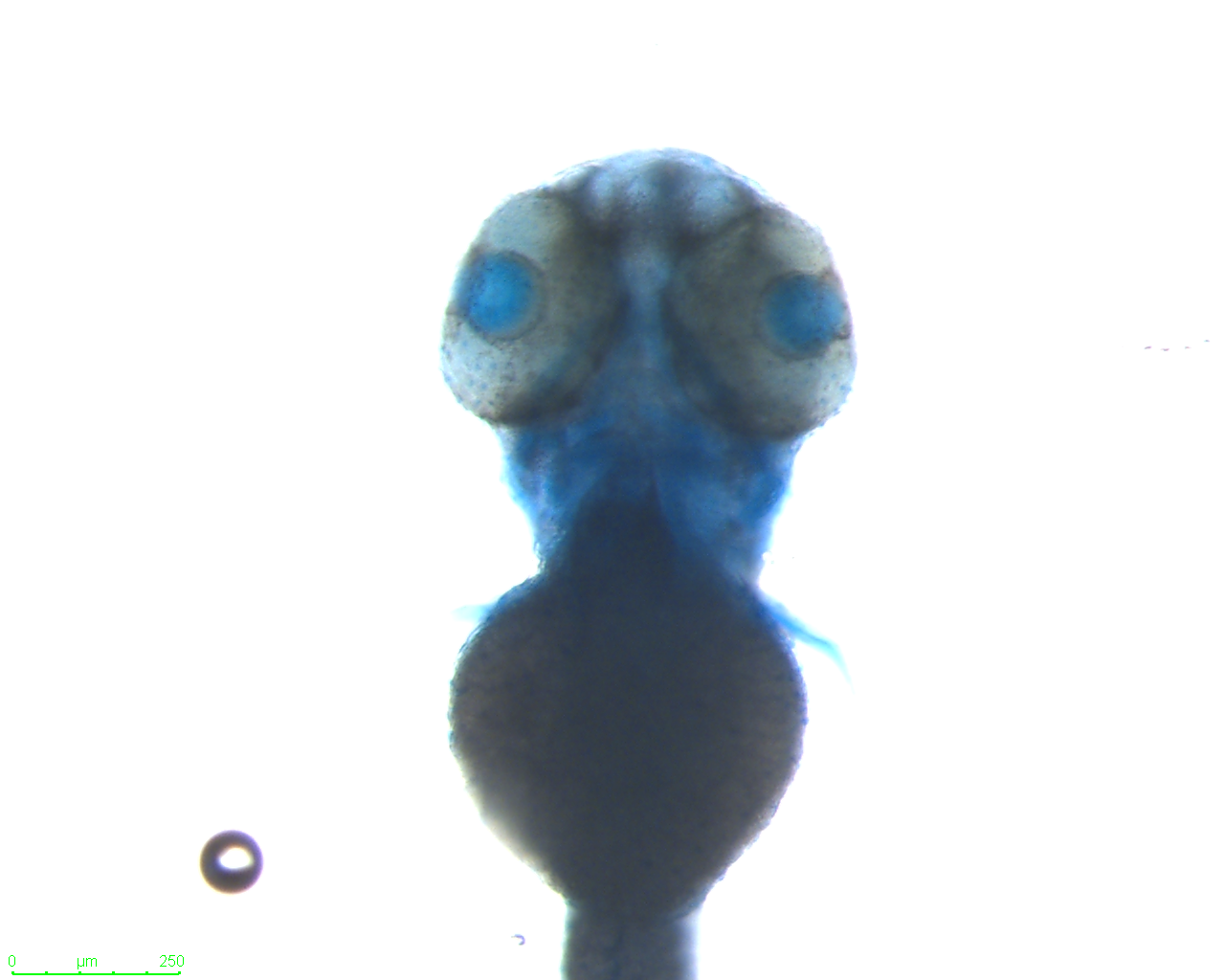

Supplement: Supplementary file 6 — Source Data [file 41467_2021_21053_MOESM6_ESM.zip › Source Data/Zebrafish Morpholino work/Third replicate/EIF5A images 090219_EIF5a_UNT_06.tif]

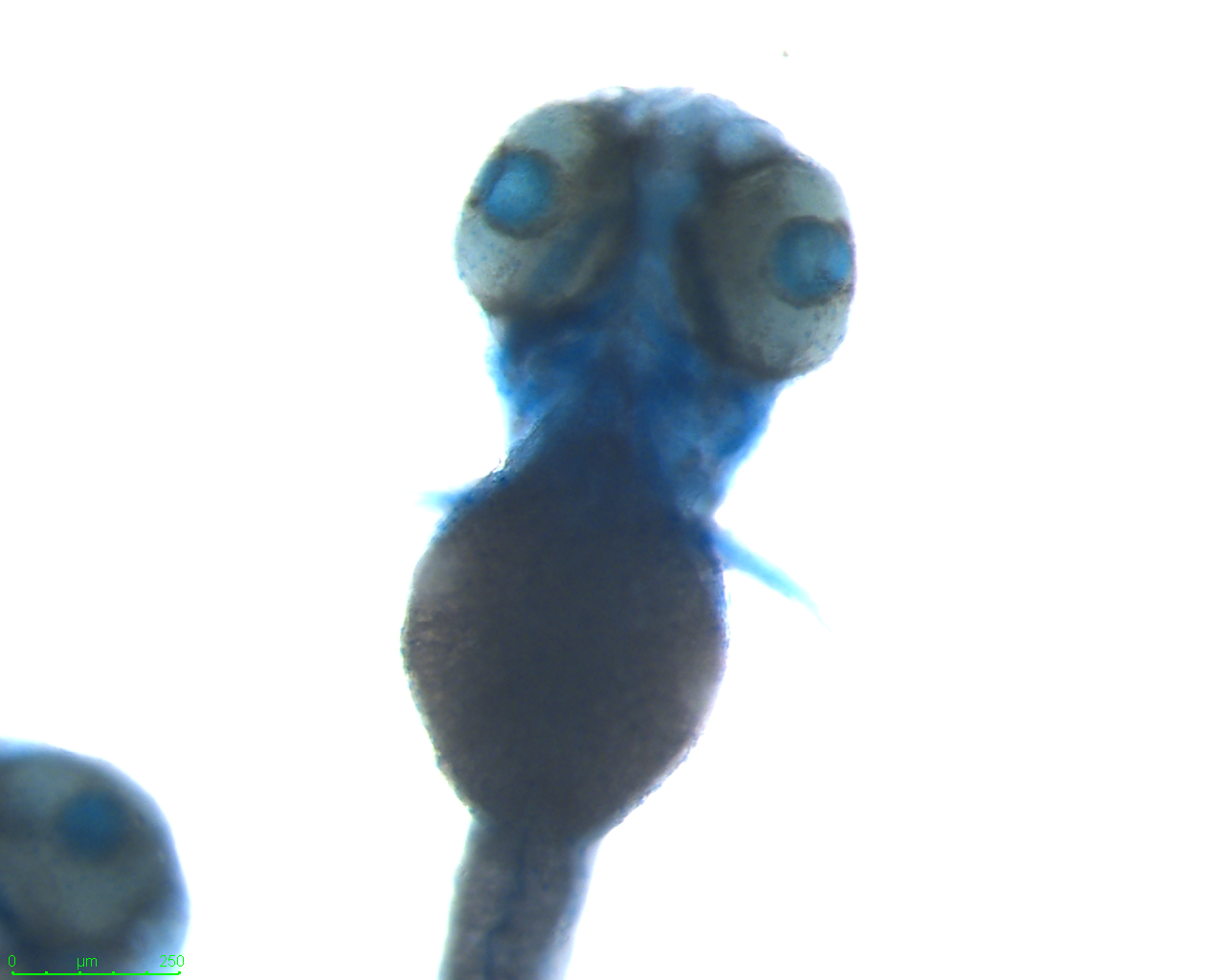

Supplement: Supplementary file 6 — Source Data [file 41467_2021_21053_MOESM6_ESM.zip › Source Data/Zebrafish Morpholino work/Third replicate/EIF5A images 090219_EIF5a_UNT_07.tif]

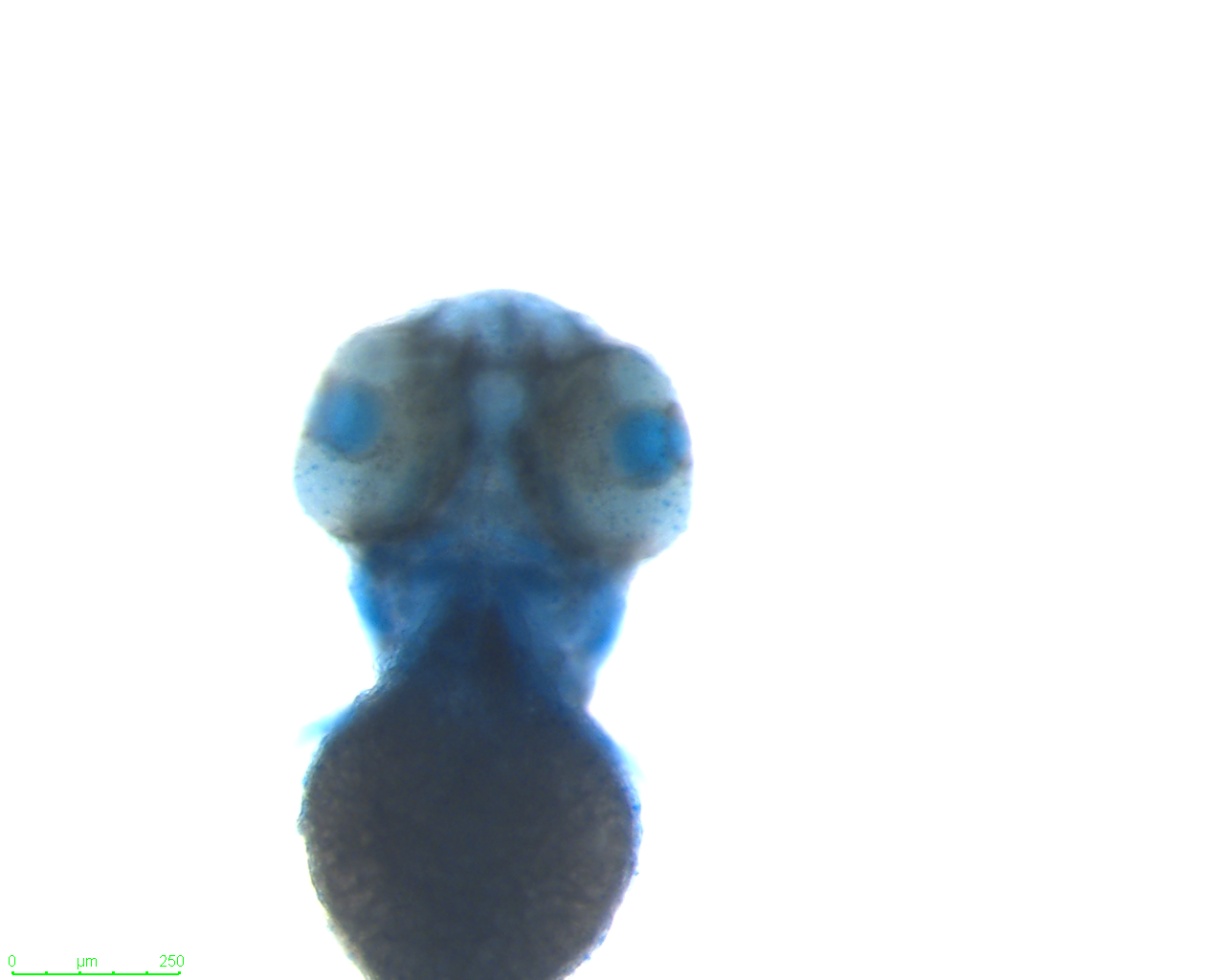

Supplement: Supplementary file 6 — Source Data [file 41467_2021_21053_MOESM6_ESM.zip › Source Data/Zebrafish Morpholino work/Third replicate/EIF5A images 090219_EIF5a_UNT_08.tif]

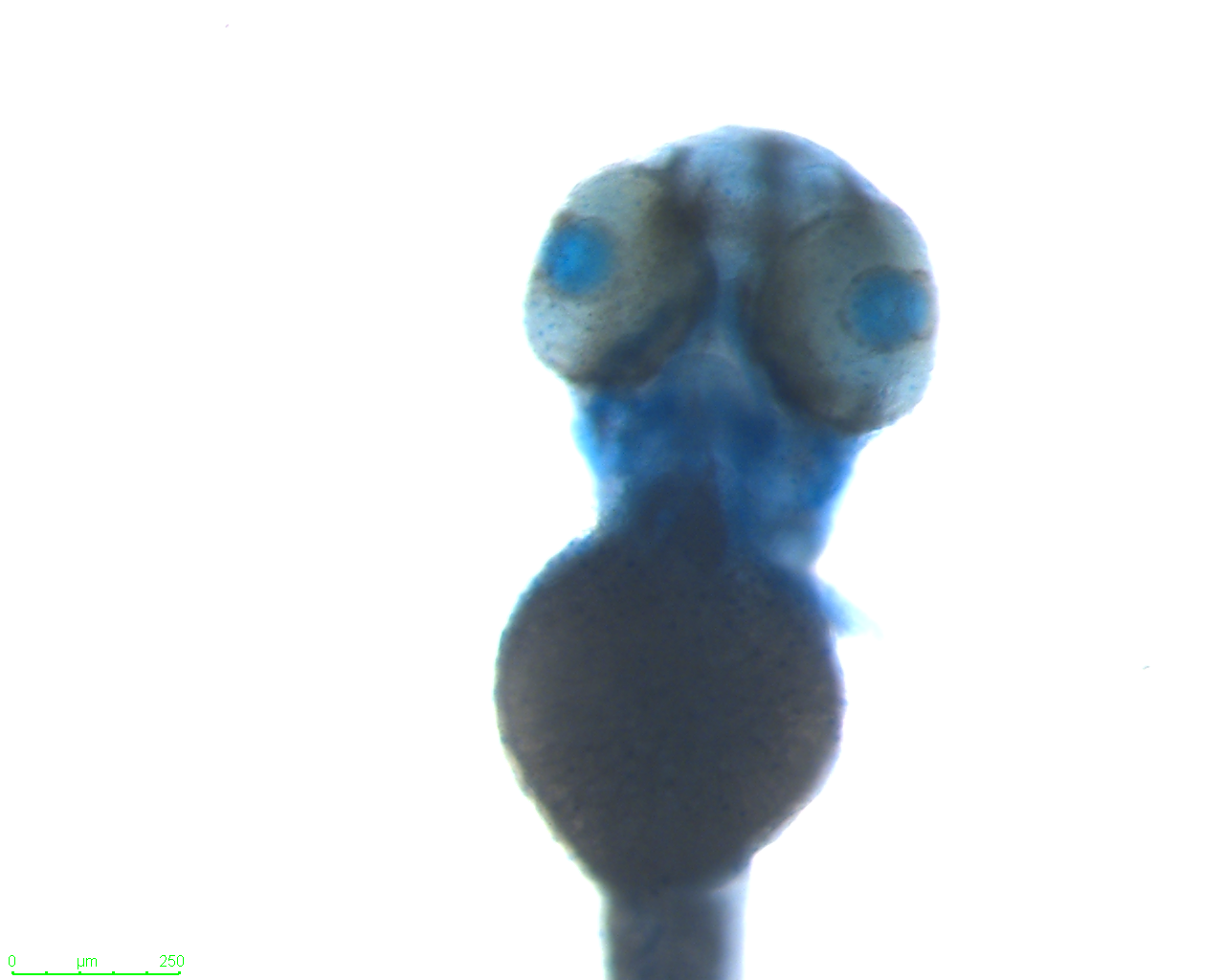

Supplement: Supplementary file 6 — Source Data [file 41467_2021_21053_MOESM6_ESM.zip › Source Data/Zebrafish Morpholino work/Third replicate/EIF5A images 090219_EIF5a_UNT_09.tif]

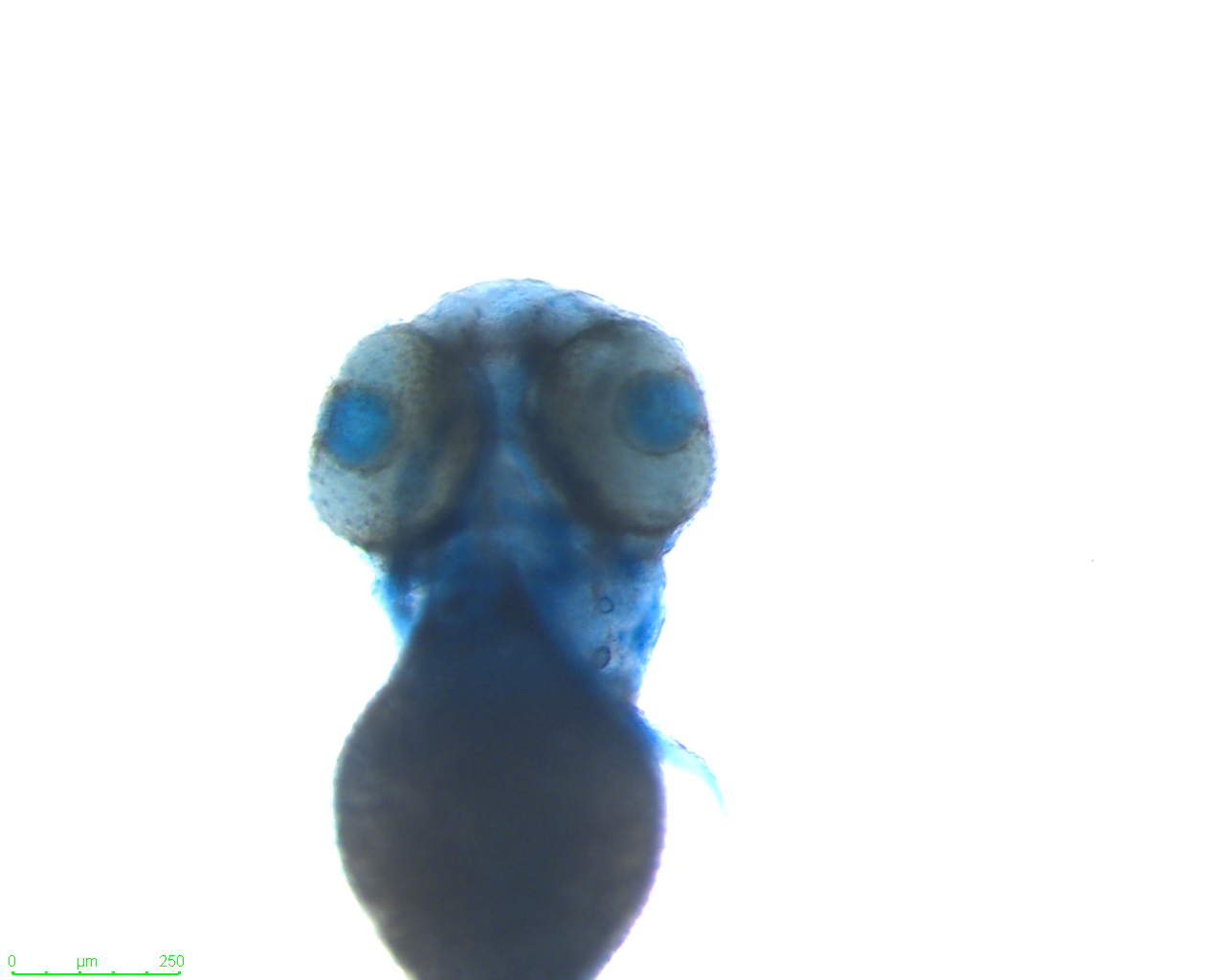

Supplement: Supplementary file 6 — Source Data [file 41467_2021_21053_MOESM6_ESM.zip › Source Data/Zebrafish Morpholino work/Third replicate/EIF5A images 090219_EIF5a_UNT_10.tif]

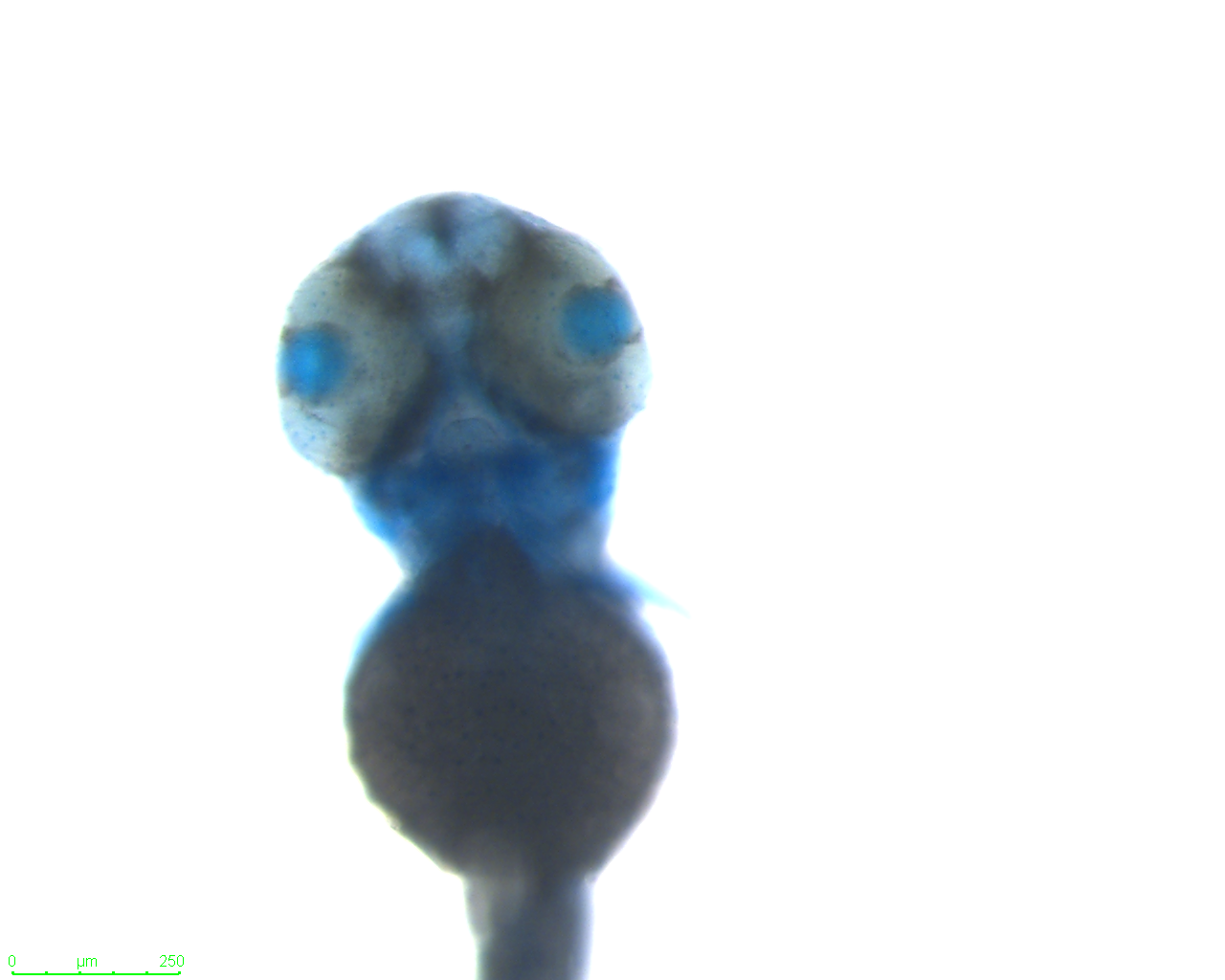

Supplement: Supplementary file 6 — Source Data [file 41467_2021_21053_MOESM6_ESM.zip › Source Data/Zebrafish Morpholino work/Third replicate/EIF5A images 090219_EIF5a_UNT_11.tif]

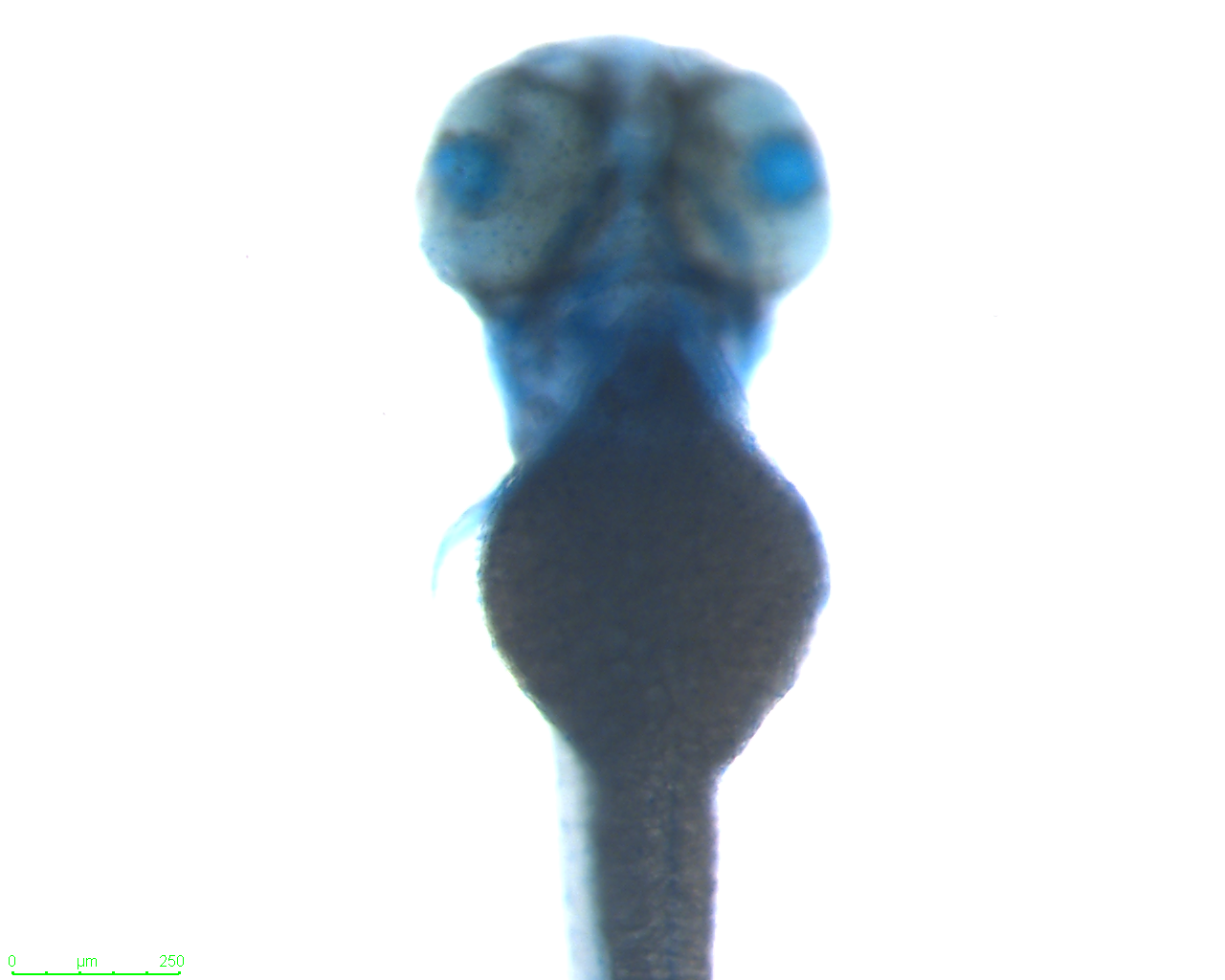

Supplement: Supplementary file 6 — Source Data [file 41467_2021_21053_MOESM6_ESM.zip › Source Data/Zebrafish Morpholino work/Third replicate/EIF5A images 090219_EIF5a_UNT_12.tif]

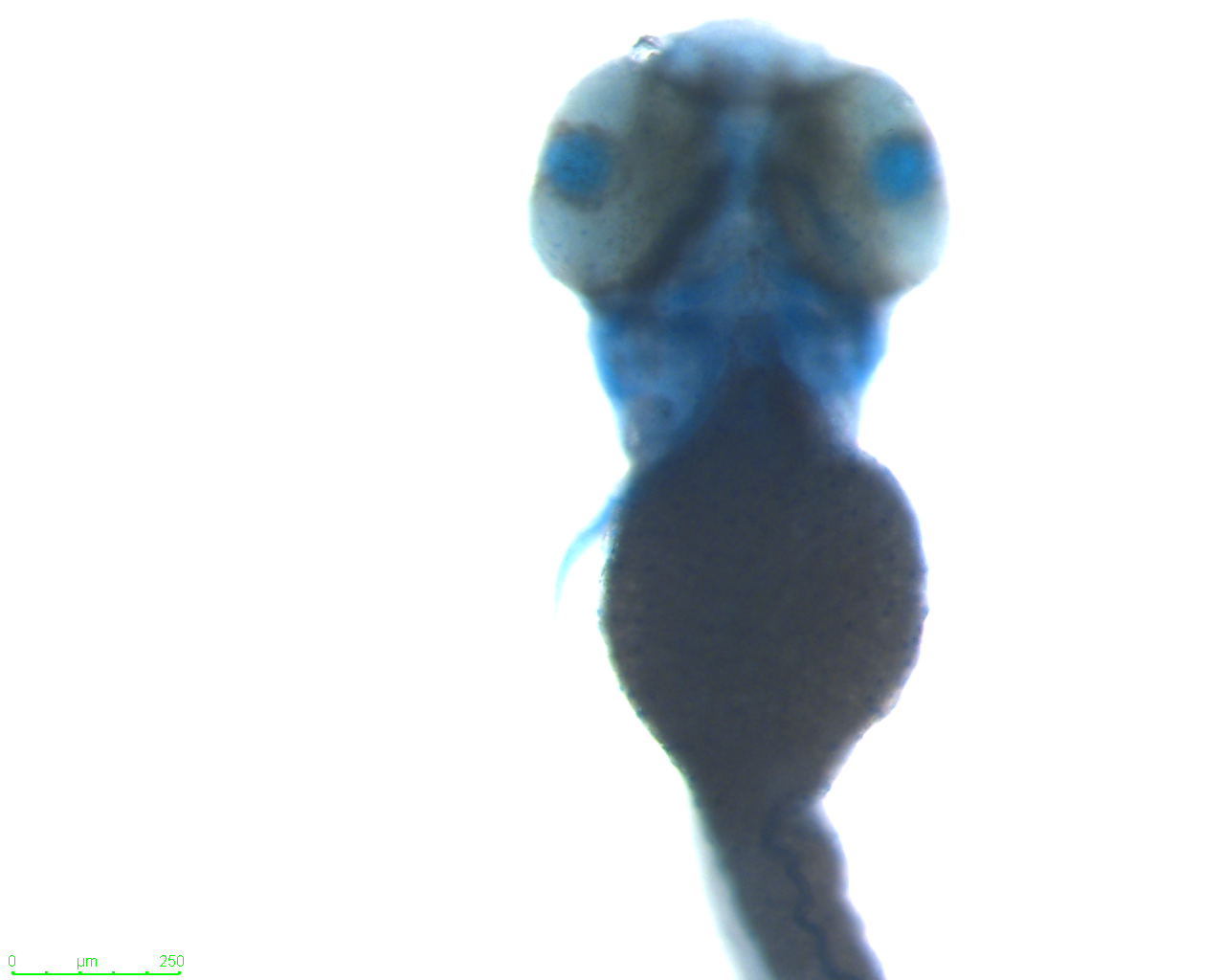

Supplement: Supplementary file 6 — Source Data [file 41467_2021_21053_MOESM6_ESM.zip › Source Data/Zebrafish Morpholino work/Third replicate/EIF5A images 090219_EIF5a_UNT_13.tif]

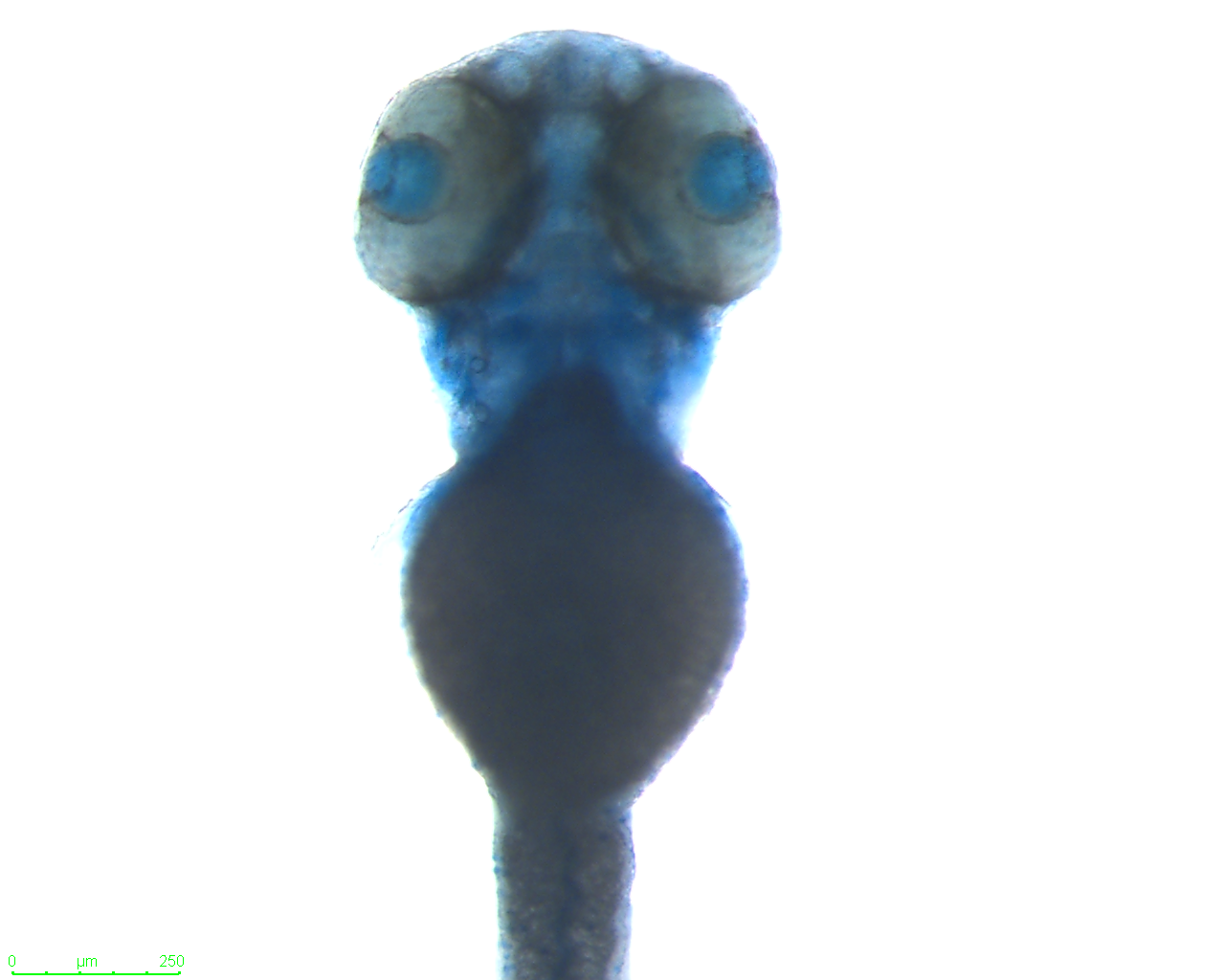

Supplement: Supplementary file 6 — Source Data [file 41467_2021_21053_MOESM6_ESM.zip › Source Data/Zebrafish Morpholino work/Third replicate/EIF5A images 090219_EIF5a_UNT_14.tif]

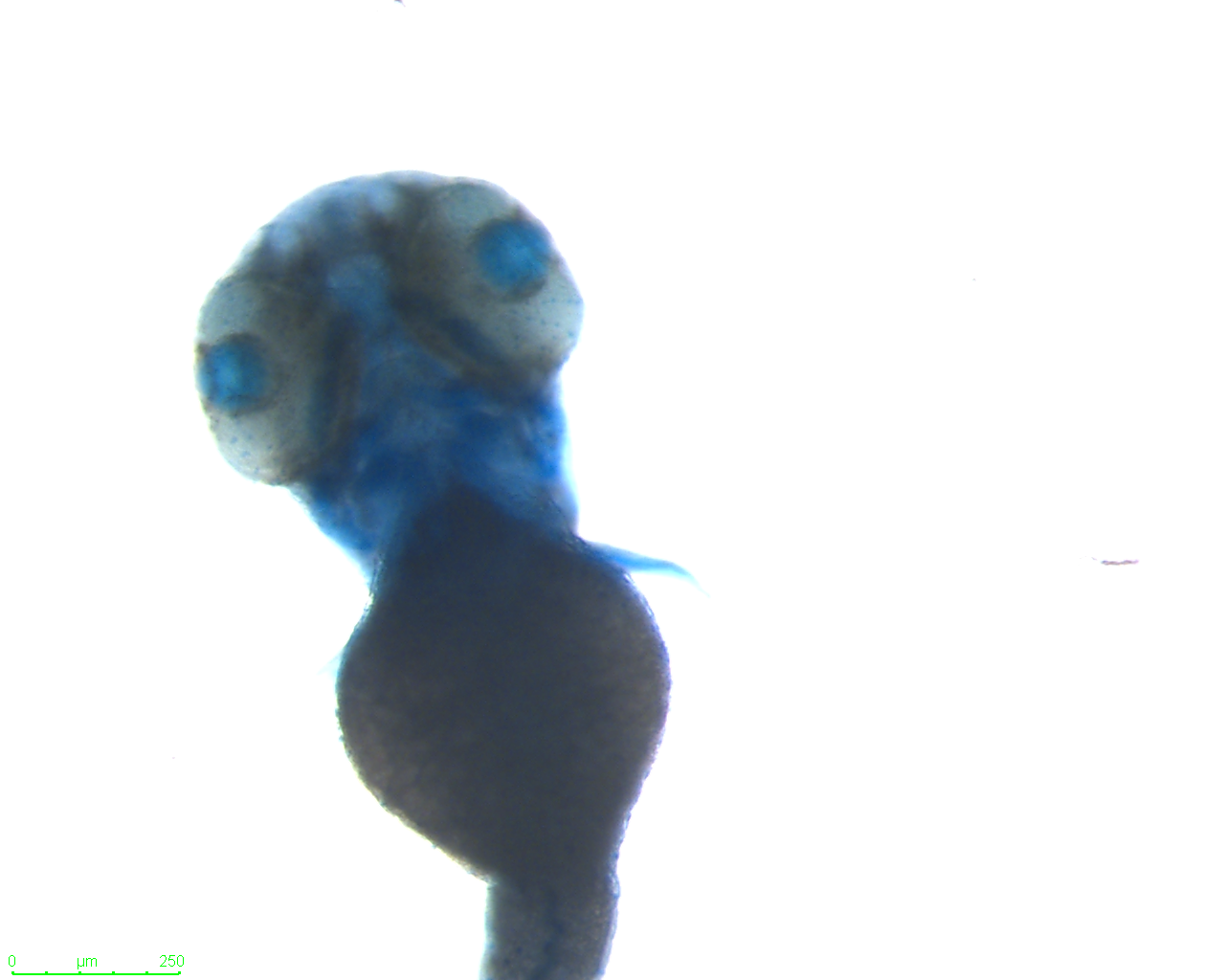

Supplement: Supplementary file 6 — Source Data [file 41467_2021_21053_MOESM6_ESM.zip › Source Data/Zebrafish Morpholino work/Third replicate/EIF5A images 090219_EIF5a_UNT_15.tif]
