# Supplementary figures and images for: Impaired eIF5A function causes a Mendelian disorder that is partially rescued in model systems by spermidine (part 1 of 2)
Source: Nat Commun. 2021 Feb 5;12:833. doi: 10.1038/s41467-021-21053-2 (PMC7864902; doi:10.1038/s41467-021-21053-2)

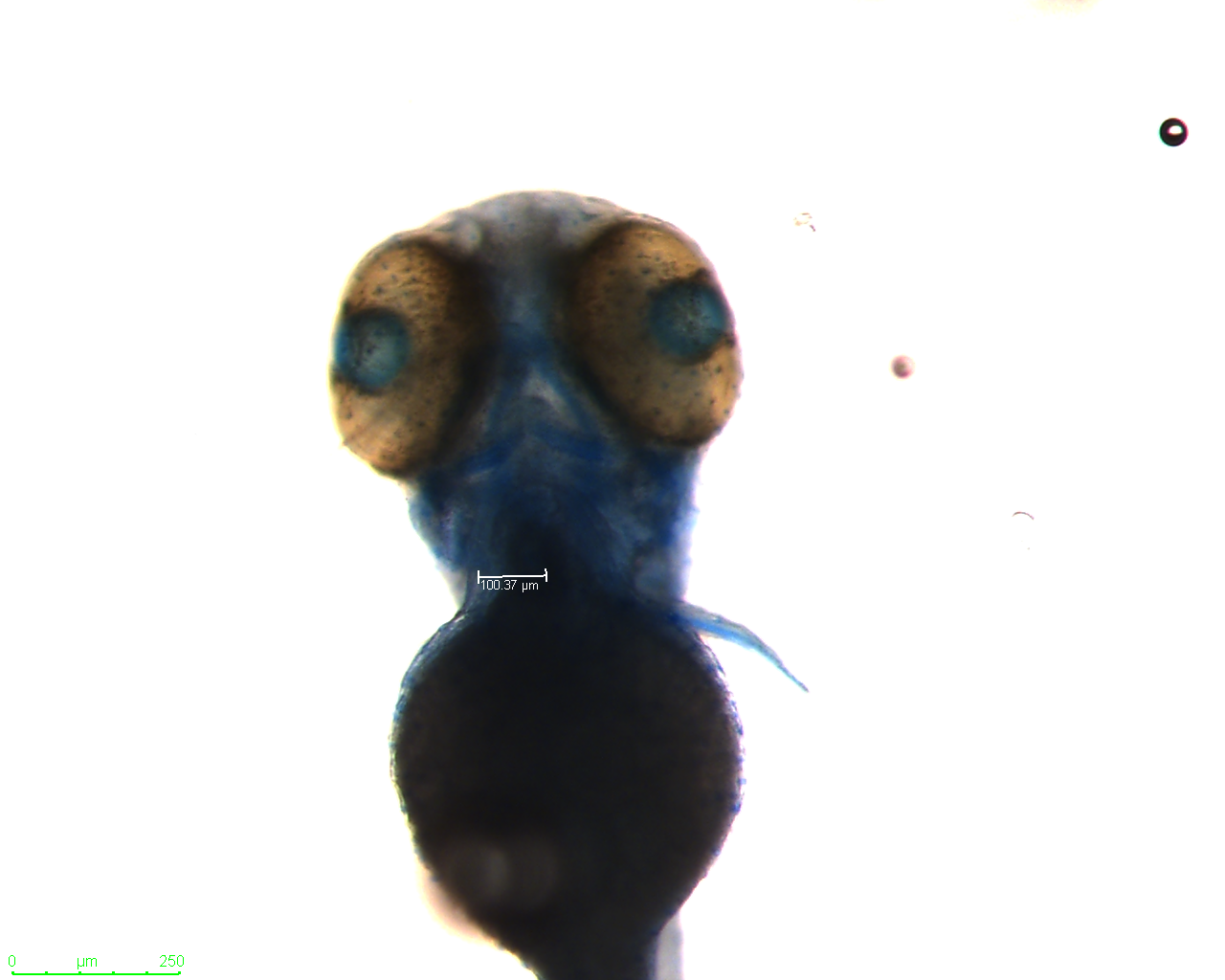

Supplement: Supplementary file 6 — Source Data [file 41467_2021_21053_MOESM6_ESM.zip › Source Data/Zebrafish Morpholino work/First replicate/EIF5A images_Control_Spermadine_01.tif]

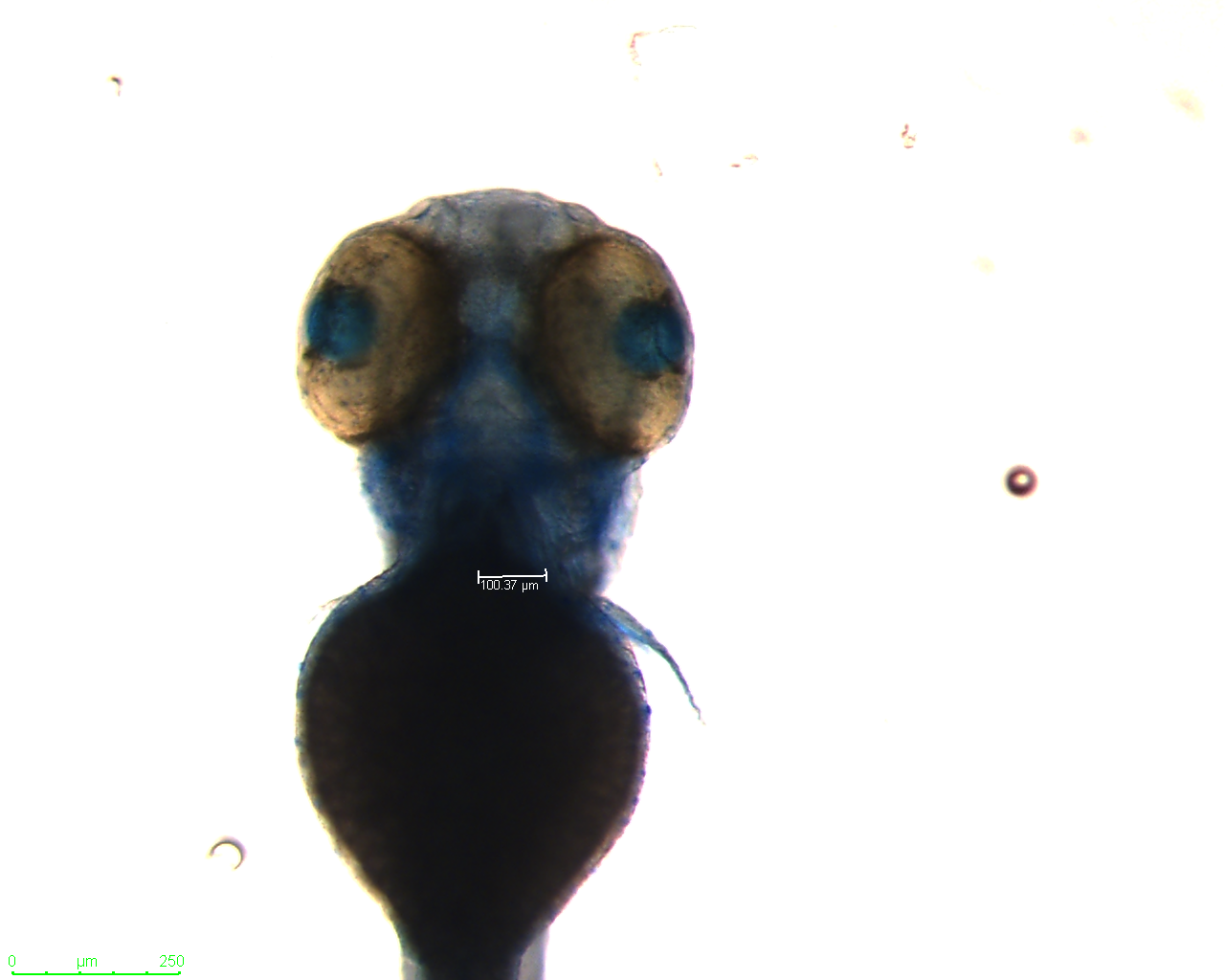

Supplement: Supplementary file 6 — Source Data [file 41467_2021_21053_MOESM6_ESM.zip › Source Data/Zebrafish Morpholino work/First replicate/EIF5A images_Control_Spermadine_02.tif]

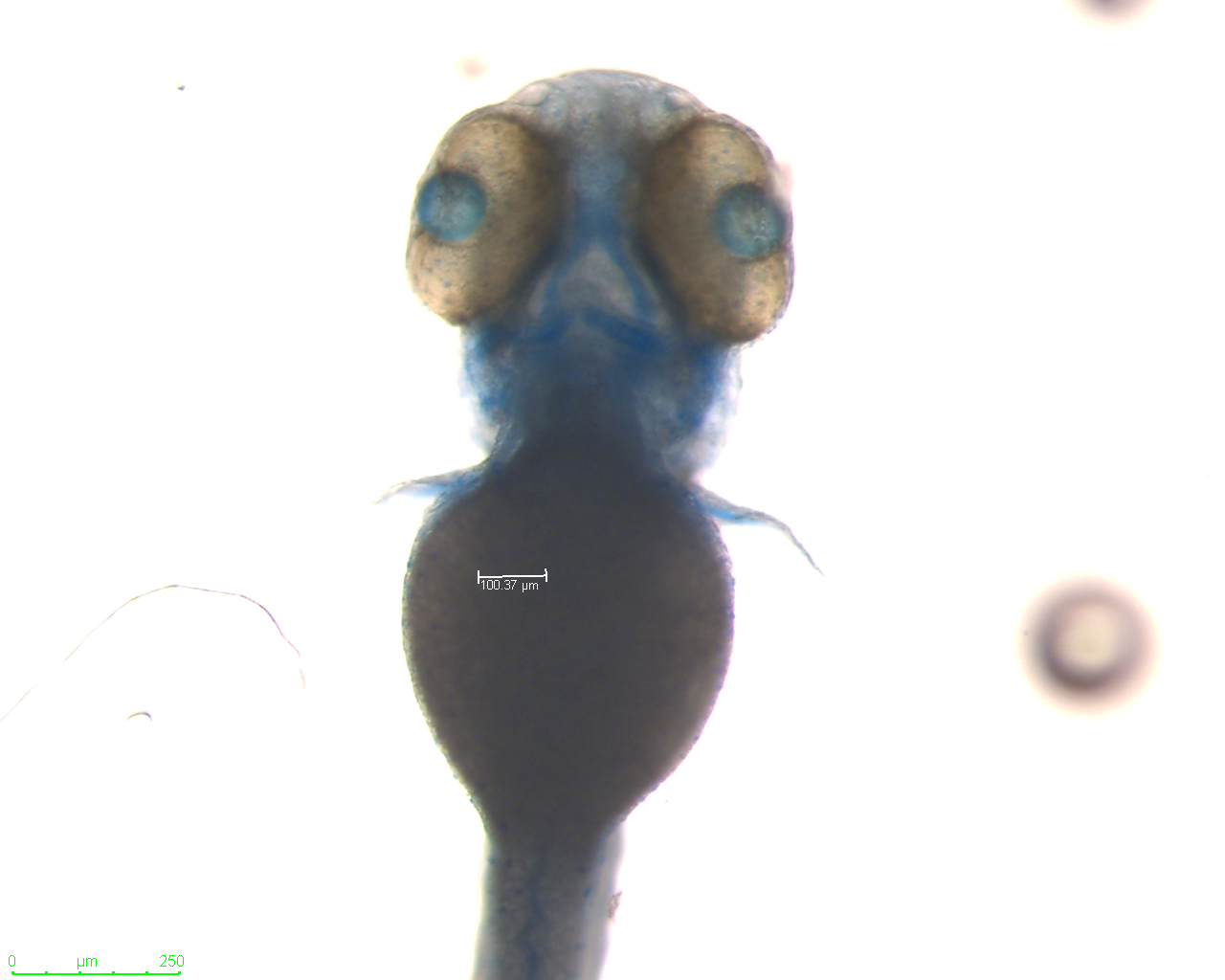

Supplement: Supplementary file 6 — Source Data [file 41467_2021_21053_MOESM6_ESM.zip › Source Data/Zebrafish Morpholino work/First replicate/EIF5A images_Control_Spermadine_03.tif]

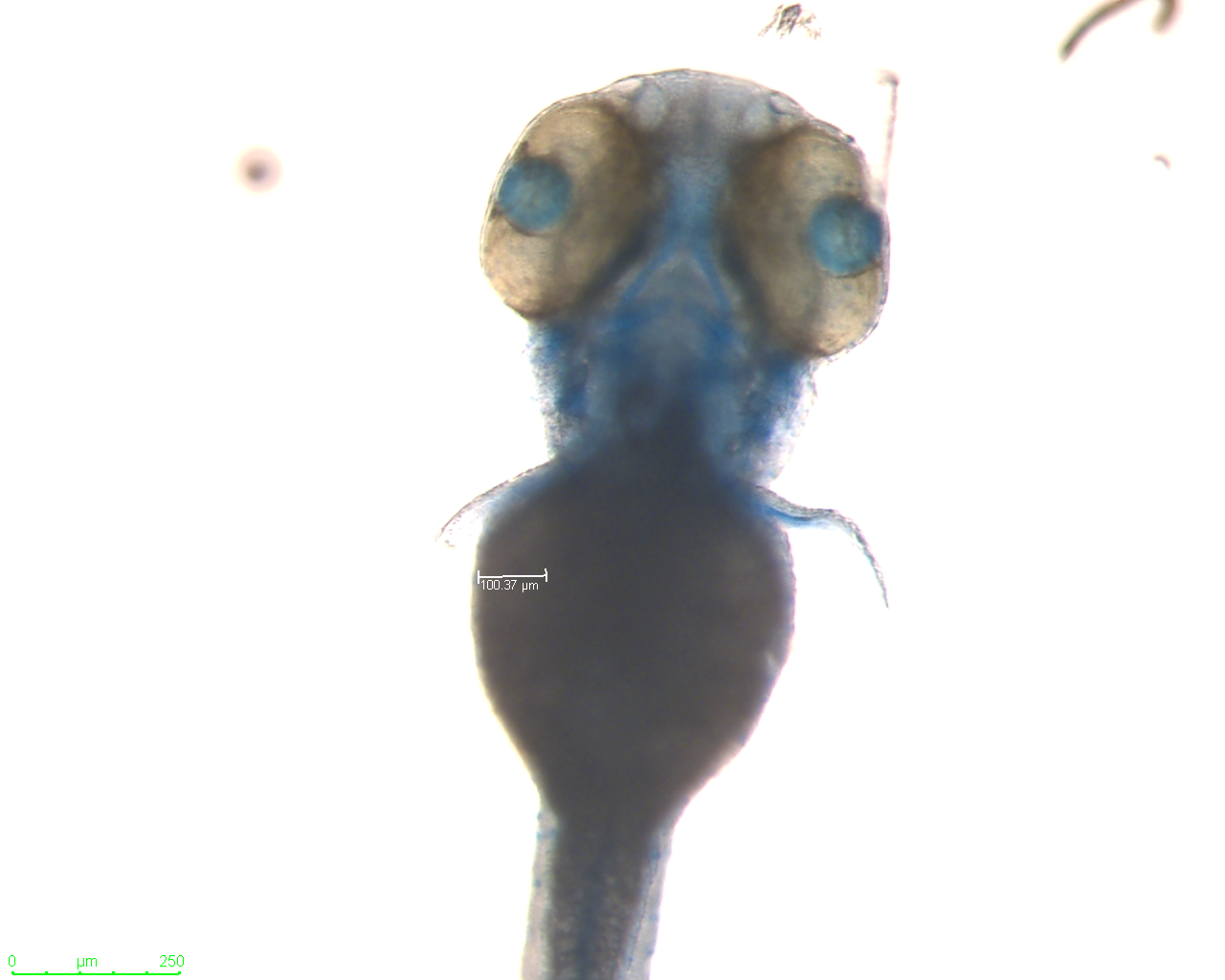

Supplement: Supplementary file 6 — Source Data [file 41467_2021_21053_MOESM6_ESM.zip › Source Data/Zebrafish Morpholino work/First replicate/EIF5A images_Control_Spermadine_04.tif]

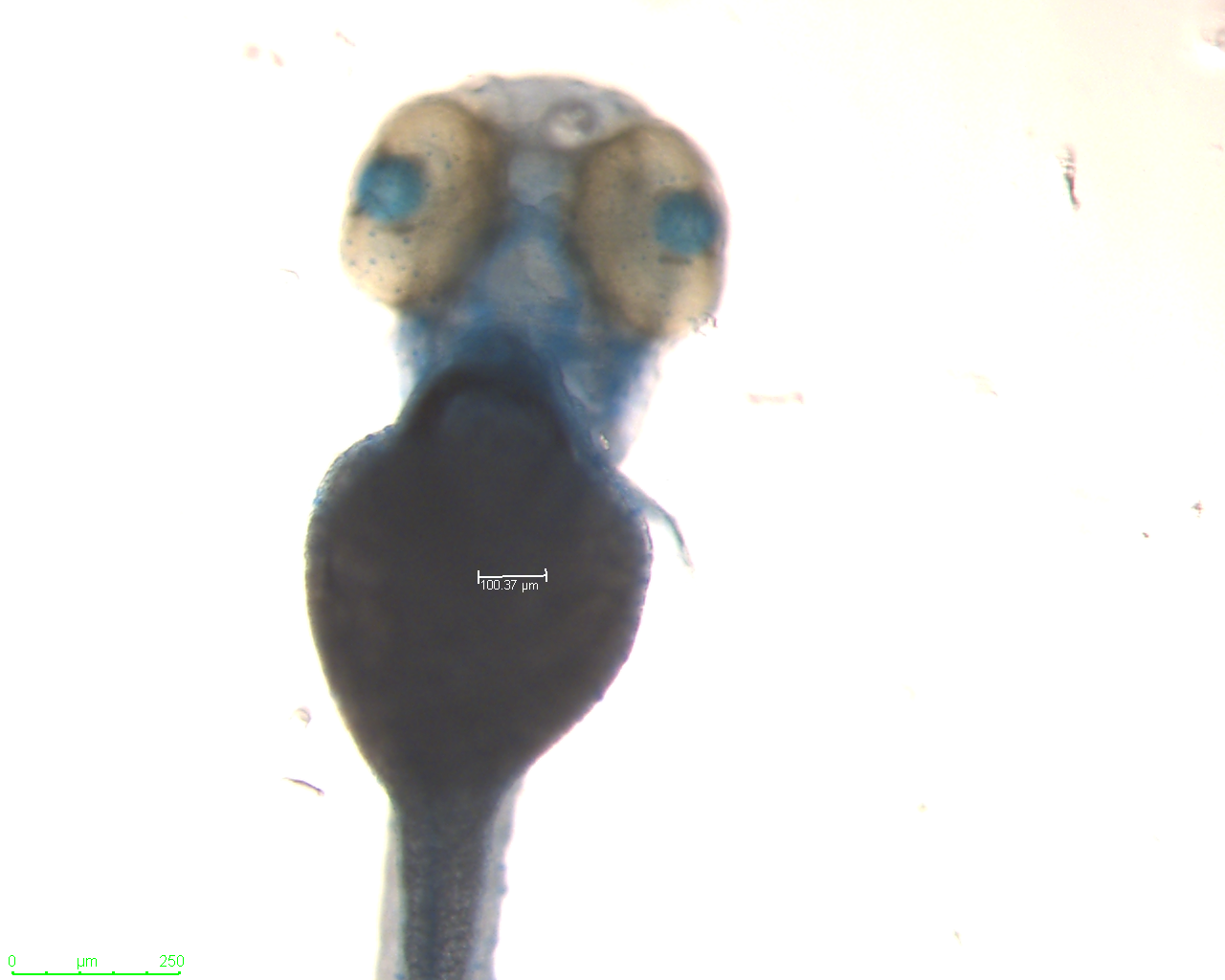

Supplement: Supplementary file 6 — Source Data [file 41467_2021_21053_MOESM6_ESM.zip › Source Data/Zebrafish Morpholino work/First replicate/EIF5A images_Control_Spermadine_05.tif]

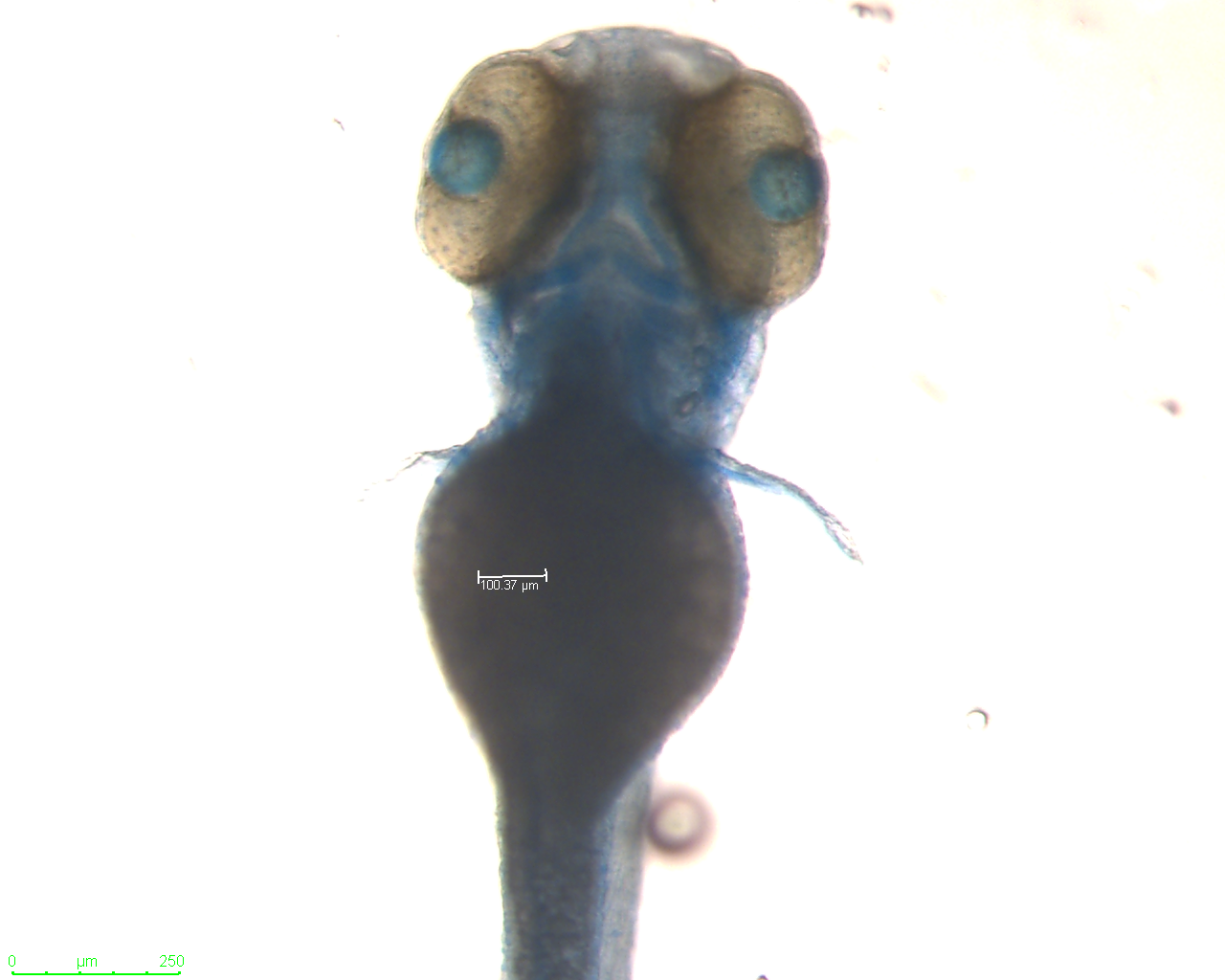

Supplement: Supplementary file 6 — Source Data [file 41467_2021_21053_MOESM6_ESM.zip › Source Data/Zebrafish Morpholino work/First replicate/EIF5A images_Control_Spermadine_06.tif]

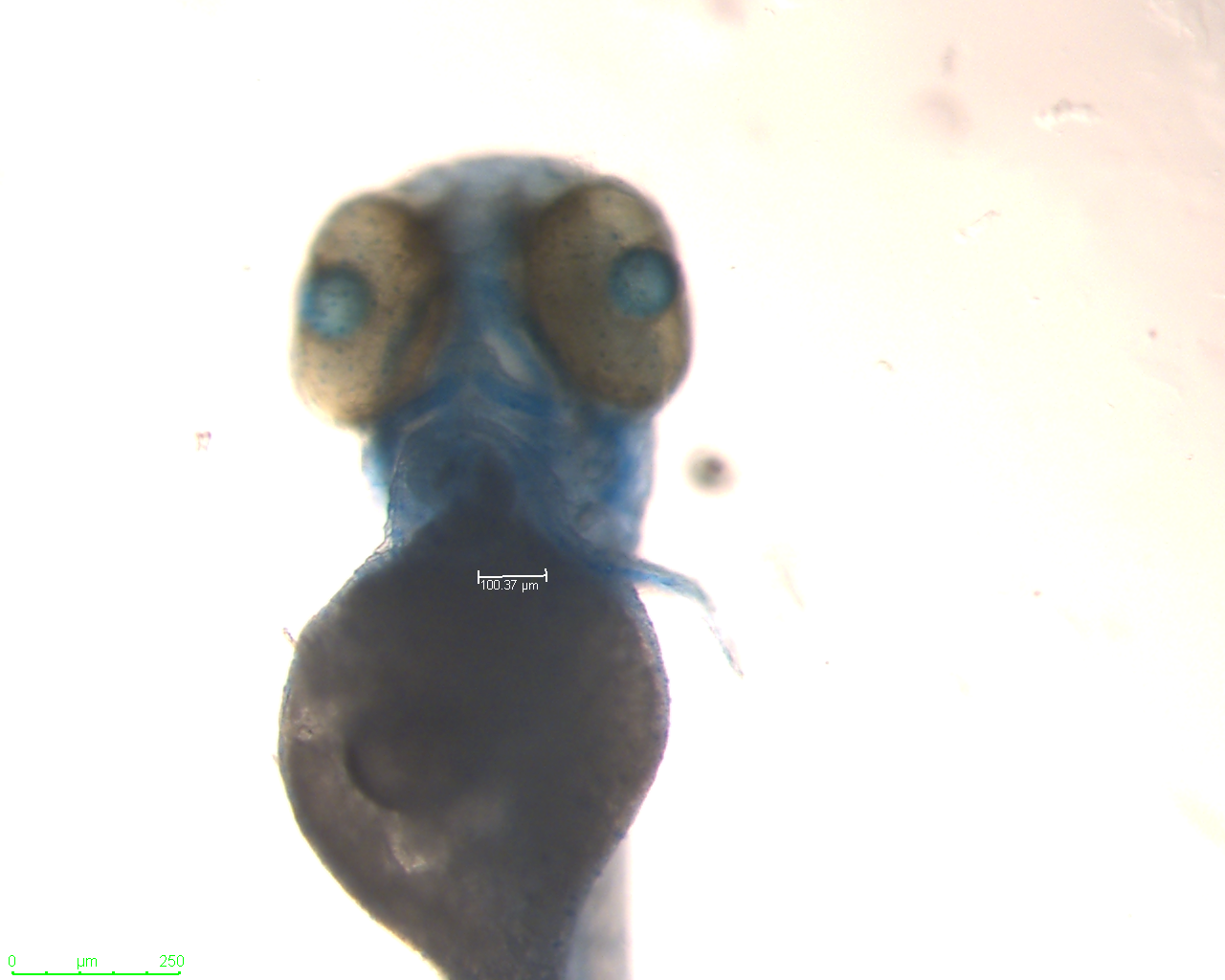

Supplement: Supplementary file 6 — Source Data [file 41467_2021_21053_MOESM6_ESM.zip › Source Data/Zebrafish Morpholino work/First replicate/EIF5A images_Control_Spermadine_07.tif]

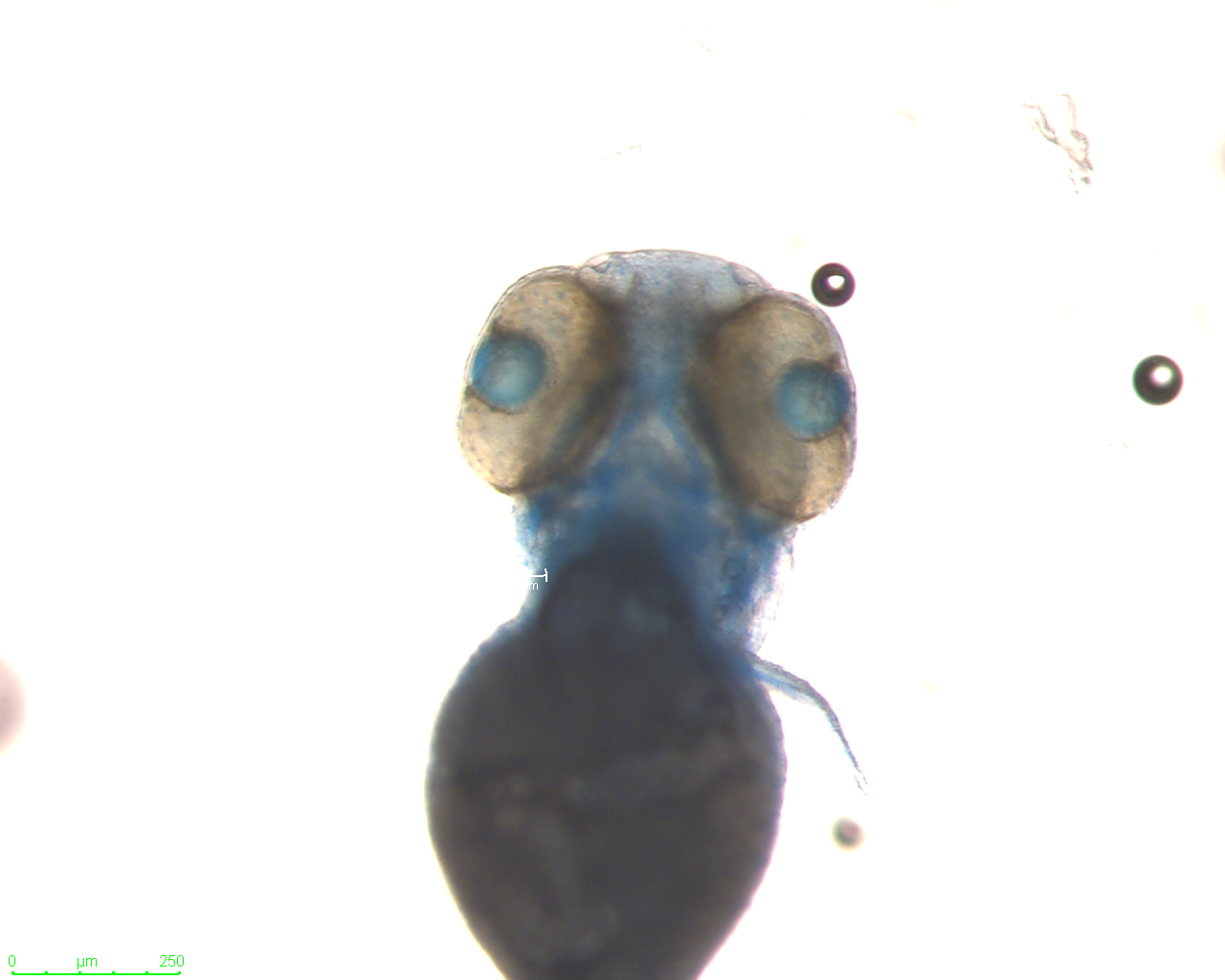

Supplement: Supplementary file 6 — Source Data [file 41467_2021_21053_MOESM6_ESM.zip › Source Data/Zebrafish Morpholino work/First replicate/EIF5A images_Control_Spermadine_08.tif]

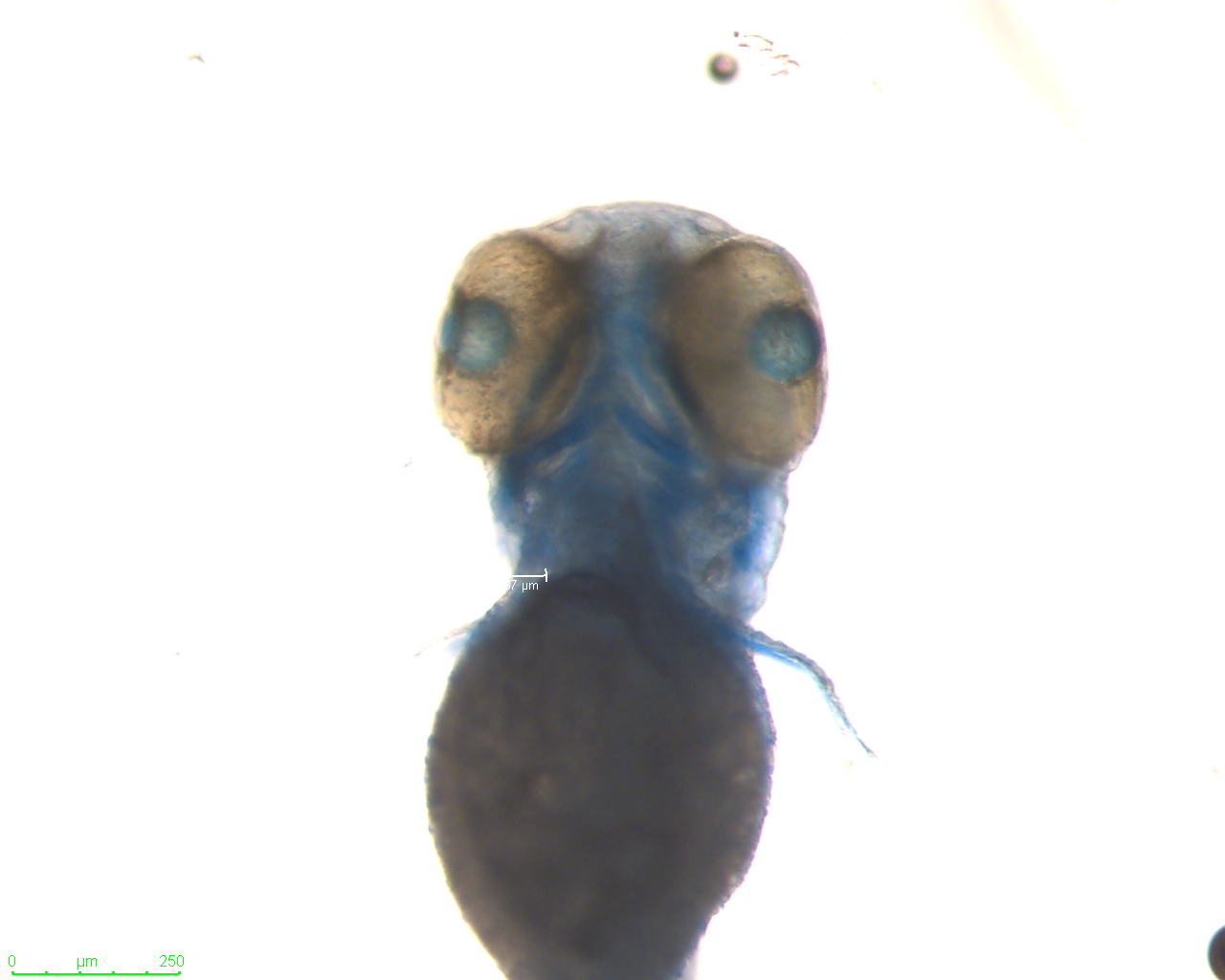

Supplement: Supplementary file 6 — Source Data [file 41467_2021_21053_MOESM6_ESM.zip › Source Data/Zebrafish Morpholino work/First replicate/EIF5A images_Control_untreated_01.tif]

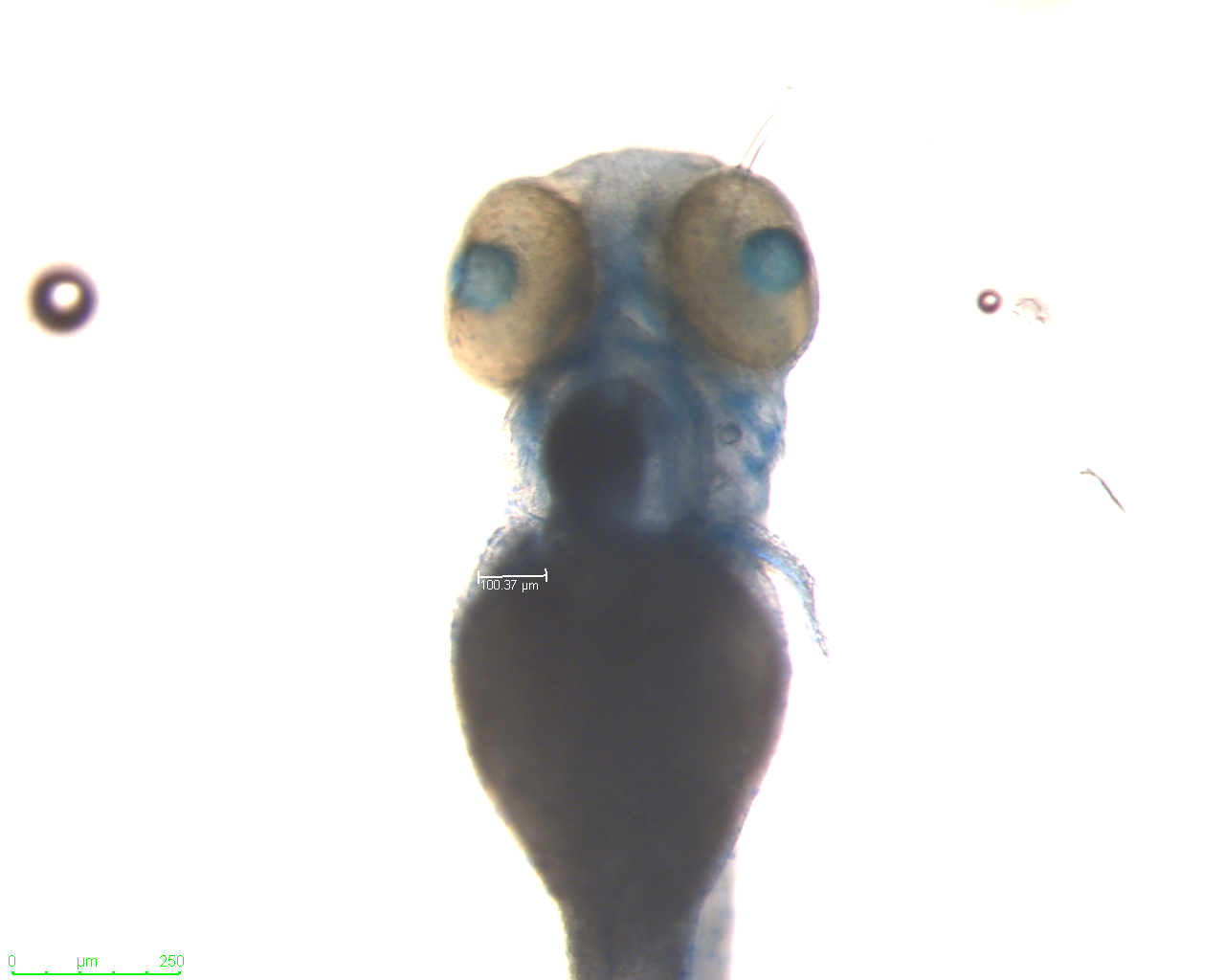

Supplement: Supplementary file 6 — Source Data [file 41467_2021_21053_MOESM6_ESM.zip › Source Data/Zebrafish Morpholino work/First replicate/EIF5A images_Control_untreated_02.tif]

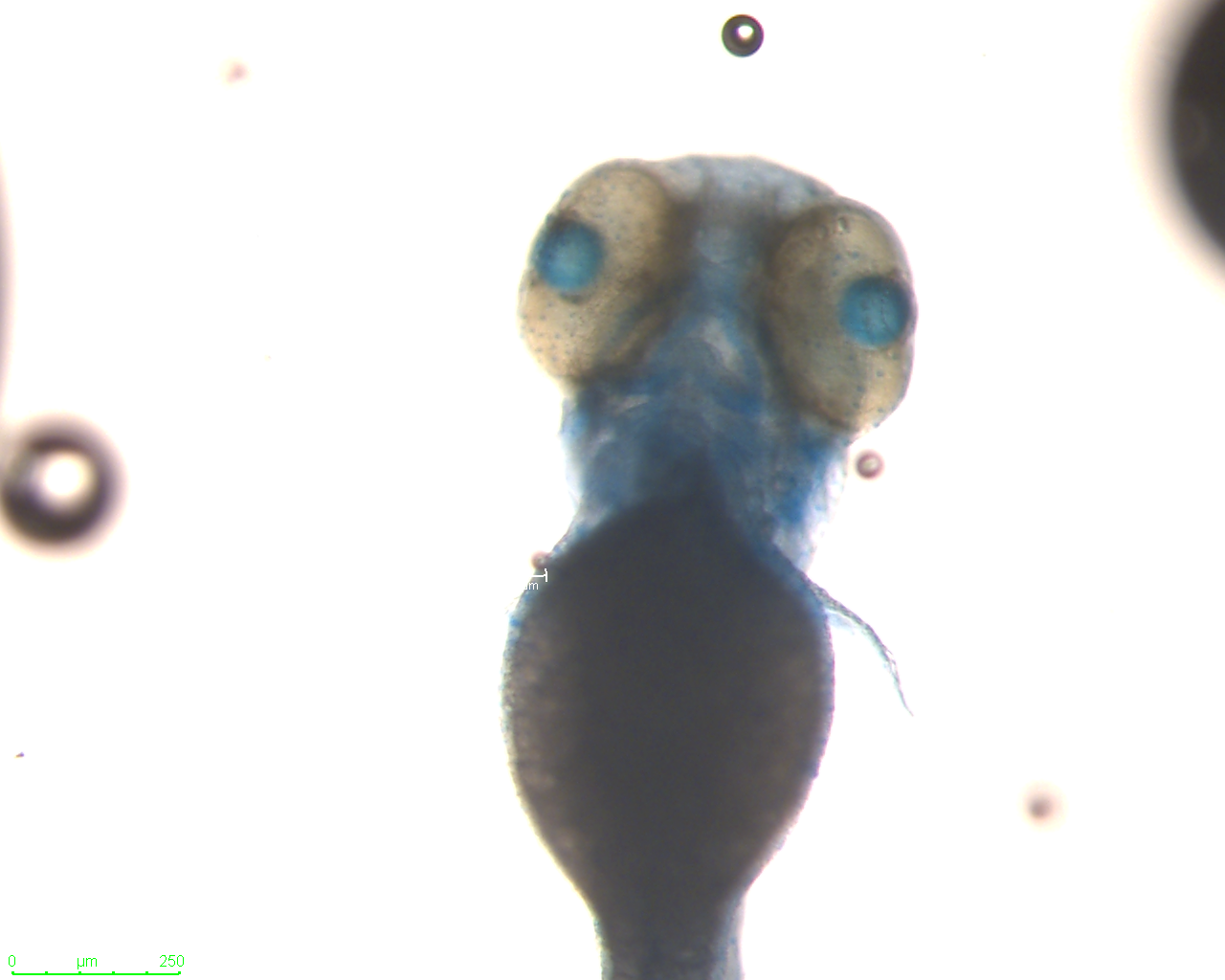

Supplement: Supplementary file 6 — Source Data [file 41467_2021_21053_MOESM6_ESM.zip › Source Data/Zebrafish Morpholino work/First replicate/EIF5A images_Control_untreated_03.tif]

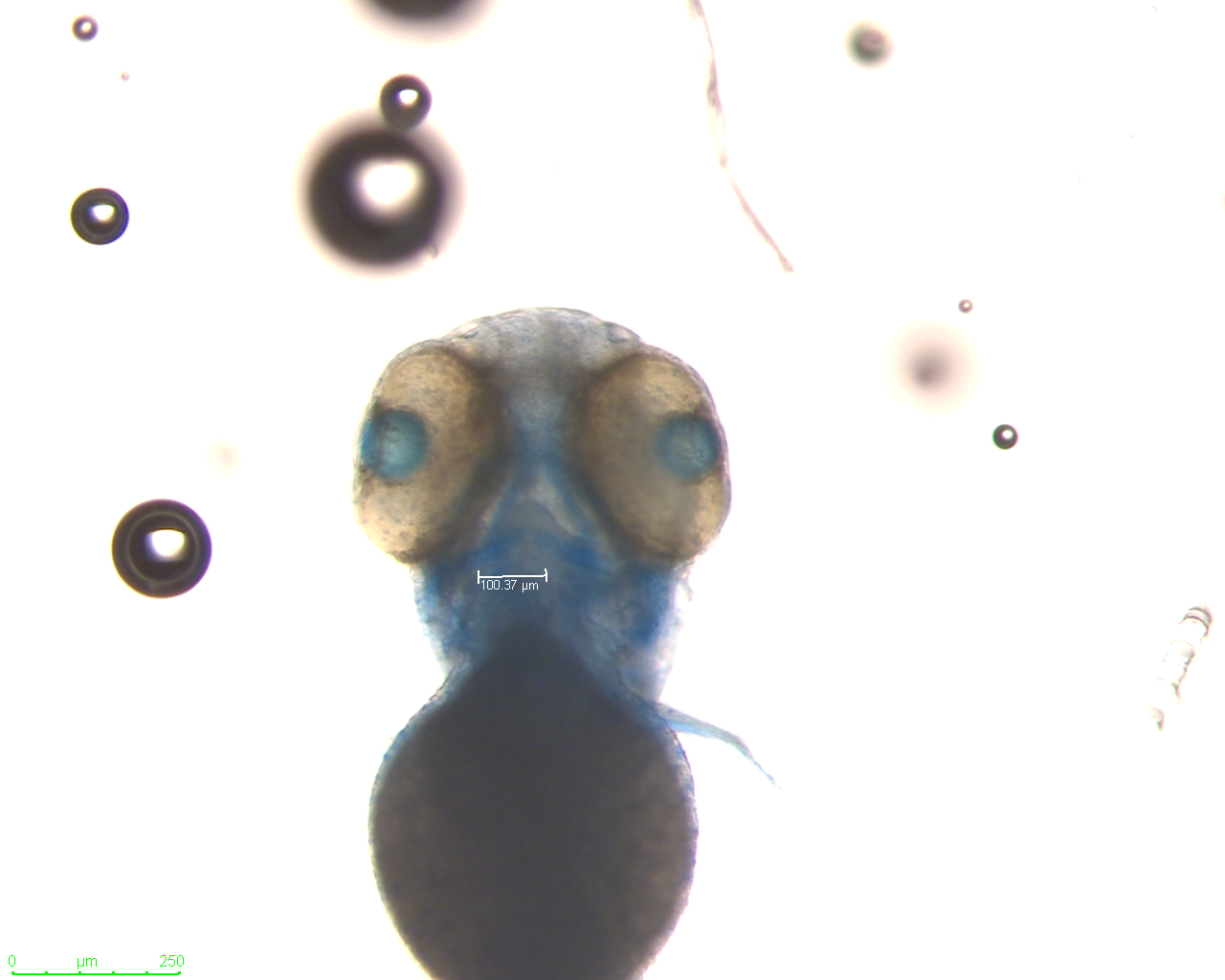

Supplement: Supplementary file 6 — Source Data [file 41467_2021_21053_MOESM6_ESM.zip › Source Data/Zebrafish Morpholino work/First replicate/EIF5A images_Control_untreated_04.tif]

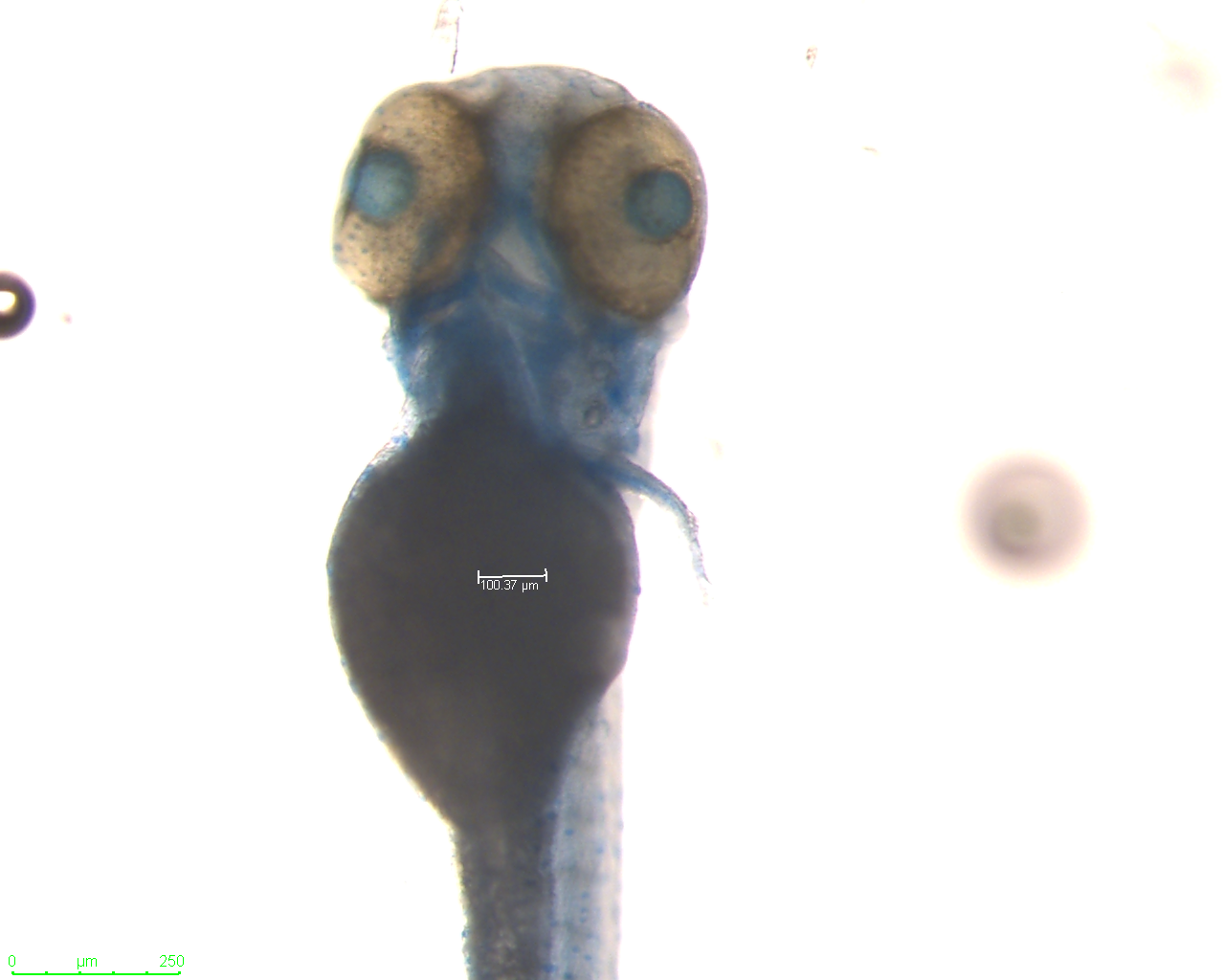

Supplement: Supplementary file 6 — Source Data [file 41467_2021_21053_MOESM6_ESM.zip › Source Data/Zebrafish Morpholino work/First replicate/EIF5A images_Control_untreated_05.tif]

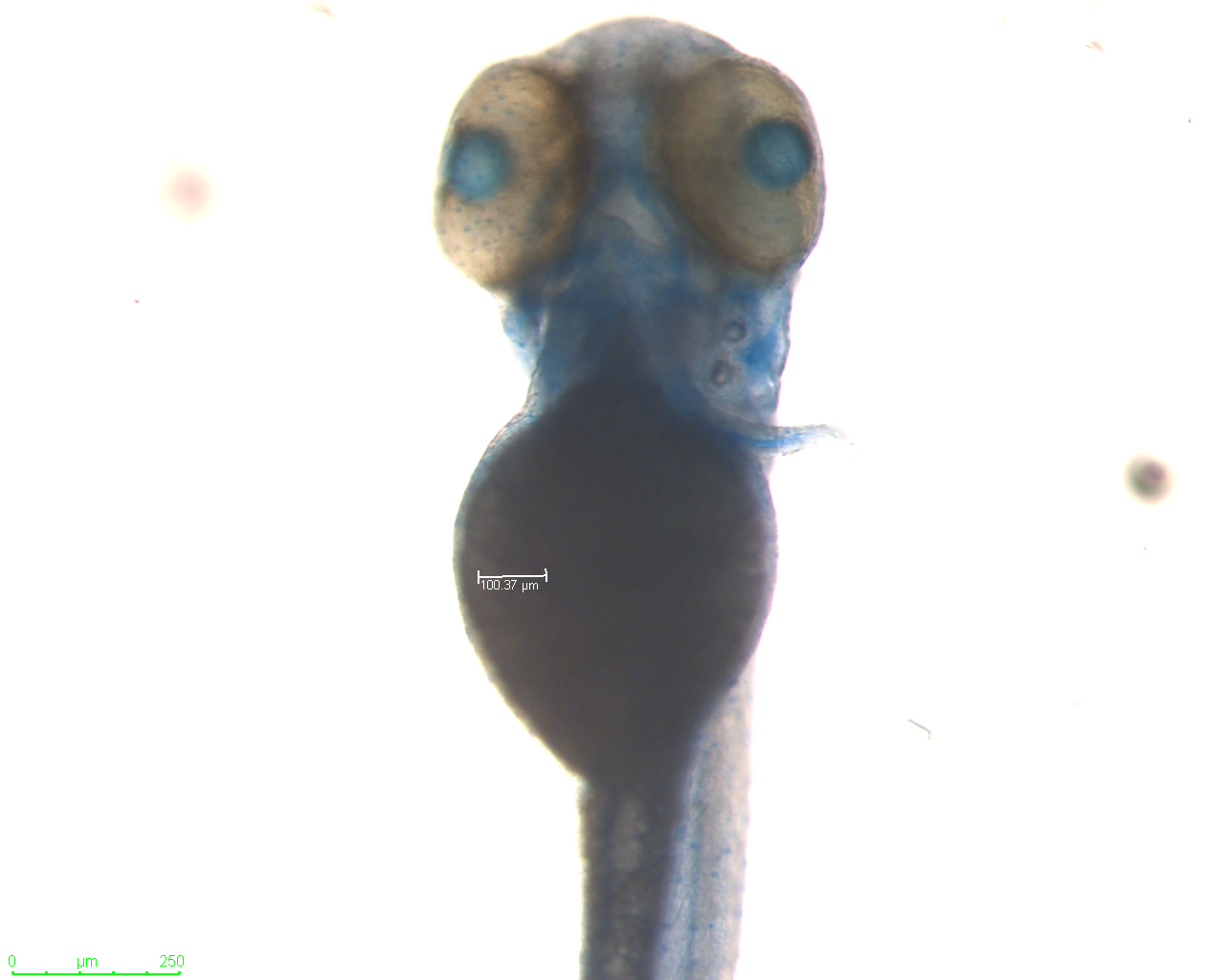

Supplement: Supplementary file 6 — Source Data [file 41467_2021_21053_MOESM6_ESM.zip › Source Data/Zebrafish Morpholino work/First replicate/EIF5A images_EIF5A_Spermadine_01.tif]

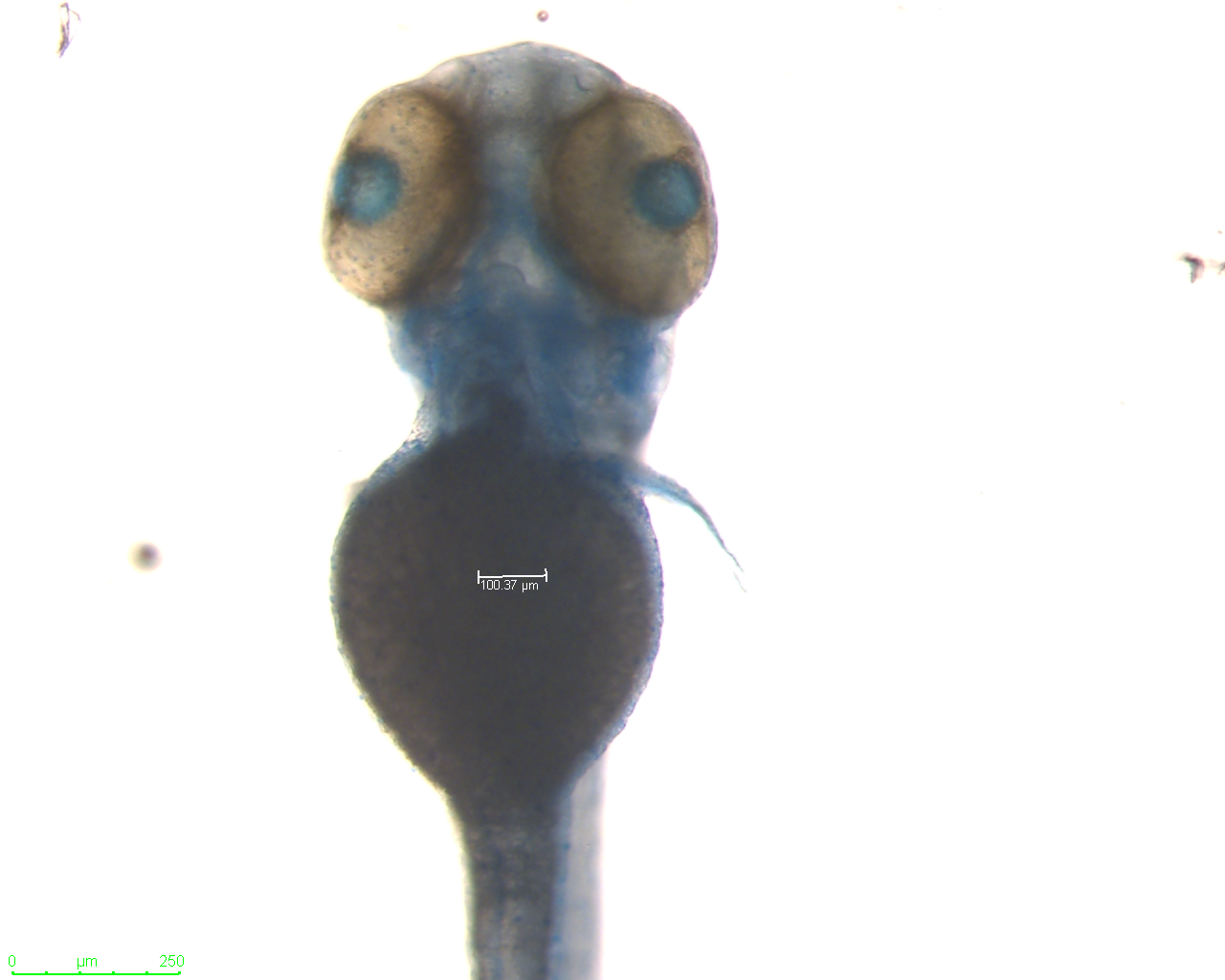

Supplement: Supplementary file 6 — Source Data [file 41467_2021_21053_MOESM6_ESM.zip › Source Data/Zebrafish Morpholino work/First replicate/EIF5A images_EIF5A_Spermadine_02.tif]

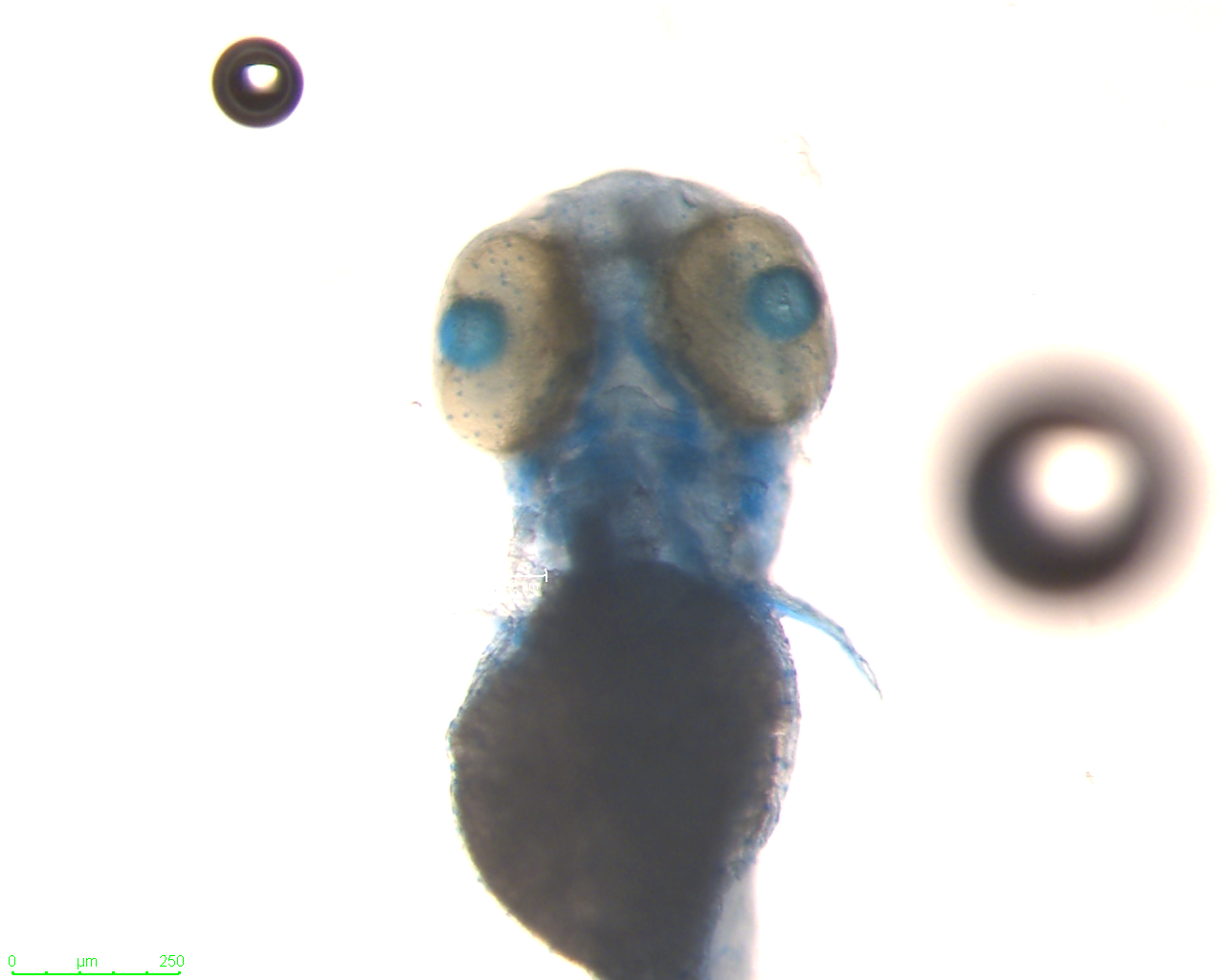

Supplement: Supplementary file 6 — Source Data [file 41467_2021_21053_MOESM6_ESM.zip › Source Data/Zebrafish Morpholino work/First replicate/EIF5A images_EIF5A_Spermadine_03.tif]

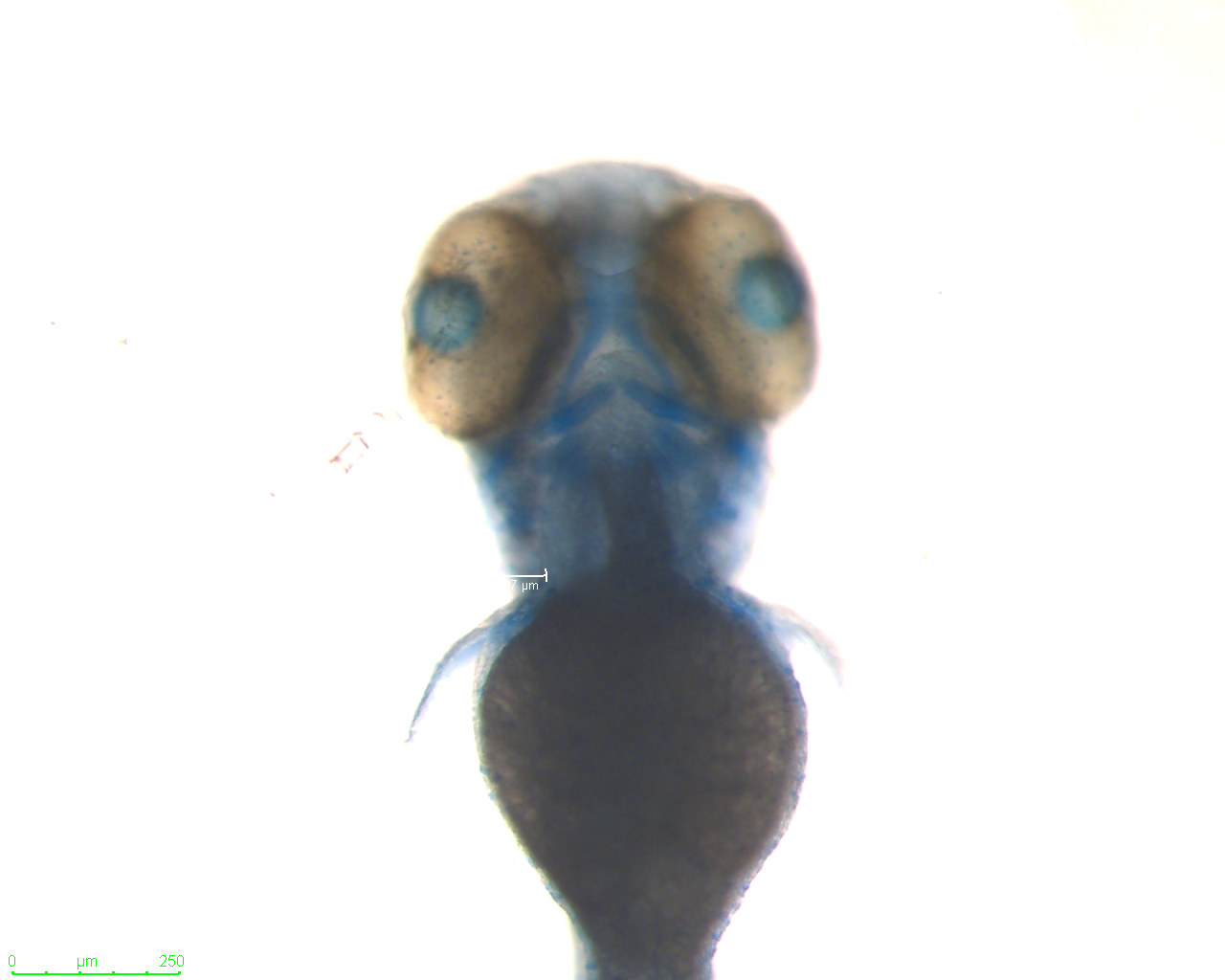

Supplement: Supplementary file 6 — Source Data [file 41467_2021_21053_MOESM6_ESM.zip › Source Data/Zebrafish Morpholino work/First replicate/EIF5A images_EIF5A_Spermadine_04.tif]

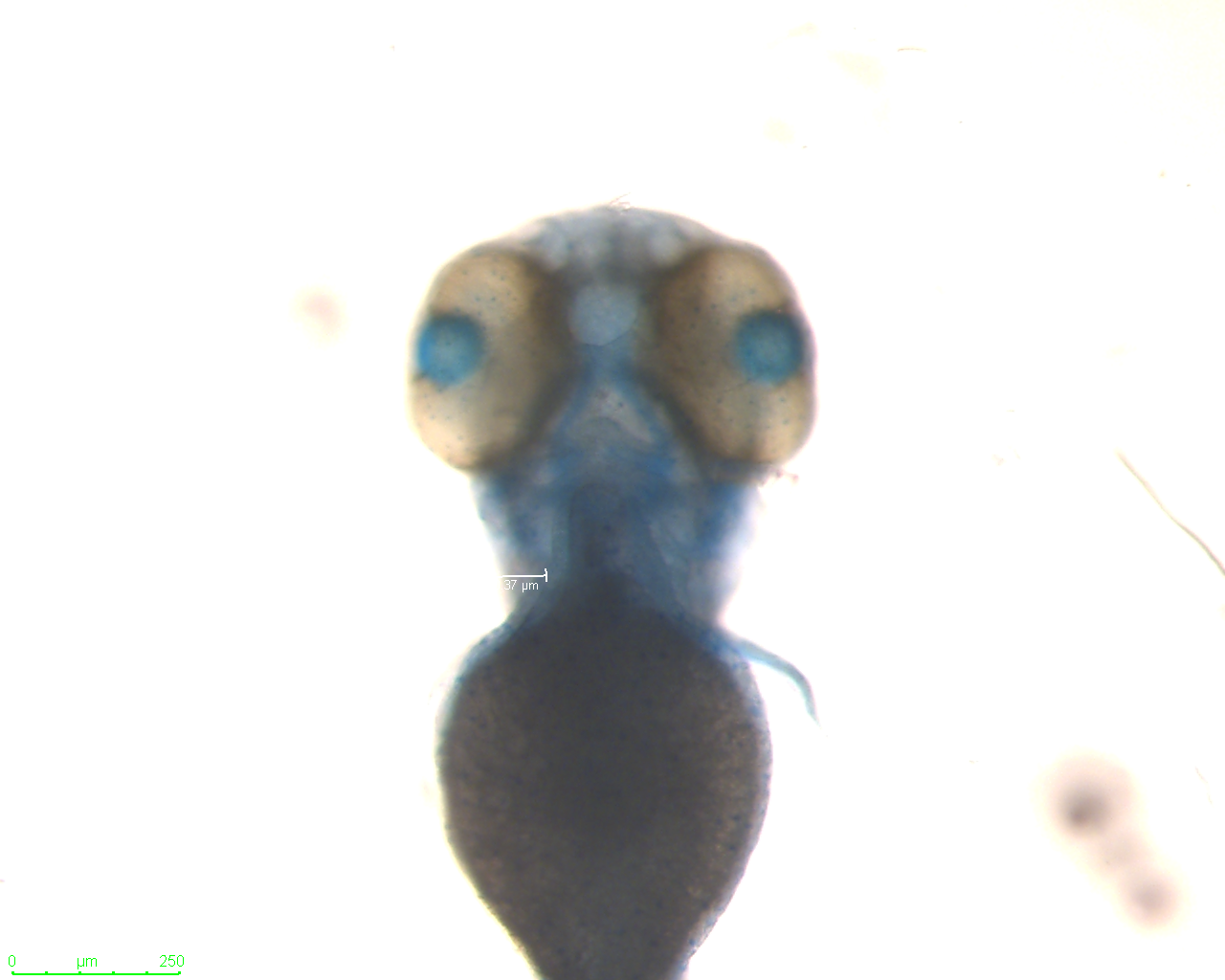

Supplement: Supplementary file 6 — Source Data [file 41467_2021_21053_MOESM6_ESM.zip › Source Data/Zebrafish Morpholino work/First replicate/EIF5A images_EIF5A_Spermadine_05.tif]

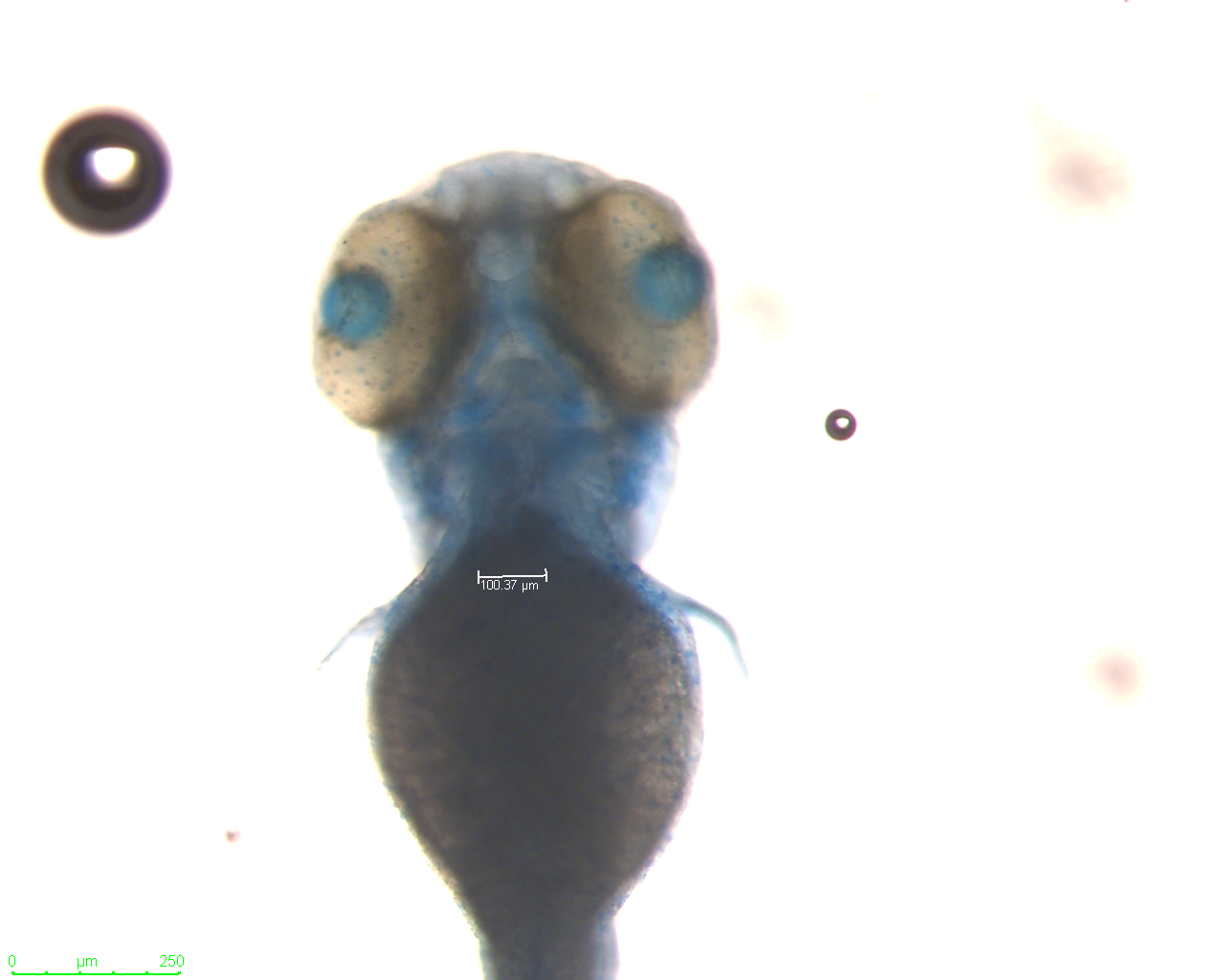

Supplement: Supplementary file 6 — Source Data [file 41467_2021_21053_MOESM6_ESM.zip › Source Data/Zebrafish Morpholino work/First replicate/EIF5A images_EIF5A_Spermadine_06.tif]

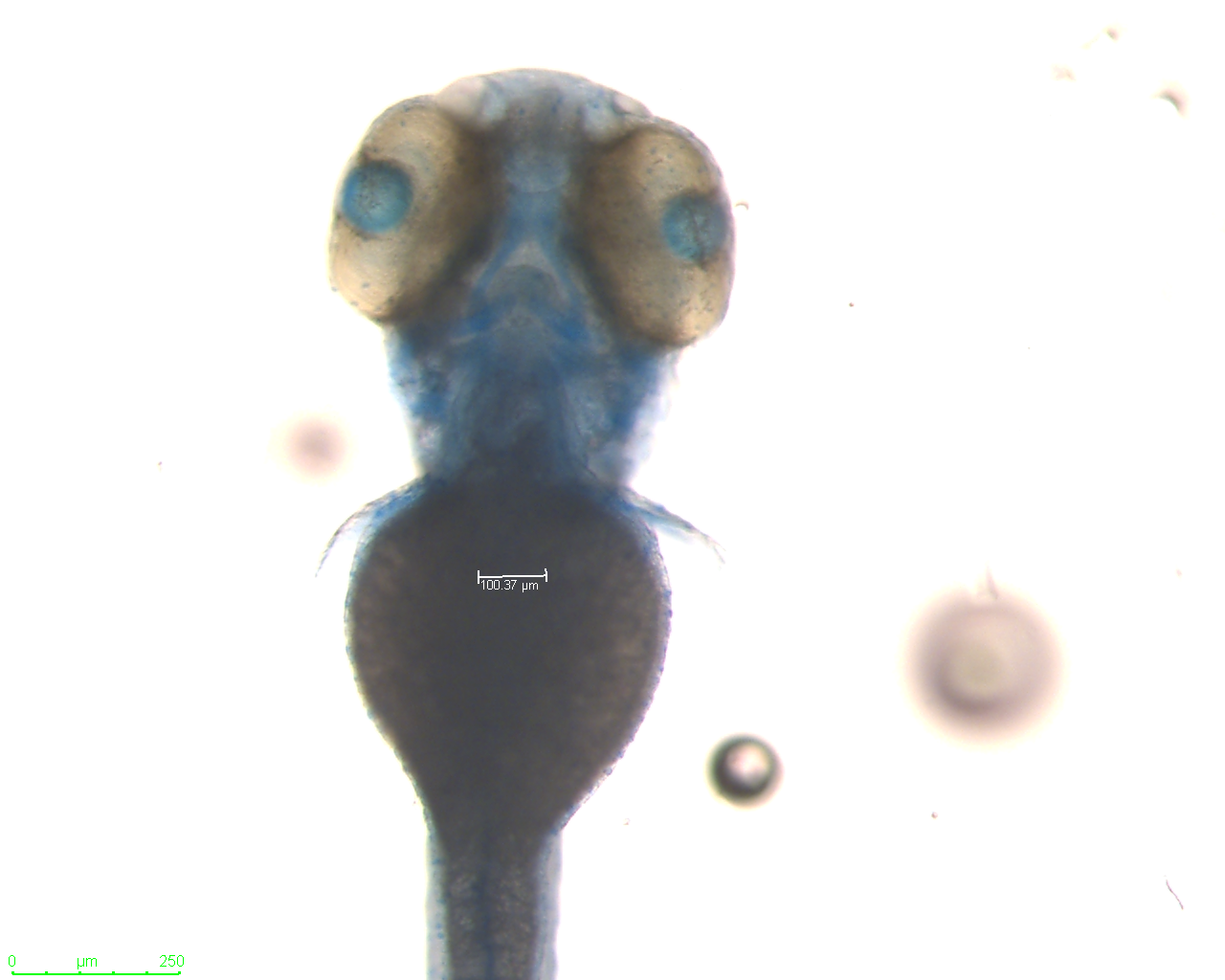

Supplement: Supplementary file 6 — Source Data [file 41467_2021_21053_MOESM6_ESM.zip › Source Data/Zebrafish Morpholino work/First replicate/EIF5A images_EIF5A_Spermadine_07.tif]

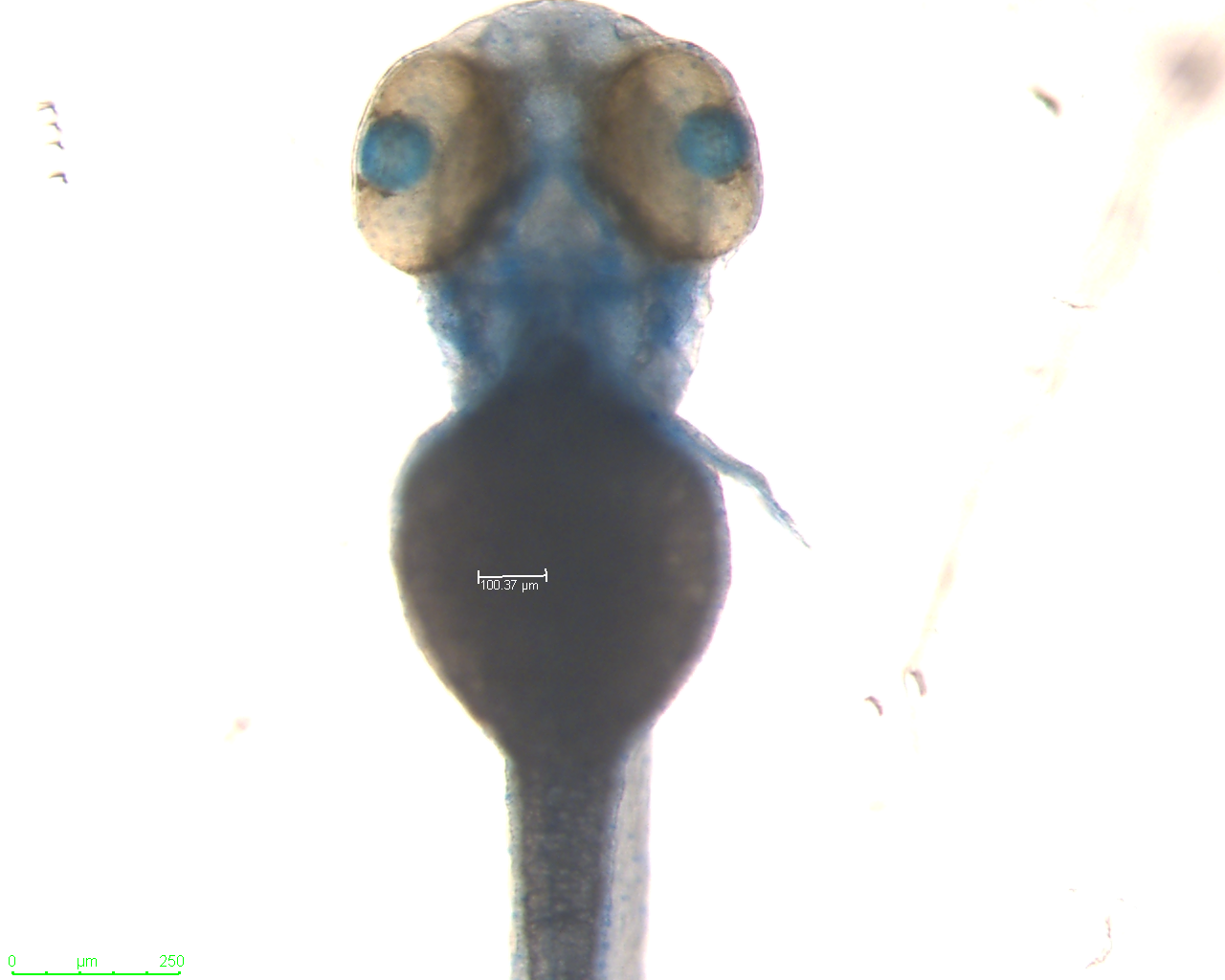

Supplement: Supplementary file 6 — Source Data [file 41467_2021_21053_MOESM6_ESM.zip › Source Data/Zebrafish Morpholino work/First replicate/EIF5A images_EIF5A_Spermadine_08.tif]

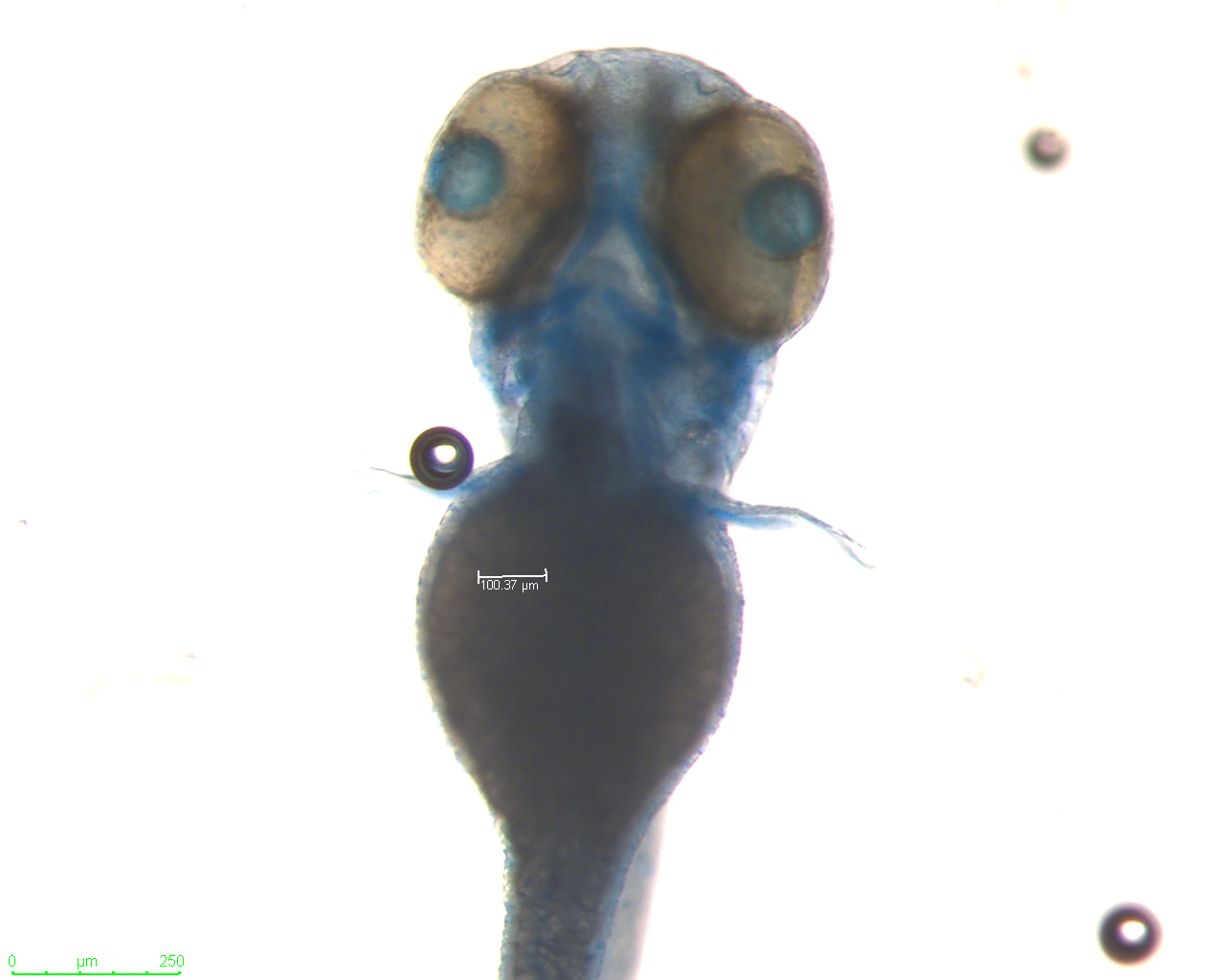

Supplement: Supplementary file 6 — Source Data [file 41467_2021_21053_MOESM6_ESM.zip › Source Data/Zebrafish Morpholino work/First replicate/EIF5A images_EIF5A_Spermadine_09.tif]

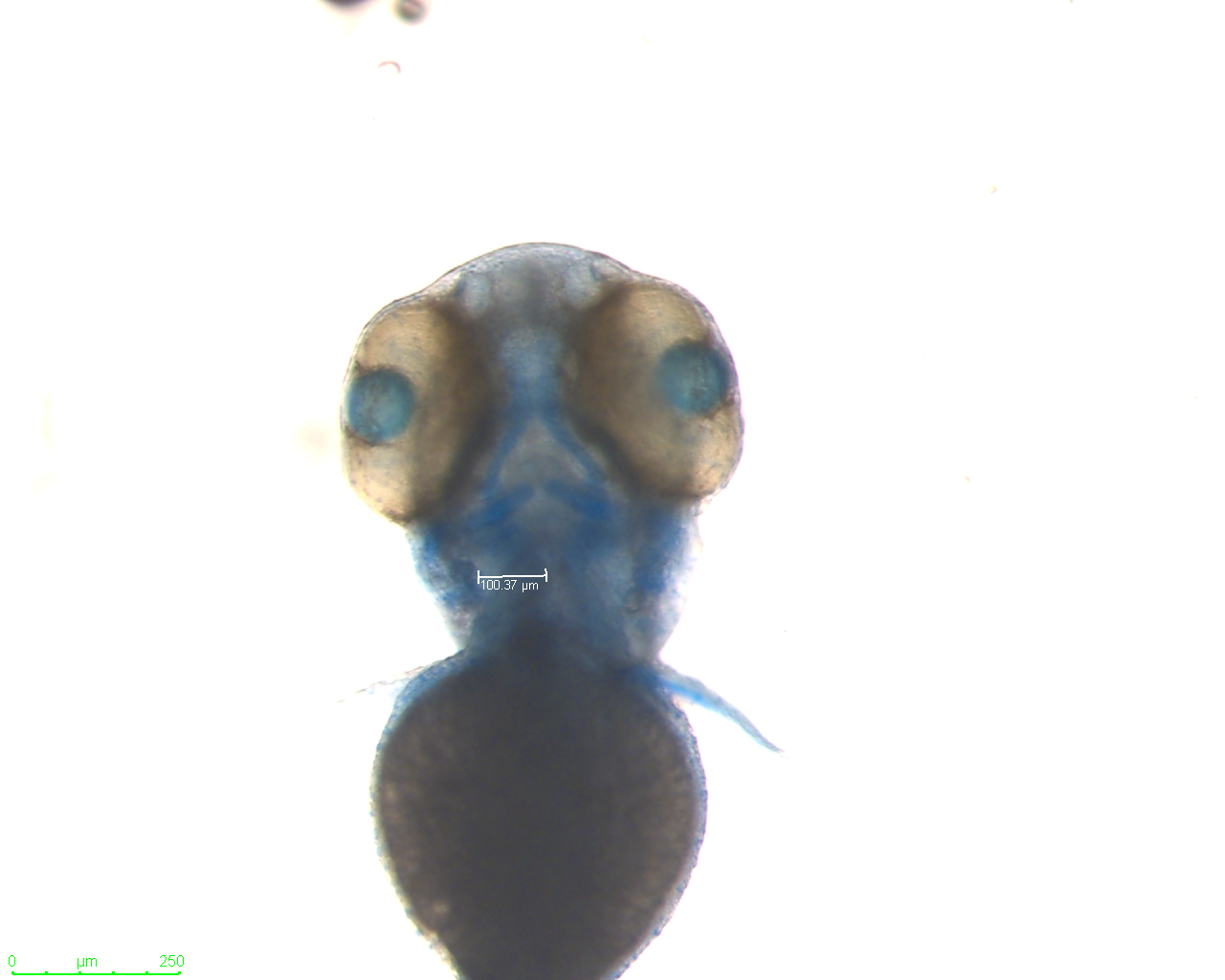

Supplement: Supplementary file 6 — Source Data [file 41467_2021_21053_MOESM6_ESM.zip › Source Data/Zebrafish Morpholino work/First replicate/EIF5A images_EIF5A_Spermadine_10.tif]

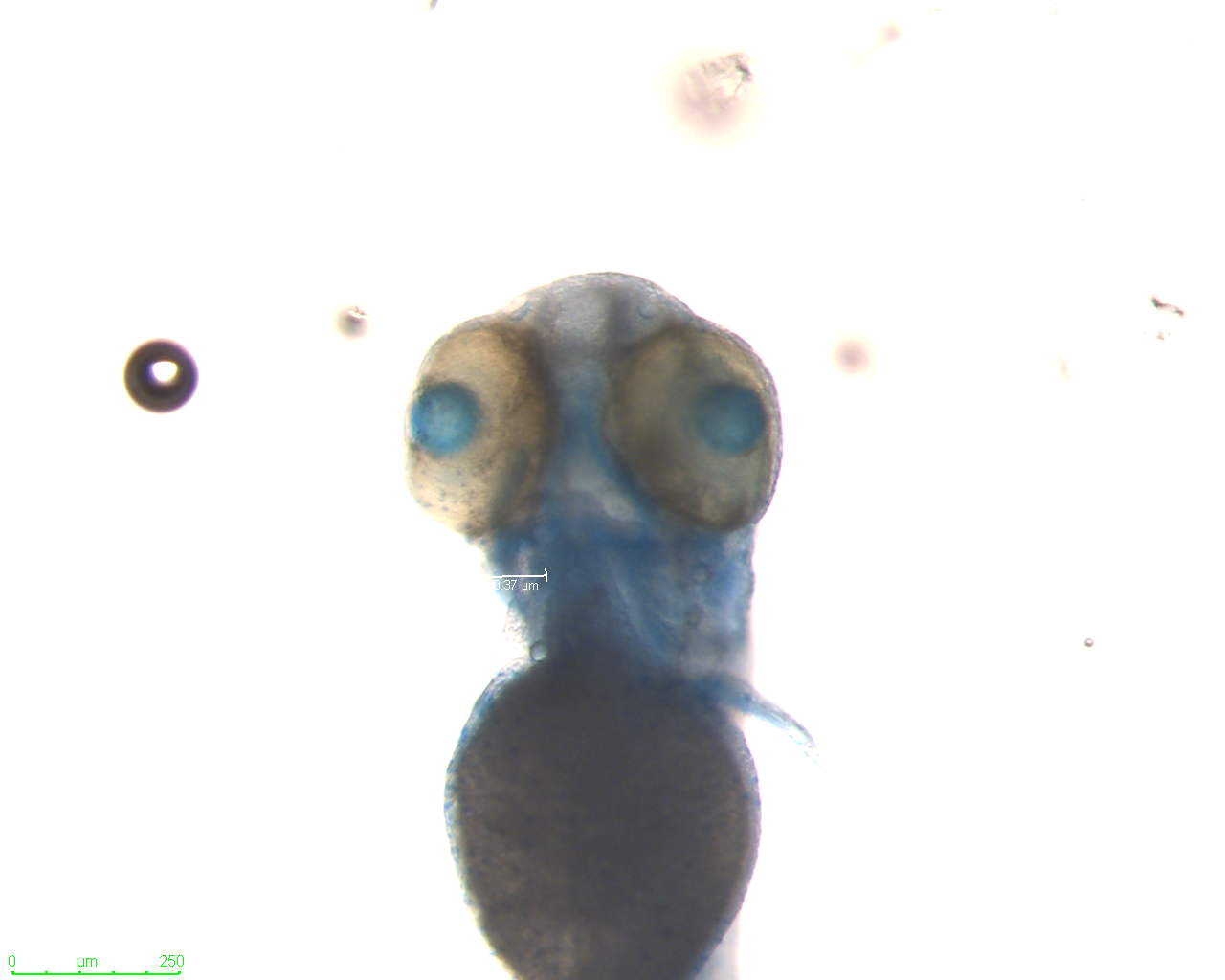

Supplement: Supplementary file 6 — Source Data [file 41467_2021_21053_MOESM6_ESM.zip › Source Data/Zebrafish Morpholino work/First replicate/EIF5A images_EIF5A_untreated_01.tif]

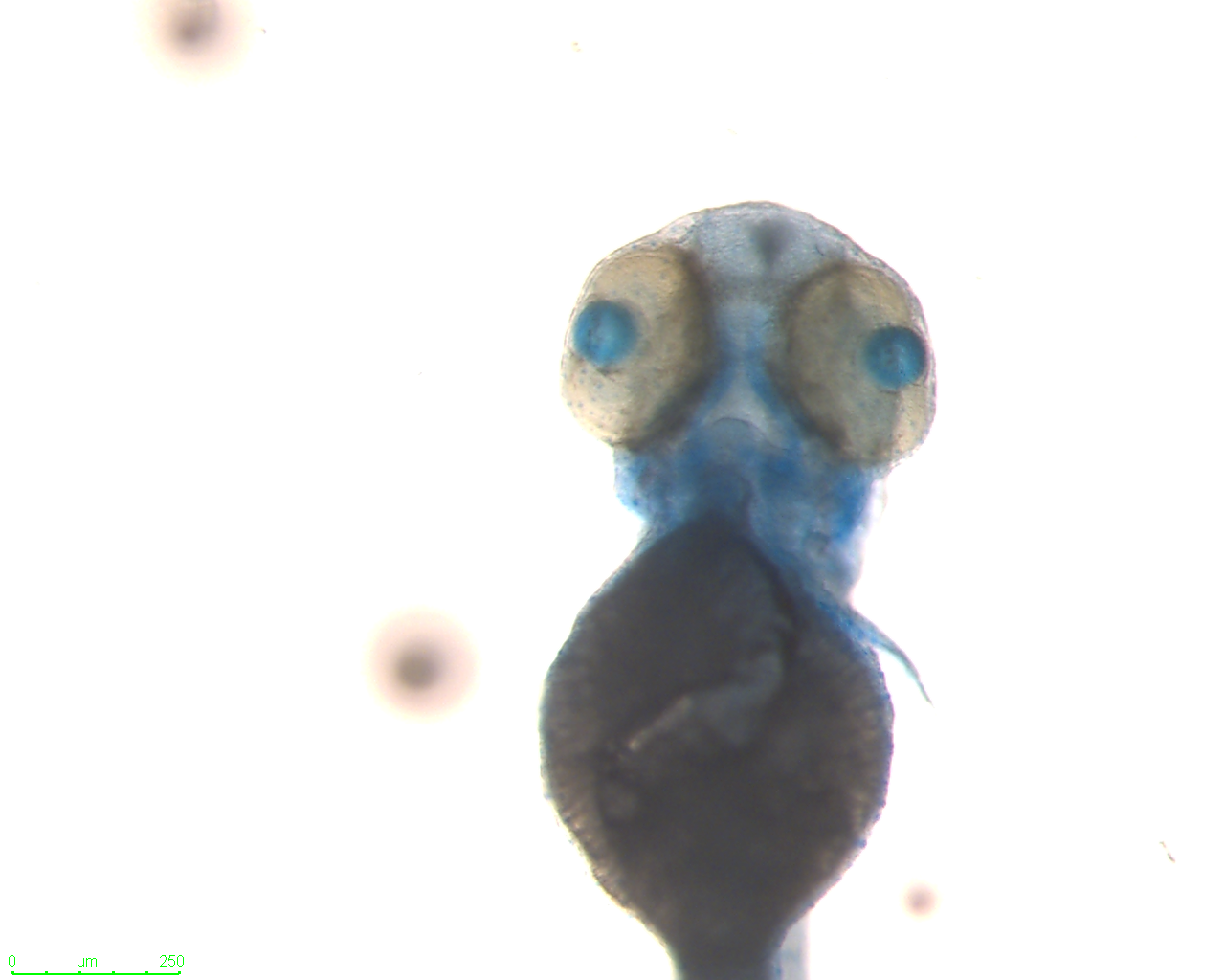

Supplement: Supplementary file 6 — Source Data [file 41467_2021_21053_MOESM6_ESM.zip › Source Data/Zebrafish Morpholino work/First replicate/EIF5A images_EIF5A_untreated_02.tif]

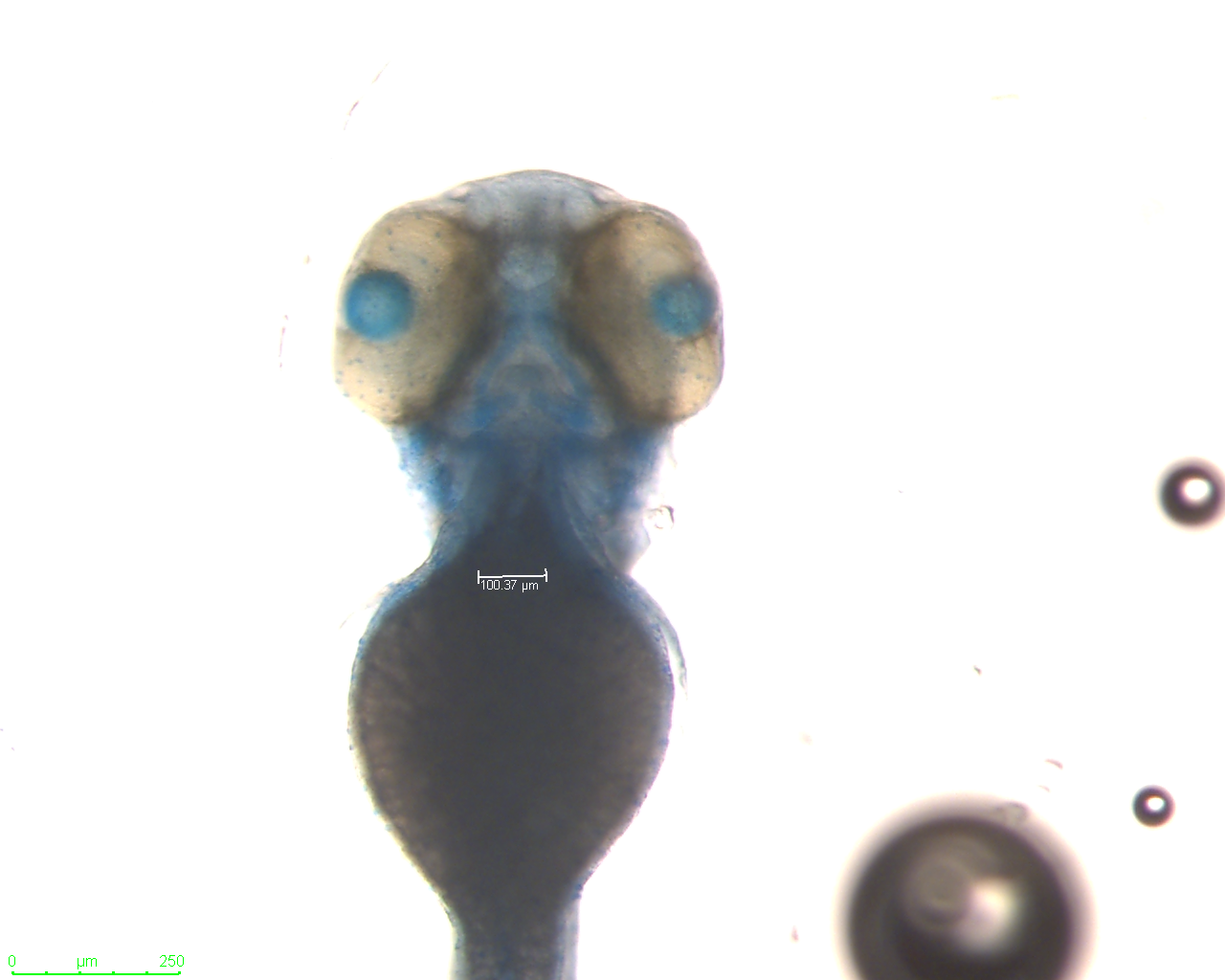

Supplement: Supplementary file 6 — Source Data [file 41467_2021_21053_MOESM6_ESM.zip › Source Data/Zebrafish Morpholino work/First replicate/EIF5A images_EIF5A_untreated_03.tif]

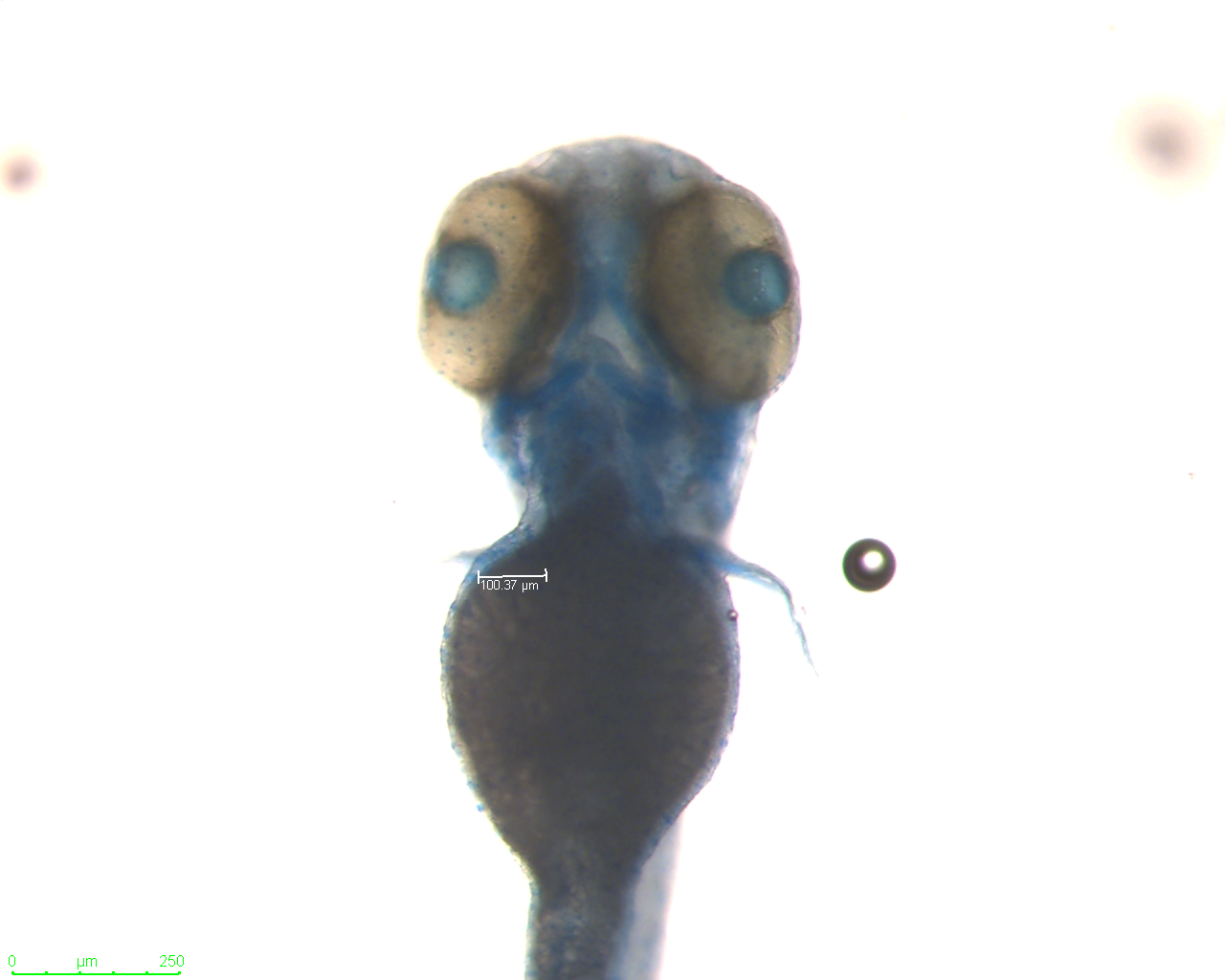

Supplement: Supplementary file 6 — Source Data [file 41467_2021_21053_MOESM6_ESM.zip › Source Data/Zebrafish Morpholino work/First replicate/EIF5A images_EIF5A_untreated_04.tif]

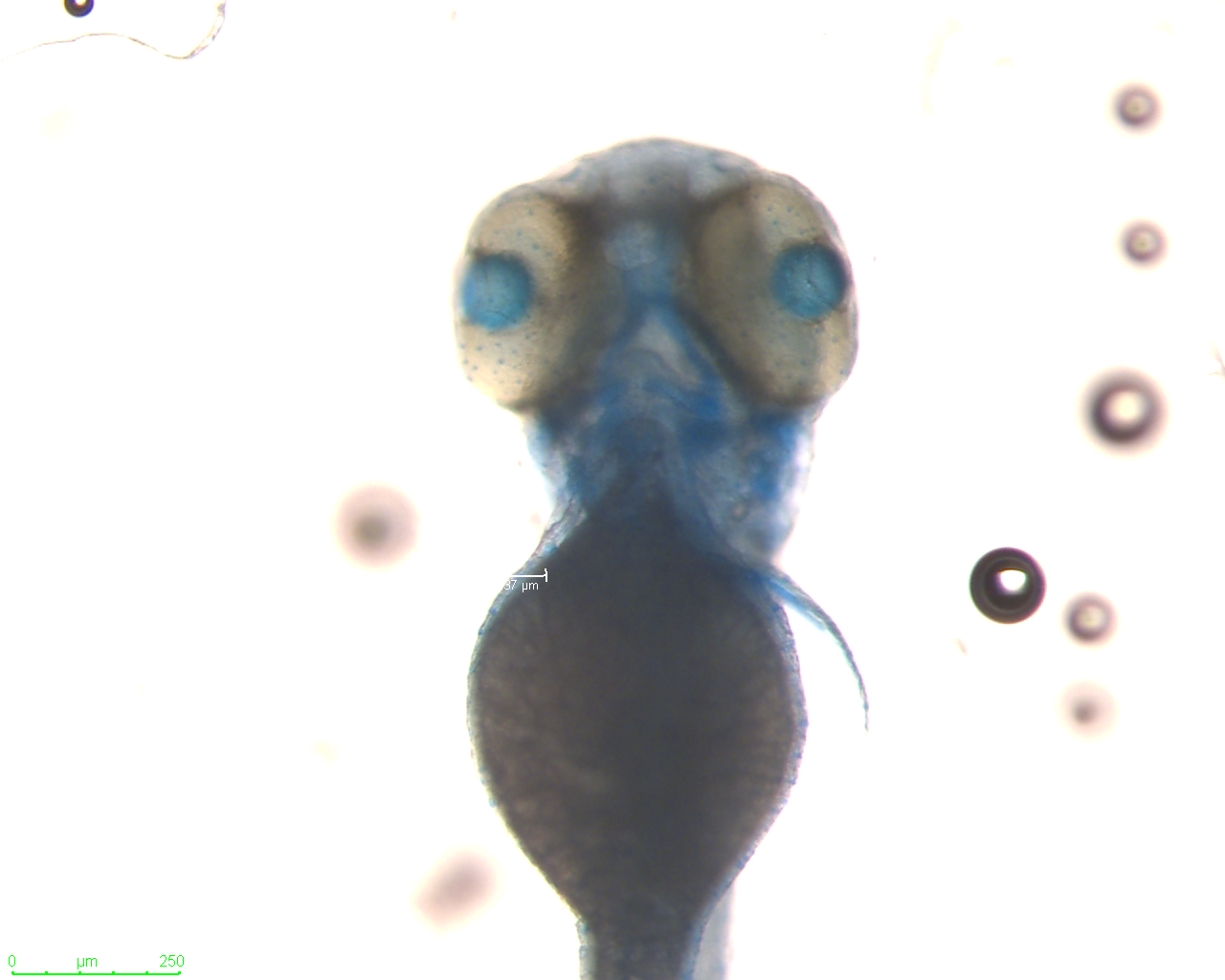

Supplement: Supplementary file 6 — Source Data [file 41467_2021_21053_MOESM6_ESM.zip › Source Data/Zebrafish Morpholino work/First replicate/EIF5A images_EIF5A_untreated_05.tif]

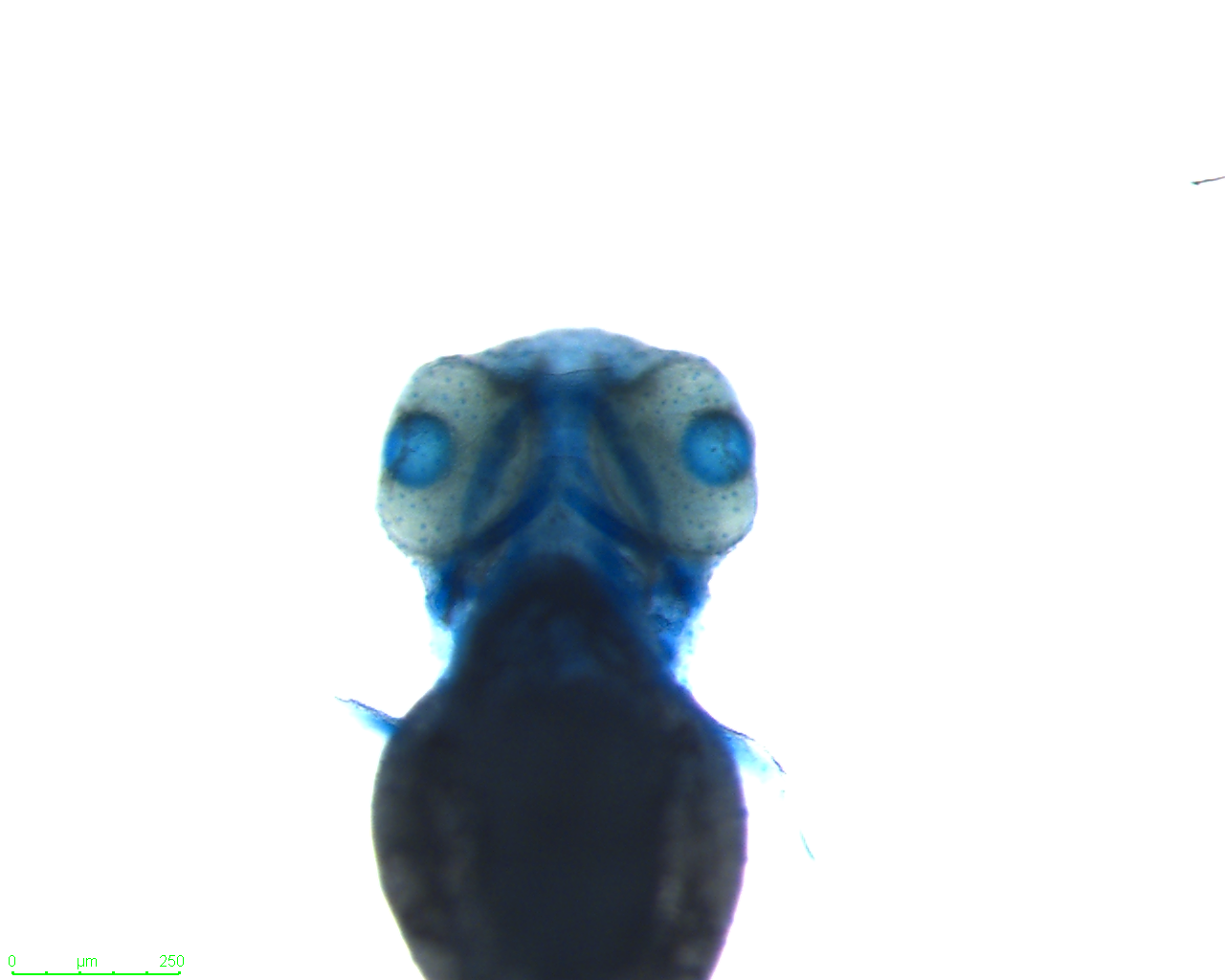

Supplement: Supplementary file 6 — Source Data [file 41467_2021_21053_MOESM6_ESM.zip › Source Data/Zebrafish Morpholino work/Second replicate/EIF5A expt 2Rs_Control_sperm 1.1.tif]

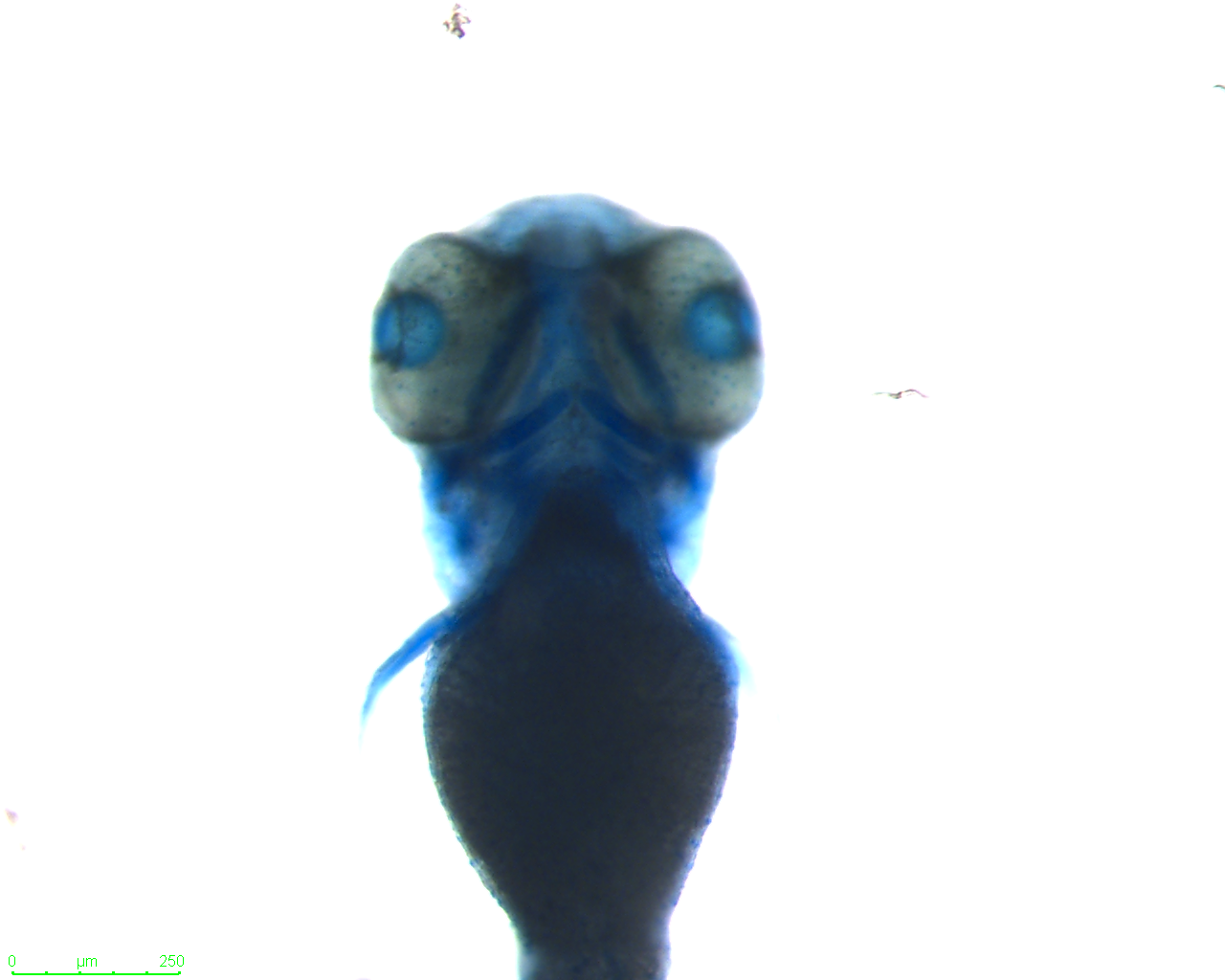

Supplement: Supplementary file 6 — Source Data [file 41467_2021_21053_MOESM6_ESM.zip › Source Data/Zebrafish Morpholino work/Second replicate/EIF5A expt 2Rs_Control_sperm 1.10.tif]

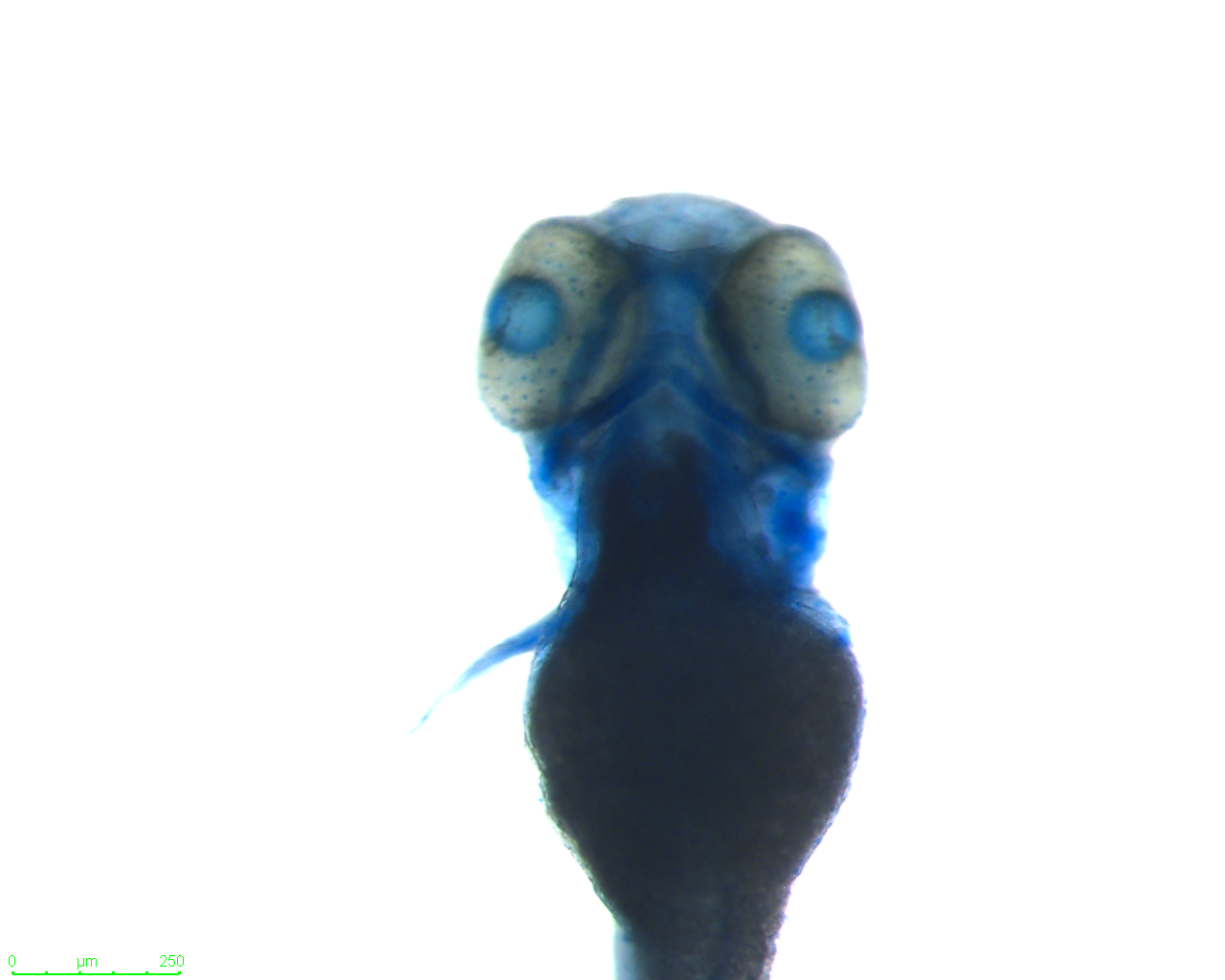

Supplement: Supplementary file 6 — Source Data [file 41467_2021_21053_MOESM6_ESM.zip › Source Data/Zebrafish Morpholino work/Second replicate/EIF5A expt 2Rs_Control_sperm 1.11.tif]

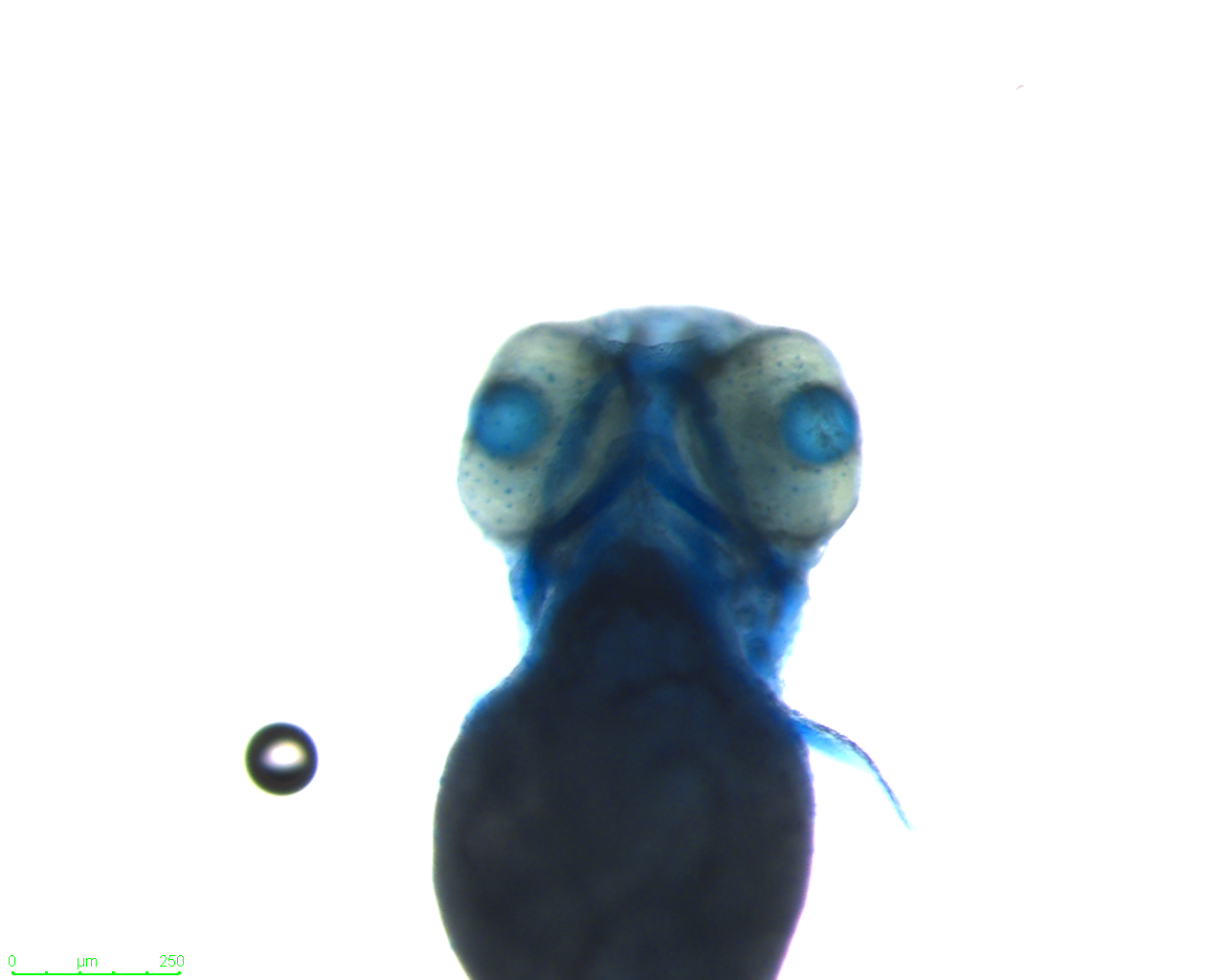

Supplement: Supplementary file 6 — Source Data [file 41467_2021_21053_MOESM6_ESM.zip › Source Data/Zebrafish Morpholino work/Second replicate/EIF5A expt 2Rs_Control_sperm 1.2.tif]

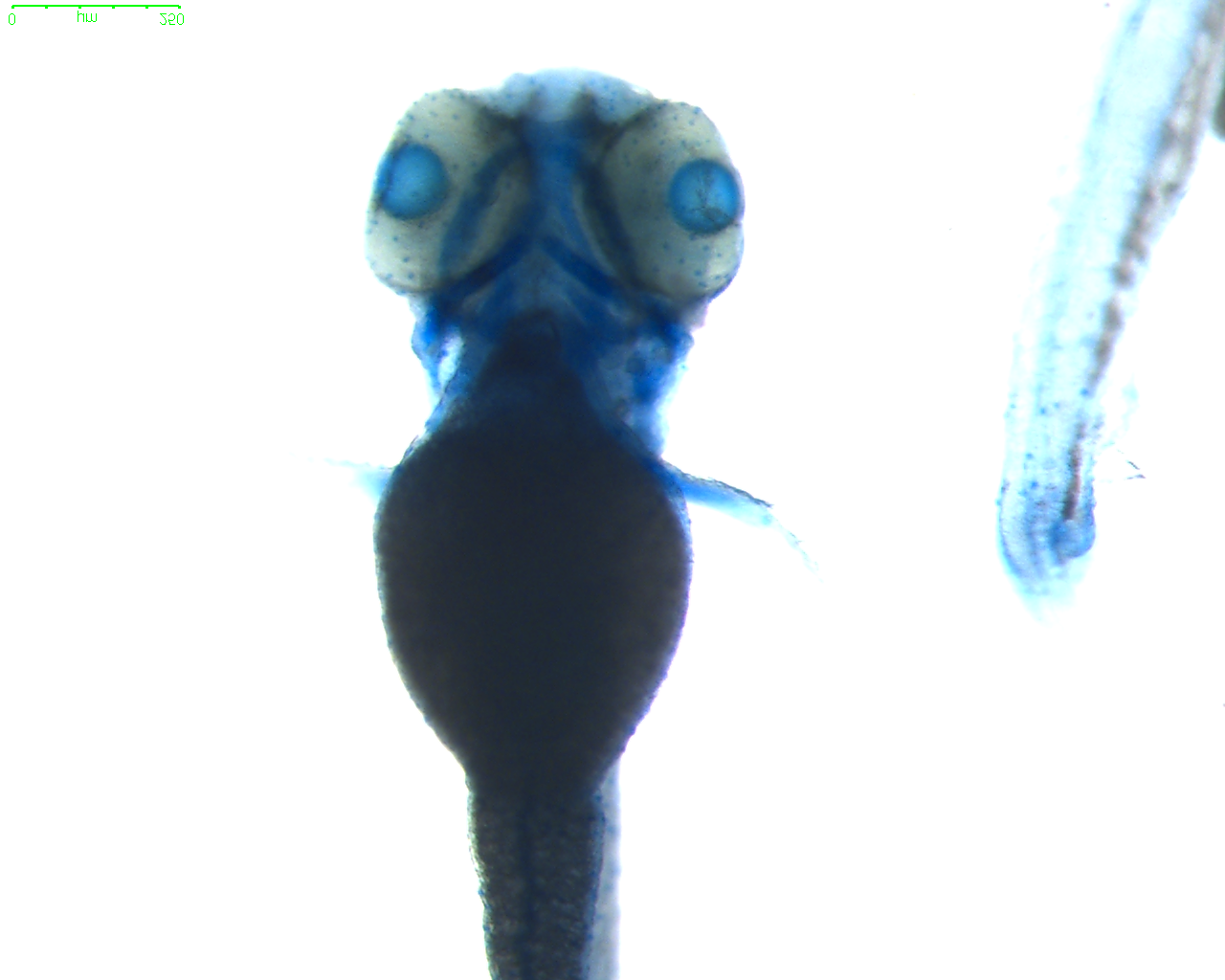

Supplement: Supplementary file 6 — Source Data [file 41467_2021_21053_MOESM6_ESM.zip › Source Data/Zebrafish Morpholino work/Second replicate/EIF5A expt 2Rs_Control_sperm 1.3.tif]

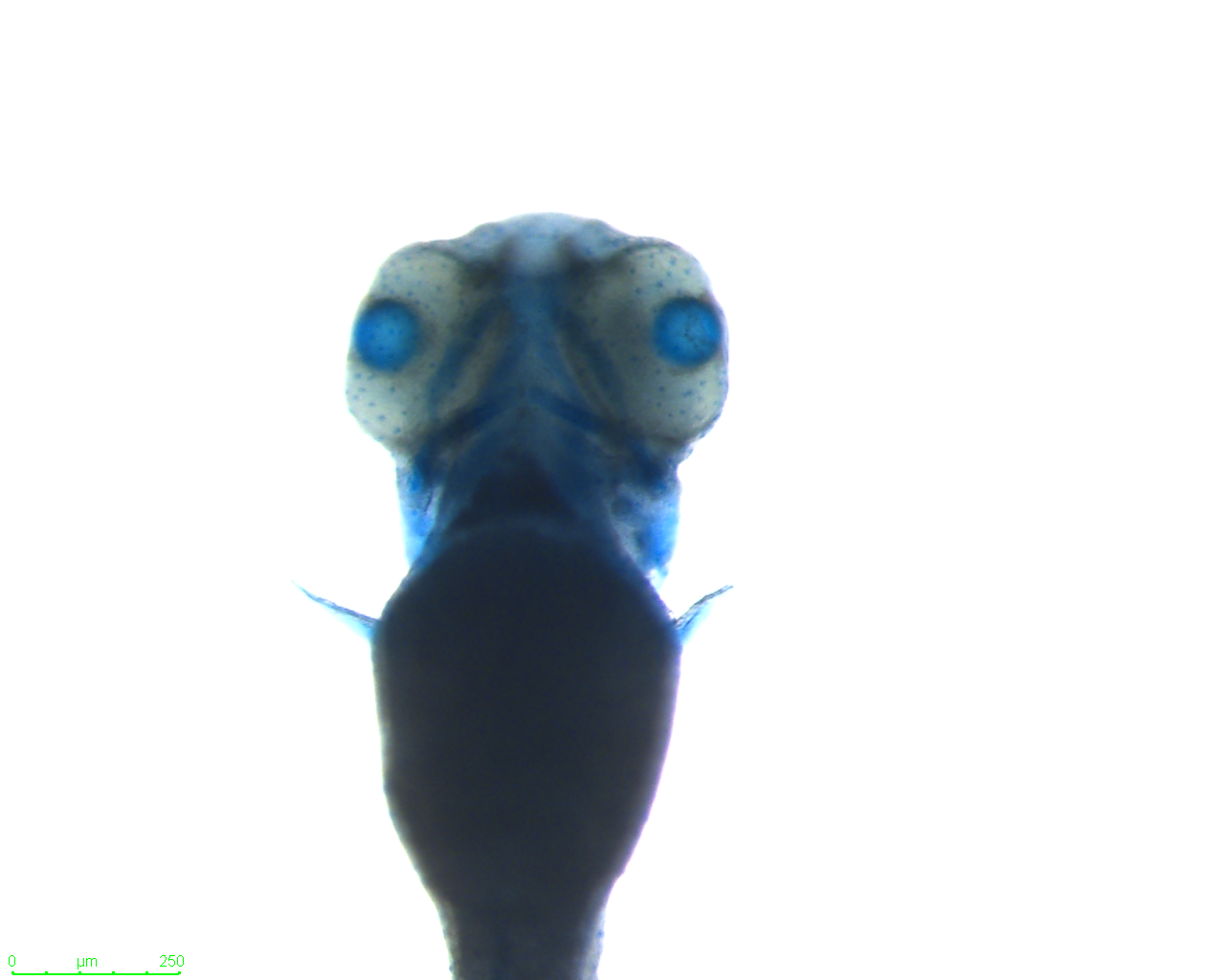

Supplement: Supplementary file 6 — Source Data [file 41467_2021_21053_MOESM6_ESM.zip › Source Data/Zebrafish Morpholino work/Second replicate/EIF5A expt 2Rs_Control_sperm 1.4.tif]

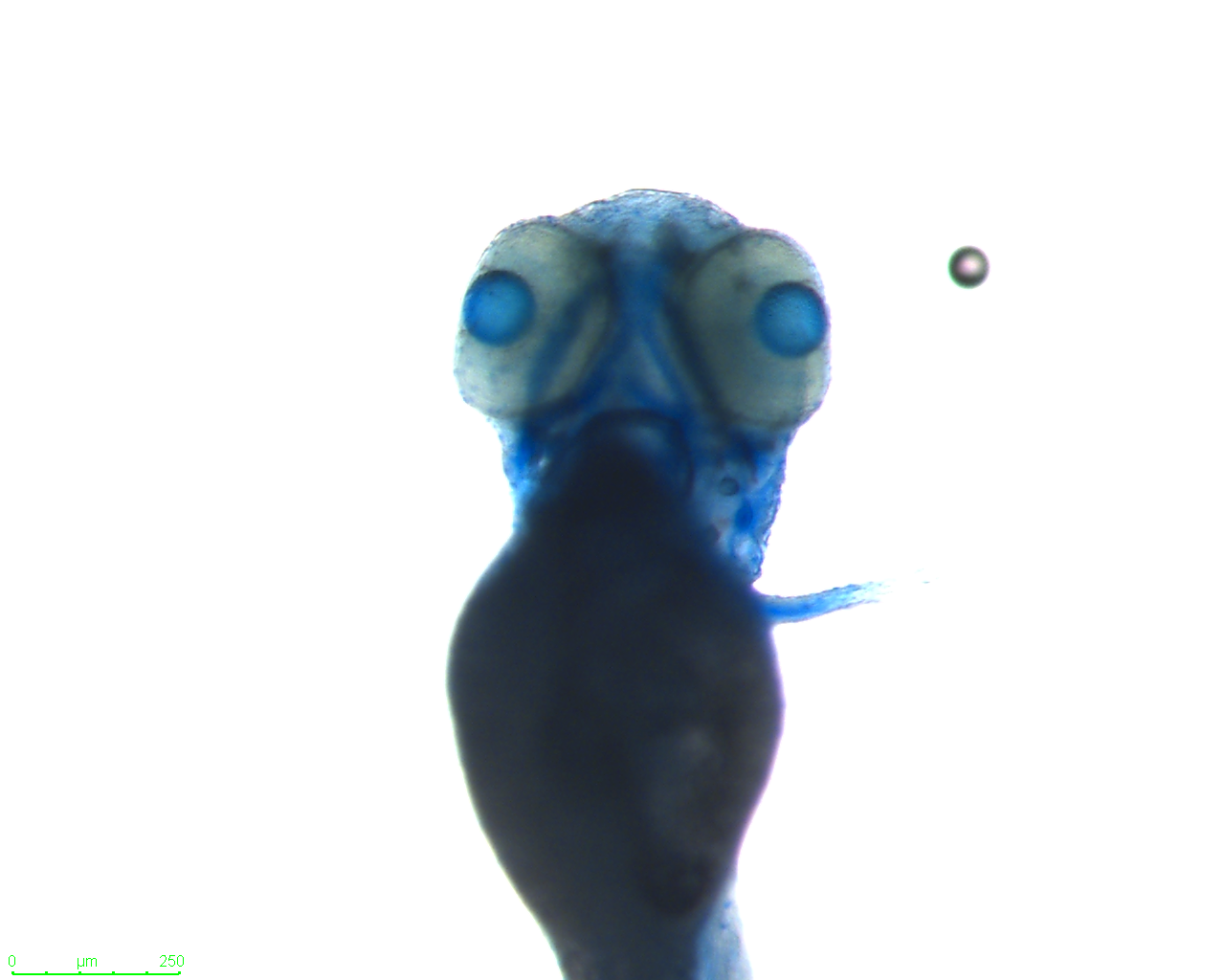

Supplement: Supplementary file 6 — Source Data [file 41467_2021_21053_MOESM6_ESM.zip › Source Data/Zebrafish Morpholino work/Second replicate/EIF5A expt 2Rs_Control_sperm 1.5.tif]

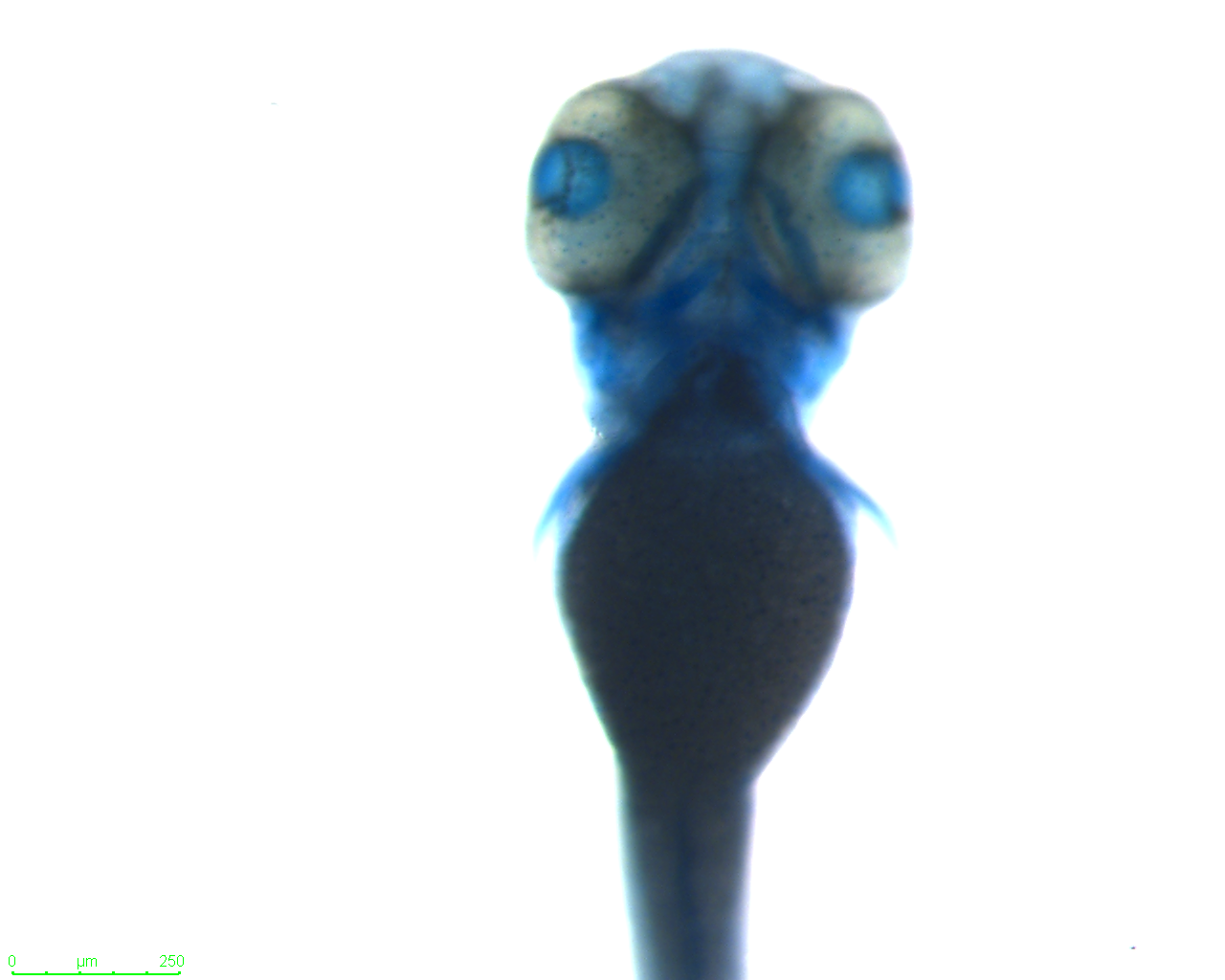

Supplement: Supplementary file 6 — Source Data [file 41467_2021_21053_MOESM6_ESM.zip › Source Data/Zebrafish Morpholino work/Second replicate/EIF5A expt 2Rs_Control_sperm 1.6.tif]

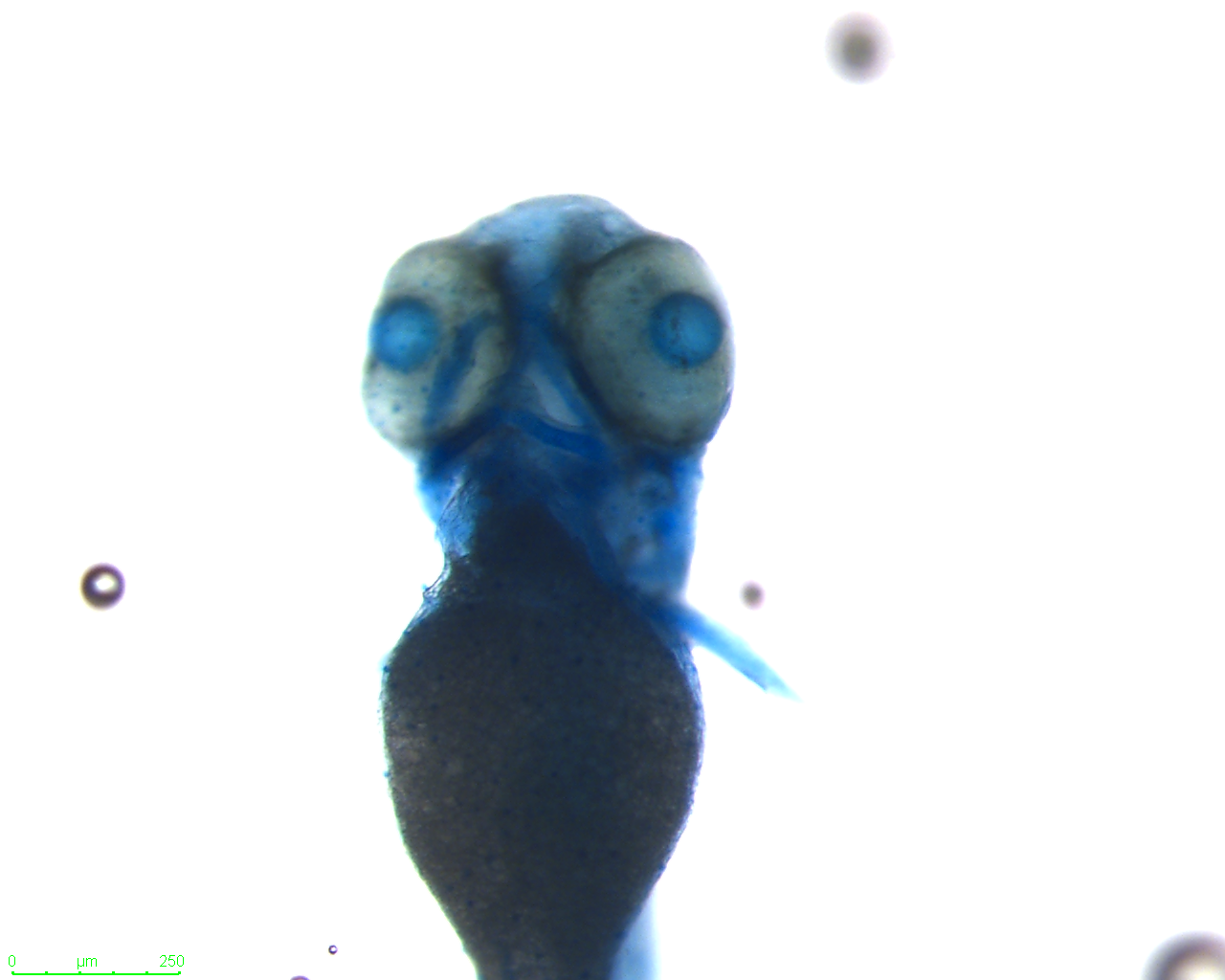

Supplement: Supplementary file 6 — Source Data [file 41467_2021_21053_MOESM6_ESM.zip › Source Data/Zebrafish Morpholino work/Second replicate/EIF5A expt 2Rs_Control_sperm 1.7.tif]

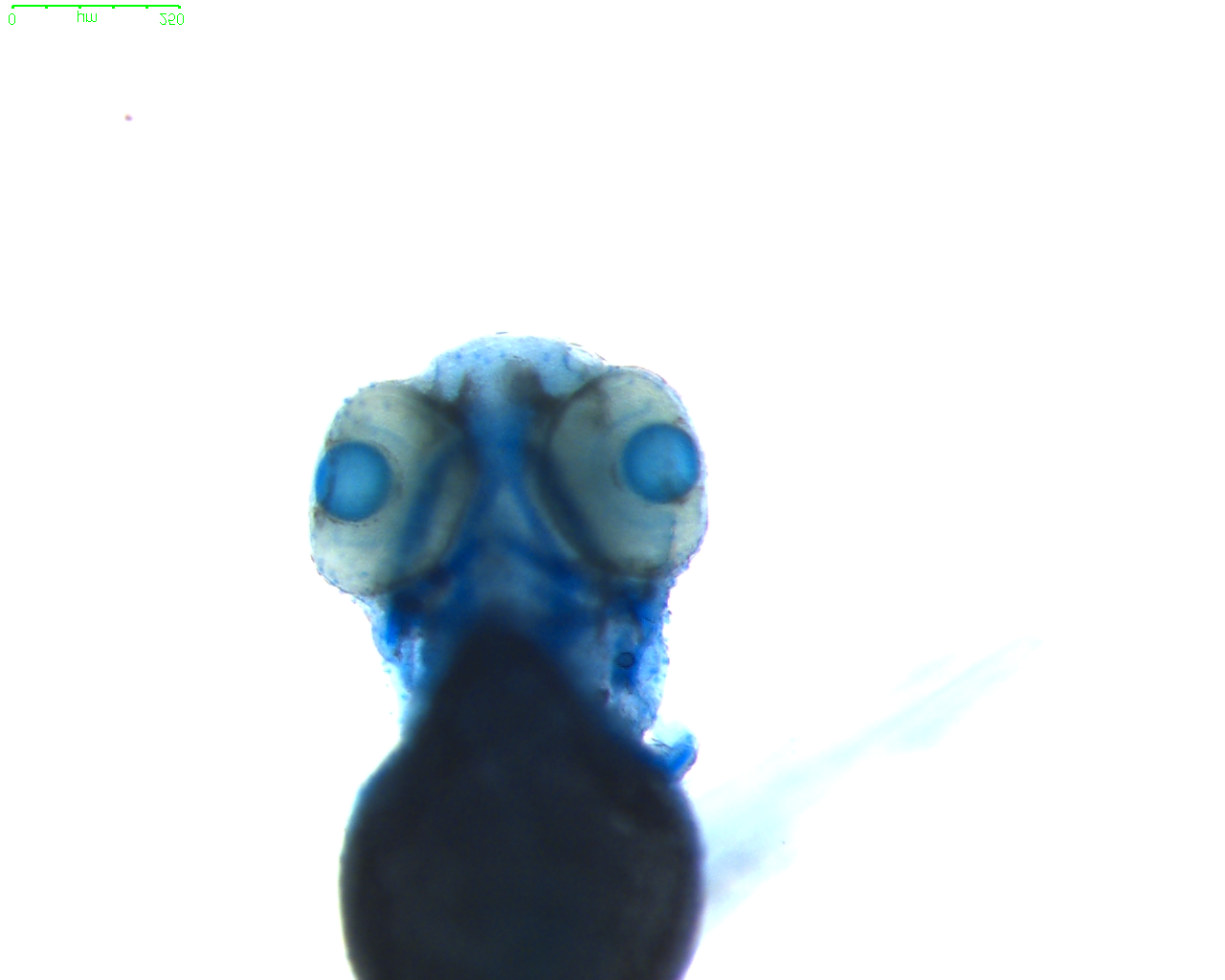

Supplement: Supplementary file 6 — Source Data [file 41467_2021_21053_MOESM6_ESM.zip › Source Data/Zebrafish Morpholino work/Second replicate/EIF5A expt 2Rs_Control_sperm 1.8.tif]

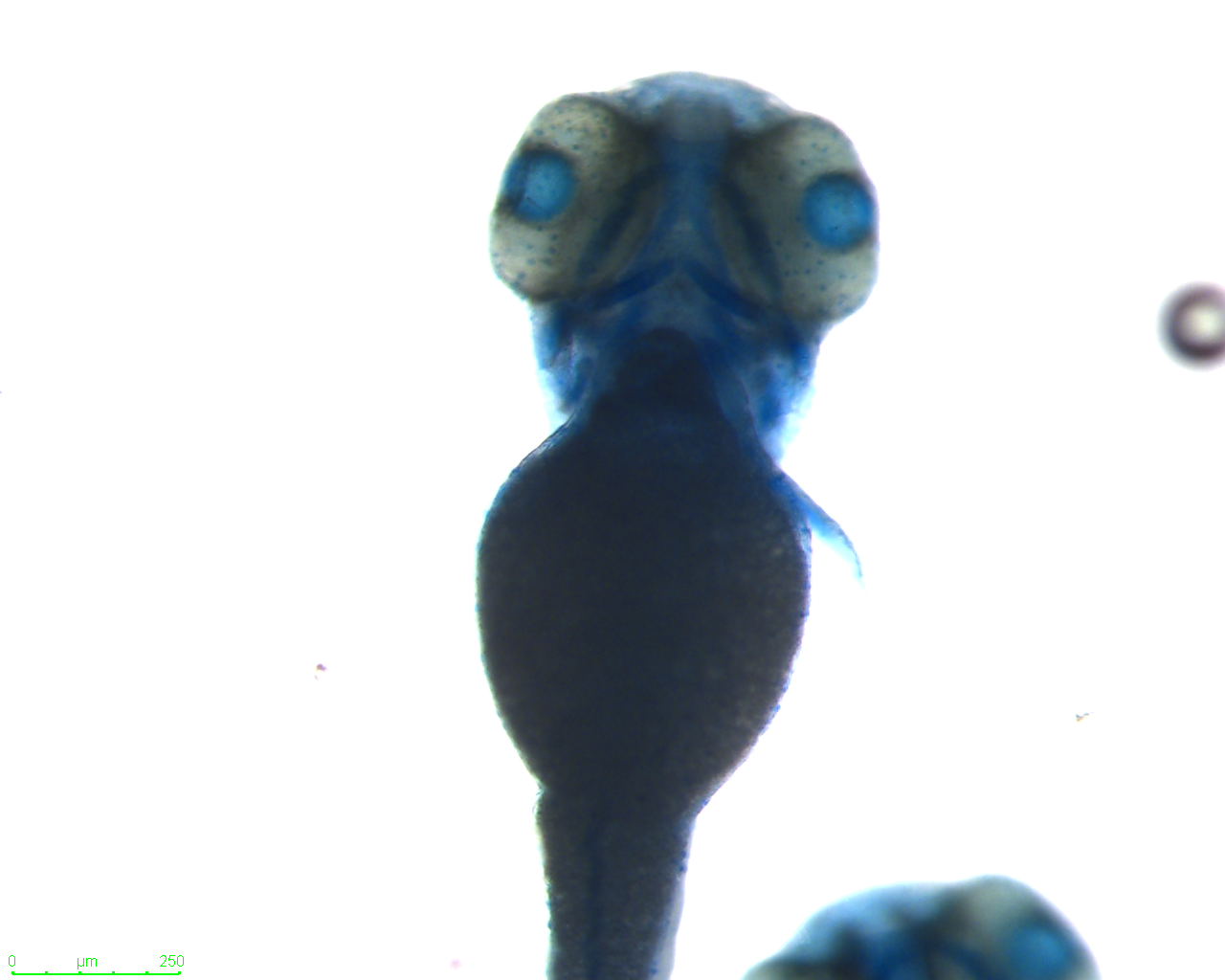

Supplement: Supplementary file 6 — Source Data [file 41467_2021_21053_MOESM6_ESM.zip › Source Data/Zebrafish Morpholino work/Second replicate/EIF5A expt 2Rs_Control_sperm 1.9.tif]

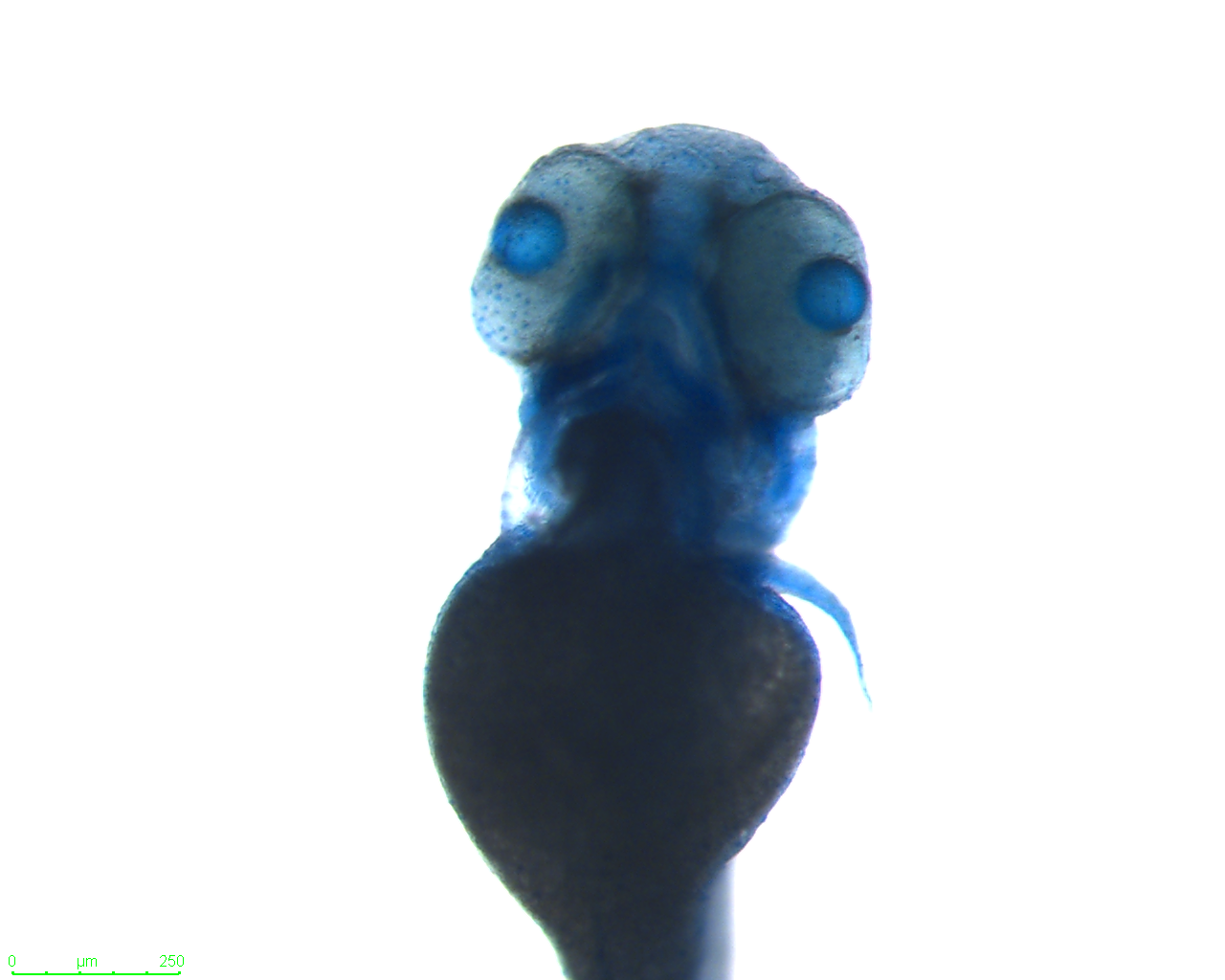

Supplement: Supplementary file 6 — Source Data [file 41467_2021_21053_MOESM6_ESM.zip › Source Data/Zebrafish Morpholino work/Second replicate/EIF5A expt 2Rs_Control_sperm 2.1.tif]

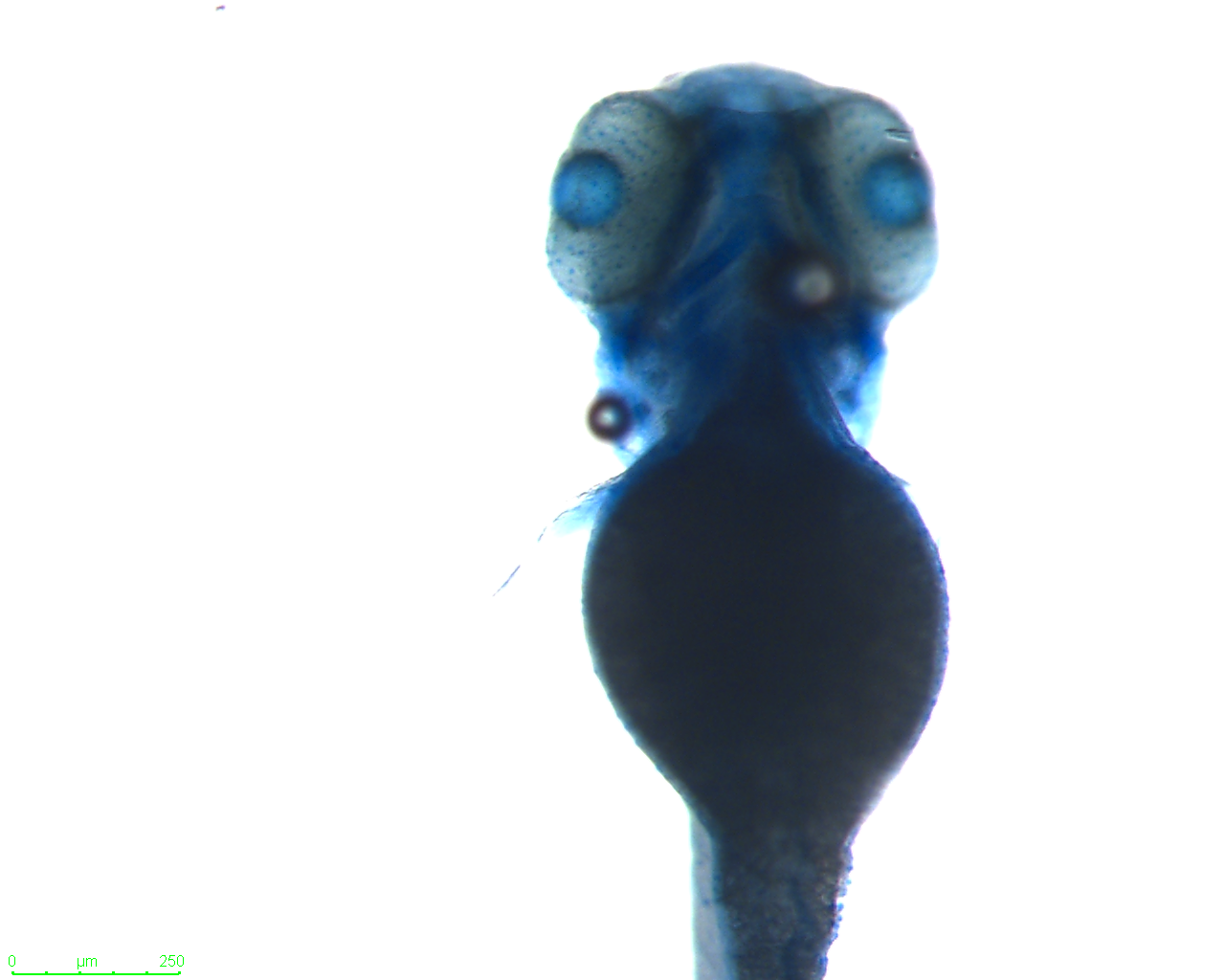

Supplement: Supplementary file 6 — Source Data [file 41467_2021_21053_MOESM6_ESM.zip › Source Data/Zebrafish Morpholino work/Second replicate/EIF5A expt 2Rs_Control_sperm 2.2.tif]

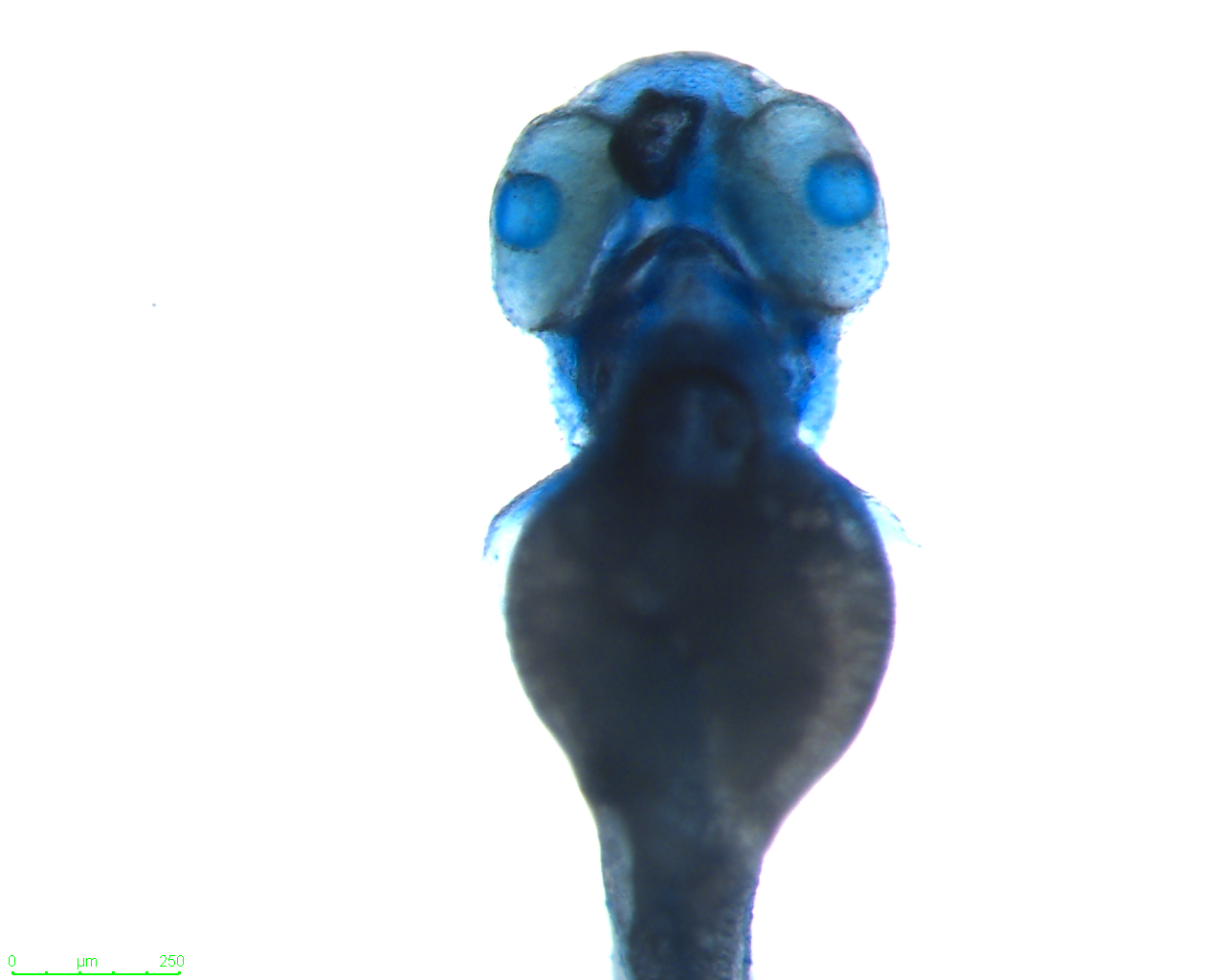

Supplement: Supplementary file 6 — Source Data [file 41467_2021_21053_MOESM6_ESM.zip › Source Data/Zebrafish Morpholino work/Second replicate/EIF5A expt 2Rs_Control_sperm 2.3.tif]

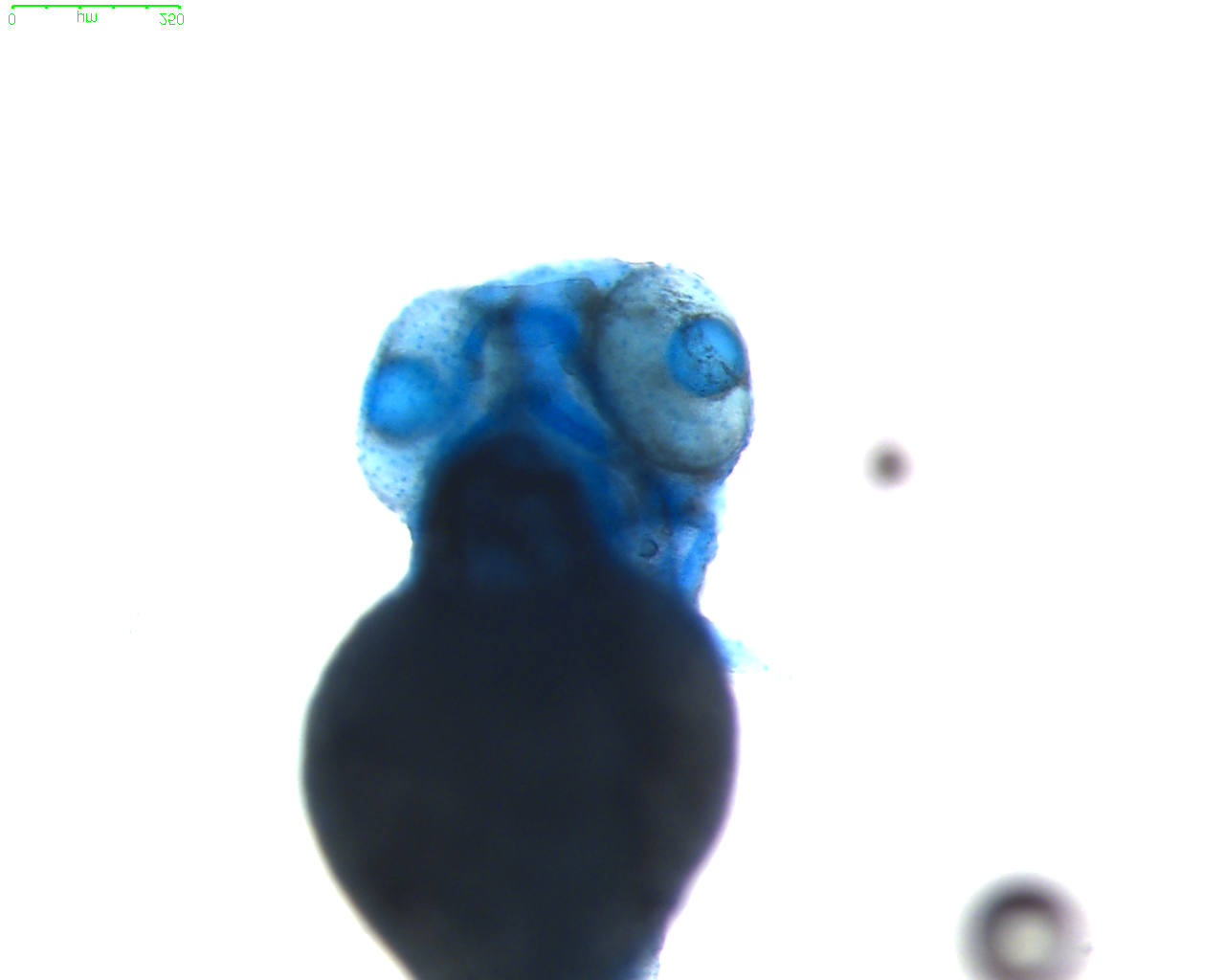

Supplement: Supplementary file 6 — Source Data [file 41467_2021_21053_MOESM6_ESM.zip › Source Data/Zebrafish Morpholino work/Second replicate/EIF5A expt 2Rs_Control_sperm 2.4.tif]

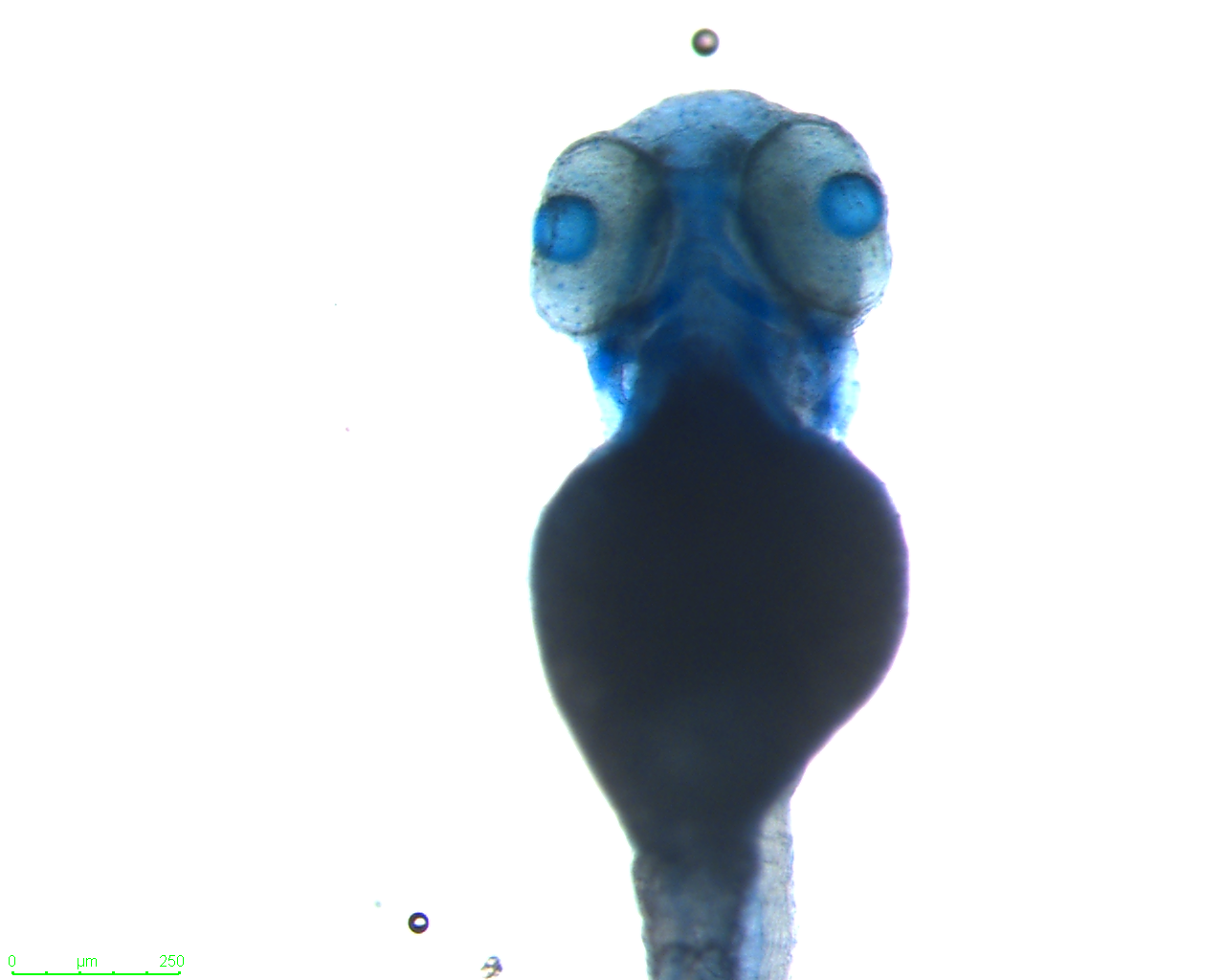

Supplement: Supplementary file 6 — Source Data [file 41467_2021_21053_MOESM6_ESM.zip › Source Data/Zebrafish Morpholino work/Second replicate/EIF5A expt 2Rs_Control_sperm 2.5.tif]

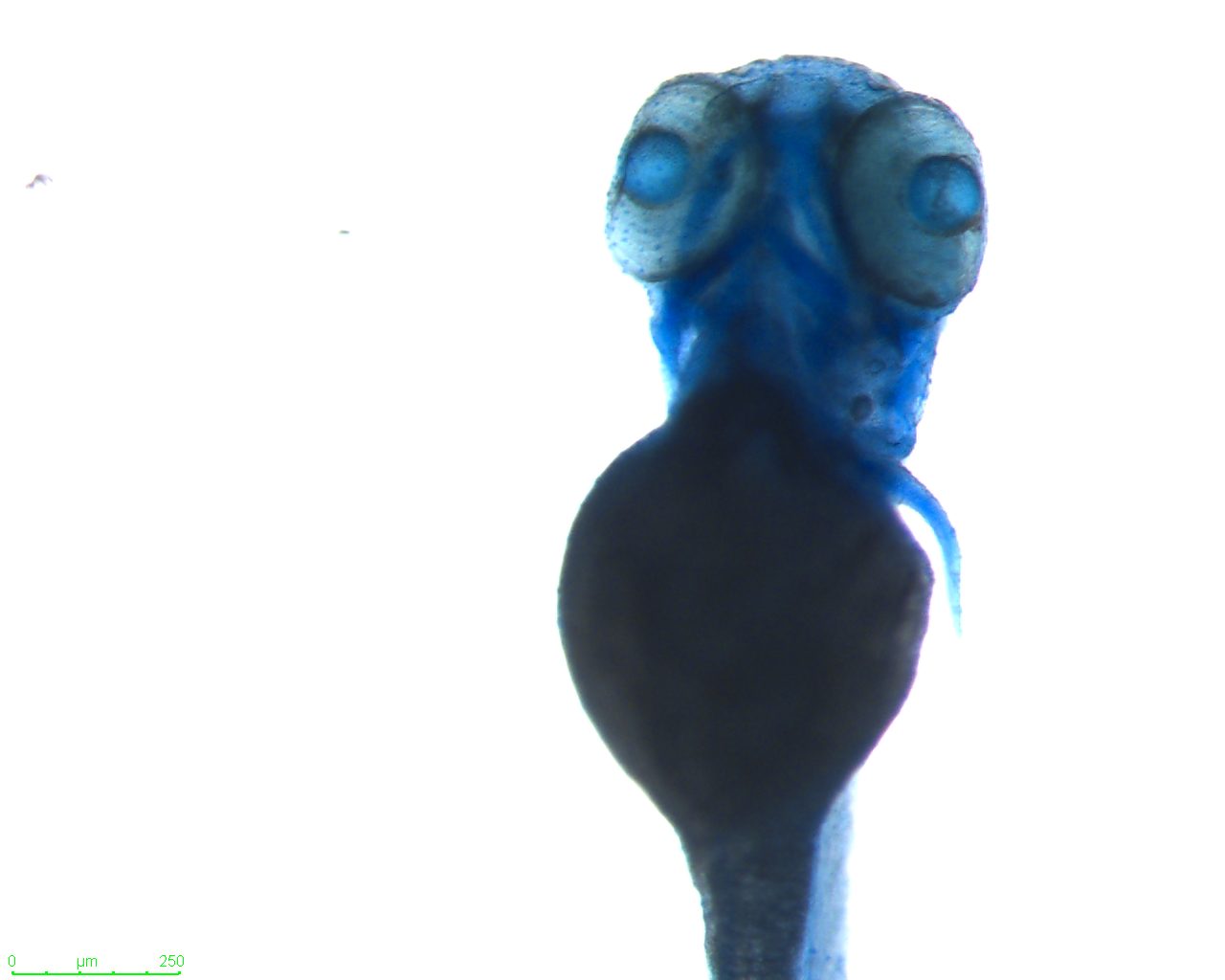

Supplement: Supplementary file 6 — Source Data [file 41467_2021_21053_MOESM6_ESM.zip › Source Data/Zebrafish Morpholino work/Second replicate/EIF5A expt 2Rs_Control_sperm 2.6.tif]

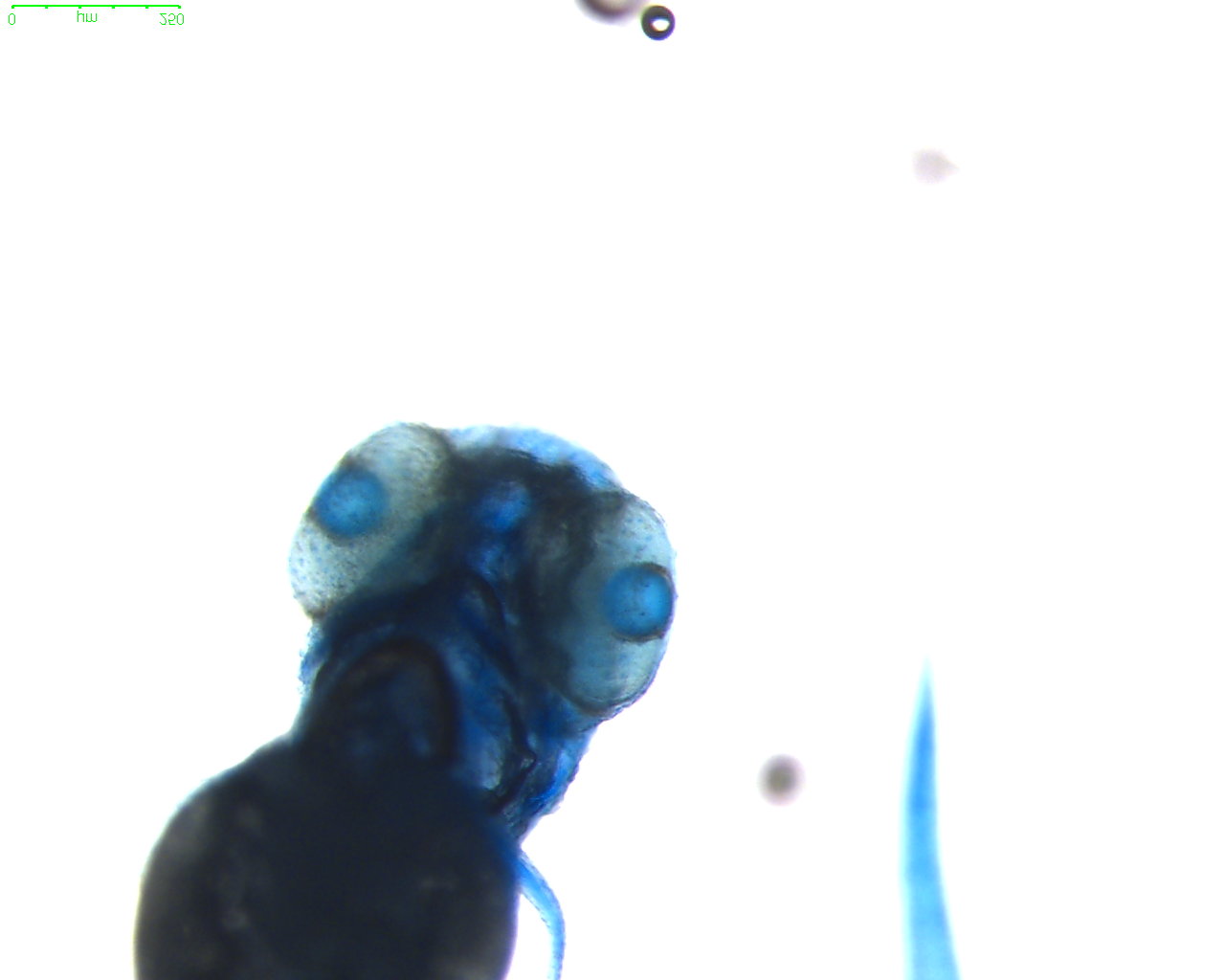

Supplement: Supplementary file 6 — Source Data [file 41467_2021_21053_MOESM6_ESM.zip › Source Data/Zebrafish Morpholino work/Second replicate/EIF5A expt 2Rs_Control_UNT 1.1.tif]

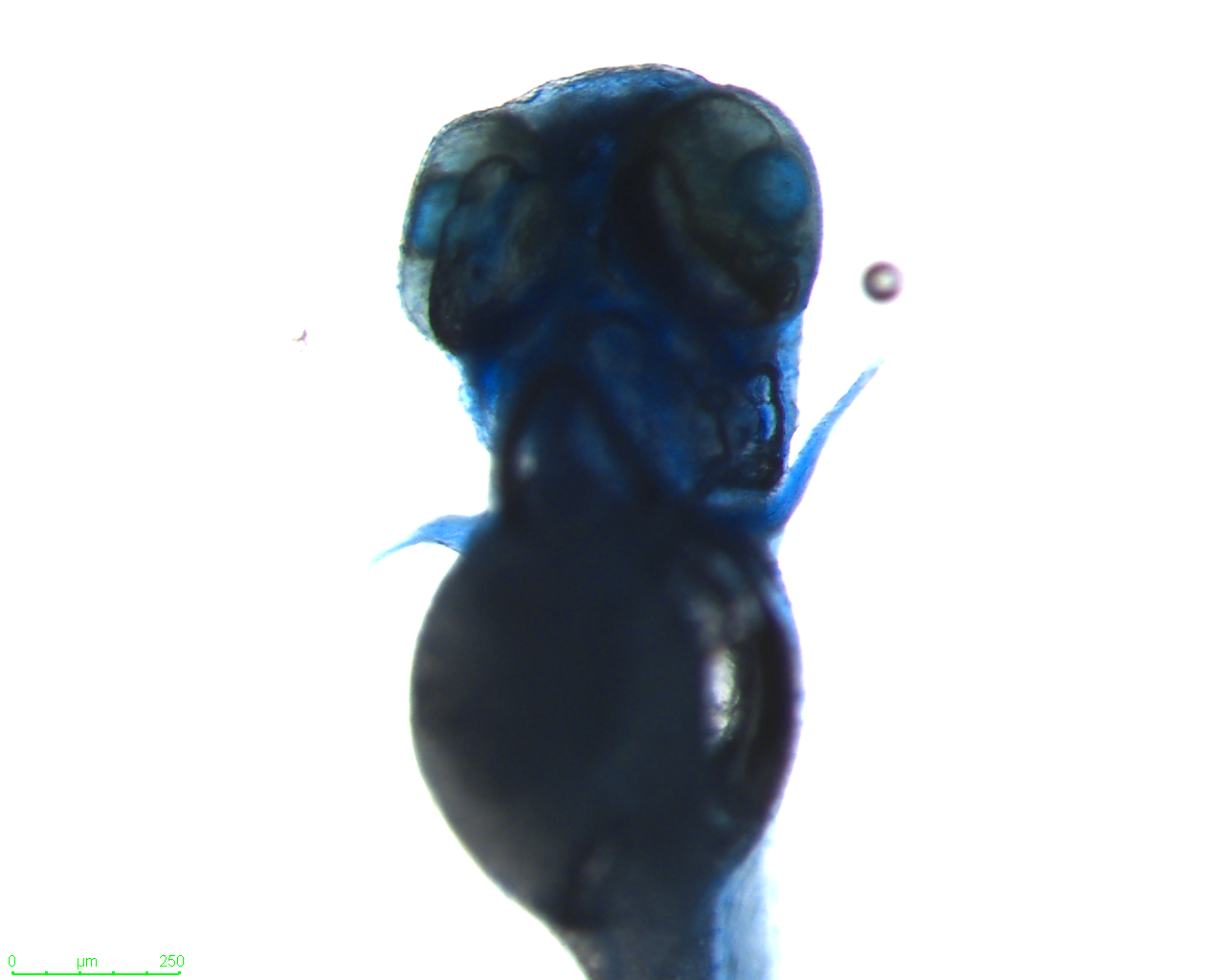

Supplement: Supplementary file 6 — Source Data [file 41467_2021_21053_MOESM6_ESM.zip › Source Data/Zebrafish Morpholino work/Second replicate/EIF5A expt 2Rs_Control_UNT 1.2.tif]

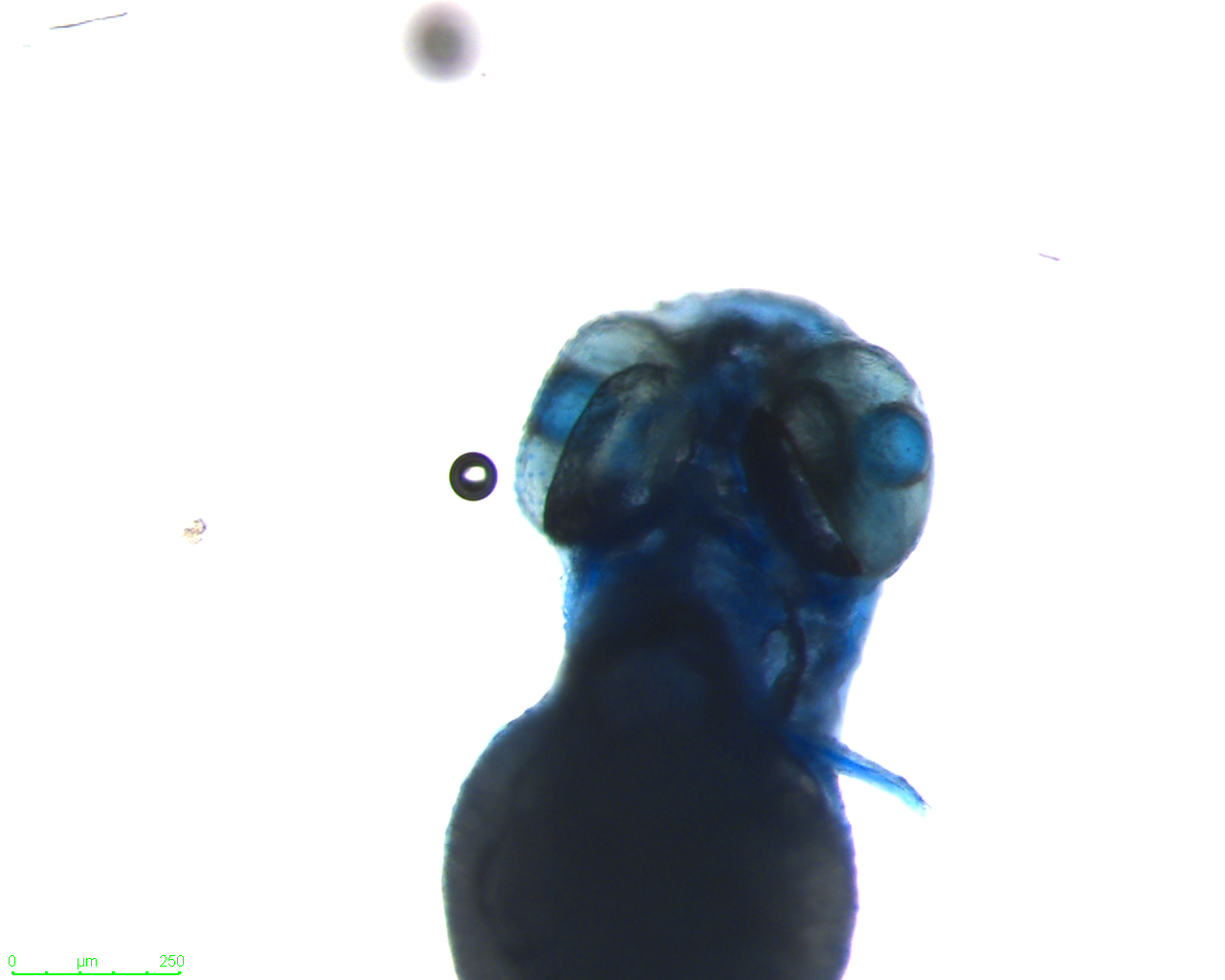

Supplement: Supplementary file 6 — Source Data [file 41467_2021_21053_MOESM6_ESM.zip › Source Data/Zebrafish Morpholino work/Second replicate/EIF5A expt 2Rs_Control_UNT 1.3.tif]

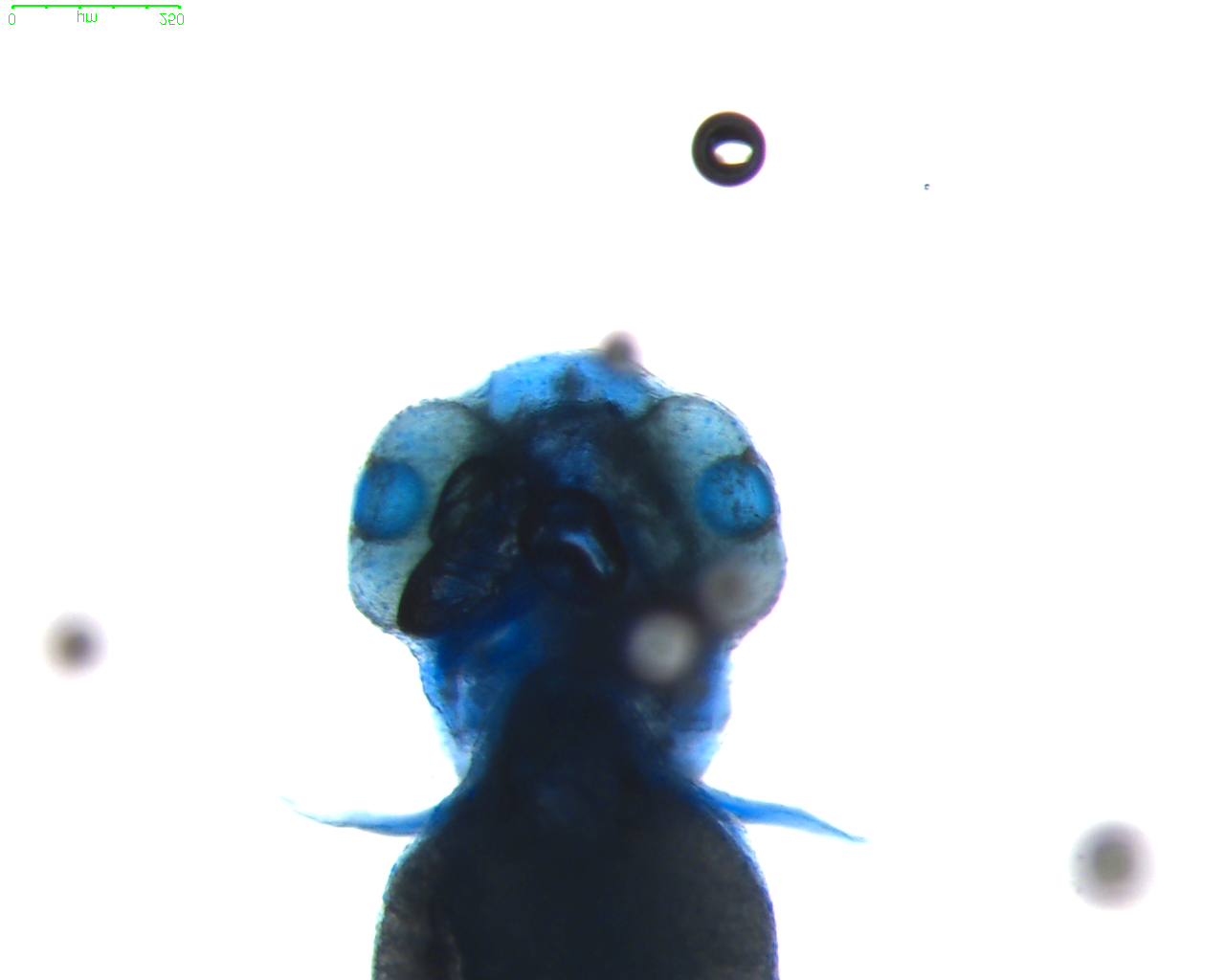

Supplement: Supplementary file 6 — Source Data [file 41467_2021_21053_MOESM6_ESM.zip › Source Data/Zebrafish Morpholino work/Second replicate/EIF5A expt 2Rs_Control_UNT 1.4.tif]

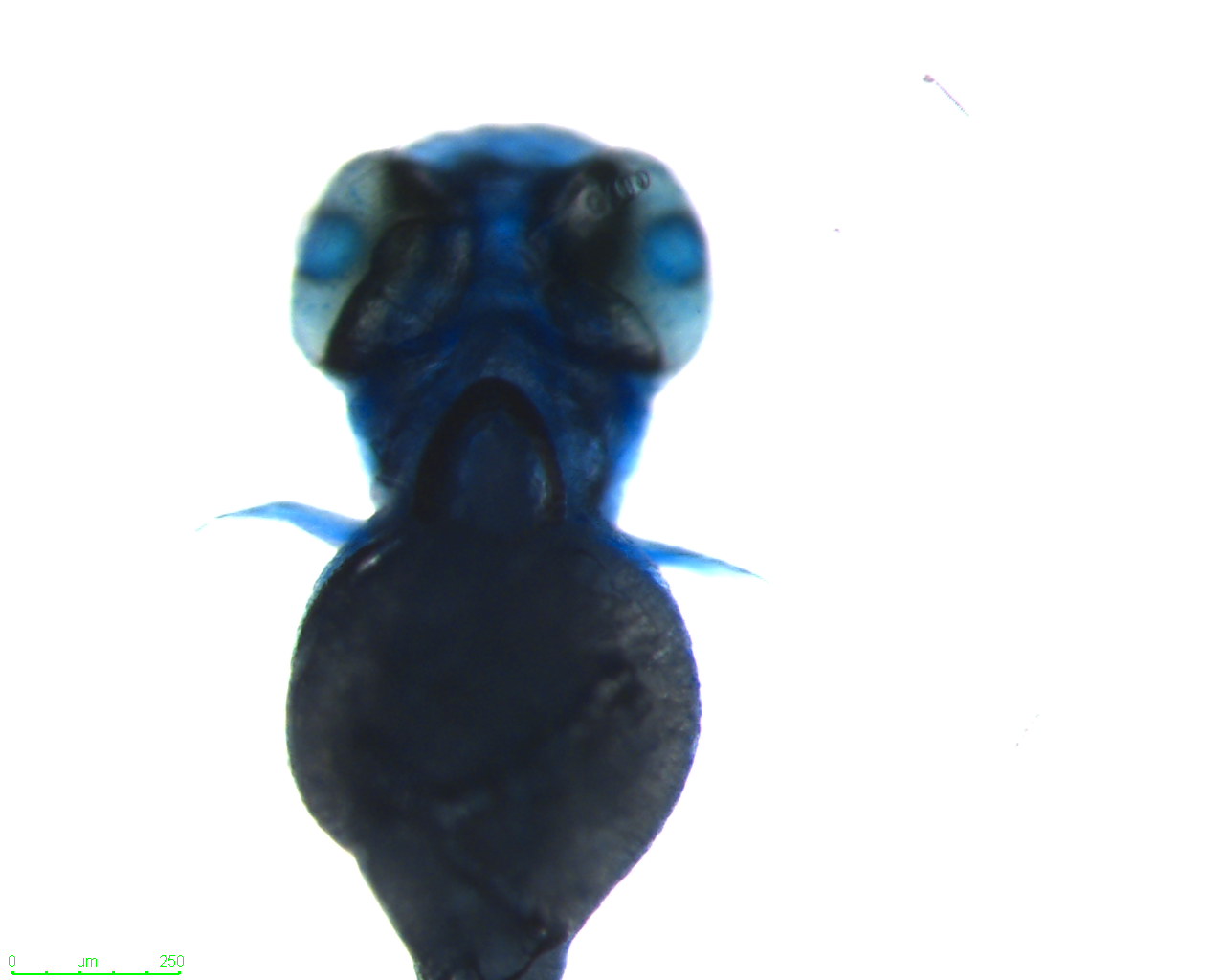

Supplement: Supplementary file 6 — Source Data [file 41467_2021_21053_MOESM6_ESM.zip › Source Data/Zebrafish Morpholino work/Second replicate/EIF5A expt 2Rs_Control_UNT 1.5.tif]

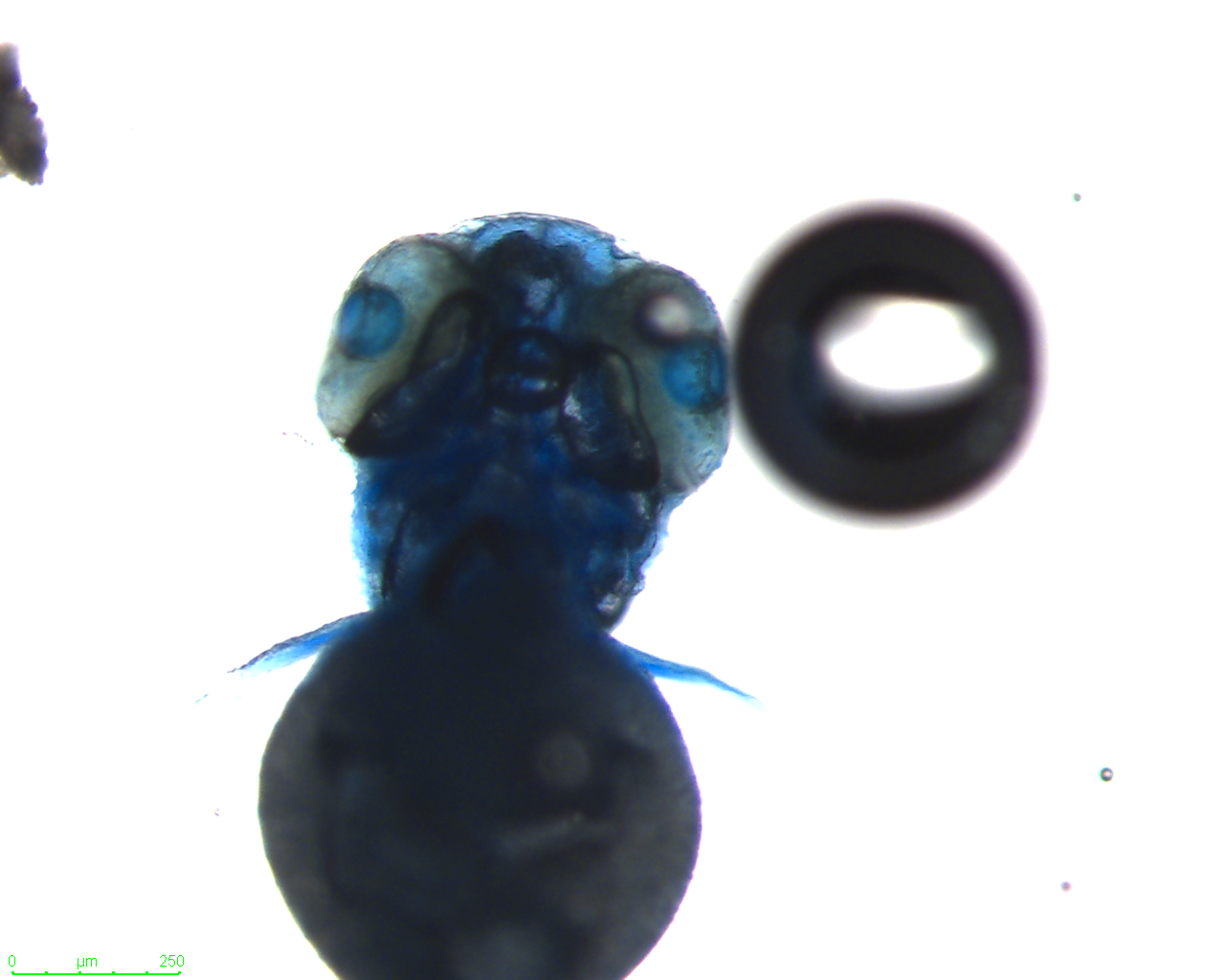

Supplement: Supplementary file 6 — Source Data [file 41467_2021_21053_MOESM6_ESM.zip › Source Data/Zebrafish Morpholino work/Second replicate/EIF5A expt 2Rs_Control_UNT 1.6.tif]

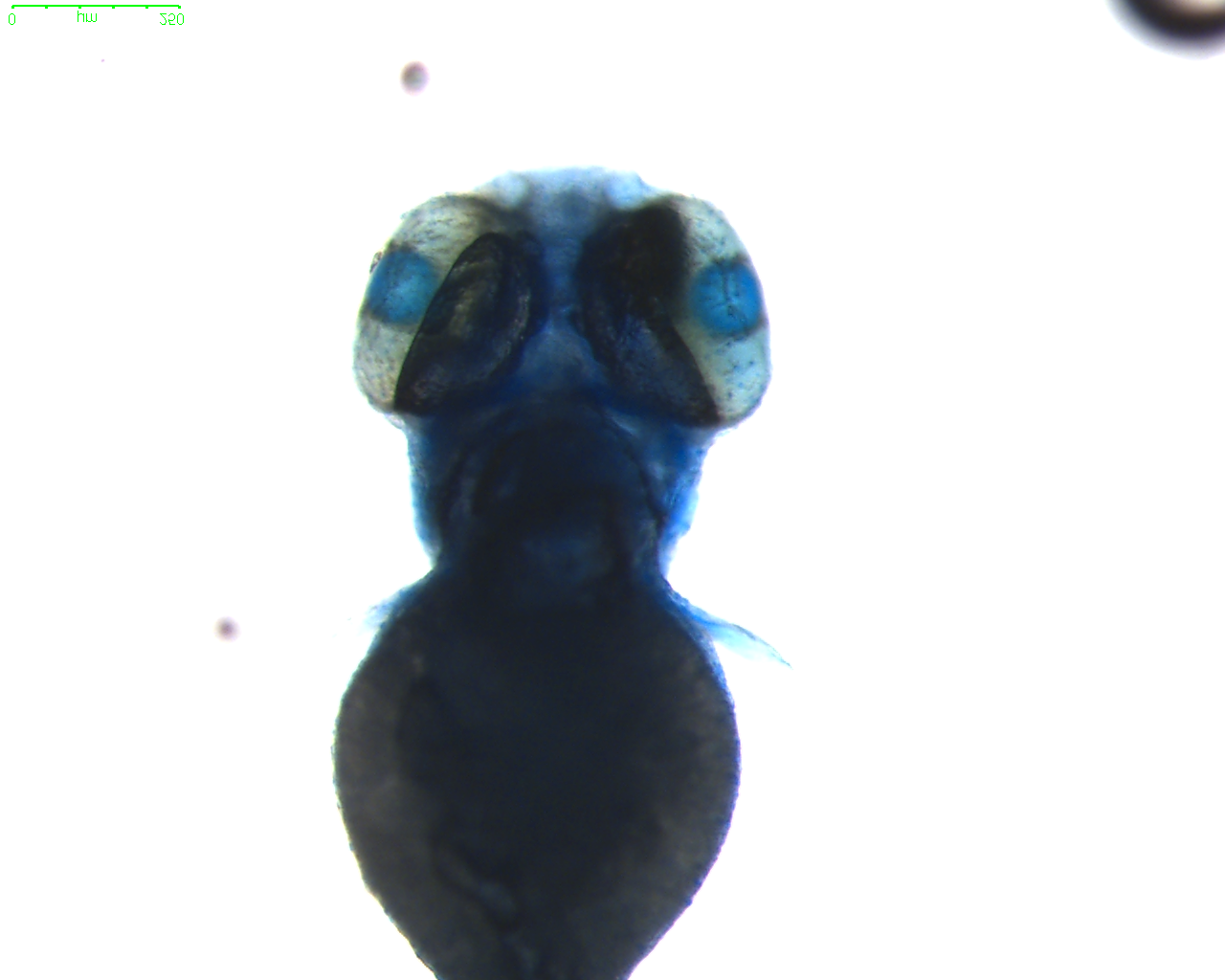

Supplement: Supplementary file 6 — Source Data [file 41467_2021_21053_MOESM6_ESM.zip › Source Data/Zebrafish Morpholino work/Second replicate/EIF5A expt 2Rs_Control_UNT 1.7.tif]

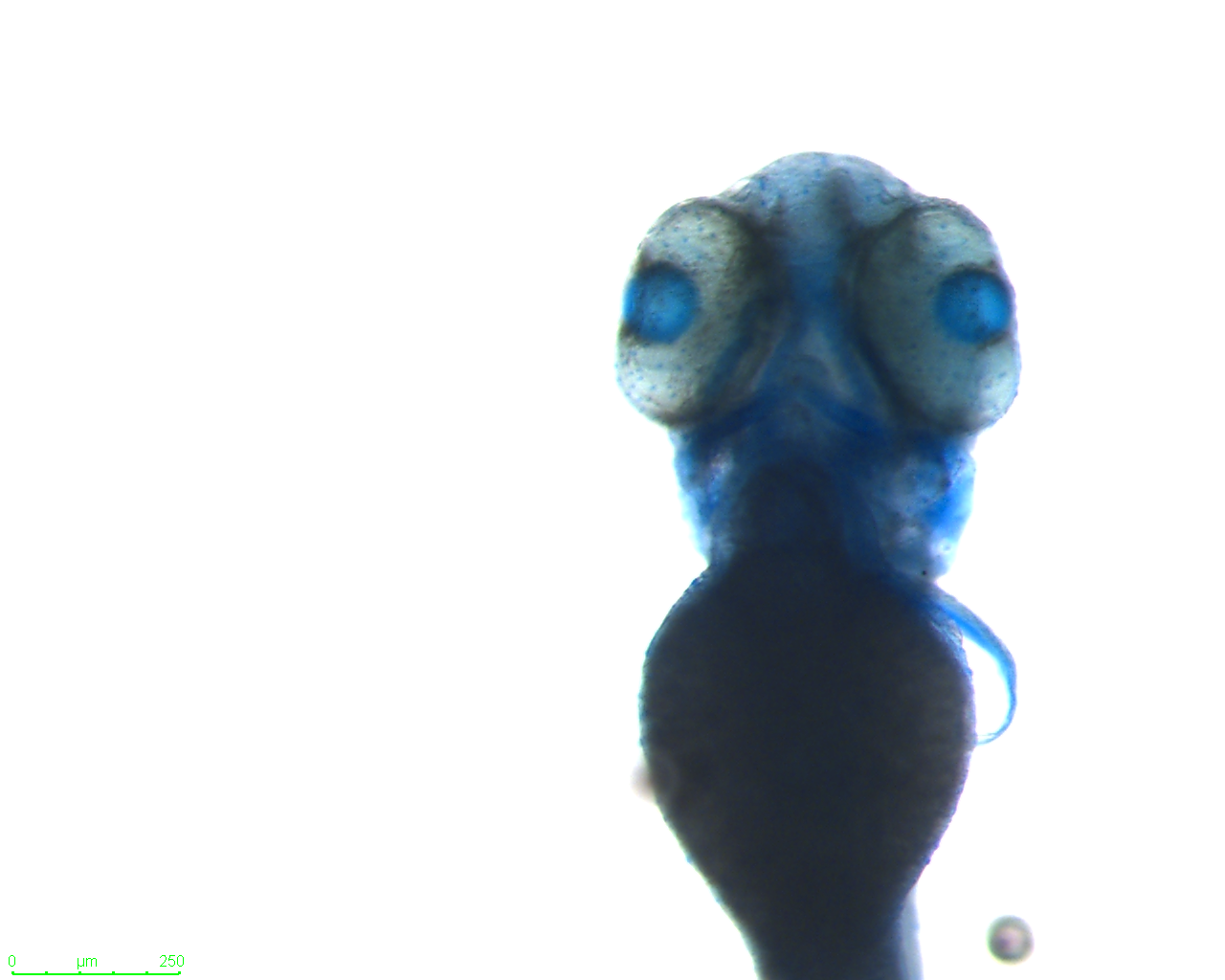

Supplement: Supplementary file 6 — Source Data [file 41467_2021_21053_MOESM6_ESM.zip › Source Data/Zebrafish Morpholino work/Second replicate/EIF5A expt 2Rs_Control_unt 2.1.tif]

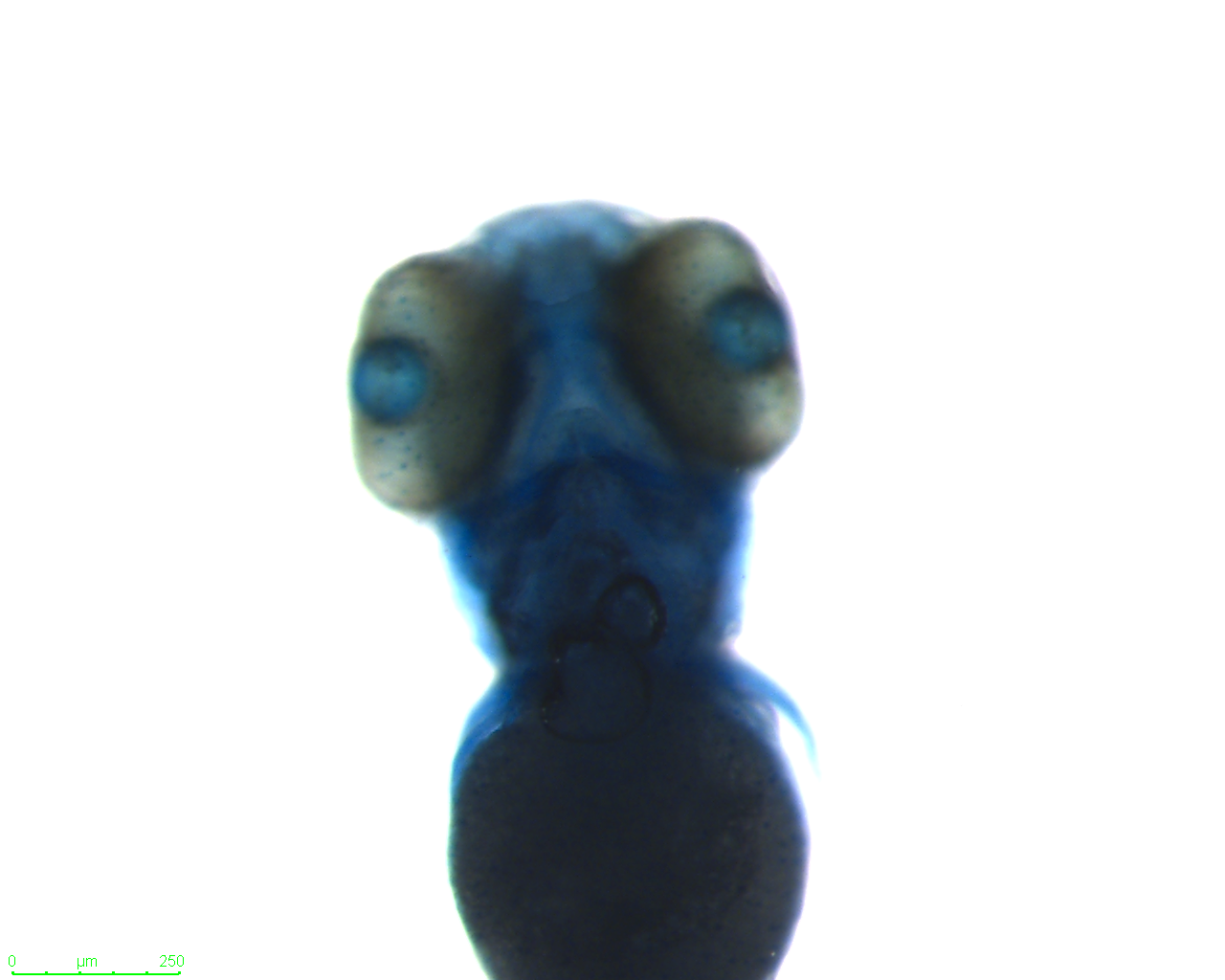

Supplement: Supplementary file 6 — Source Data [file 41467_2021_21053_MOESM6_ESM.zip › Source Data/Zebrafish Morpholino work/Second replicate/EIF5A expt 2Rs_Control_unt 2.2.tif]

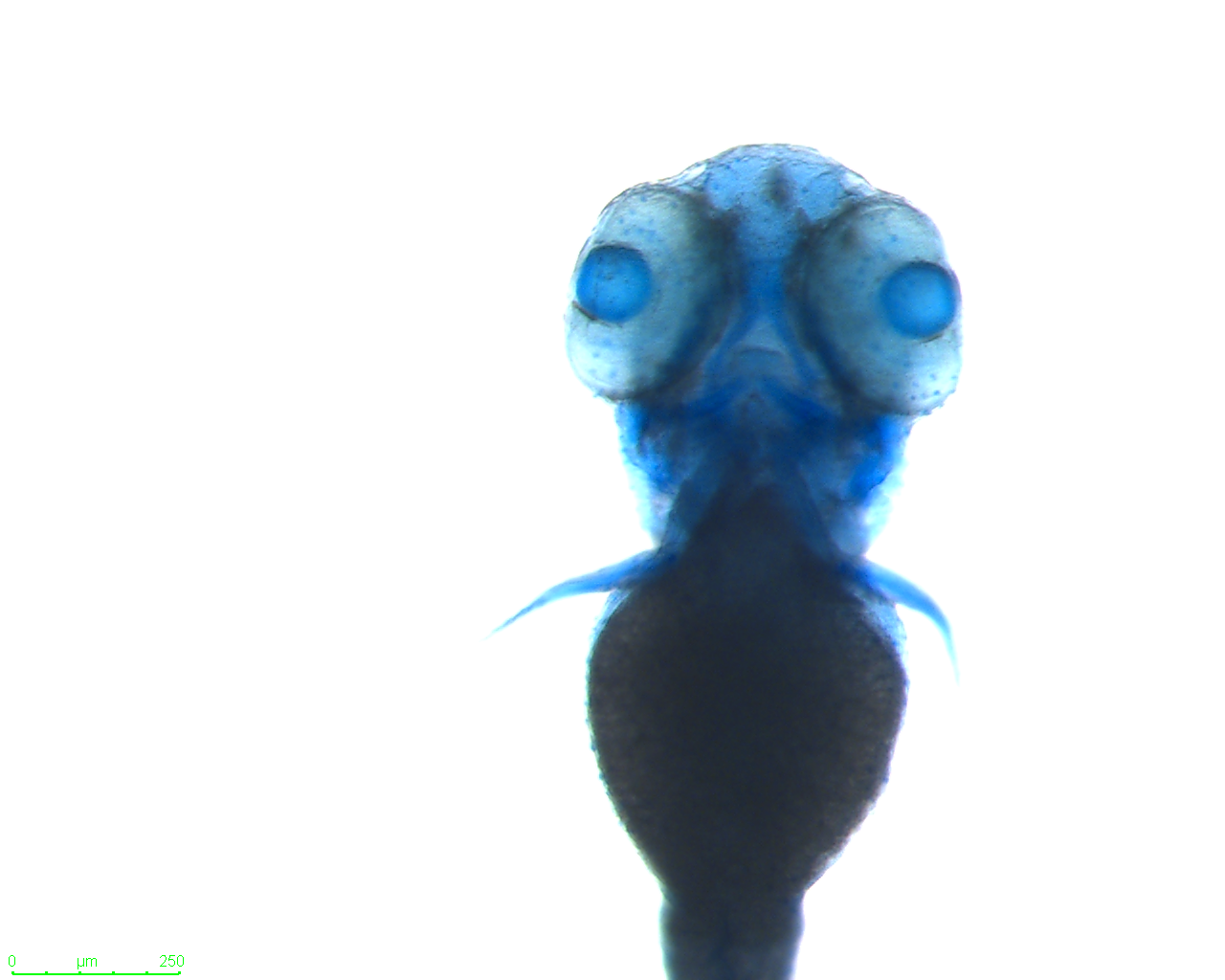

Supplement: Supplementary file 6 — Source Data [file 41467_2021_21053_MOESM6_ESM.zip › Source Data/Zebrafish Morpholino work/Second replicate/EIF5A expt 2Rs_Control_unt 2.3.tif]

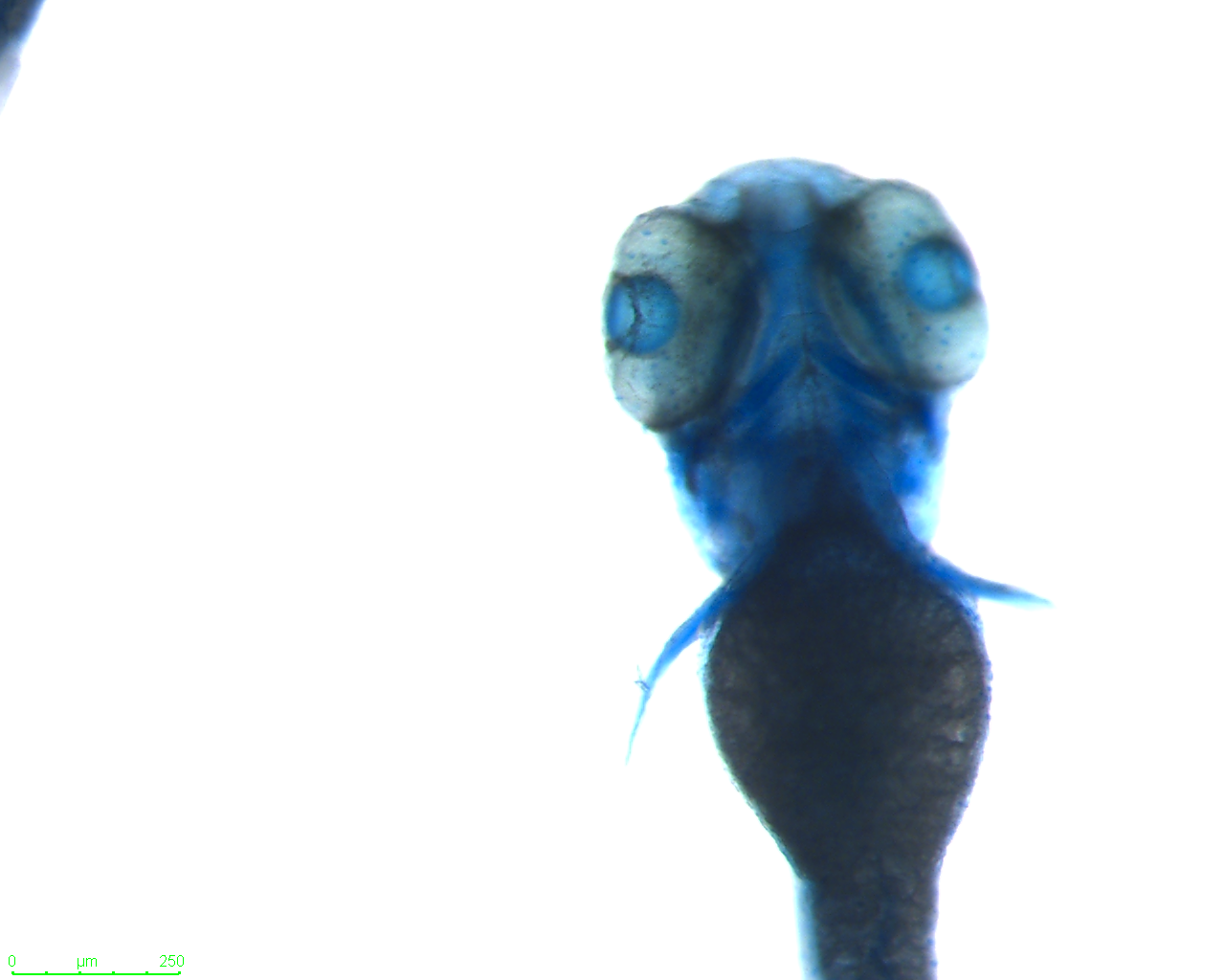

Supplement: Supplementary file 6 — Source Data [file 41467_2021_21053_MOESM6_ESM.zip › Source Data/Zebrafish Morpholino work/Second replicate/EIF5A expt 2Rs_Control_unt 2.4.tif]

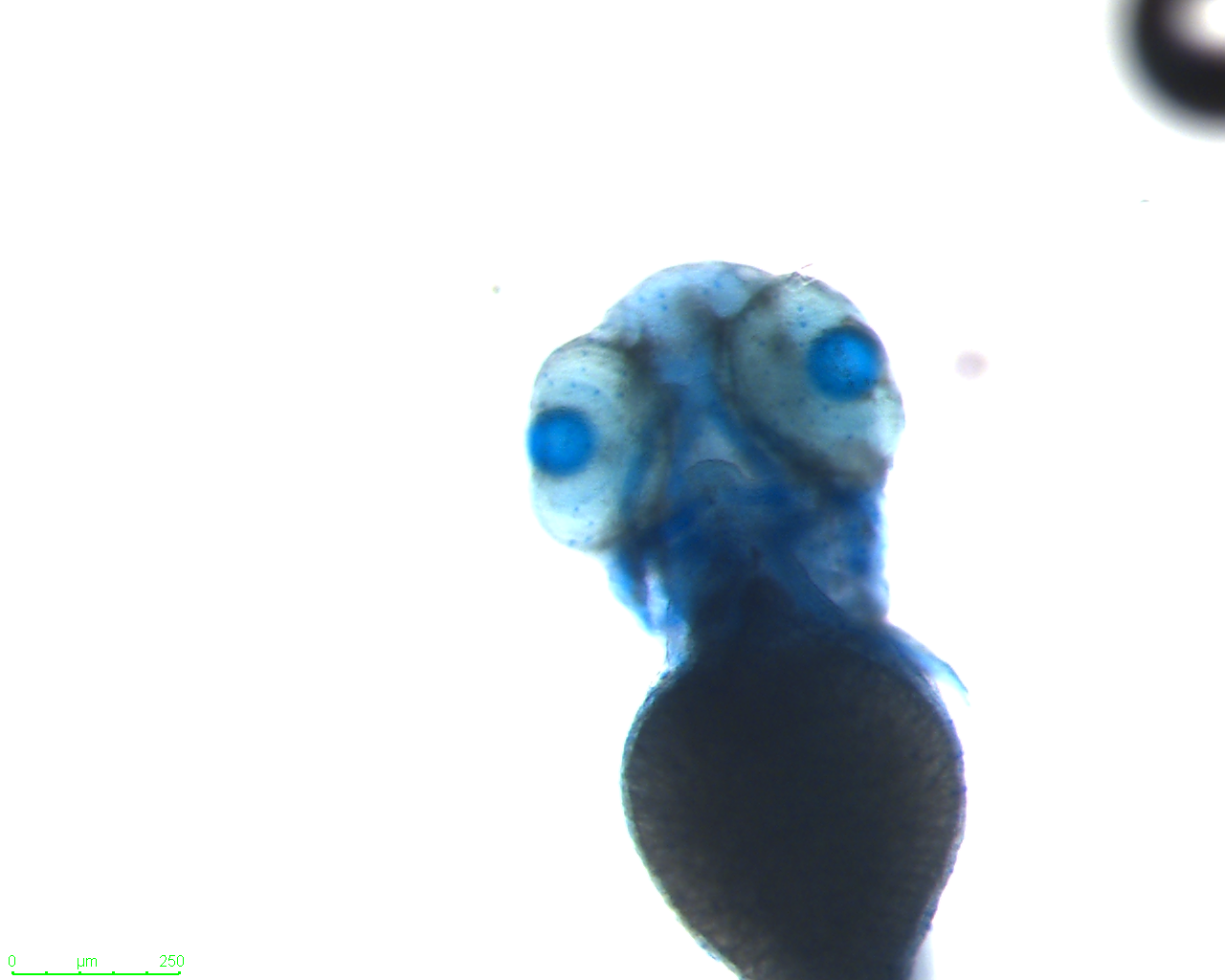

Supplement: Supplementary file 6 — Source Data [file 41467_2021_21053_MOESM6_ESM.zip › Source Data/Zebrafish Morpholino work/Second replicate/EIF5A expt 2Rs_Control_unt 2.5.tif]

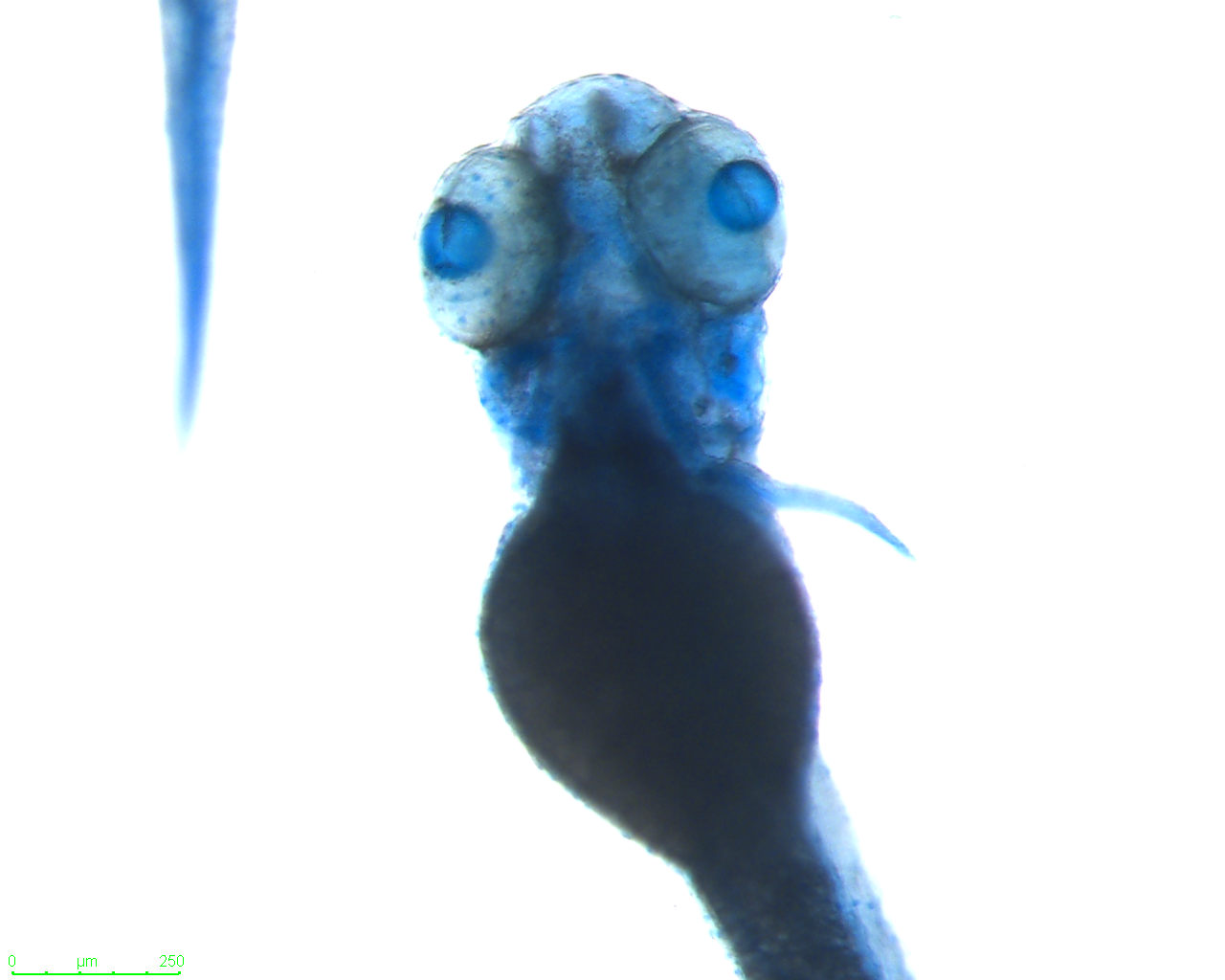

Supplement: Supplementary file 6 — Source Data [file 41467_2021_21053_MOESM6_ESM.zip › Source Data/Zebrafish Morpholino work/Second replicate/EIF5A expt 2Rs_Control_unt 2.6.tif]

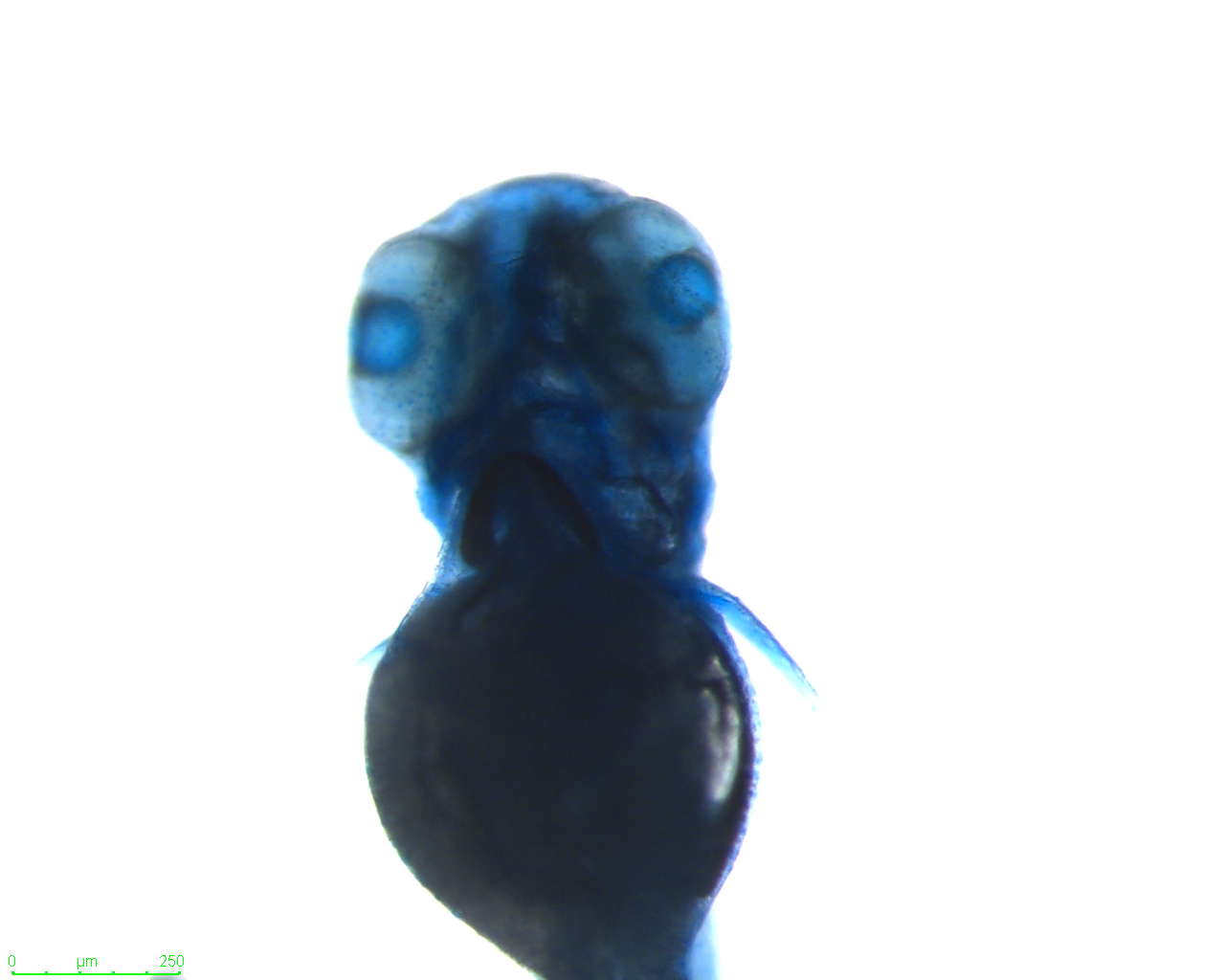

Supplement: Supplementary file 6 — Source Data [file 41467_2021_21053_MOESM6_ESM.zip › Source Data/Zebrafish Morpholino work/Second replicate/EIF5A expt 2Rs_EIF5A_sperm 1.1.tif]

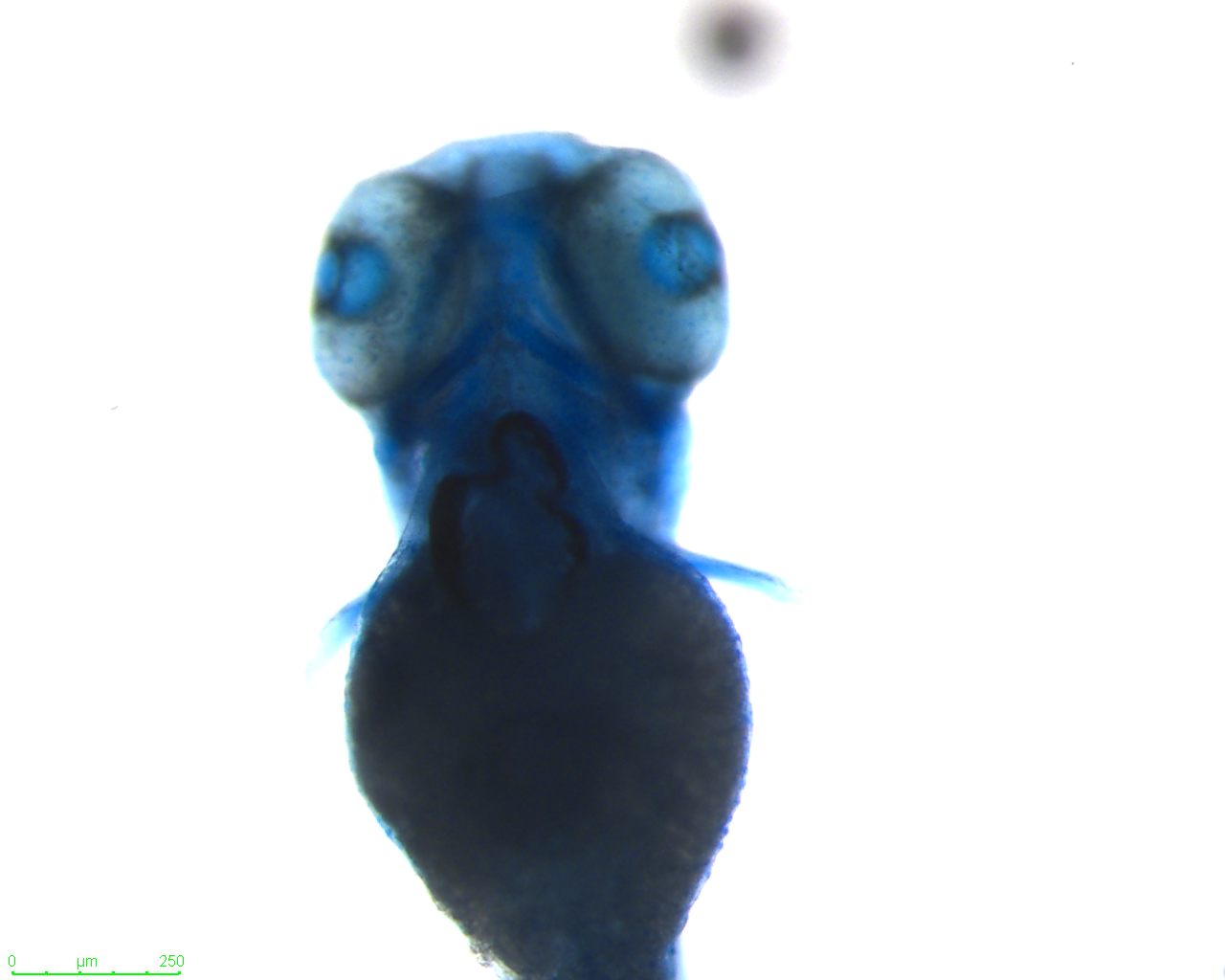

Supplement: Supplementary file 6 — Source Data [file 41467_2021_21053_MOESM6_ESM.zip › Source Data/Zebrafish Morpholino work/Second replicate/EIF5A expt 2Rs_EIF5A_sperm 1.2.tif]

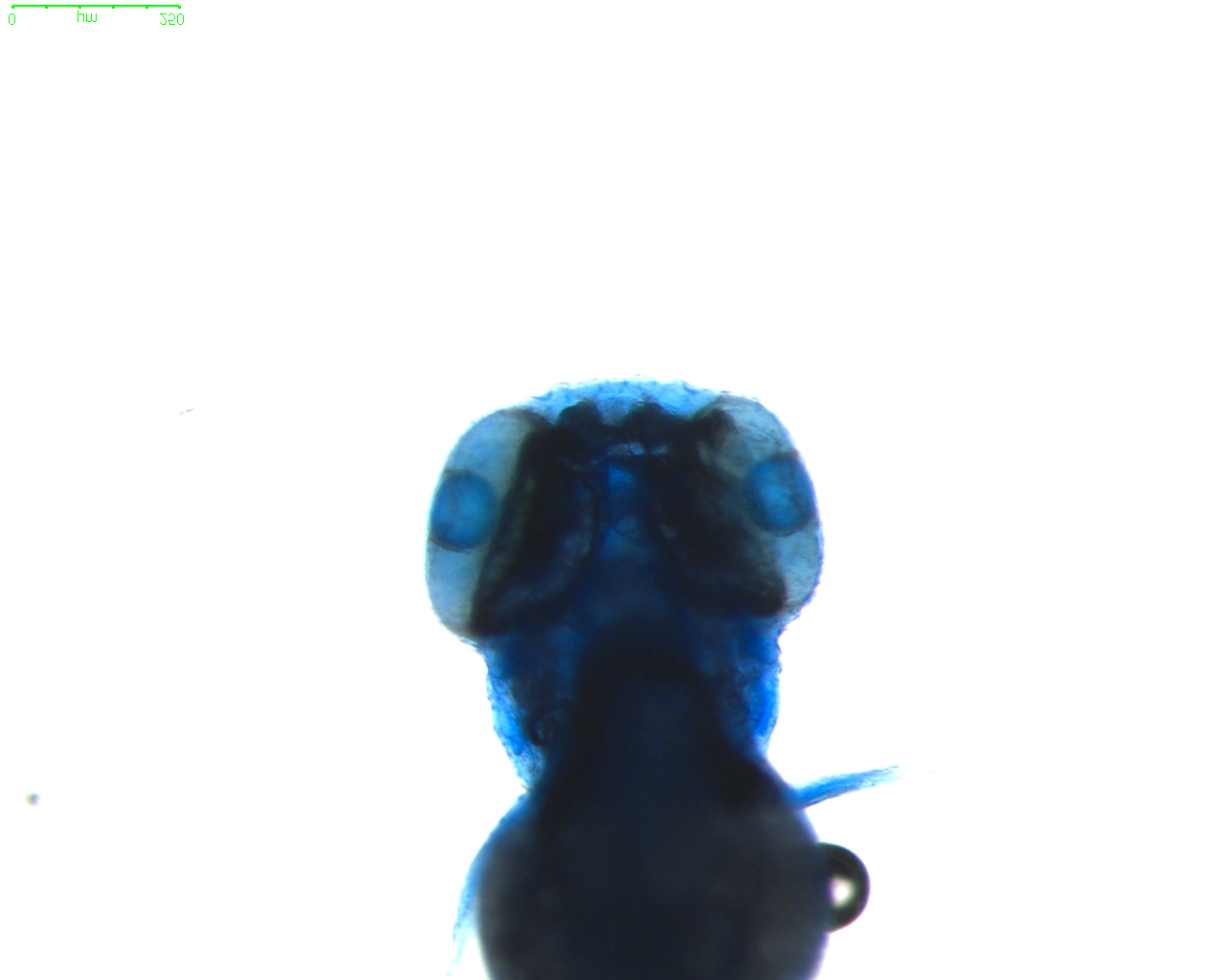

Supplement: Supplementary file 6 — Source Data [file 41467_2021_21053_MOESM6_ESM.zip › Source Data/Zebrafish Morpholino work/Second replicate/EIF5A expt 2Rs_EIF5A_sperm 1.3.tif]

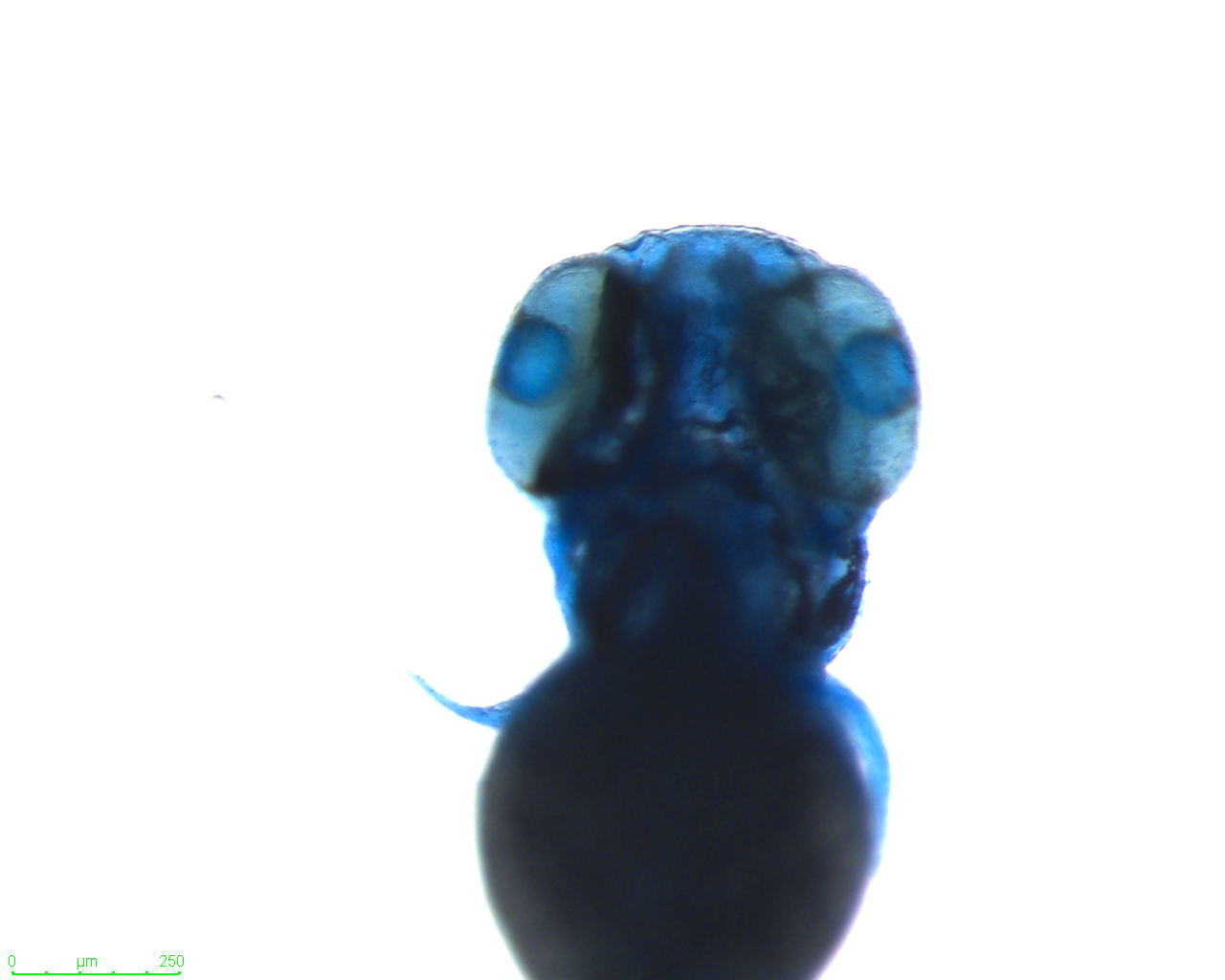

Supplement: Supplementary file 6 — Source Data [file 41467_2021_21053_MOESM6_ESM.zip › Source Data/Zebrafish Morpholino work/Second replicate/EIF5A expt 2Rs_EIF5A_sperm 1.4.tif]

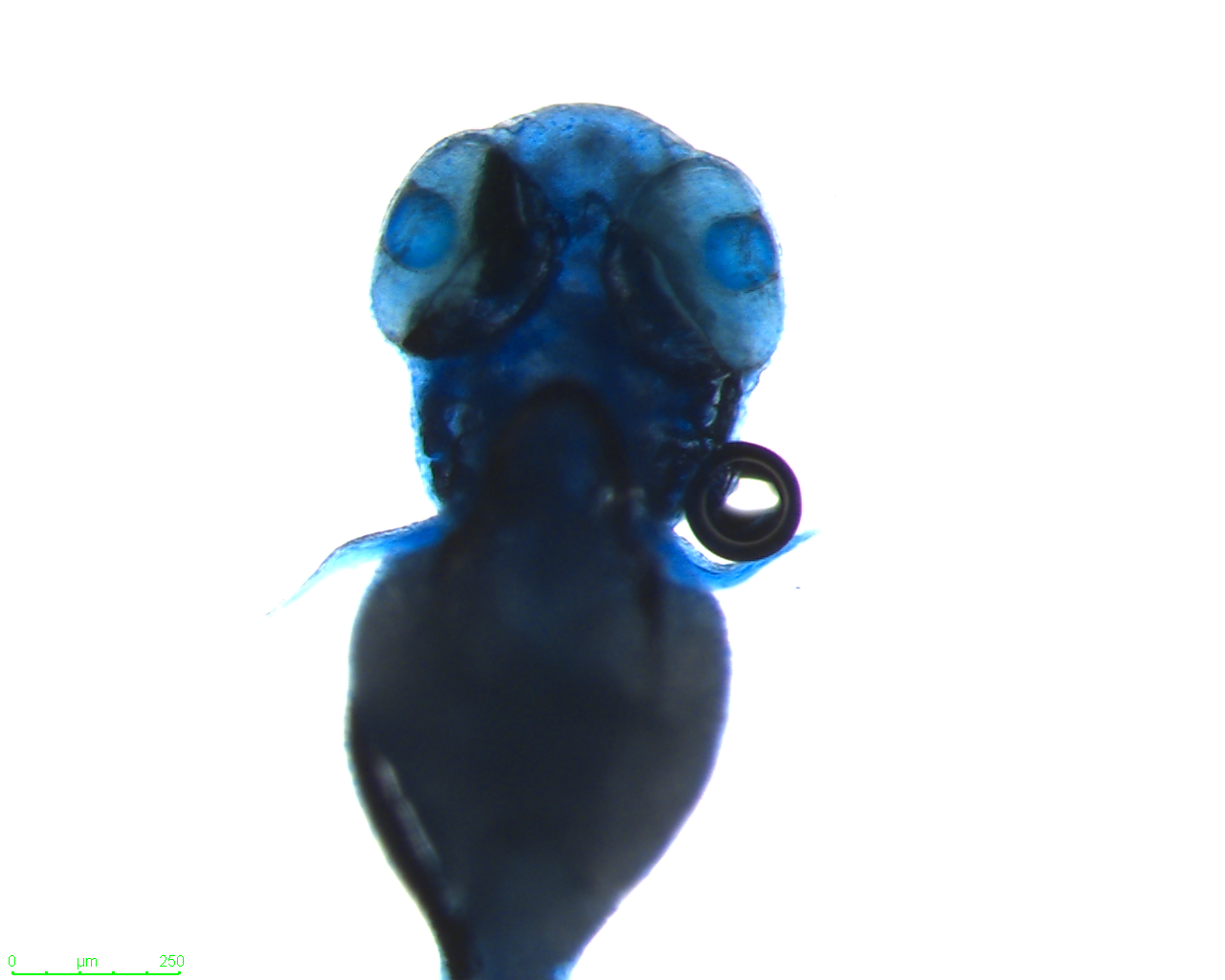

Supplement: Supplementary file 6 — Source Data [file 41467_2021_21053_MOESM6_ESM.zip › Source Data/Zebrafish Morpholino work/Second replicate/EIF5A expt 2Rs_EIF5A_sperm 1.5.tif]

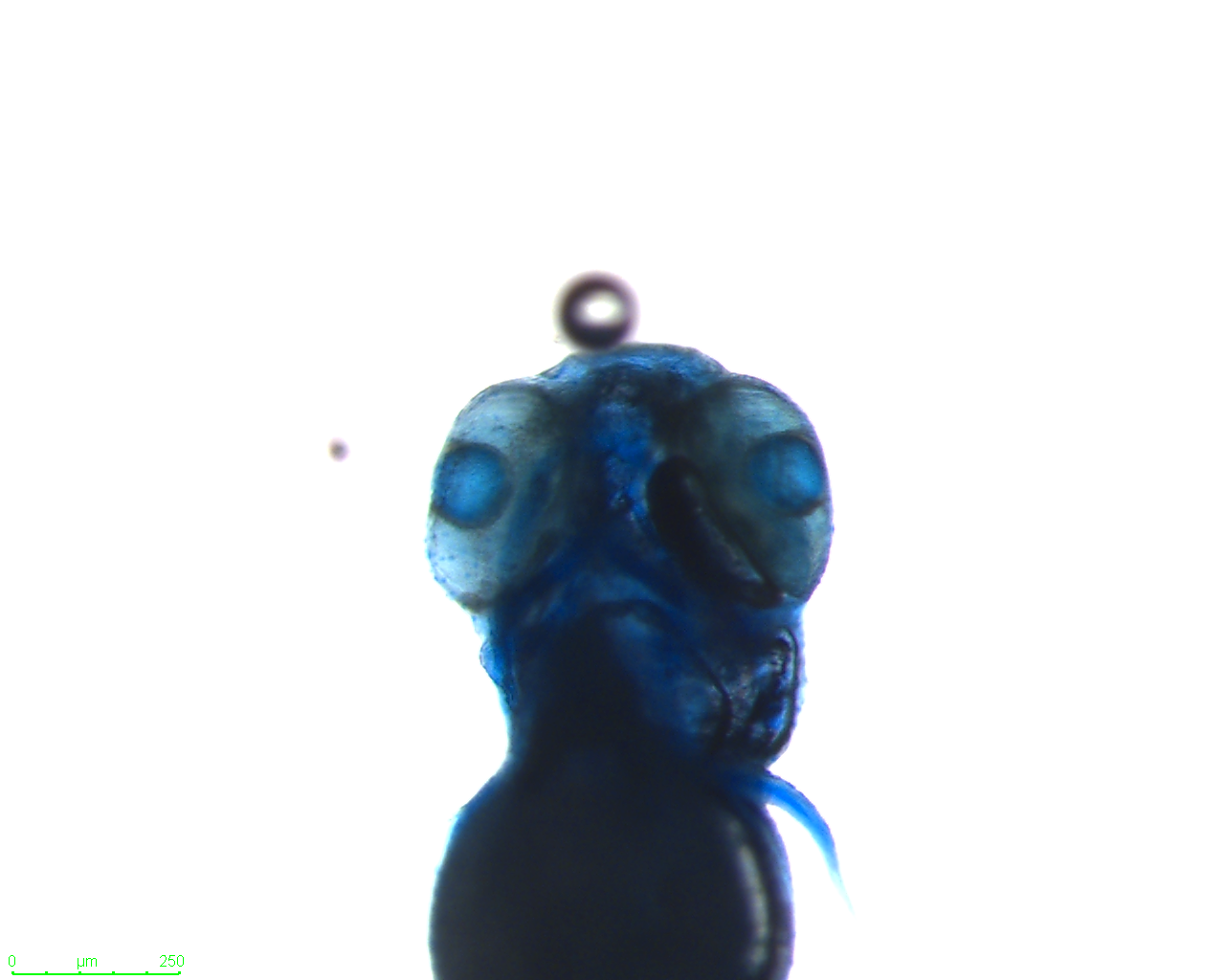

Supplement: Supplementary file 6 — Source Data [file 41467_2021_21053_MOESM6_ESM.zip › Source Data/Zebrafish Morpholino work/Second replicate/EIF5A expt 2Rs_EIF5A_sperm 1.6.tif]

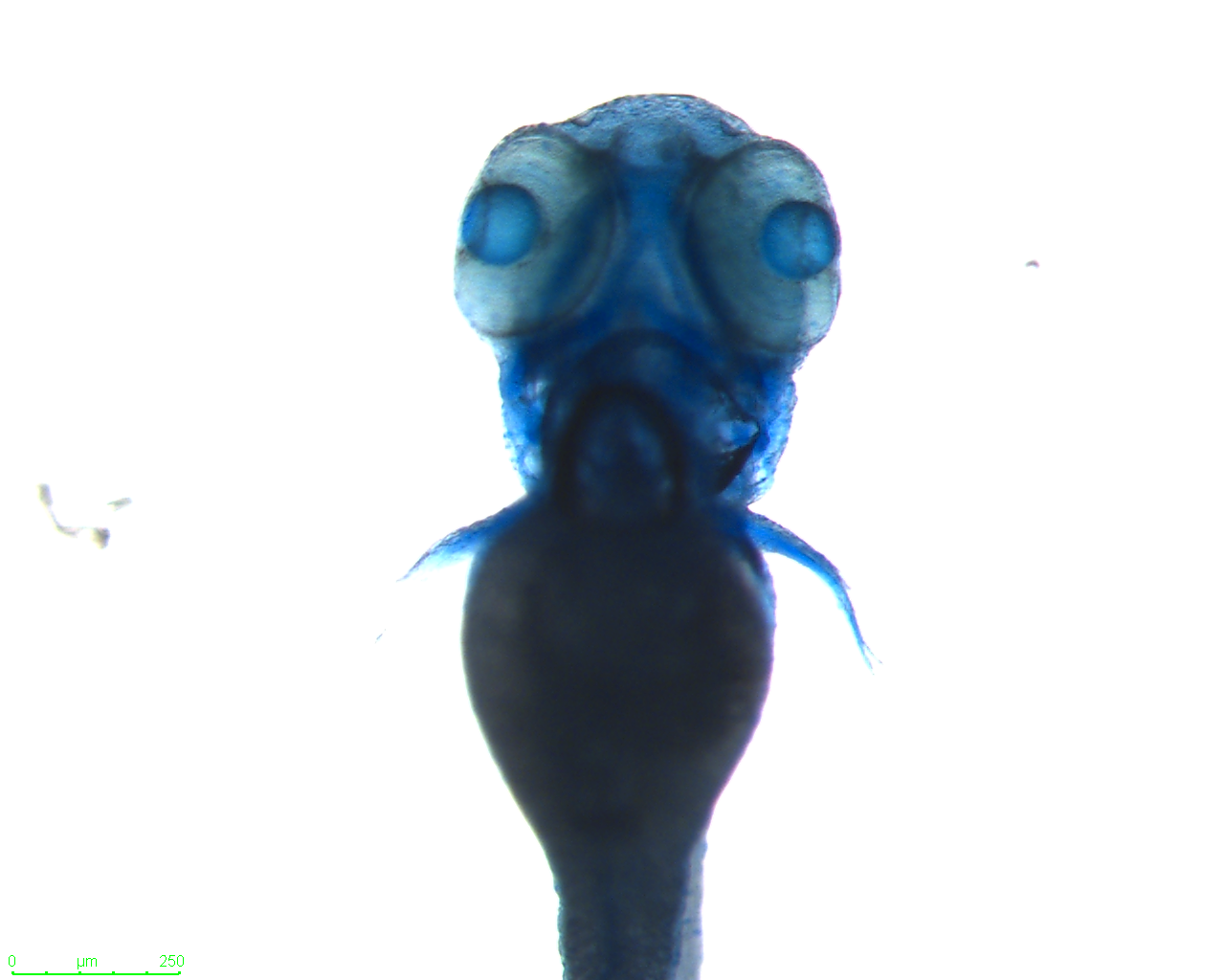

Supplement: Supplementary file 6 — Source Data [file 41467_2021_21053_MOESM6_ESM.zip › Source Data/Zebrafish Morpholino work/Second replicate/EIF5A expt 2Rs_EIF5A_sperm_2.1.tif]

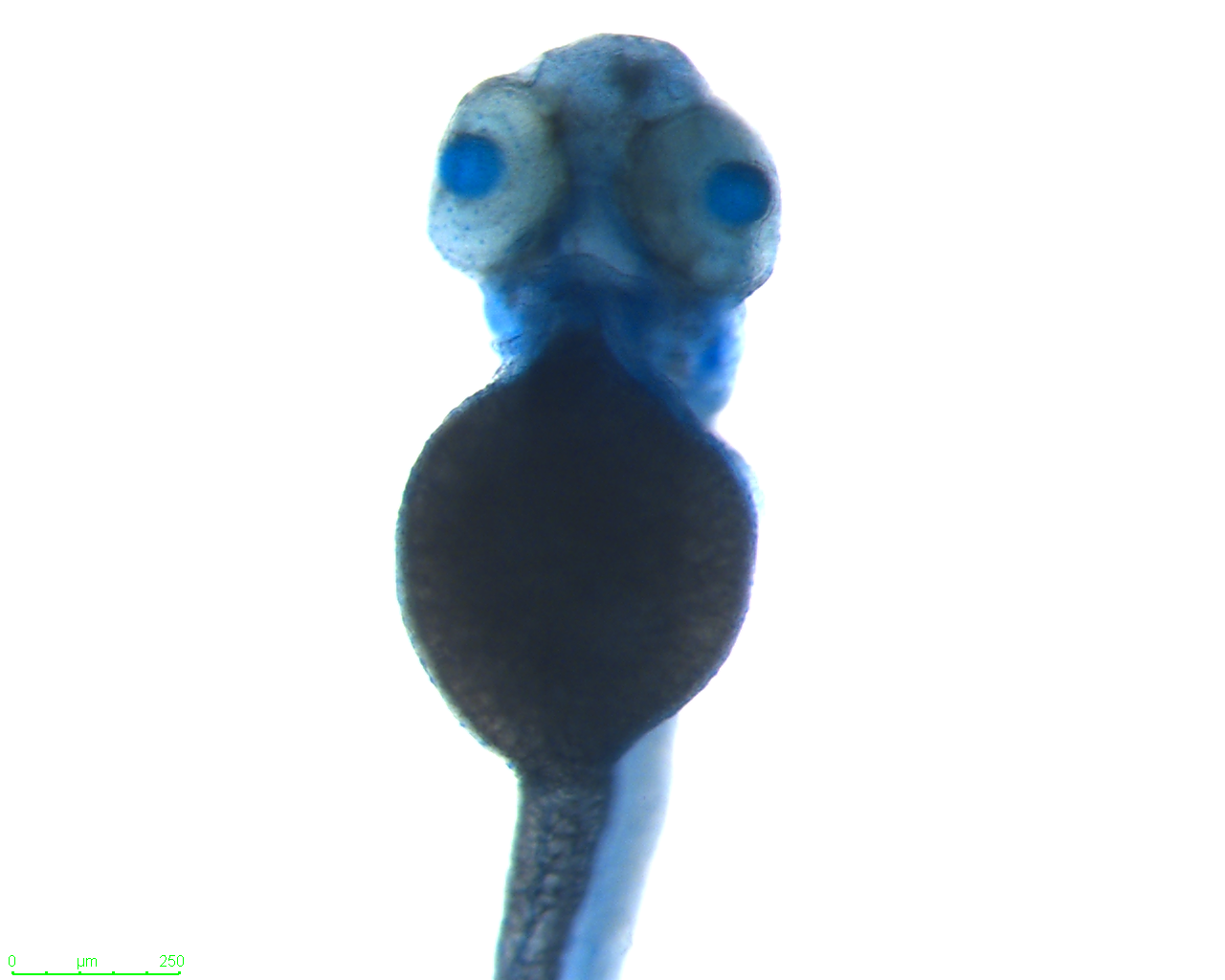

Supplement: Supplementary file 6 — Source Data [file 41467_2021_21053_MOESM6_ESM.zip › Source Data/Zebrafish Morpholino work/Second replicate/EIF5A expt 2Rs_EIF5A_sperm_2.2.tif]

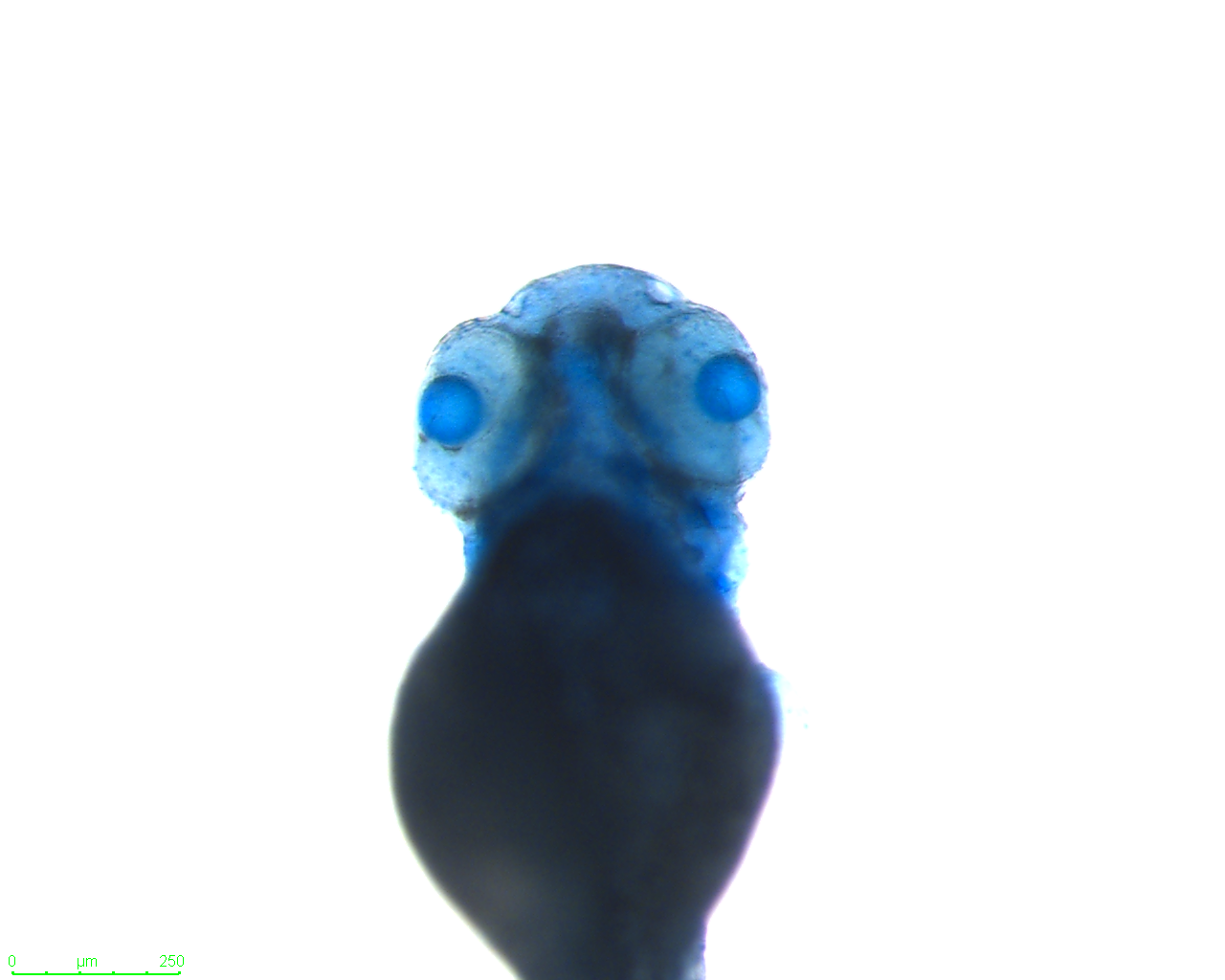

Supplement: Supplementary file 6 — Source Data [file 41467_2021_21053_MOESM6_ESM.zip › Source Data/Zebrafish Morpholino work/Second replicate/EIF5A expt 2Rs_EIF5A_sperm_2.3.tif]

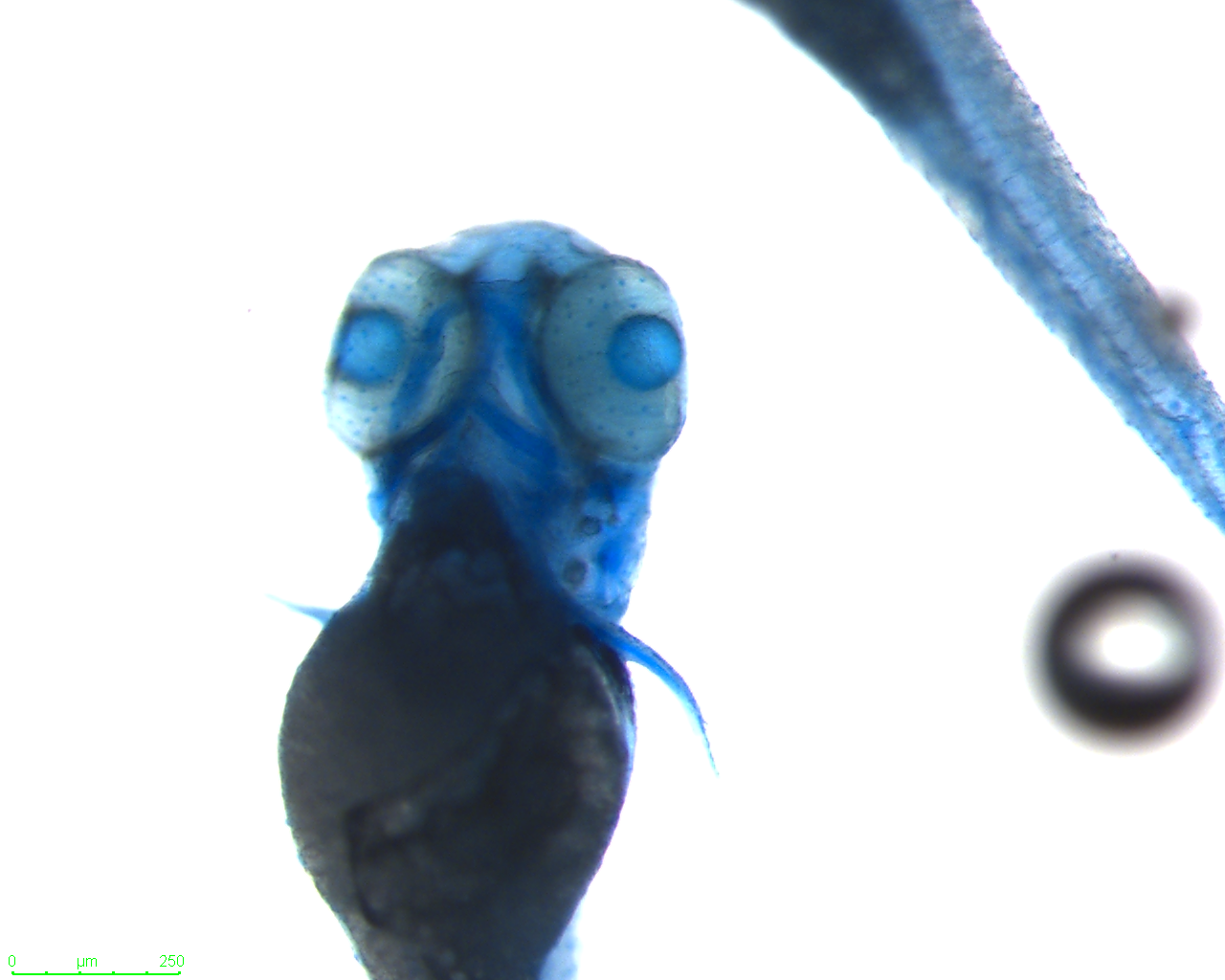

Supplement: Supplementary file 6 — Source Data [file 41467_2021_21053_MOESM6_ESM.zip › Source Data/Zebrafish Morpholino work/Second replicate/EIF5A expt 2Rs_EIF5A_sperm_2.4.tif]

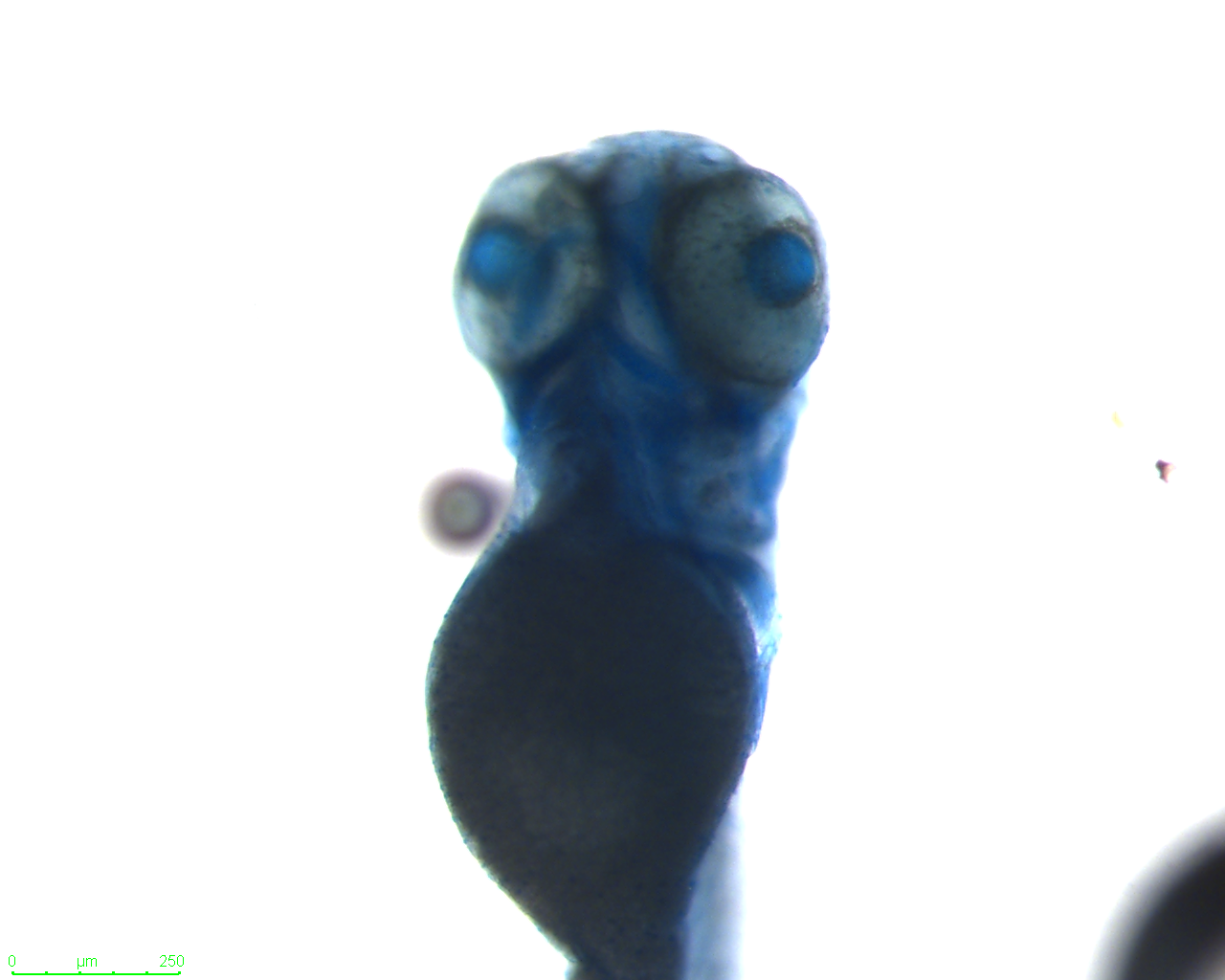

Supplement: Supplementary file 6 — Source Data [file 41467_2021_21053_MOESM6_ESM.zip › Source Data/Zebrafish Morpholino work/Second replicate/EIF5A expt 2Rs_EIF5A_unt 1.1.tif]

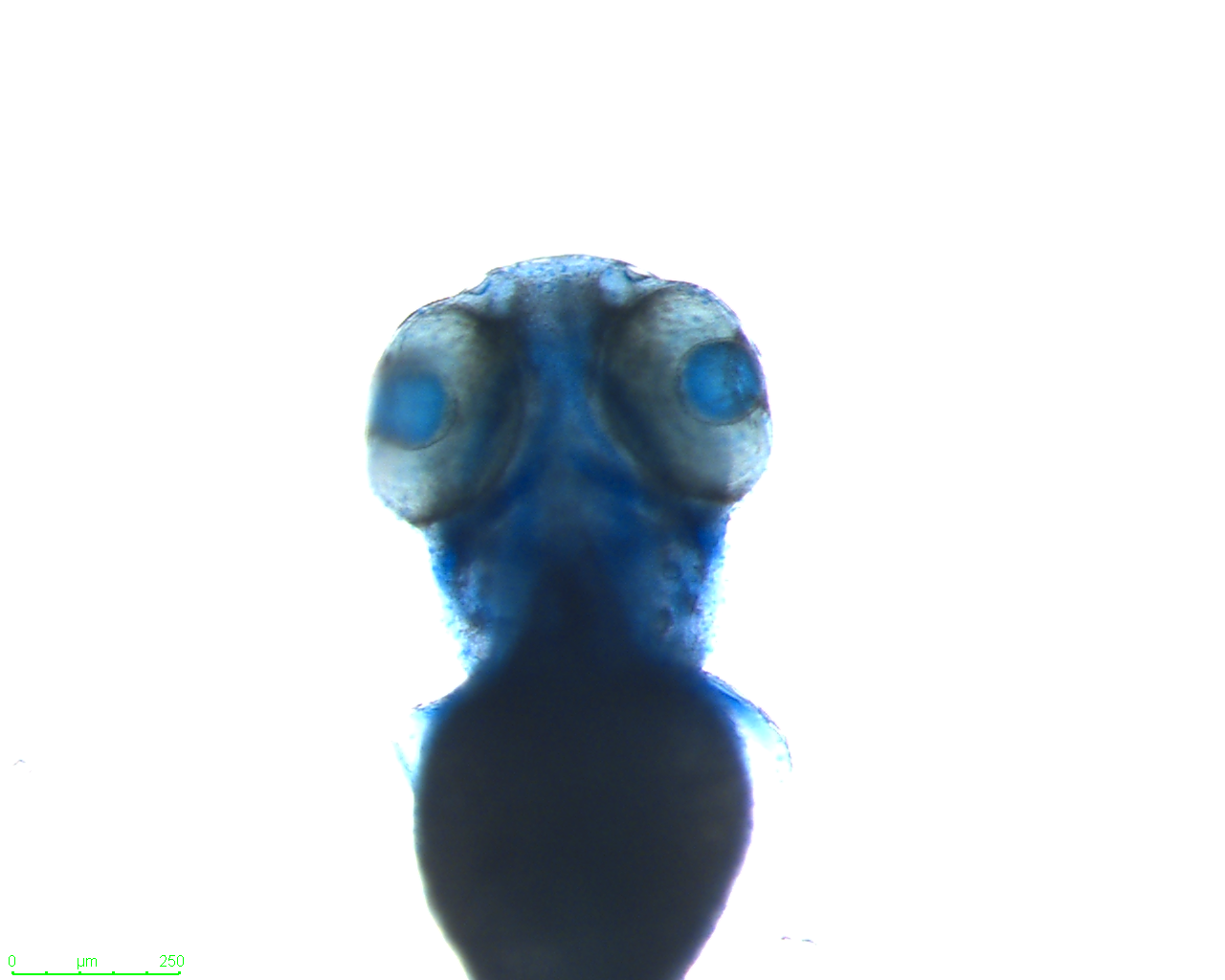

Supplement: Supplementary file 6 — Source Data [file 41467_2021_21053_MOESM6_ESM.zip › Source Data/Zebrafish Morpholino work/Second replicate/EIF5A expt 2Rs_EIF5A_unt 1.10.tif]

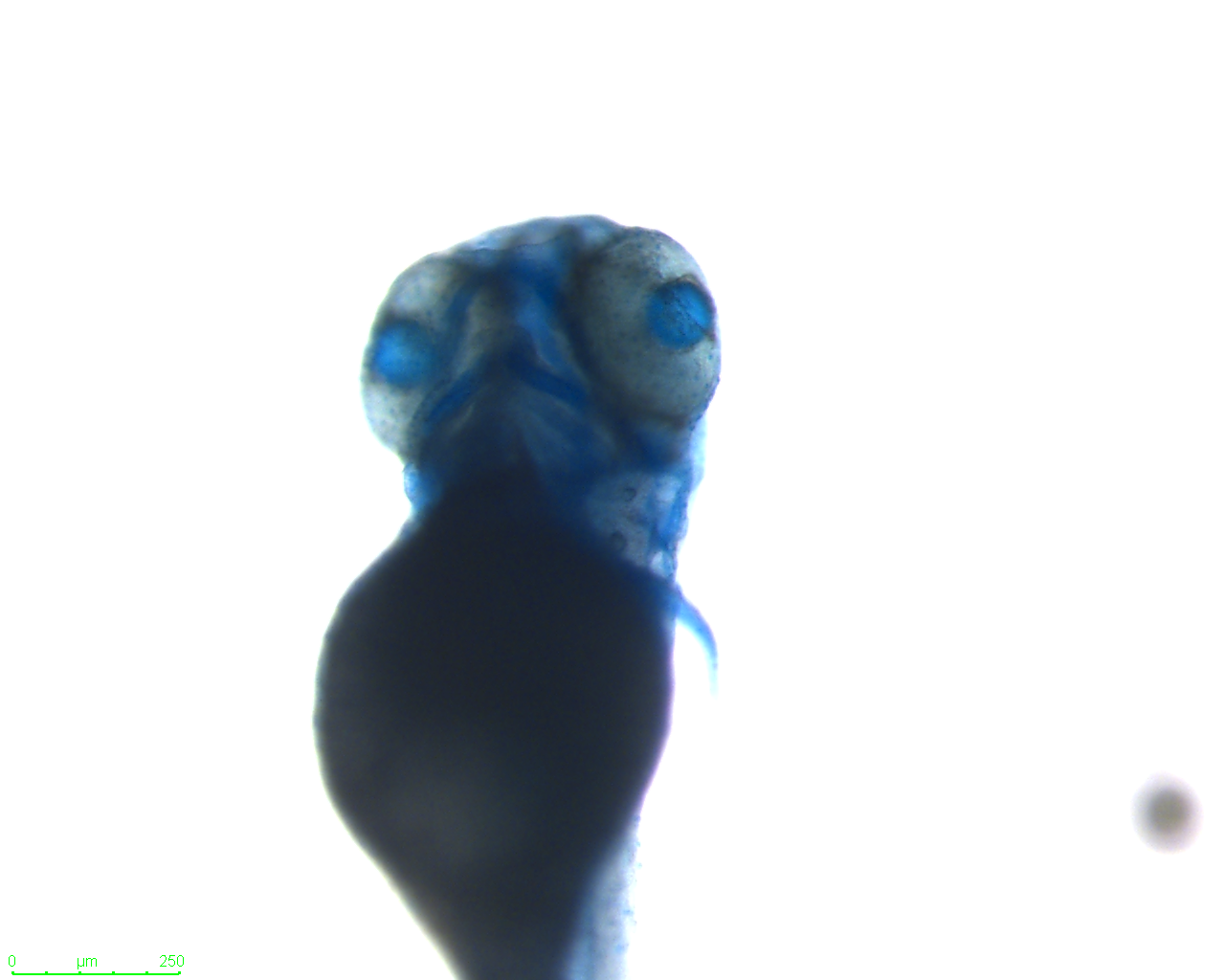

Supplement: Supplementary file 6 — Source Data [file 41467_2021_21053_MOESM6_ESM.zip › Source Data/Zebrafish Morpholino work/Second replicate/EIF5A expt 2Rs_EIF5A_unt 1.2.tif]

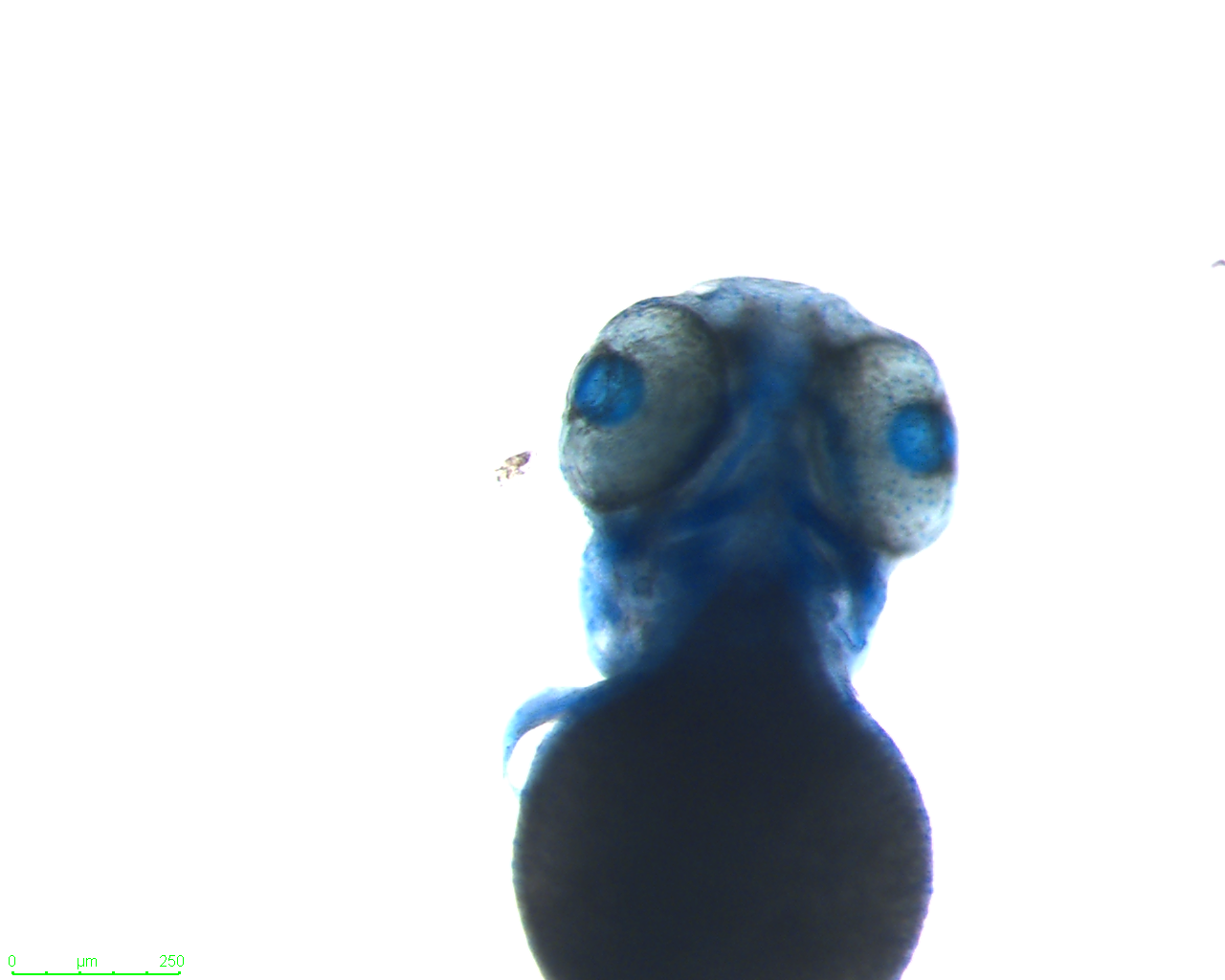

Supplement: Supplementary file 6 — Source Data [file 41467_2021_21053_MOESM6_ESM.zip › Source Data/Zebrafish Morpholino work/Second replicate/EIF5A expt 2Rs_EIF5A_unt 1.3.tif]

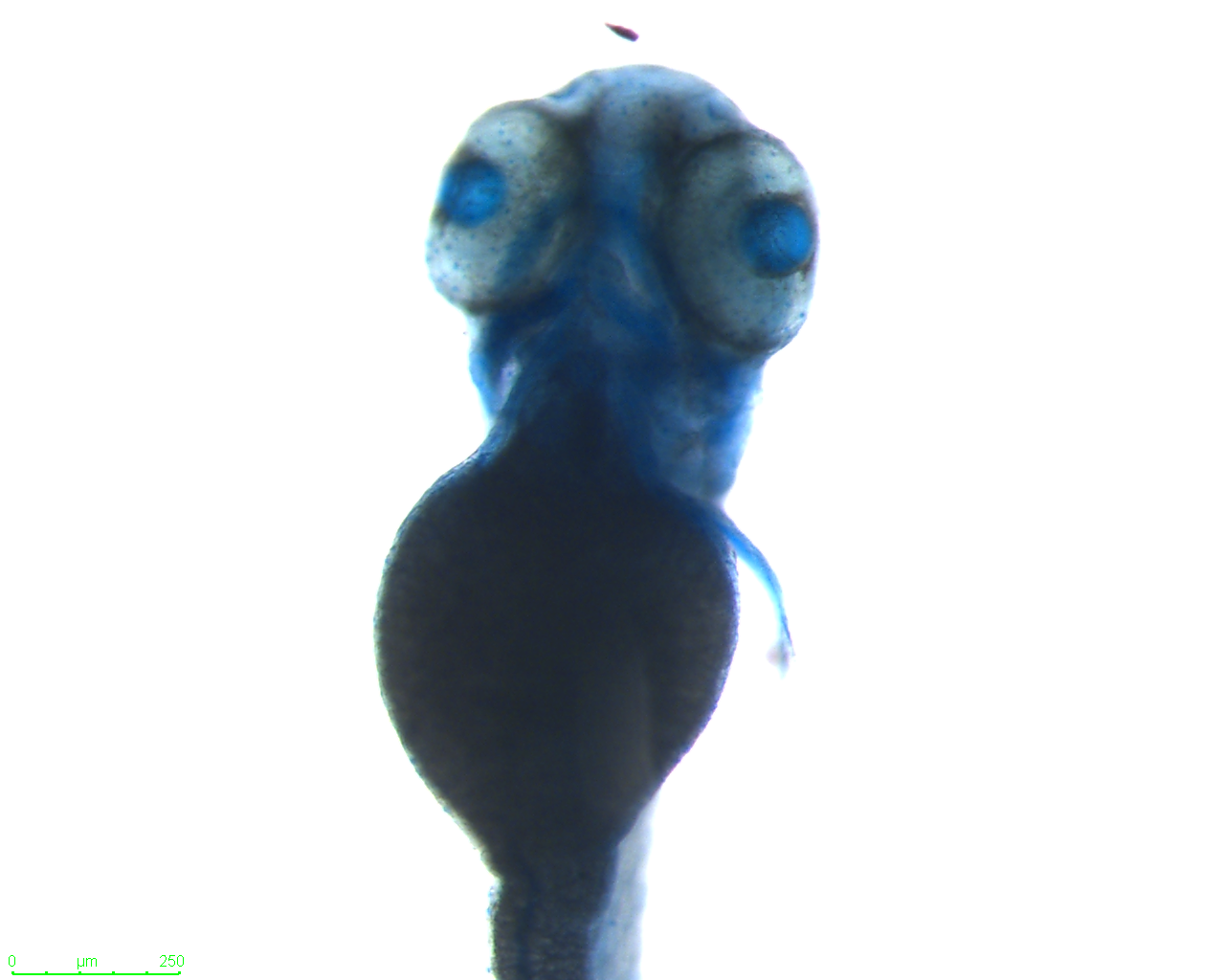

Supplement: Supplementary file 6 — Source Data [file 41467_2021_21053_MOESM6_ESM.zip › Source Data/Zebrafish Morpholino work/Second replicate/EIF5A expt 2Rs_EIF5A_unt 1.4.tif]

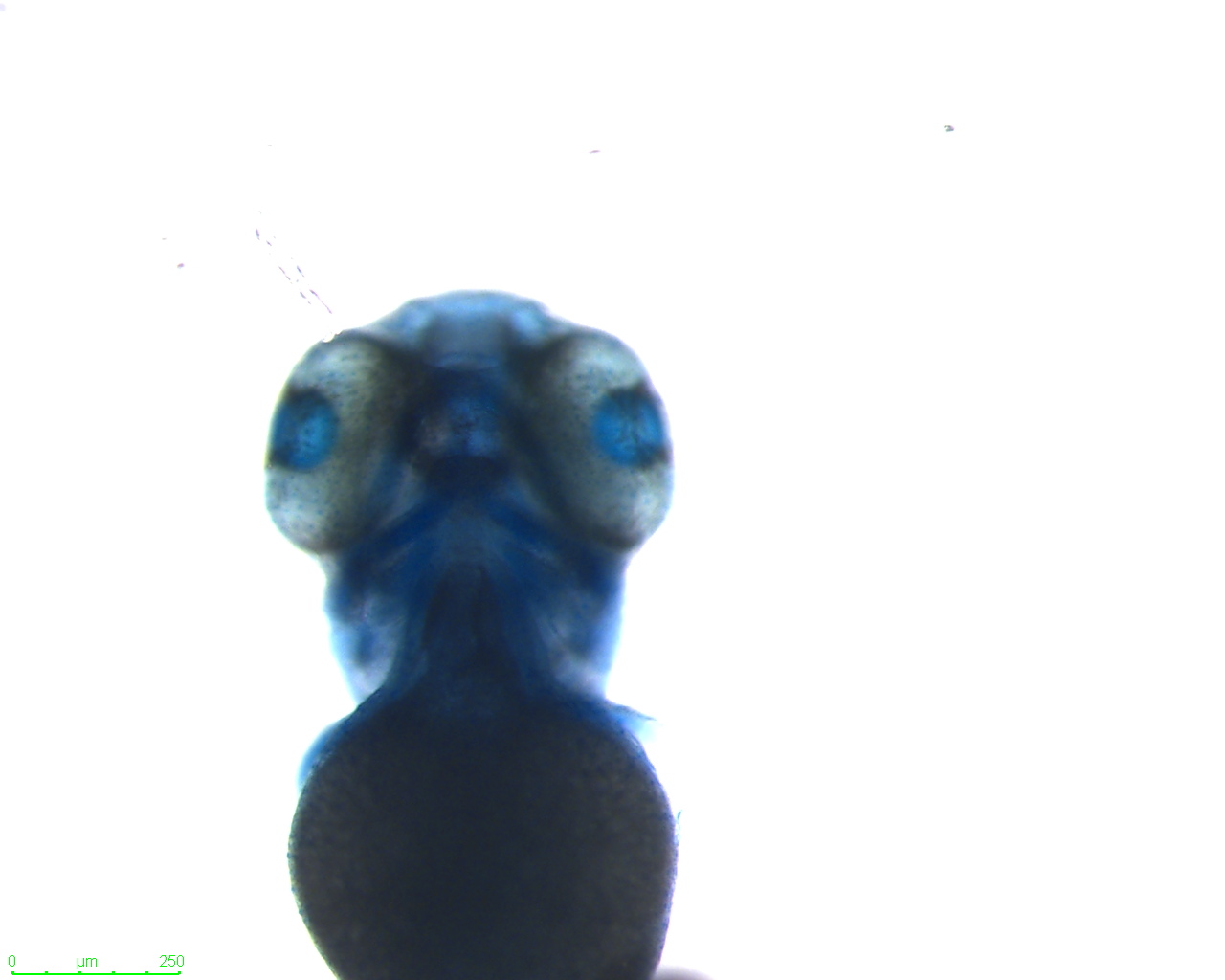

Supplement: Supplementary file 6 — Source Data [file 41467_2021_21053_MOESM6_ESM.zip › Source Data/Zebrafish Morpholino work/Second replicate/EIF5A expt 2Rs_EIF5A_unt 1.5.tif]

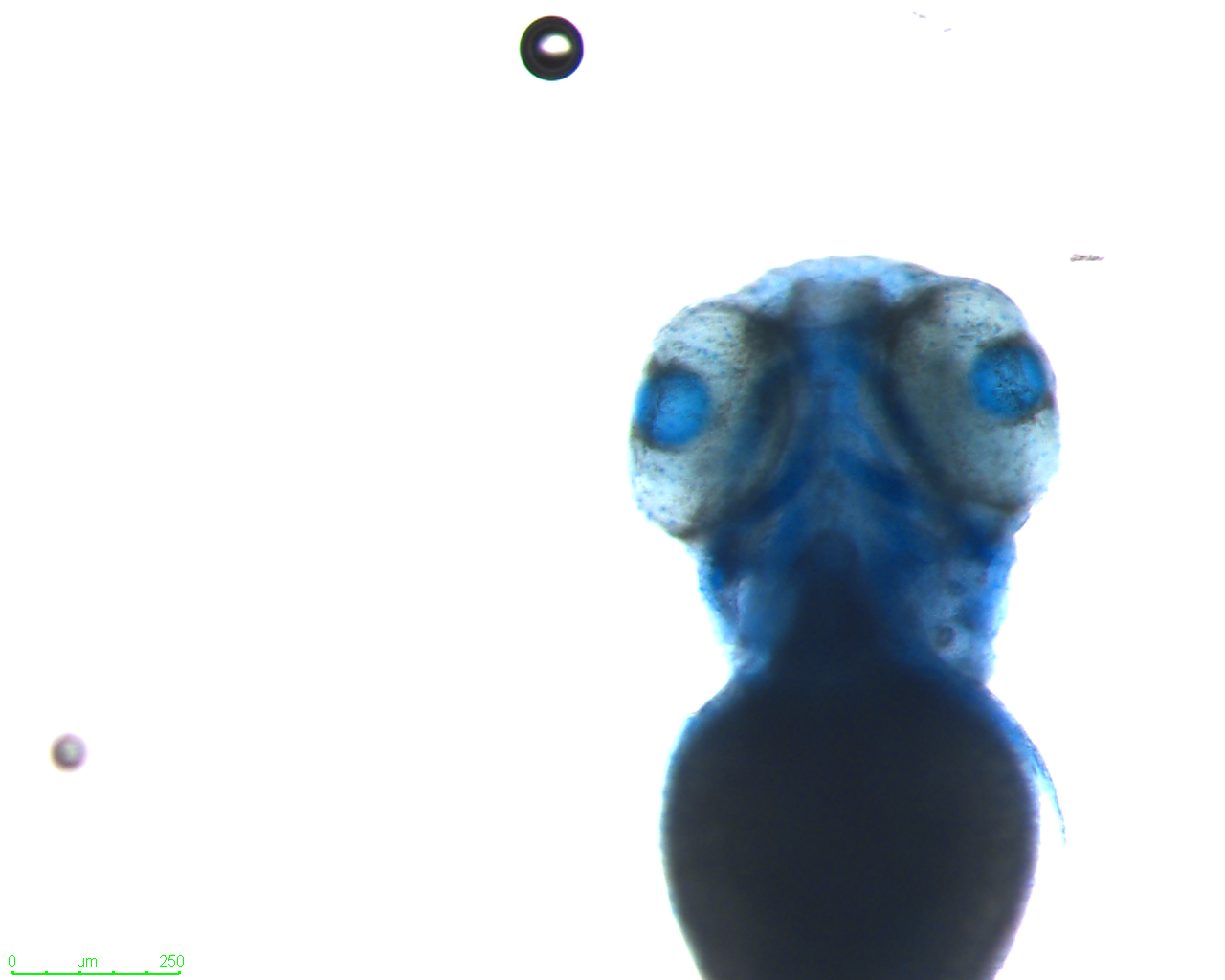

Supplement: Supplementary file 6 — Source Data [file 41467_2021_21053_MOESM6_ESM.zip › Source Data/Zebrafish Morpholino work/Second replicate/EIF5A expt 2Rs_EIF5A_unt 1.6.tif]

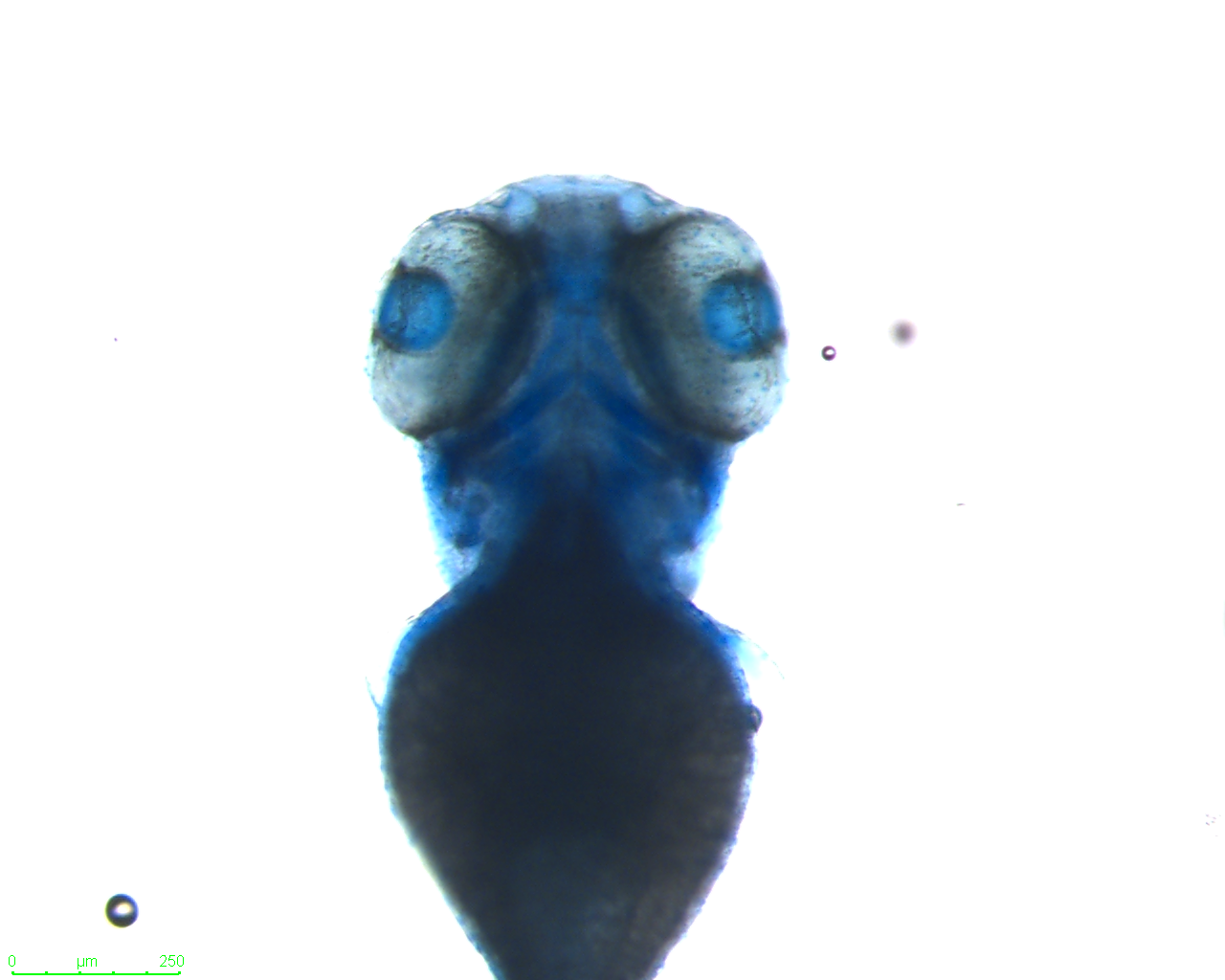

Supplement: Supplementary file 6 — Source Data [file 41467_2021_21053_MOESM6_ESM.zip › Source Data/Zebrafish Morpholino work/Second replicate/EIF5A expt 2Rs_EIF5A_unt 1.7.tif]

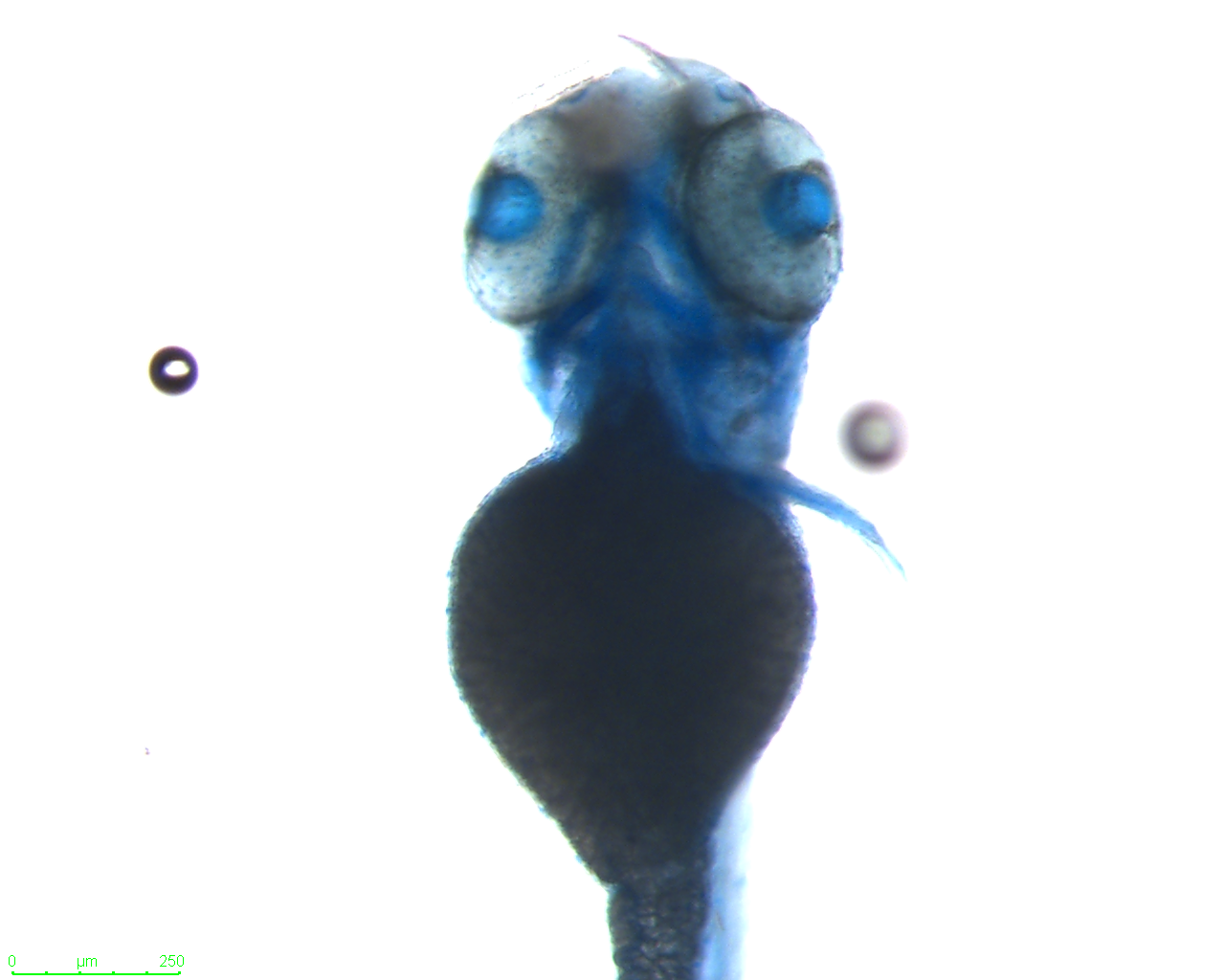

Supplement: Supplementary file 6 — Source Data [file 41467_2021_21053_MOESM6_ESM.zip › Source Data/Zebrafish Morpholino work/Second replicate/EIF5A expt 2Rs_EIF5A_unt 1.8.tif]

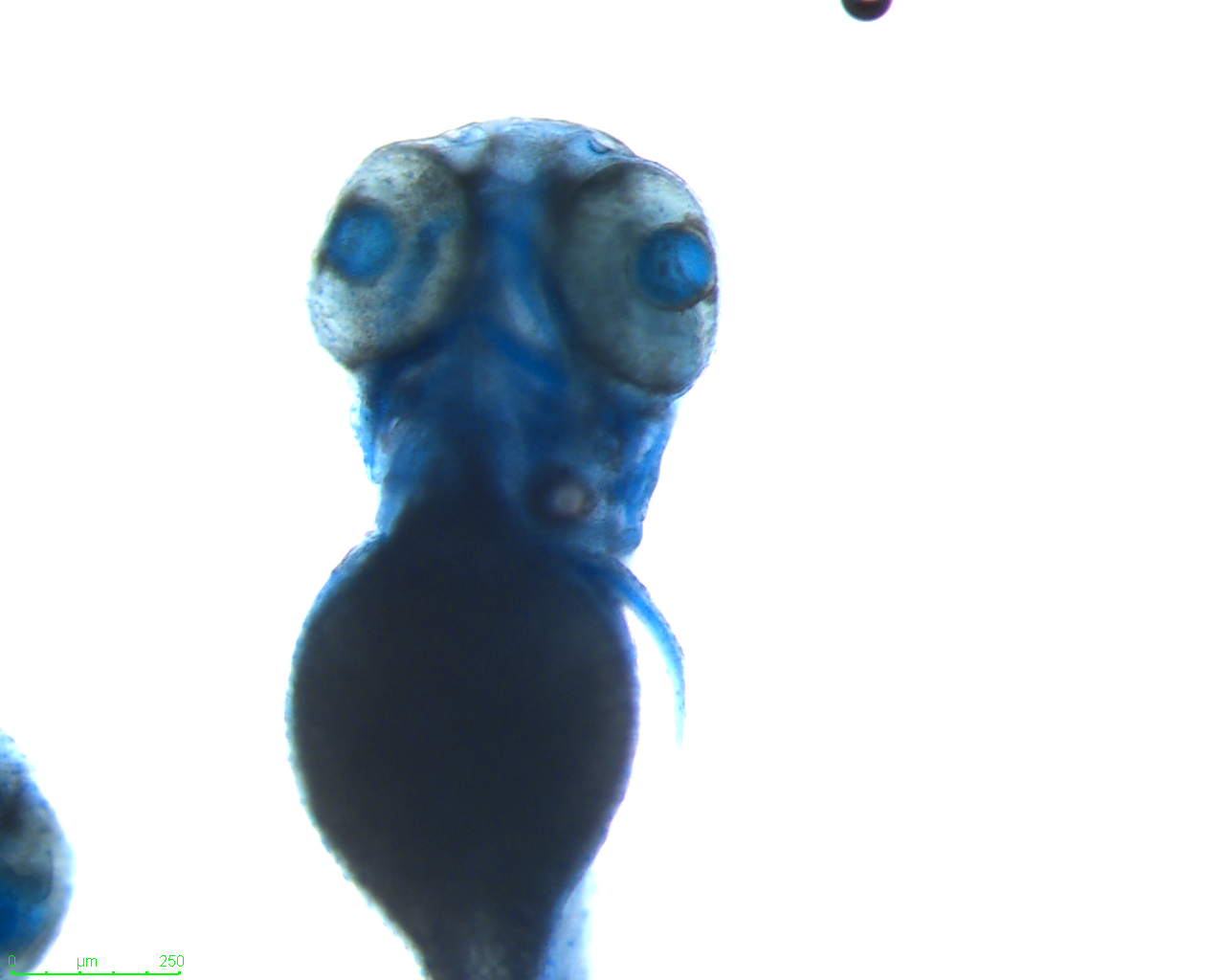

Supplement: Supplementary file 6 — Source Data [file 41467_2021_21053_MOESM6_ESM.zip › Source Data/Zebrafish Morpholino work/Second replicate/EIF5A expt 2Rs_EIF5A_unt 1.9.tif]

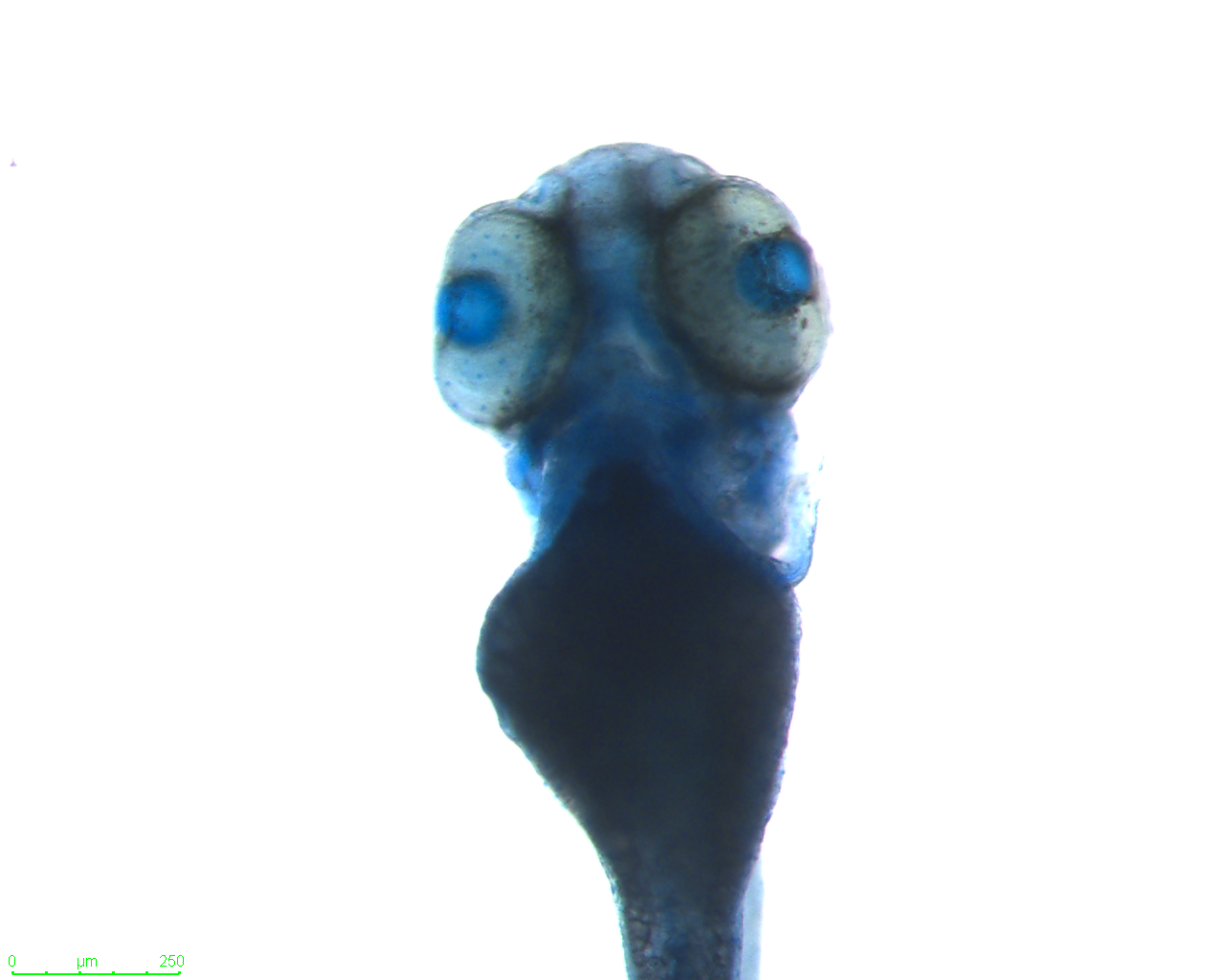

Supplement: Supplementary file 6 — Source Data [file 41467_2021_21053_MOESM6_ESM.zip › Source Data/Zebrafish Morpholino work/Second replicate/EIF5A expt 2Rs_EIF5A_UNT 2.1.tif]

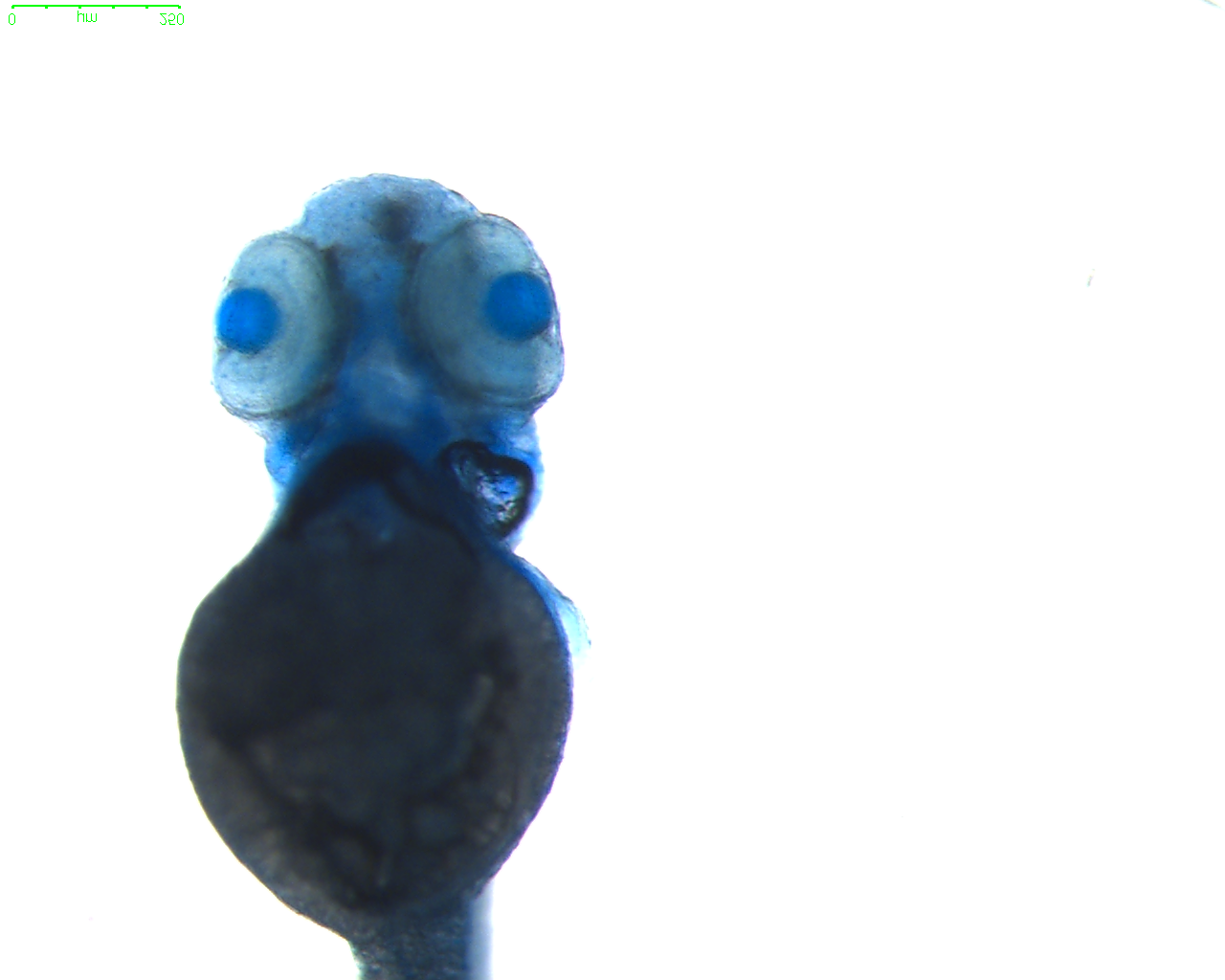

Supplement: Supplementary file 6 — Source Data [file 41467_2021_21053_MOESM6_ESM.zip › Source Data/Zebrafish Morpholino work/Second replicate/EIF5A expt 2Rs_EIF5A_UNT 2.2.tif]

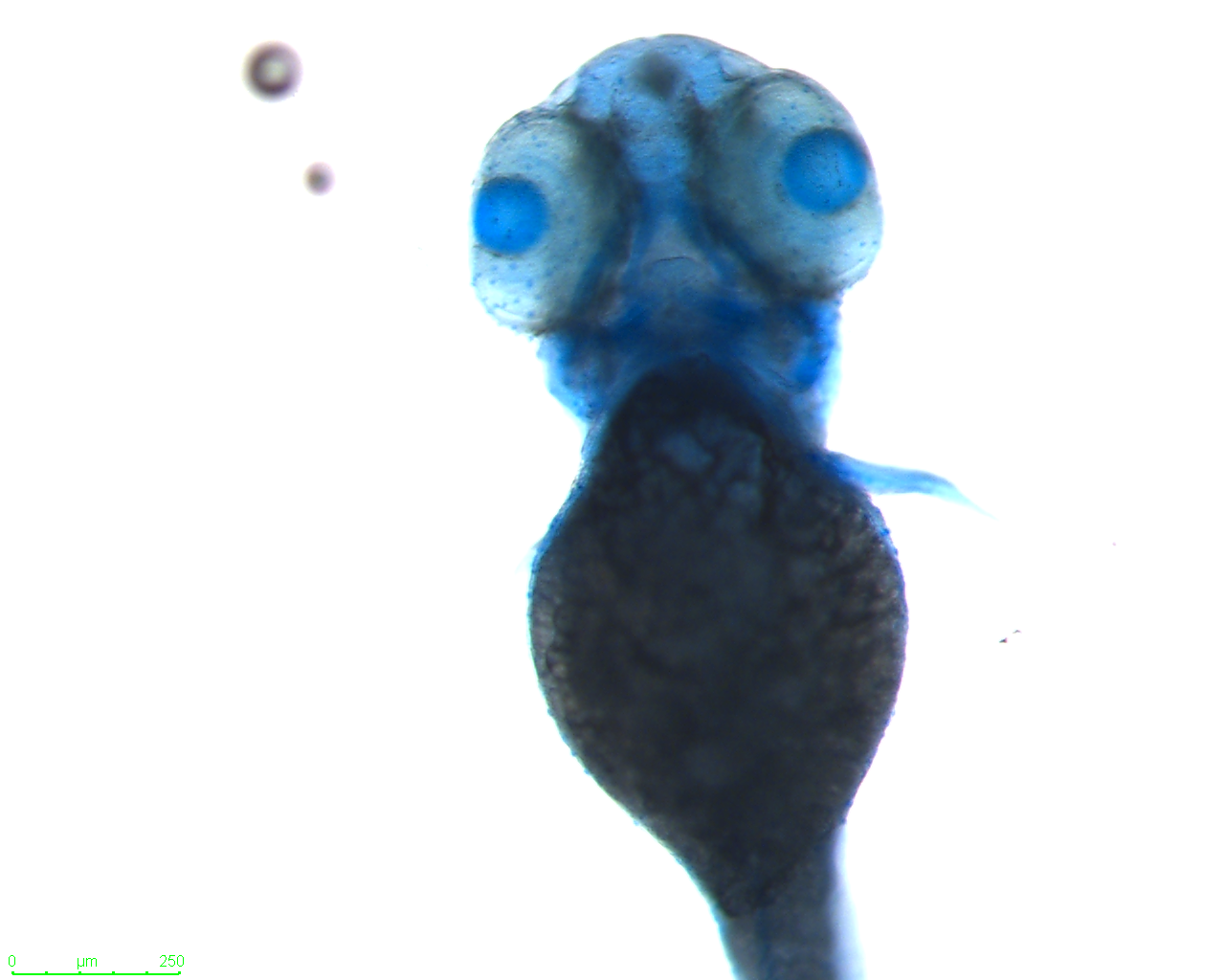

Supplement: Supplementary file 6 — Source Data [file 41467_2021_21053_MOESM6_ESM.zip › Source Data/Zebrafish Morpholino work/Second replicate/EIF5A expt 2Rs_EIF5A_UNT 2.3.tif]

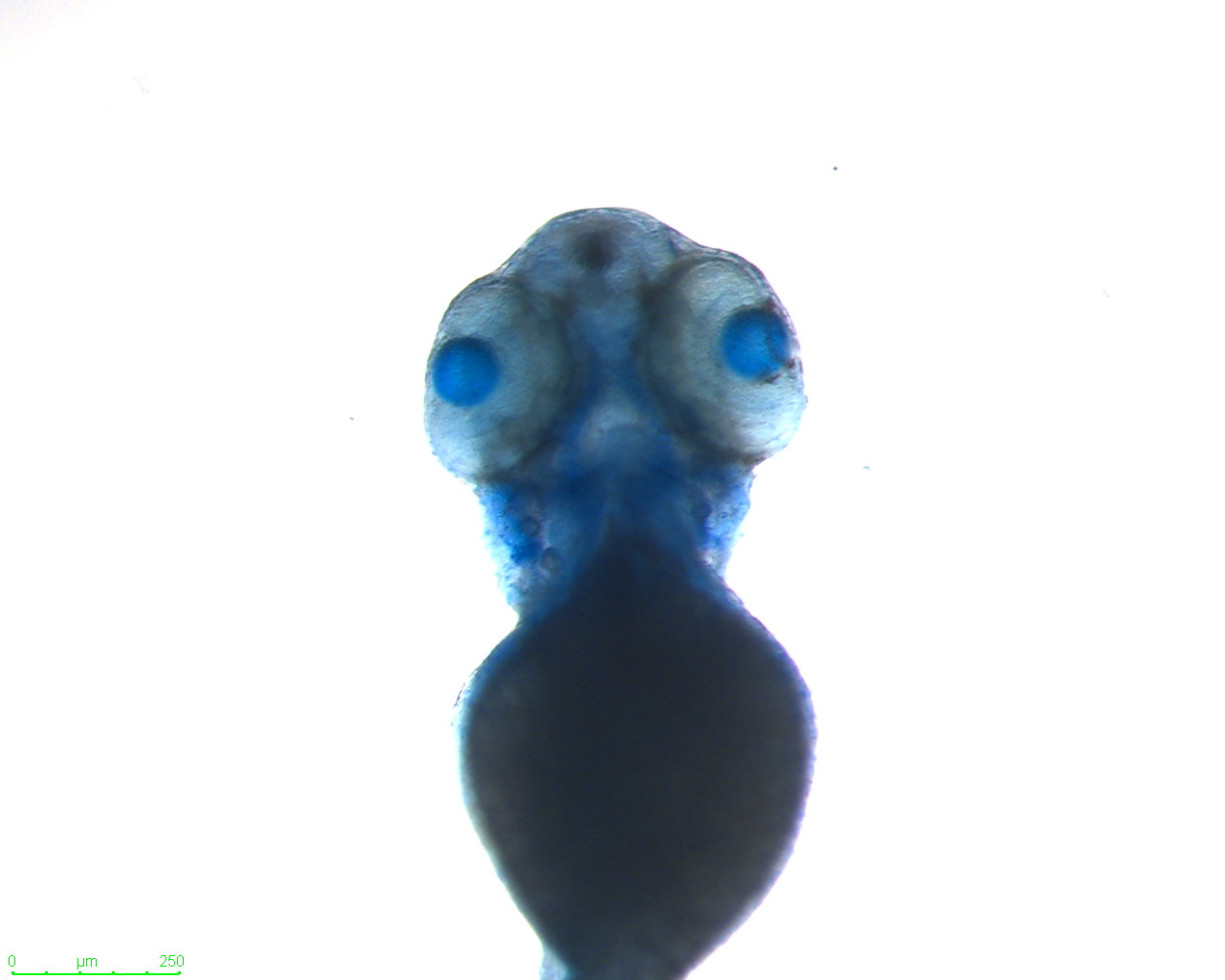

Supplement: Supplementary file 6 — Source Data [file 41467_2021_21053_MOESM6_ESM.zip › Source Data/Zebrafish Morpholino work/Second replicate/EIF5A expt 2Rs_EIF5A_UNT 2.4.tif]

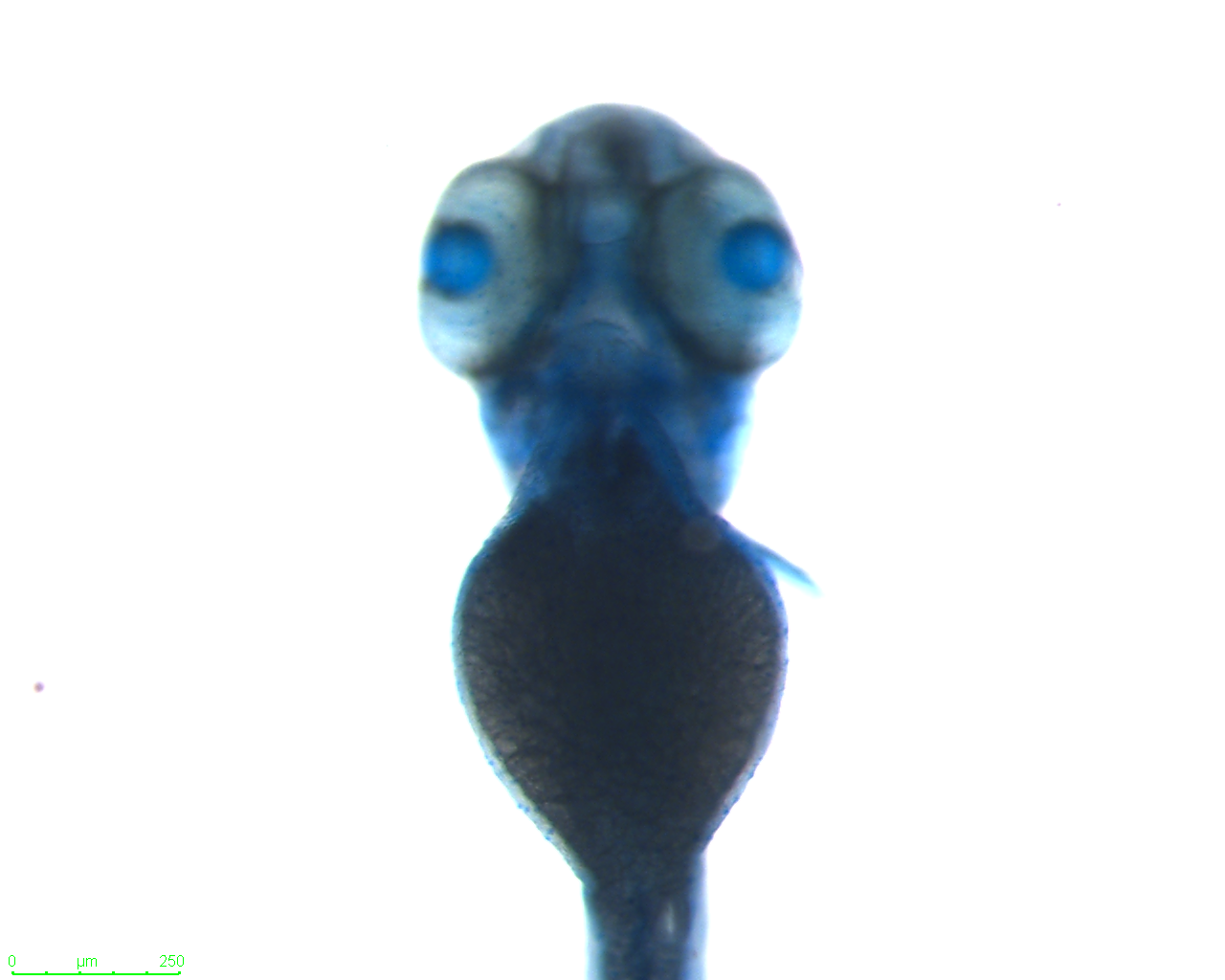

Supplement: Supplementary file 6 — Source Data [file 41467_2021_21053_MOESM6_ESM.zip › Source Data/Zebrafish Morpholino work/Second replicate/EIF5A expt 2Rs_EIF5A_UNT 2.5.tif]

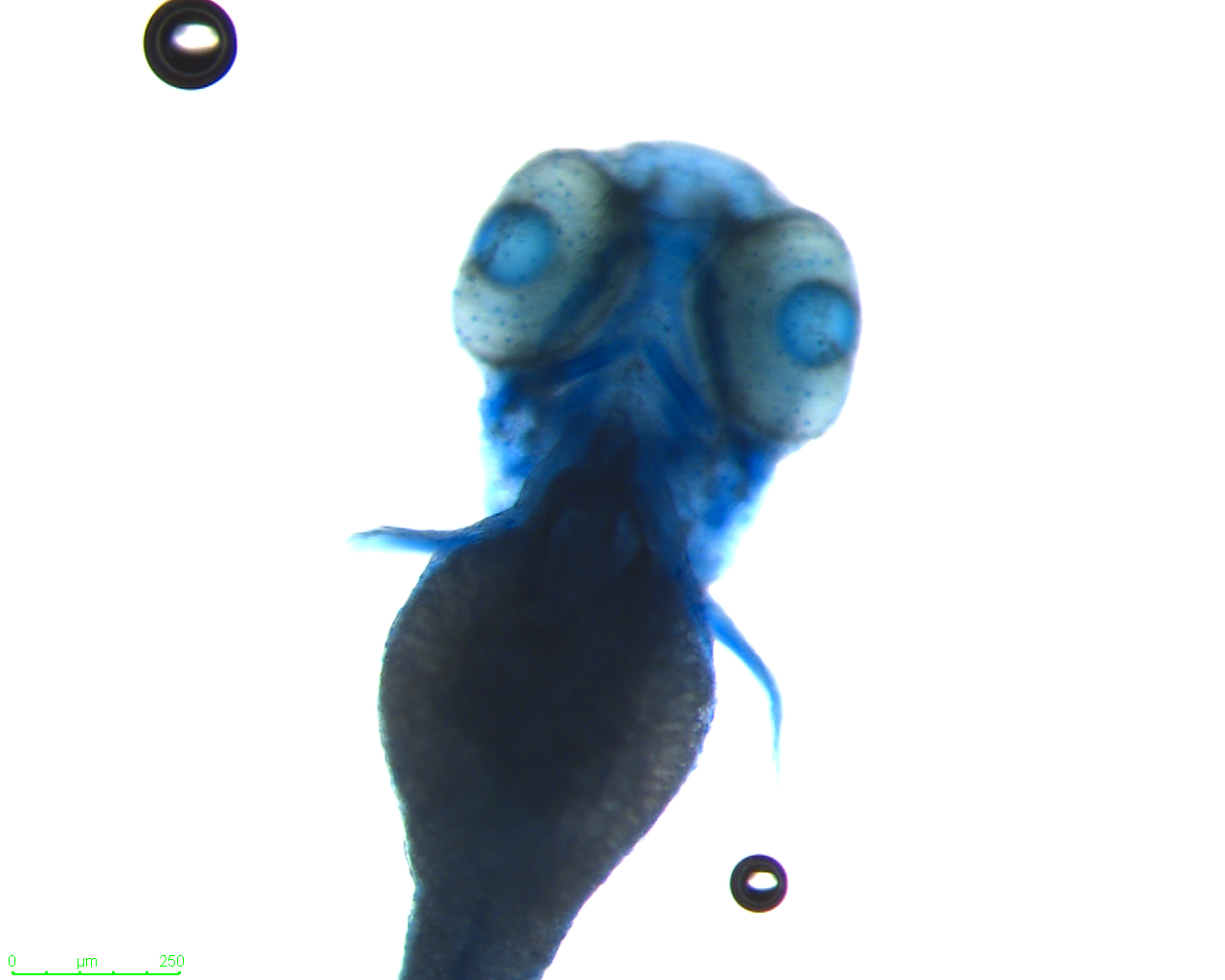

Supplement: Supplementary file 6 — Source Data [file 41467_2021_21053_MOESM6_ESM.zip › Source Data/Zebrafish Morpholino work/Second replicate/EIF5A expt 2Rs_EIF5A_UNT 2.6.tif]

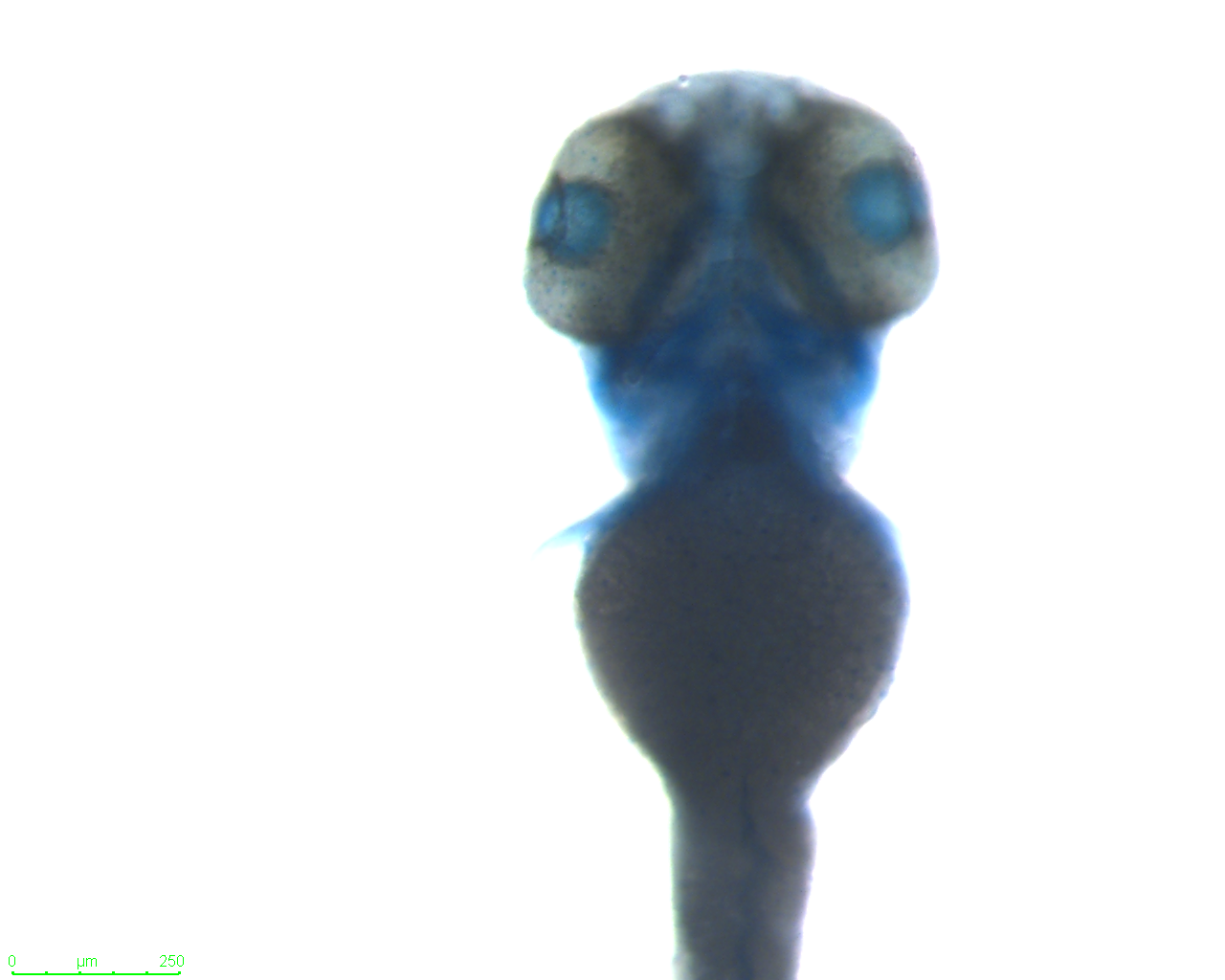

Supplement: Supplementary file 6 — Source Data [file 41467_2021_21053_MOESM6_ESM.zip › Source Data/Zebrafish Morpholino work/Third replicate/EIF5A images 090219_Control_Sperm_01.tif]

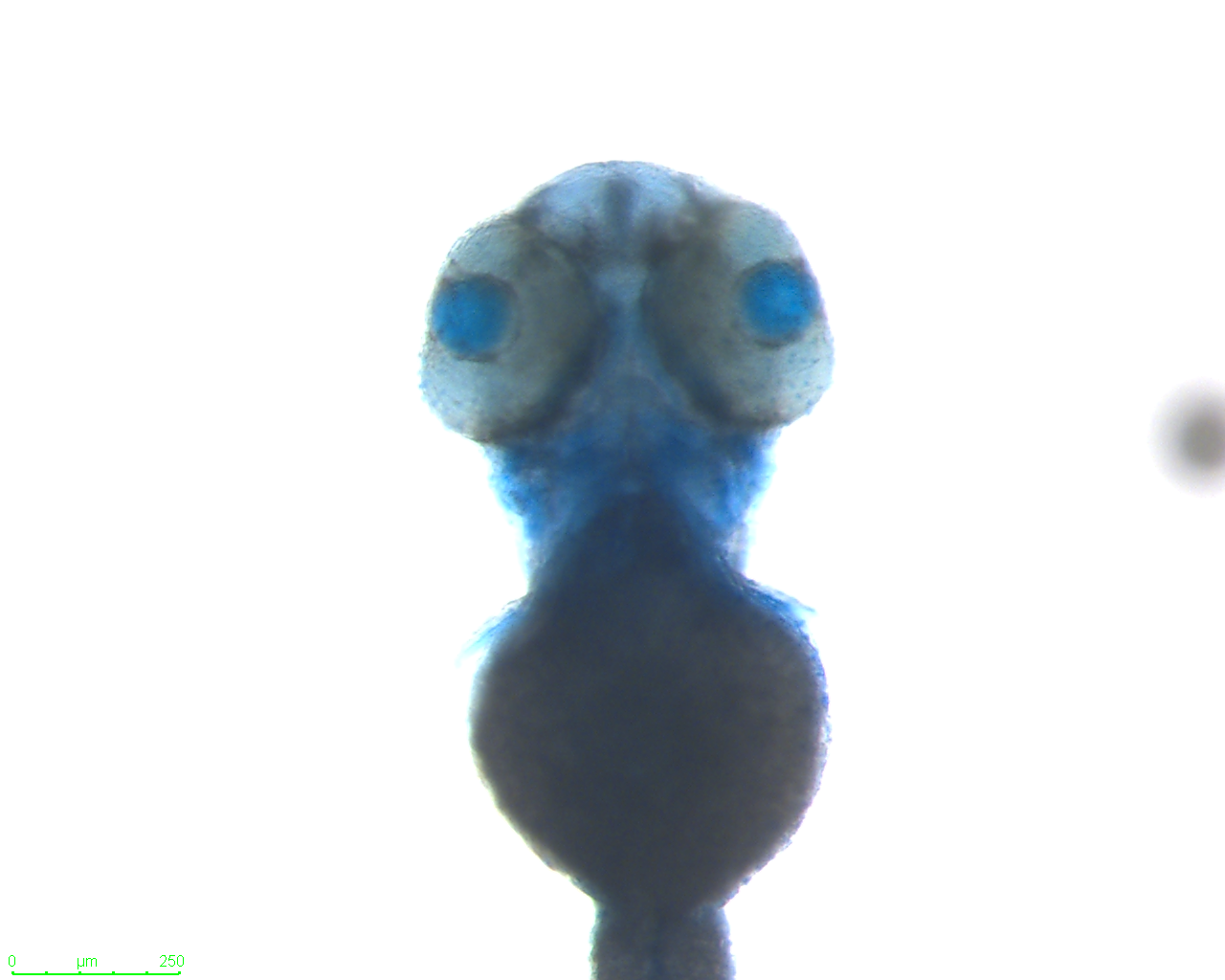

Supplement: Supplementary file 6 — Source Data [file 41467_2021_21053_MOESM6_ESM.zip › Source Data/Zebrafish Morpholino work/Third replicate/EIF5A images 090219_Control_Sperm_02.tif]

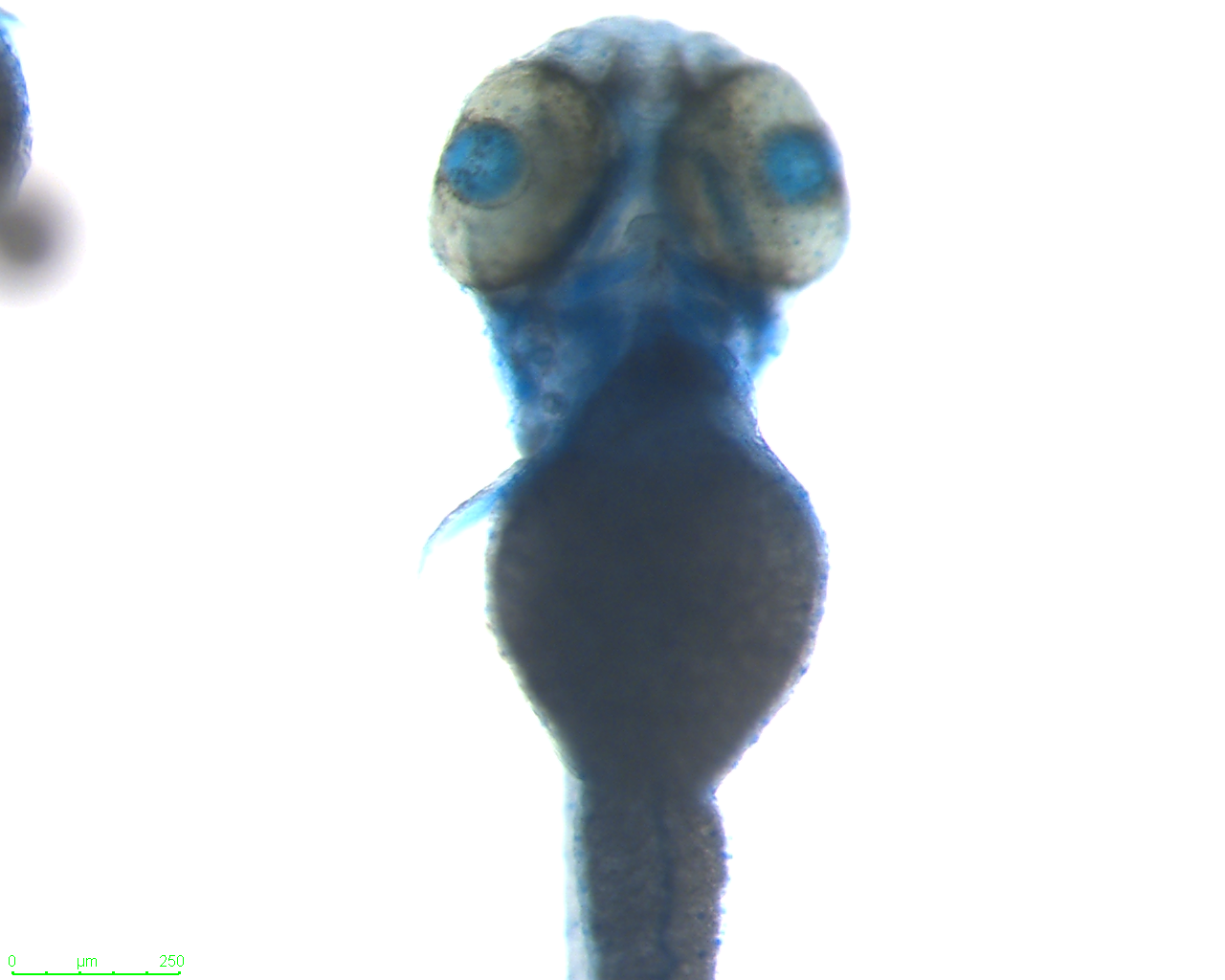

Supplement: Supplementary file 6 — Source Data [file 41467_2021_21053_MOESM6_ESM.zip › Source Data/Zebrafish Morpholino work/Third replicate/EIF5A images 090219_Control_Sperm_03.tif]

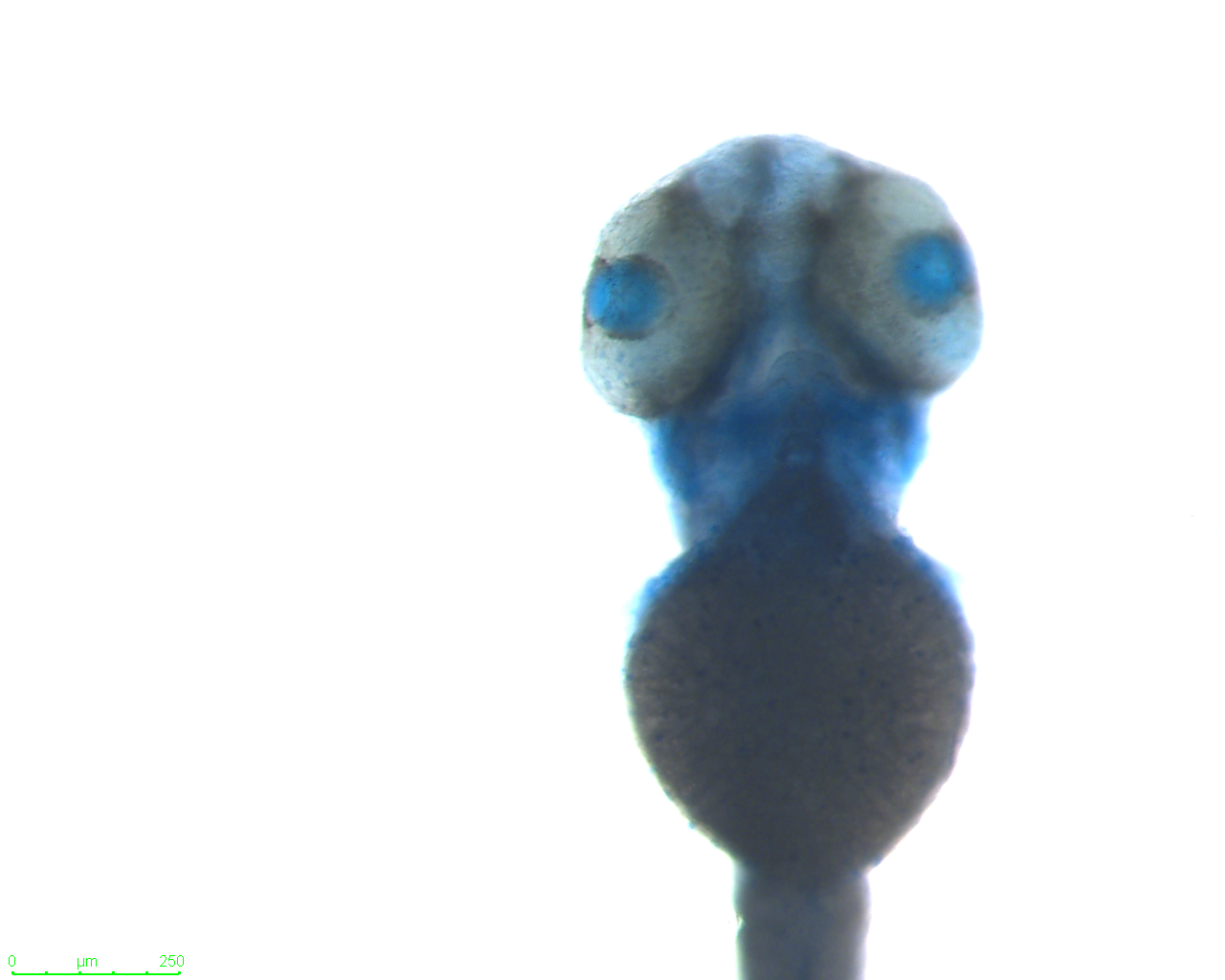

Supplement: Supplementary file 6 — Source Data [file 41467_2021_21053_MOESM6_ESM.zip › Source Data/Zebrafish Morpholino work/Third replicate/EIF5A images 090219_Control_Sperm_04.tif]

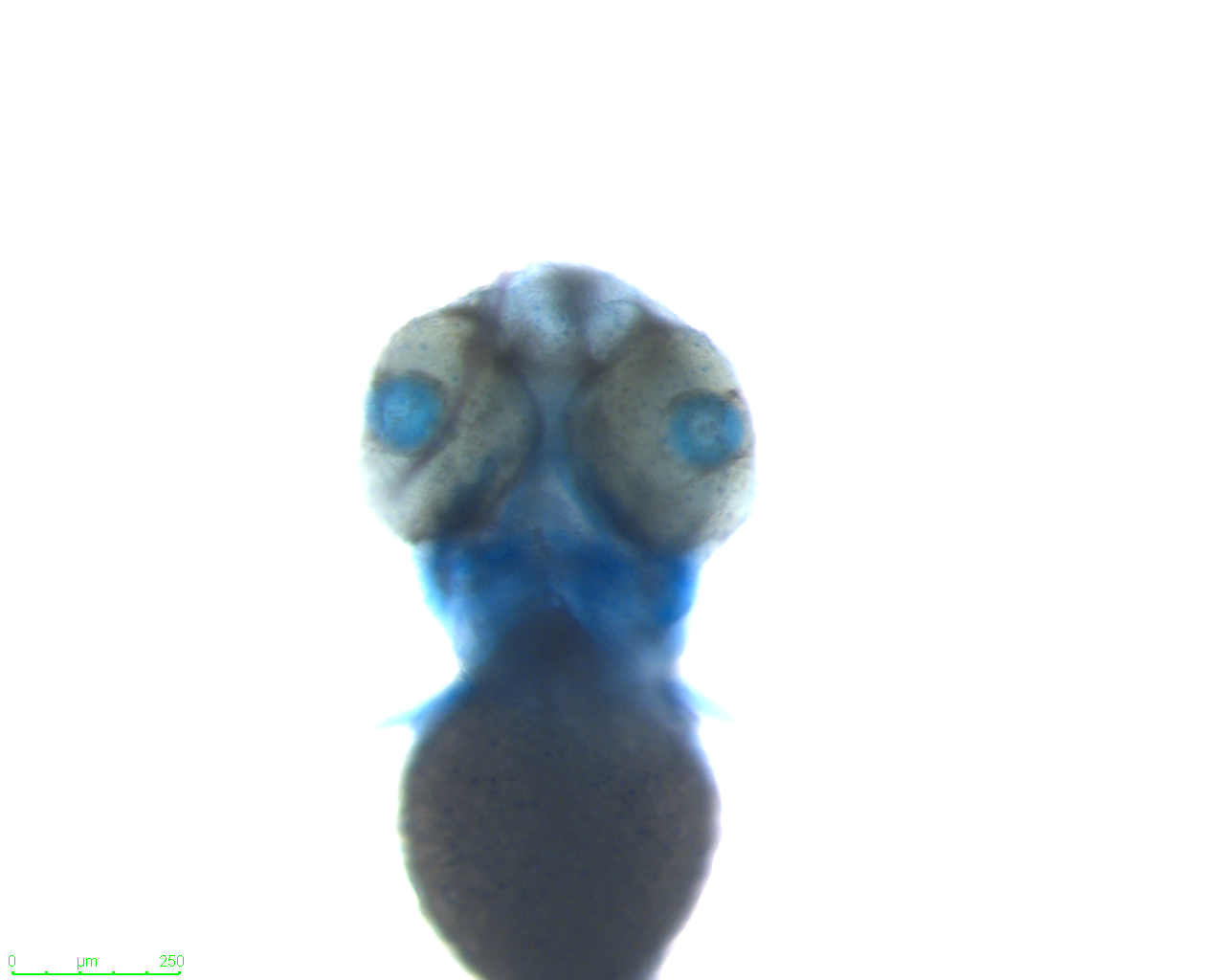

Supplement: Supplementary file 6 — Source Data [file 41467_2021_21053_MOESM6_ESM.zip › Source Data/Zebrafish Morpholino work/Third replicate/EIF5A images 090219_Control_Sperm_05.tif]

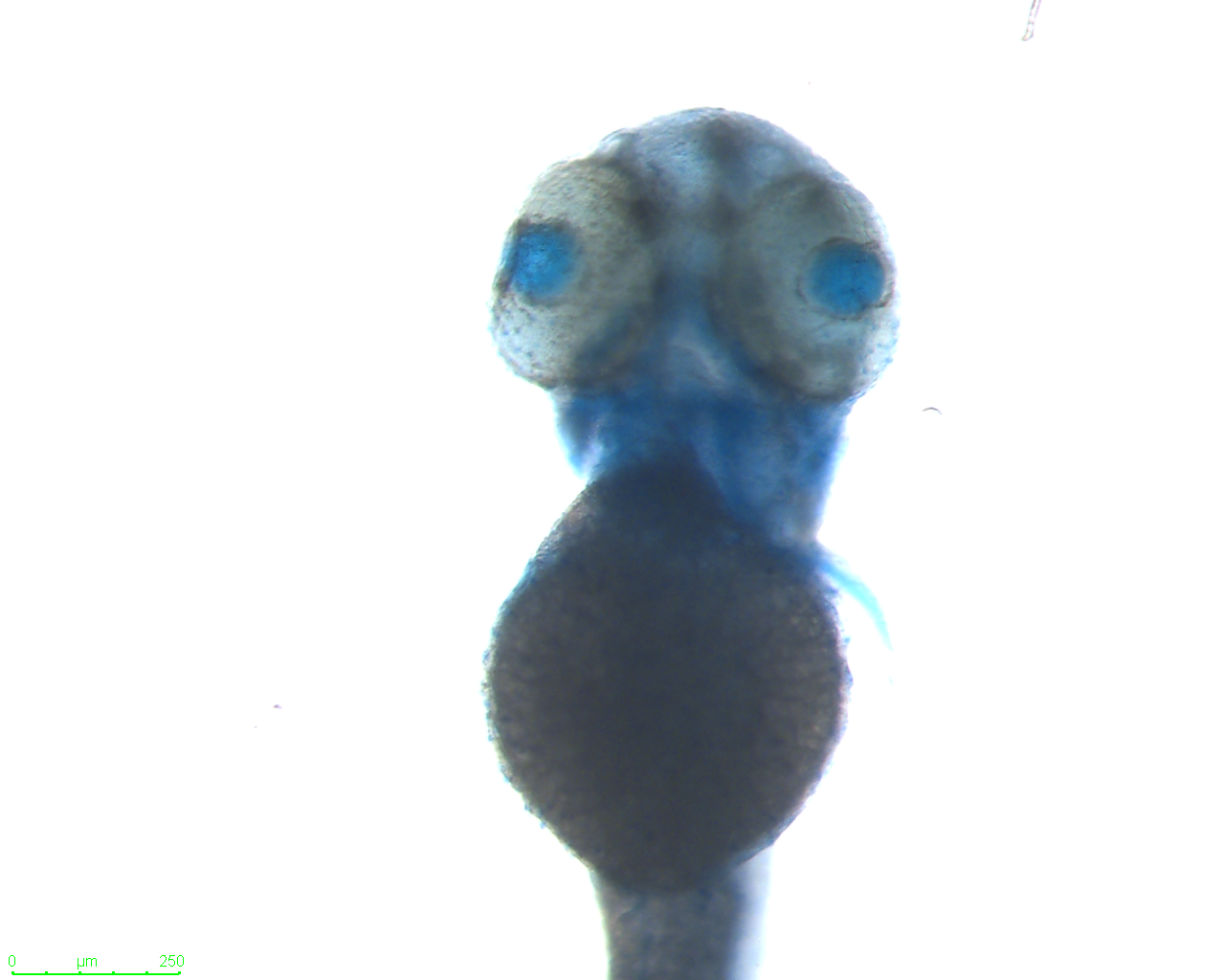

Supplement: Supplementary file 6 — Source Data [file 41467_2021_21053_MOESM6_ESM.zip › Source Data/Zebrafish Morpholino work/Third replicate/EIF5A images 090219_Control_Sperm_06.tif]

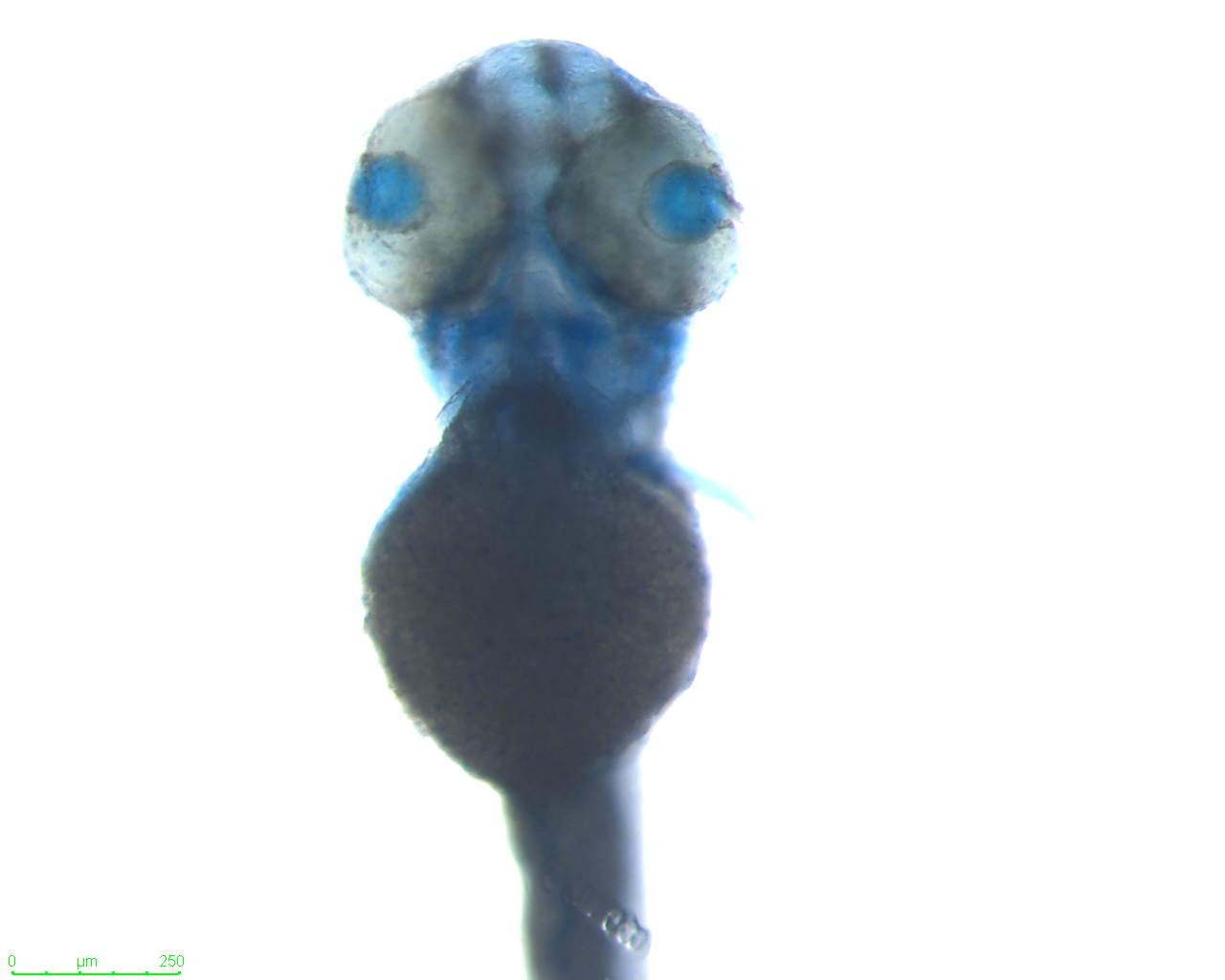

Supplement: Supplementary file 6 — Source Data [file 41467_2021_21053_MOESM6_ESM.zip › Source Data/Zebrafish Morpholino work/Third replicate/EIF5A images 090219_Control_Sperm_07.tif]

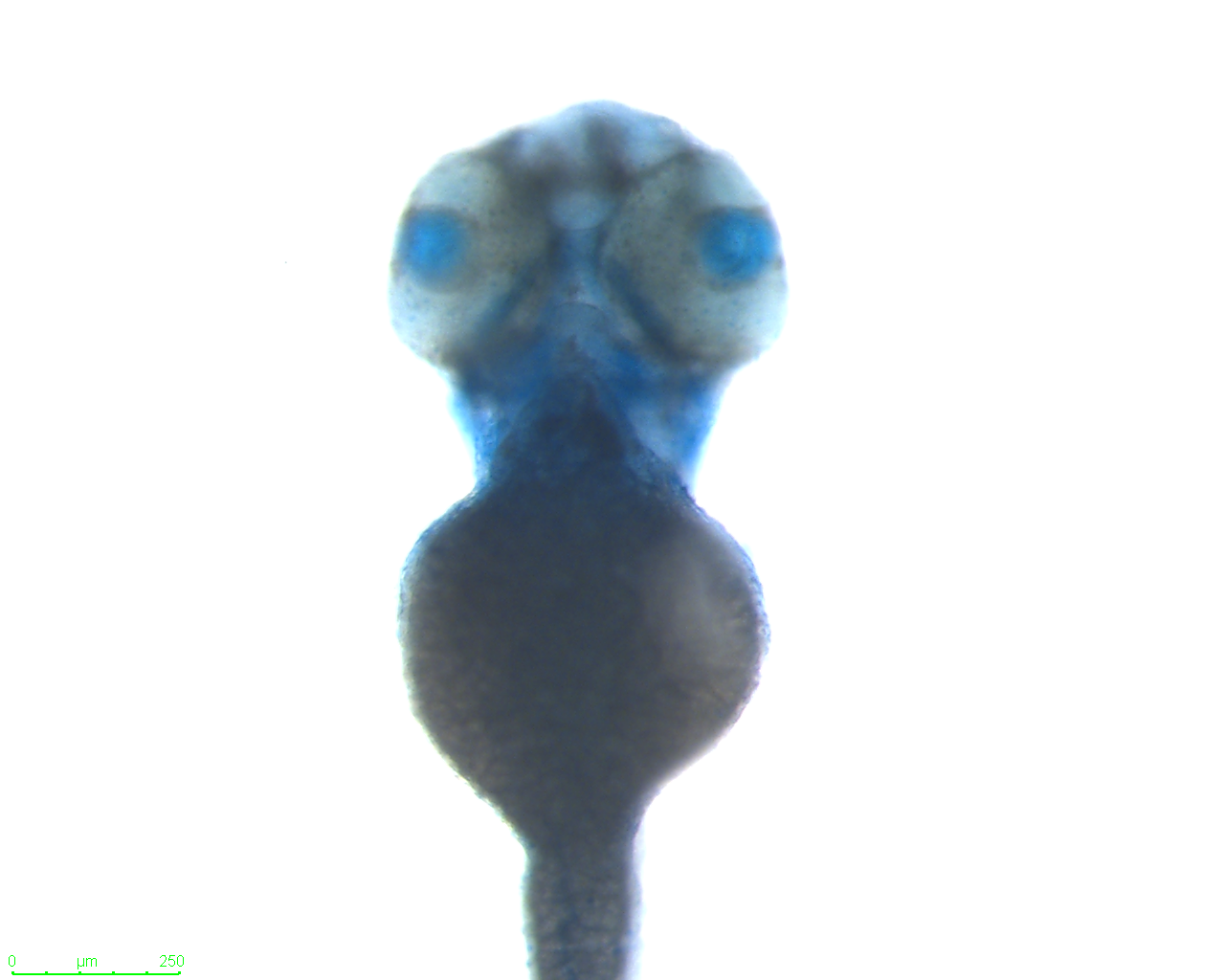

Supplement: Supplementary file 6 — Source Data [file 41467_2021_21053_MOESM6_ESM.zip › Source Data/Zebrafish Morpholino work/Third replicate/EIF5A images 090219_Control_Sperm_08.tif]

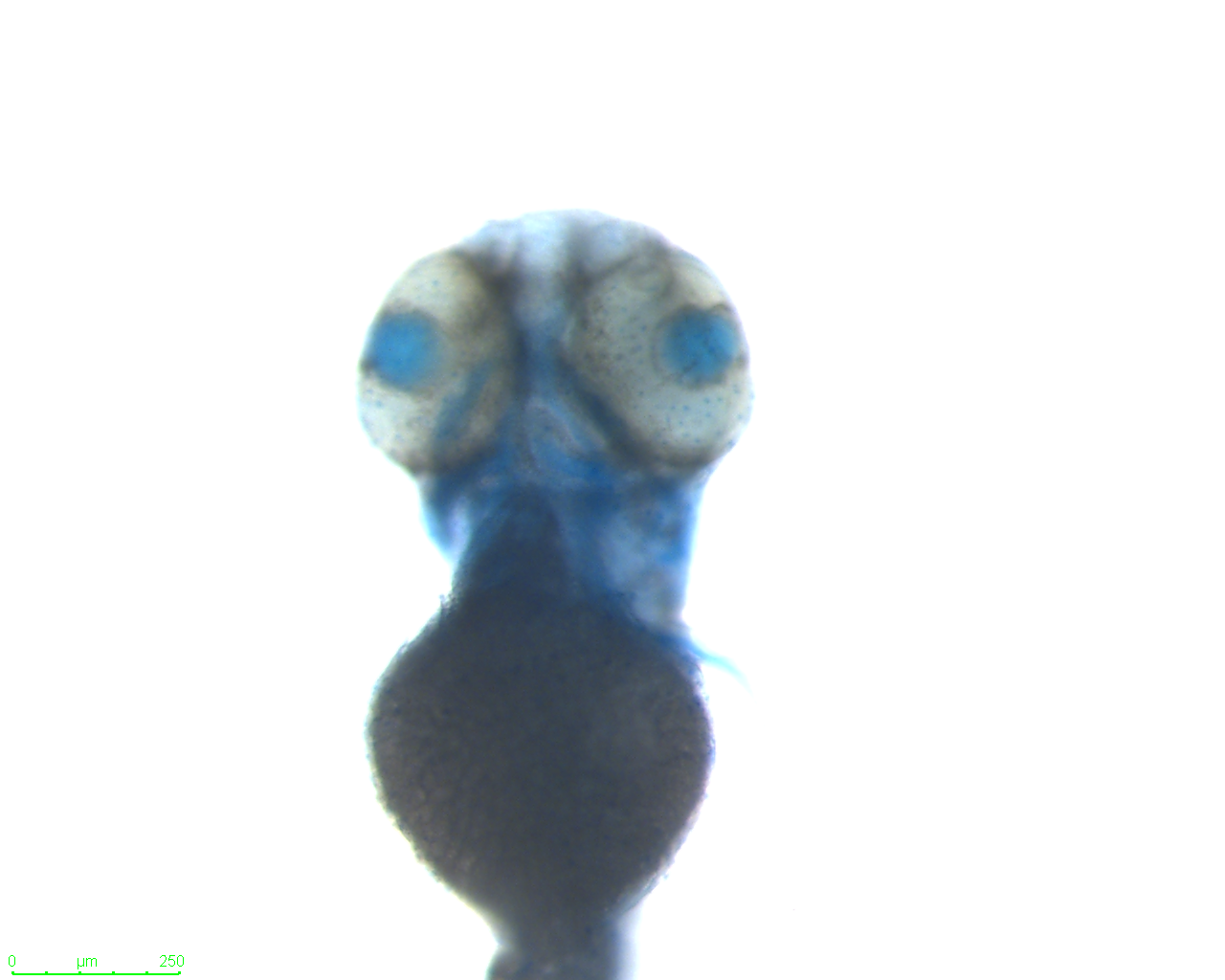

Supplement: Supplementary file 6 — Source Data [file 41467_2021_21053_MOESM6_ESM.zip › Source Data/Zebrafish Morpholino work/Third replicate/EIF5A images 090219_Control_Sperm_09.tif]

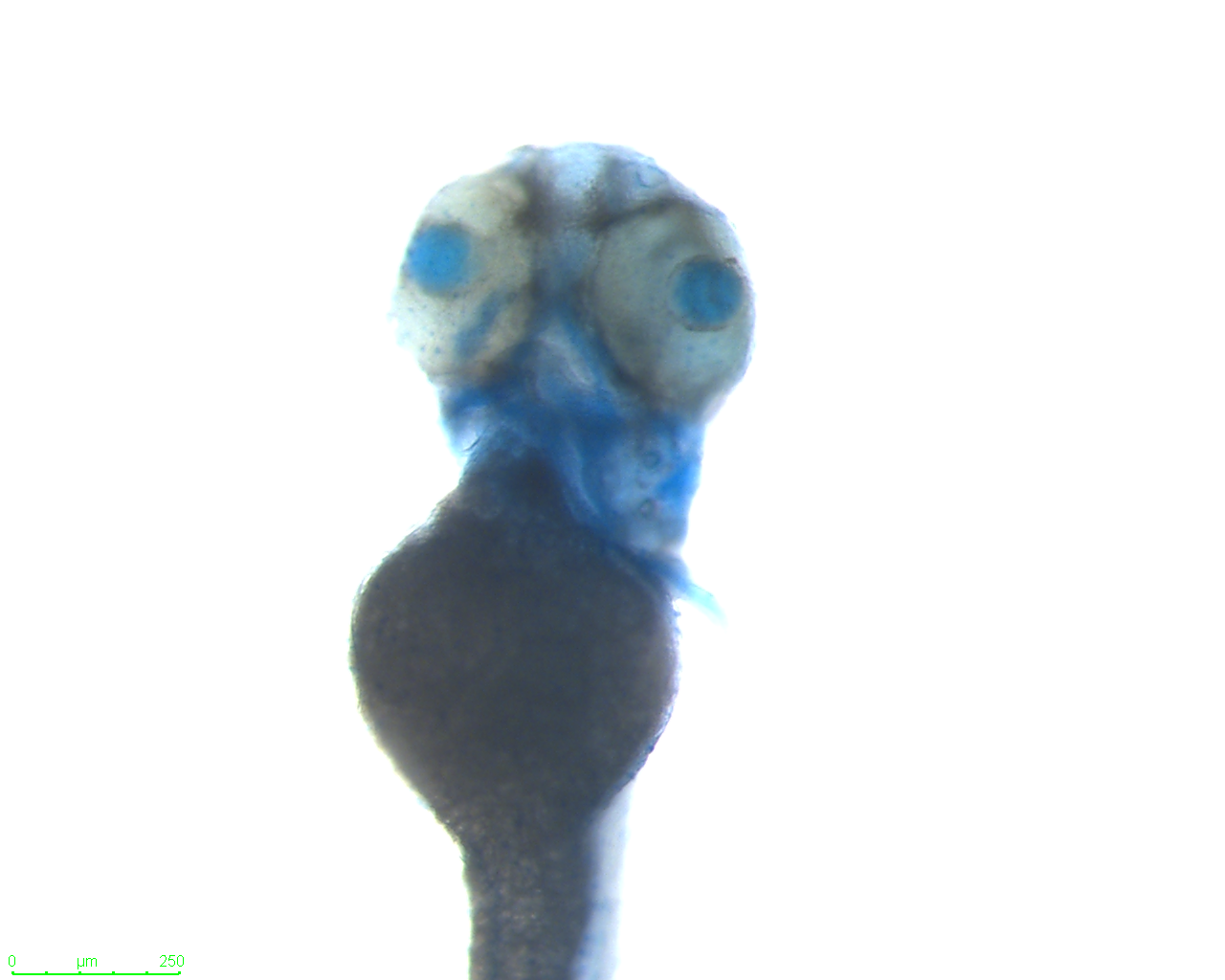

Supplement: Supplementary file 6 — Source Data [file 41467_2021_21053_MOESM6_ESM.zip › Source Data/Zebrafish Morpholino work/Third replicate/EIF5A images 090219_Control_Sperm_10.tif]

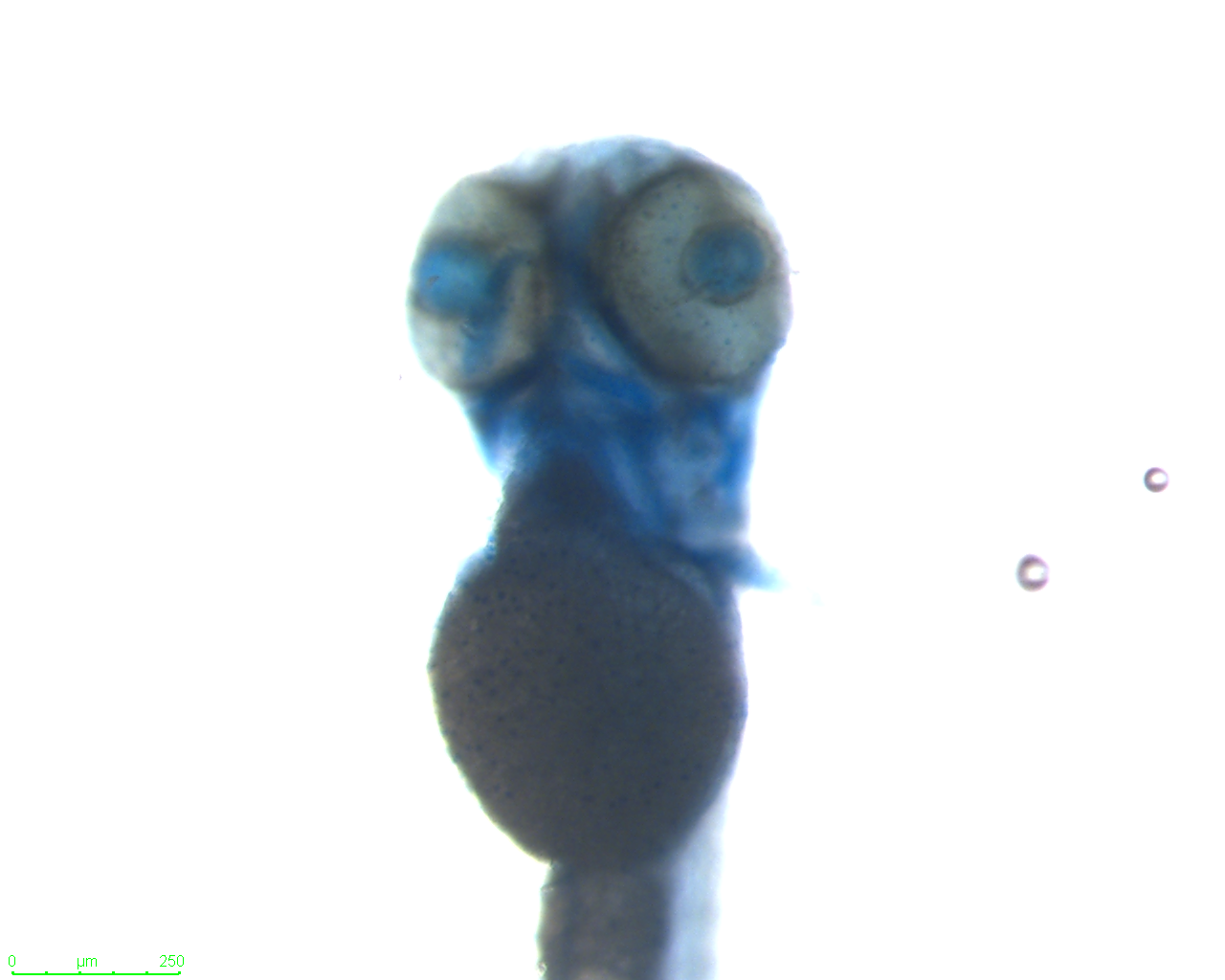

Supplement: Supplementary file 6 — Source Data [file 41467_2021_21053_MOESM6_ESM.zip › Source Data/Zebrafish Morpholino work/Third replicate/EIF5A images 090219_Control_Sperm_11.tif]

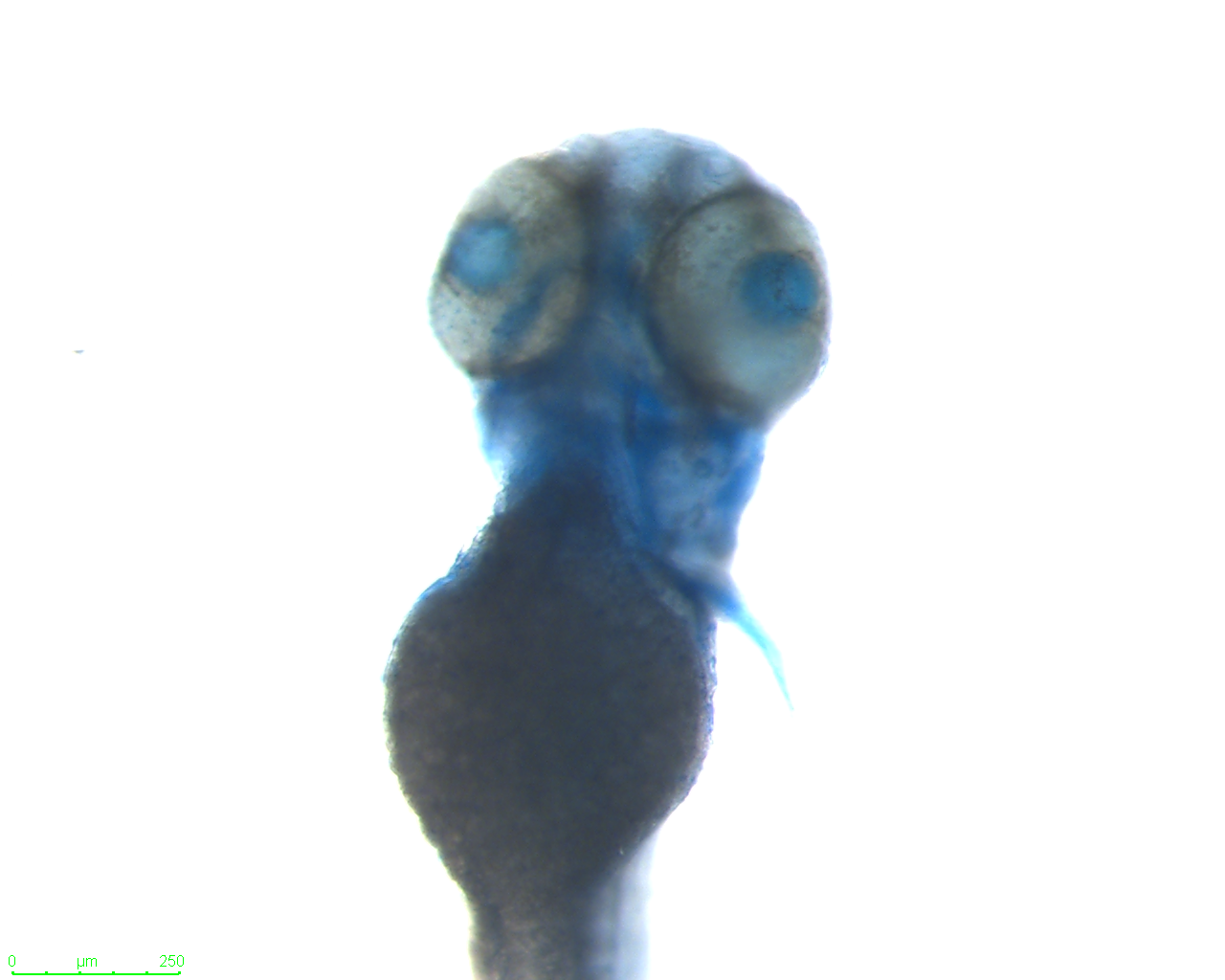

Supplement: Supplementary file 6 — Source Data [file 41467_2021_21053_MOESM6_ESM.zip › Source Data/Zebrafish Morpholino work/Third replicate/EIF5A images 090219_Control_Sperm_12.tif]

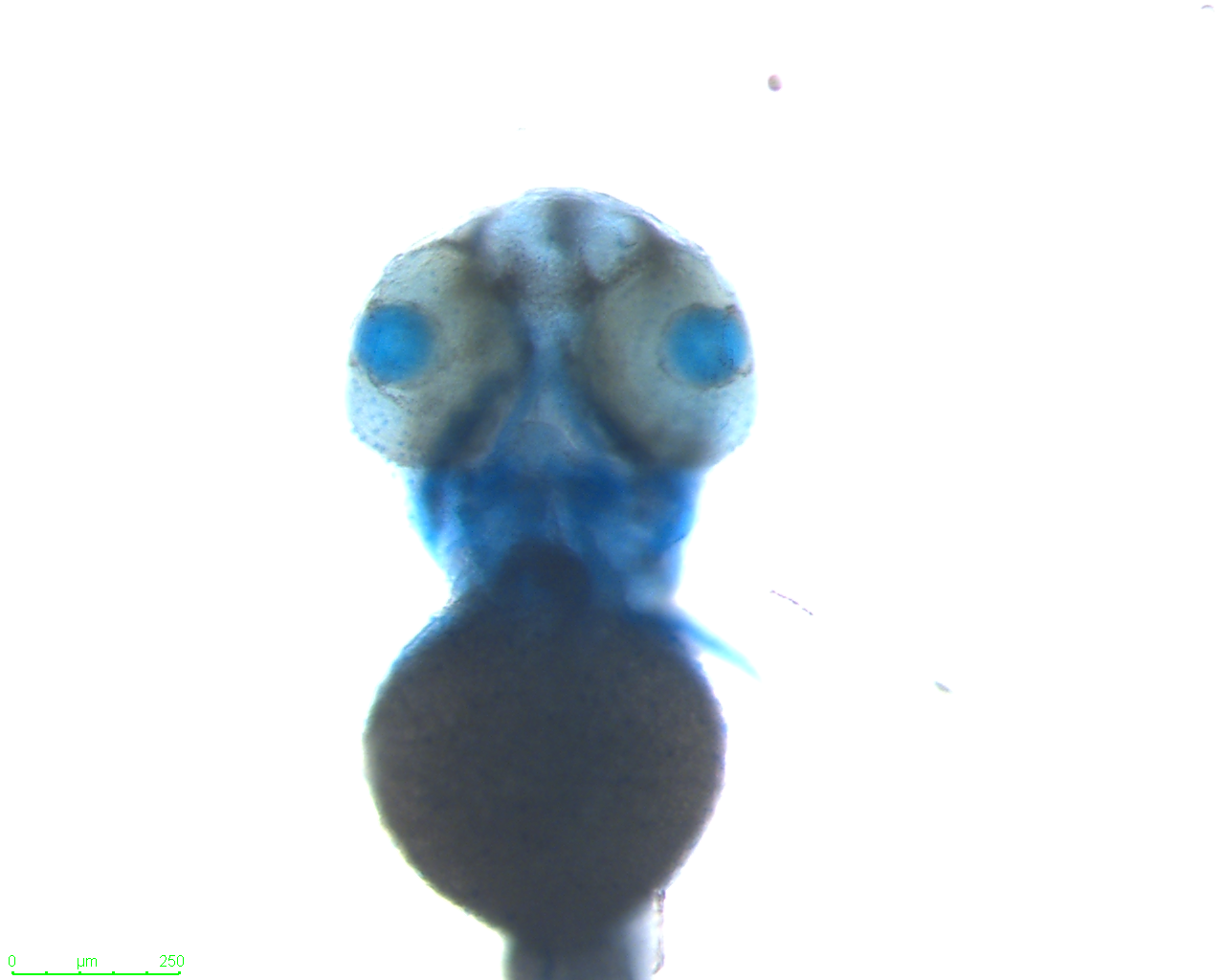

Supplement: Supplementary file 6 — Source Data [file 41467_2021_21053_MOESM6_ESM.zip › Source Data/Zebrafish Morpholino work/Third replicate/EIF5A images 090219_Control_Sperm_13.tif]

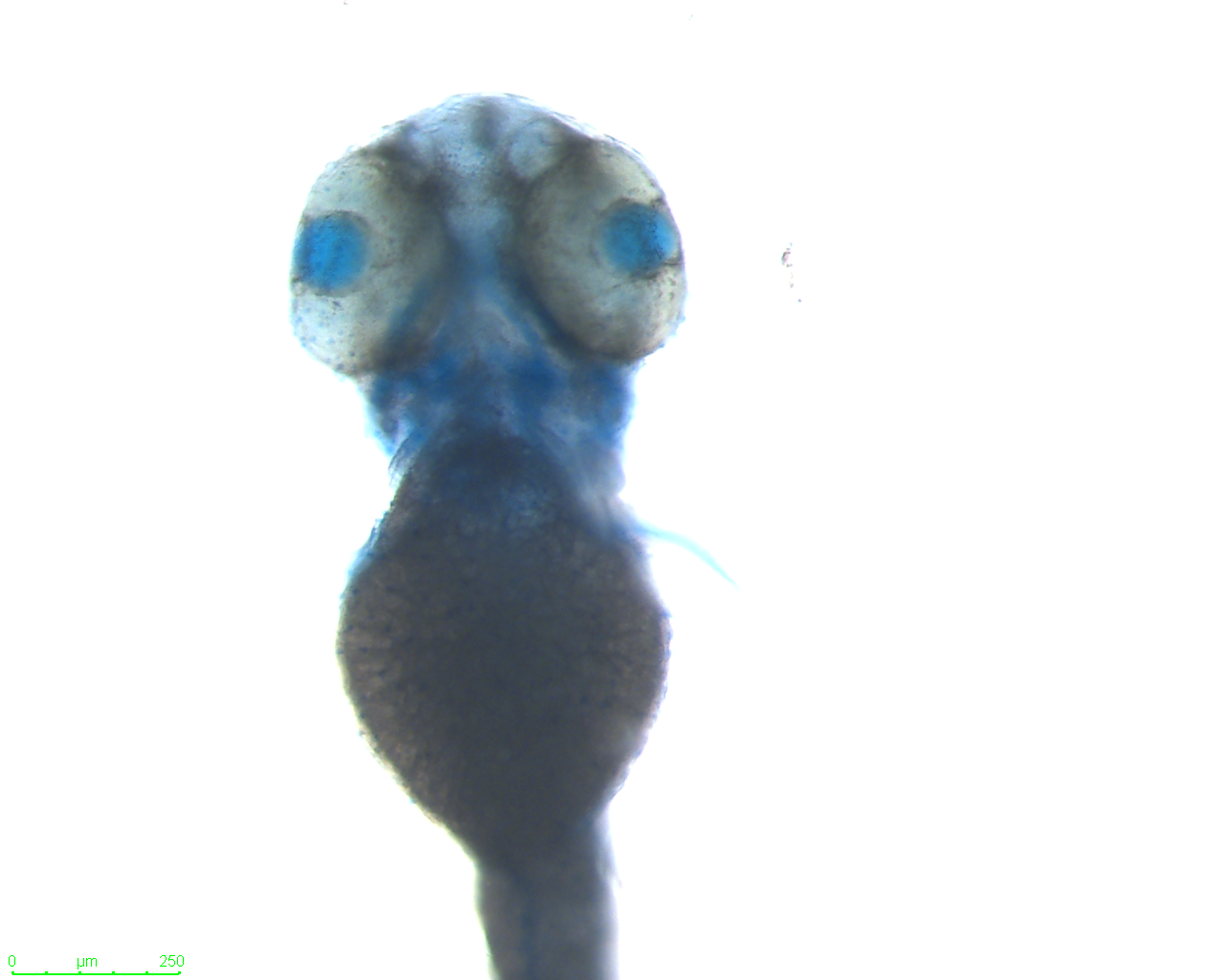

Supplement: Supplementary file 6 — Source Data [file 41467_2021_21053_MOESM6_ESM.zip › Source Data/Zebrafish Morpholino work/Third replicate/EIF5A images 090219_Control_Sperm_14.tif]

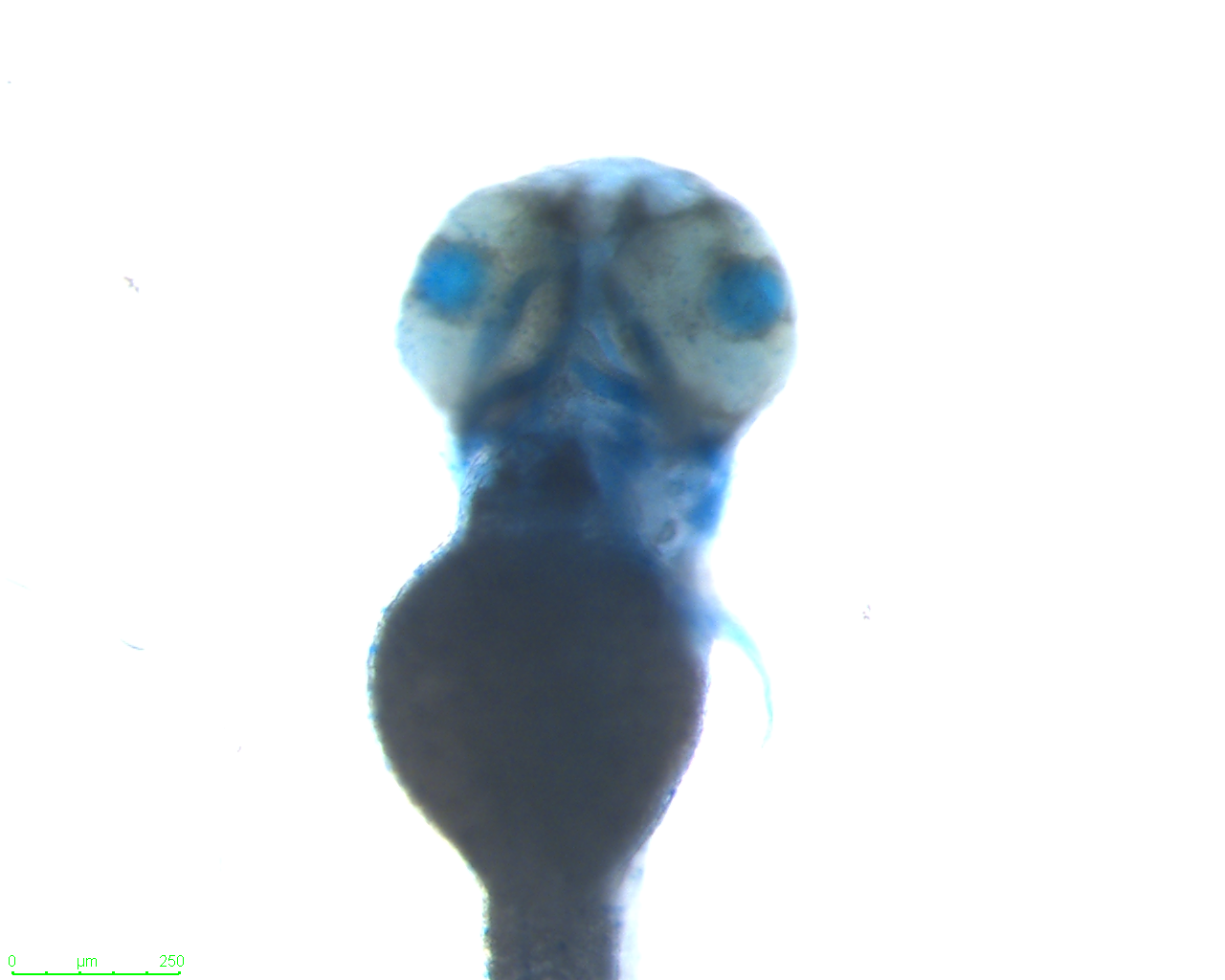

Supplement: Supplementary file 6 — Source Data [file 41467_2021_21053_MOESM6_ESM.zip › Source Data/Zebrafish Morpholino work/Third replicate/EIF5A images 090219_Control_Sperm_15.tif]

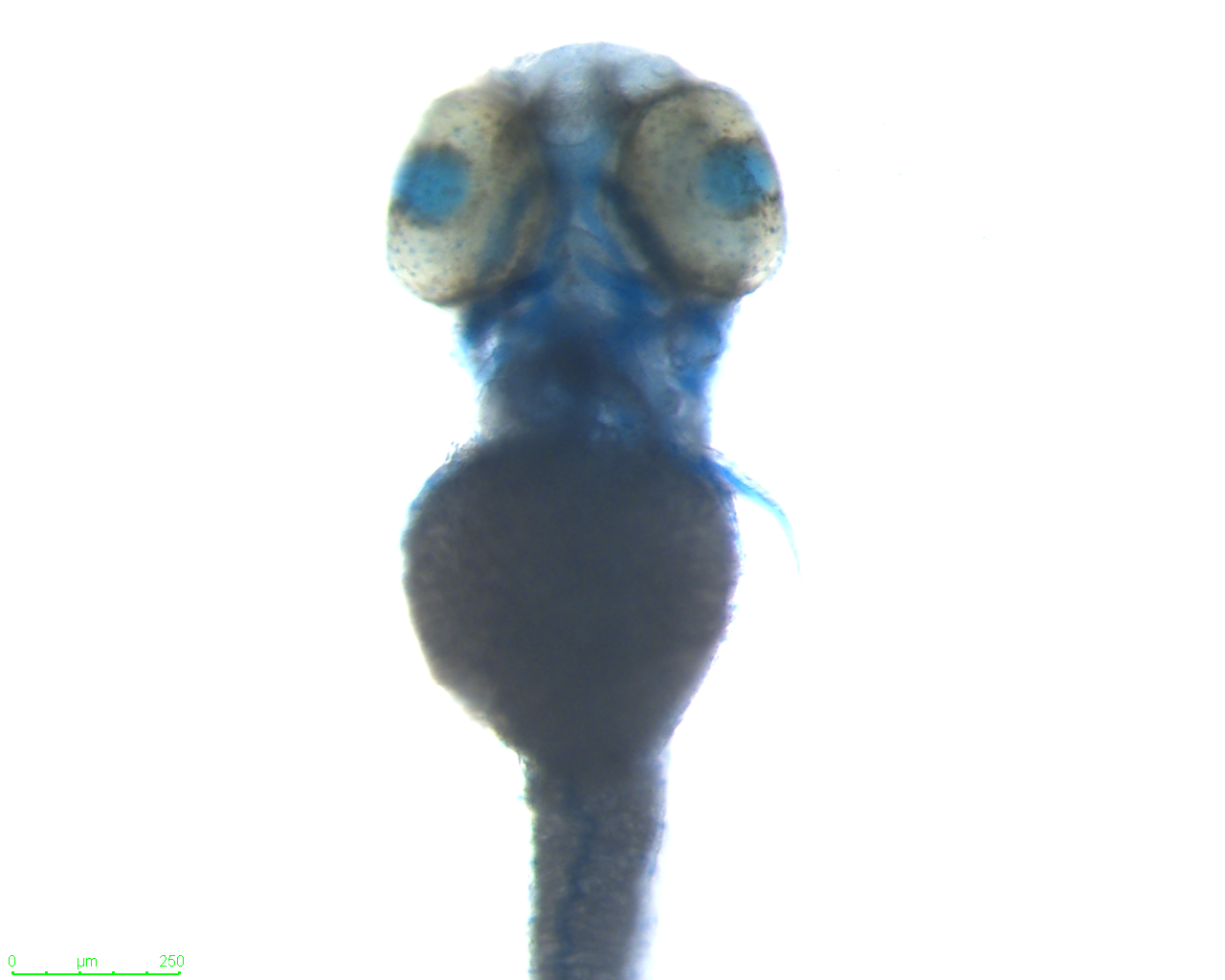

Supplement: Supplementary file 6 — Source Data [file 41467_2021_21053_MOESM6_ESM.zip › Source Data/Zebrafish Morpholino work/Third replicate/EIF5A images 090219_Control_UNT_01.tif]
